# Clinical pharmacokinetics of metronidazole: a systematic review and meta-analysis

**DOI:** 10.1128/aac.01904-24

**Published:** 2025-07-31

**Authors:** Iqra Shahzad, Mohammed S. Alasmari, Ammara Zamir, Muhammad Fawad Rasool, Faleh Alqahtani

**Affiliations:** 1 Department of Pharmacy Practice, Faculty of Pharmacy, Bahauddin Zakariya University 511766, Multan, Pakistan; 2 General Directorate of Medical Services, Security Forces Hospital Program, Ministry of Interior 48061, Riyadh, Saudi Arabia; 3 Department of Pharmacology and Toxicology, College of Pharmacy, King Saud University 108788, Riyadh, Saudi Arabia; 4 King Salman Center for Disability Research 600567, Riyadh, Saudi Arabia; Providence Portland Medical Center, Portland, Oregon, USA

**Keywords:** metronidazole, pharmacokinetics, *C*_max_, AUC, clearance

## Abstract

Metronidazole (MTZ) is used in various clinical settings; however, its pharmacokinetics may vary across patient populations due to physiological and pathological differences. Understanding these variations is important for personalized dosing and optimization of therapeutic outcomes. This study aimed to systematically review clinical pharmacokinetic studies of MTZ and perform a meta-analysis of the area under the concentration-time curve (AUC). AUC was selected for meta-analysis as it provides a direct and comprehensive measure of total drug exposure over time, facilitating standardized comparisons across populations. Four databases, including PubMed, ScienceDirect, Cochrane Library, and Google Scholar, were screened for pharmacokinetic studies on MTZ using systematic search strategies until July 2024. Out of 1,882 articles identified in the literature search, only 67 studies that fulfilled eligibility criteria were included in this systematic review. A meta-analysis of AUC was performed using random-effects models, with heterogeneity assessed by *I*² statistic. Effect sizes (pooled AUC) were compared across populations and visually presented with their corresponding 95% confidence intervals. Meta-analysis revealed significant differences in AUC across populations, with substantial heterogeneity among studies. This study provides a comprehensive evaluation of the MTZ pharmacokinetic profile across diverse patient populations. The findings emphasize the importance of tailored dosing strategies and support evidence-based clinical decision-making for optimizing the safety and efficacy of MTZ.

## INTRODUCTION

Metronidazole (MTZ) is a synthetic antimicrobial drug of the Nitroimidazole family that was derived from azomycin and produced by the two genera, that is, Proteobacteria and Actinobacteria. It was introduced in 1959 (1, 2), and its uses against anaerobic infections were first published by Shinn in 1962 (3), followed by its approval from the U.S. Food and Drug Administration (FDA) in 1963 for various anaerobic bacteria and protozoa infections (4–7). It is used to treat trichomoniasis and vaginosis during pregnancy, infections after cesarean section, postpartum hemorrhage, and bowel surgery, Crohn’s disease, giardiasis, amoebiasis, dysentery, liver abscess, meningitis, endocarditis, dental, skin, bone, and joint infections (2, 4, 8–11). The off-label uses of MTZ include the treatment of pouchitis, balantidiases, animal bite infections, *Helicobacter pylori* ulcer, periodontitis, and tetanus (12). The exact mechanism of action of MTZ is still unknown, but it penetrates the bacterial cell membrane by passive diffusion, where ferredoxin reduces the compound’s nitro group to nitro radicals, causing DNA strand breakage resulting in cell death (13, 14). It is available in oral (PO), intravenous (IV), and topical (TOP) dosage forms (15).

MTZ belongs to the Biopharmaceutical Classification System class 1, being a highly soluble and permeable drug. It has excellent gastrointestinal (GIT) absorption with more than 90% oral bioavailability, and its elimination half-life (*t*_1/2_) ranges from 6 to 14 h (16). The plasma protein binding and volume of distribution of MTZ are 10–15% and 0.51–1.1 L/kg, respectively (17, 18). The liver metabolizes MTZ into two metabolites, that is, l-(2-hydroxy-ethyl)−2-hydroxymethyl-5-nitroimidazole (hydroxy metabolite) and 2-methyl-5-nitroimidazole-l-acetic acid (acid metabolite) (19, 20). Its major route of excretion is the kidney, which accounts for 60–80% of the total dose (18, 21).

MTZ has the chemical formula C_6_H_9_N_3_O_3_ and a molecular weight of 171.16 g/mol (22, 23). Its water solubility is 10 mg/mL at 25°C with the pK_a_ and log *P* values of 2.62 and −0.02, respectively (18, 24, 25). Various analytical techniques have been documented in the literature which includes high-performance liquid chromatography (HPLC), liquid chromatography-mass spectrometry (LCMS), high-performance liquid chromatography-ultraviolet spectroscopy (HPLC-UV), thin layer chromatography (TLC), ultra-performance liquid chromatography (UPLC), liquid chromatography (LC), polarography, densitometry, and bioassay (1, 26–32).

MTZ belongs to pregnancy risk category B and may permeate into the breast milk but would not harm the child. Furthermore, it can also cross the blood-brain barrier but rarely cause neurotoxicity (8, 33, 34). The common adverse effects of MTZ include GIT problems, unpleasant taste, tongue furrowing, dizziness, lethargy, minor skin rashes, neutropenia, and disulfiram-like reaction with alcohol (9, 35). It is contraindicated in patients with Cockayne syndrome and interacts with alcohol, disulfiram, and warfarin (35, 36). According to a study, MTZ must always be administered with another broad-spectrum antibiotic to avoid resistance (37).

Although a few reviews on MTZ PK have been published (17, 38, 39), detailed and focused analyses specific to MTZ remain limited. This work provides new insights into MTZ’s pharmacokinetics (PK) profile by comprehensively evaluating data from various studies involving healthy individuals, patients with diverse conditions, and special populations. The study systematically reviewed clinical PK data of MTZ across different patient populations and performed a meta-analysis of the AUC to characterize MTZ exposure. By analyzing data from IV, oral, rectal, and vaginal administration routes, this study identified gaps in the existing literature to guide the development of PK models. These findings aim to support personalized treatment strategies and optimize dosing regimens for improved therapeutic outcomes.

## MATERIALS AND METHODS

### Study design and search strategy for literature screening

**Fig 1:**
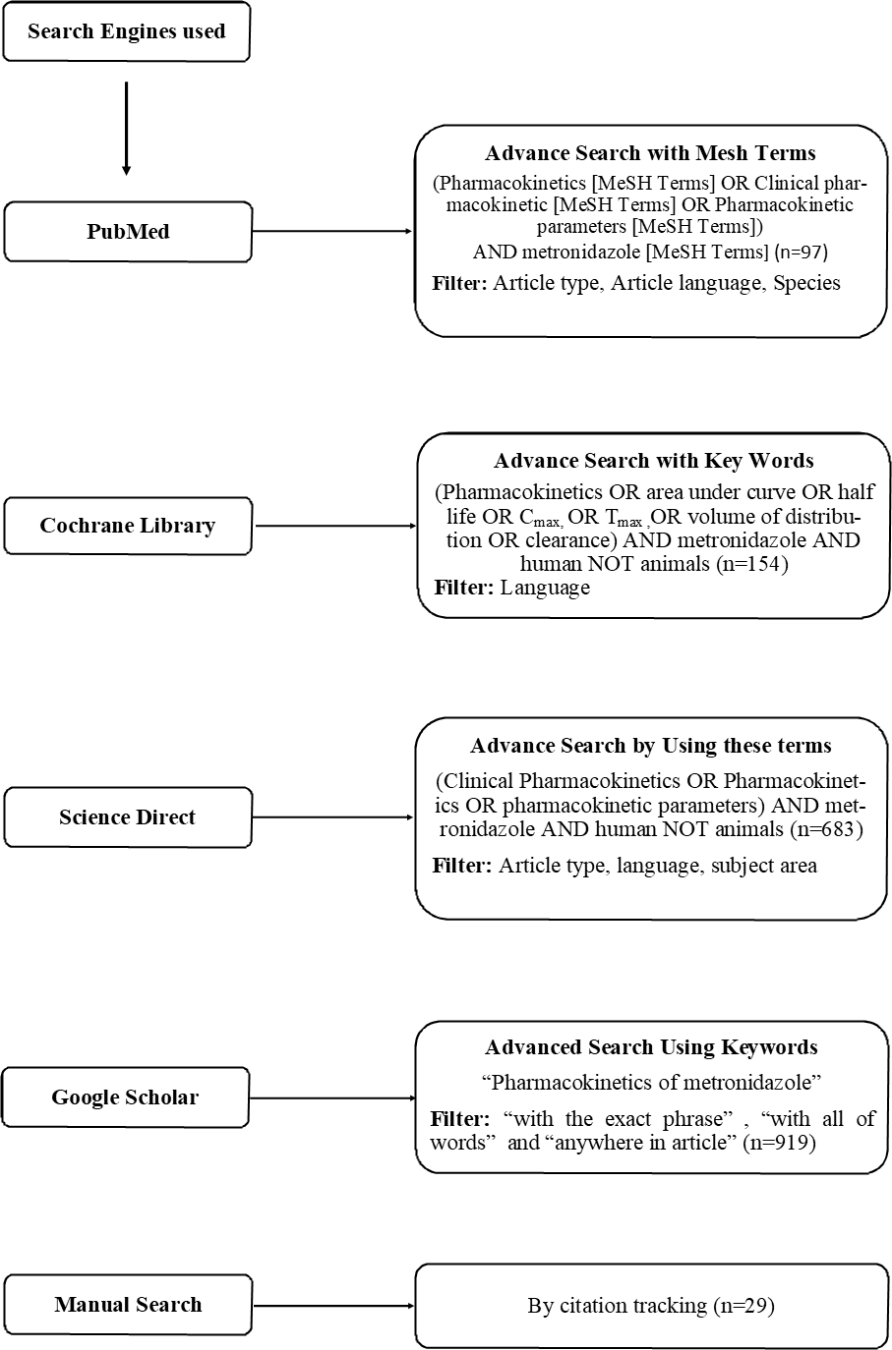
Literature search strategy.

Following the Preferred Reporting Items for Systematic Reviews and Meta-Analysis (PRISMA) (40) and Cochrane Handbook guidelines (41), the current systematic review was conducted. The published studies on the PK of MTZ were retrieved using Google Scholar, PubMed, Science Direct, Cochrane Library, and citation tracking after a thorough literature review until July 2024. In addition, a Boolean strategy, mesh terms, keywords, and free-text terms were employed to narrow the literature search. The search was conducted using the following terms: PK, clinical PK, pharmacokinetic parameters, area under concentration-time curve (AUC), half-life (*t*_1/2_), maximum plasma concentration (*C*_max_), time required to reach maximum plasma concentration (*T*_max_), volume of distribution (*V*_d_), clearance (Cl), MTZ, and human. Figure 1 details all the applied search methodologies.

### Eligibility criteria (inclusion/exclusion)

All relevant studies extracted through a detailed literature review were then exported to EndNote version 20, and the duplicates were removed using the option “find duplicates.” The retrieved articles underwent screening based on the titles, abstracts, animals, non-accessibility, books, language, thesis, and irrelevance. Moreover, conference abstracts, short reviews, letters to the editor, and commentaries were also excluded. After meeting the eligibility criteria, full-text reading was used to screen the articles further. The details of all excluded articles are given in Table S1. The original research articles presenting PK parameters of MTZ in healthy, diseased, and special populations were included. These inclusions were made regardless of dosage forms, dosing regimen, route of administration, and year of publication. Furthermore, all publications that reported drug-drug interactions and drug-food interactions were included to observe the effect of these variables on the PK of MTZ.

### Extraction of data

The required data extracted from eligible studies were as follows: authors, populations, number of participants, age of subjects, dosage form, route of administration, drug used, regimen (dose and frequency), and assay method (see Table 1). Additionally, the PK data included volume of distribution (*V*_d_), elimination half-life (*t*_1/2_), the area under concentration-time curve from 0 to ∞ (AUC_0–∞_), renal clearance (Cl_R_), total body clearance (Cl_T_), maximum plasma concentration (*C*_max_), and time required to reach maximum plasma concentration (*T*_max_). For uniformity, the units of PK parameters were changed to standard units so that readers could more effectively compare the results. Moreover, the data screening and extraction were carried out by two independent reviewers, that is, Muhammad Fawad Rasool and Ammara Zamir.

**TABLE 1:** Characteristics of included studies^*e*^

Sr. No.	Authors	Population	*N* ^*a*^	Age (years)	Route	Dosage form	Drugs used	Dose (mg)	Frequency	Method of assay
1	Fonnes et al. (1)	Patients with appendectomy	8	18–48	IP	Irrigation	MTZ	1,000	QD	HPLC
2	Sakurai et al. (26)	Healthy	12	20–35	PO	Tablet	MTZ	250	BID	LCMS
							AMOX	750		
							VPZ	20		
3	Visser et al. (42)	Pregnant women	16	≥18	IV	Infusion	MTZ	500	Single	HPLC
4	de C Bergamaschi et al. (6)	Healthy	13	18–30	PO	Tablet	MTZ	750	Single	HPLC
5	Somogyi et al. (19)	Renal dysfunction	6	23–75	IV	Infusion	MTZ	500	BID	HPLC
6	Houghton et al. (7)	Renal dysfunctions	32	18–60	IV	Infusion	MTZ	500	Single	HPLC
7	Shaffer et al. (10)	Patients with enteric disease	12	≥18	PO	Tablet	MTZ	400	Single	HPLC
8	Lau et al. (20)	Hepatic dysfunction	8	31–75	IV	Infusion	MTZ	7.5^*d*^	Single	HPLC
9	Passmore et al. (9)	Nursing mothers and infants	12	≥18	PO	Tablet	MTZ	400	TID	HPLC
10	Plaisance et al. (43)	Severely ill patients	14	32–86	IV	Infusion	MTZ	500	Single	HPLC
11	Heisterberg and Branebjerg (44)	Nursing mothers and infants	25	≥18	PO	Tablet	MTZ	400	TID	HPLC
12	Daneshmend et al. (45)	Hepatic dysfunction	25	20–67	PO	Tablet	MTZ	500	Single	HPLC
13	Ti et al. (46)	Patients with anaerobic infection	54	21–90	IV	Infusion	MTZ	500	TID	HPLC
14	Loft et al. (47)	Elderly	19	30–86	IV	Infusion	MTZ	500	Single	HPLC
15	Jensen and Gugler (48)	Healthy	7	19–31	IV	Infusion	MTZ	400	Single	HPLC
					PO	Tablet			Single	
					PO	Tablet			BID	
16	Dilger et al. (49)	Healthy	12	18–30	PO	Tablet	MTZ	750	BID	LCMS
							BDS	3		
17	Muscará et al. (50)	Liver dysfunction	52	≥18	IV	Infusion	MTZ	500	Single	HPLC
18	Ashiq et al. (51)	Patients with amoebiasis	12	25–35	PO	Tablet	MTZ	400	Single	HPLC
19	Thiercelin et al. (52)	Patient undergoing GIT surgery	17	42–73	IV	Infusion	MTZ	500	TID	HPLC
					PO	Tablet				
20	Guay et al. (53)	Renal patients on dialysis	10	29–68	IV	Infusion	MTZ	750	Single	HPLC
21	Loft et al. (54)	Hepatic encephalopathy	16	50–81	IV	Infusion	MTZ	500	Single	HPLC
22	Amon et al. (27)	Female children with vaginitis	20	6^*b*^–13	PO	Tablet	MTZ	10–20^*d*^	BID	Polarography
23	Dorn et al. (28)	Obese	30	21–65	IV	Infusion	MTZ	500	Single	HPLC-UV
24	Karjagin et al. (55)	Patients with septic shock	6	32–69	IV	Infusion	MTZ	500	Single	HPLC
25	Montalli et al. (56)	Smokers	26	12–24	PO	Tablet	MTZ	750	Single	HPLC-UV
26	Eradiri et al. (57)	Crohn’s disease	6	25–62	PO	Tablet	MTZ	250	TID	HPLC
27	Sachwarts et al. (29)	Healthy men	4	24–27	PO	Tablet	MTZ	75	Single	TLC
28	Amon et al. (58)	Pregnant women	19	29 ± 9	PO	Tablet	MTZ	1,000	Single	HPLC
								250		
29	Bergan et al. (30)	Patients with enteric disease	34	30–62	PO	Tablet	MTZ	500	Single	TLC-densitometry
30	Kim and Park (59)	Healthy	12	22–30	PO	Tablet	MTZ	500	TID	HPLC
							FEXO	120		
31	Hanifah and Mustofa (60)	Healthy men and women	12	20–40	PO	Tablet	MTZ	500	Single	HPLC-UV
32	Lares-Asseff et al. (61)	Malnourished children	20	3–43^*c*^	PO	Suspension	MTZ	30^*c*^	Single	HPLC
33	Ljungberg et al. (62)	Severely ill patients	11	32–74	IV	Infusion	MTZ	500	Single	HPLC
34	Farre et al. (63)	Hepatic dysfunction	19	33–68	PO	Infusion	MTZ	8^*c*^	Single	HPLC
35	Loft et al. (64)	Healthy	6	26–39	PO	Tablet	MTZ	250	BID	HPLC
							Cimetidine	200	TID	
36	Somogyi et al. (65)	Renal failure	6	18–60	IV	Infusion	MTZ	500	Single	HPLC
37	Kurji et al. (66)	Diabetics	24	55 ± 10	PO	Tablet	MTZ	500	Single	HPLC
38	Jager-Roman et al. (67)	Infants	11	28–40^*b*^	IV	Infusion	MTZ	7.5^*c*^	Single	HPLC
39	Das et al. (31)	Healthy	27	18–50	IV	Infusion	MTZ	500	QD	UPLC
							AVI	500		
							CAZ	2,000		
40	David et al. (68)	Healthy	14	19–35	PO	Tablet	MTZ	400	Single	HPLC
						Capsule	OMEP	20	BID	
41	Goddard et al. (69)	Healthy	24	19–37	IV	Infusion	MTZ	500	Single	Bioassay
					PO	Capsule	OMEP	40	BID	
42	Calafatti et al. (70)	*H. pylori*^+^ patients	28	19 ± 35	IV	Infusion	MTZ	500	Single	HPLC-LC
					PO	Capsules	OMEP	20	BID	
43	Houghton et al. (71)	Healthy	19	19–50	IV	Infusion	MTZ	500	Single	HPLC
					PO	Tablet				
44	Obodozie et al. (32)	Healthy	11	20–45	PO	Tablet	MTZ	400	Single	LC
							NIPRD-AM1	500		
45	Rajnarayana et al. (72)	Healthy	12	20–30	PO	Tablet	MTZ	800	Single	HPLC
							Diosmin	500	QD	
46	Rajnarayana et al. (73)	Healthy	12	23–30	PO	Tablet	MTZ	400	TID	HPLC-LC
							SIL	140	QD	
47	Melande et al. (74)	Healthy	10	25–35	PO	Tablet	MTZ	400	Single	Bioassay
48	Wang et al. (75)	Pregnant women	20	≥18	PO	Tablet	MTZ	250	BID	HPLC
49	Wang et al. (76)	Healthy	10	22–27	PO	Tablet	MTZ	400	BID	HPLC
							MDZ	15	Single	
50	Pierce et al. (77)	Healthy	29	18–55	PO	Tablet	MTZ	750	BID	LCMS
							MMX	4,800	Single	
51	Jessa et al. (78)	Healthy	8	19–25	IV	Infusion	MTZ	500	Single	HPLC
					PO	Capsule	OMEP	40	BID	
52	Mattila et al. (79)	Healthy	5	22–23	IV	Infusion	MTZ	500	Single	HPLC
					PO	Tablet		500		
					PR	SUP		1,000		
					VAG	Pessary		500		
53	Mattie et al. (80)	Patients with mixed infection	15	16–76	PO	Tablet	MTZ	500	TID	HPLC
54	Ventura Cerdá et al. (81)	Patient undergoing Colorectal Surgery	36	>18	IV	Infusion	MTZ	1,500	Single	HPLC
55	Hamberg et al. (82)	Healthy	9	21–29	PO	Tablet	MTZ	500	Single	HPLC
56	Sprandel et al. (83)	Healthy	18	18–45	IV	Infusion	MTZ	500	Single	HPLC
								1,000		
								1,500		
57	Wu et al. (84)	Healthy	12	23–30	PO	Tab	MTZ	125	Single	LCMS
58	Salas-Herrera et al. (85)	Healthy	12	22–31	VAG	Pessary	MTZ	500	Single	HPLC
59	Bergan and Arnold (15)	Healthy	10	17–29	PR	Suppository Tab	MTZ	500	Single	HPLC
								1,000		
								2,000		
60	Fredricsson et al. (86)	Healthy	5	21–24	PO	Tab	MTZ	400	Single	HPLC
61	Roux et al. (87)	Renal impairment	9	58–79	IV	Infusion	MTZ	525	Single	HPLC
62	Houghton et al. (88)	Healthy	3	21–33	PO	Tab	MTZ	400	Single	HPLC
63	Loft et al. (89)	Healthy	8	30 ± 6	IV	Infusion	MTZ	2,000	Single	HPLC
64	Cunningham et al. (90)	Healthy	12	24–35	PO	Tab	MTZ	500	Single	HPLC
65	Lau et al. (91)	Healthy	9	≥18	IV	Infusion	MTZ	2,000	Single	HPLC
66	Amon et al. (92)	Healthy	27	≥18	PO	Tab	MTZ	250	Single	Polar
								1,000		
67	Alper et al. (93)	Healthy	9	≥18	VAG	Cream	MTZ	500	Single	HPLC

^*a*^ number.

^*b*^ weeks.

^*c*^ months.

^*d*^ mg/kg.

^*e*^ ALP: alprazolam, AMOX: amoxicillin, AVI: avibactam, BDS: budesonide, BID: twice a day, CAZ: ceftazidime, FEXO: fexofenadine, HPLC: high-performance liquid chromatography, IP: Intraperitoneal, IV: intravenous, LC: liquid chromatography, LCMS: liquid chromatography-mass spectrometry, LRP: lorazepam, MDZ: midazolam, MMX: mesalamine, MTZ: metronidazole, OMEP: omeprazole, PHT: phenytoin, PO: per oral, PR: per rectal, QD: every day, QID: four times a day, SIL: silymarin, TID: thrice a day, TLC: thin layer chromatography, UPLC: ultra-performance liquid chromatography, UV: ultraviolet spectroscopy, VAG: Intravaginal, VPZ: vonoprazan.

### Quality assessment of selected literature

First, the quality of the retrieved studies was evaluated by the Jadad tool, a 5-item checklist, by checking the presence of randomization, blinding, and dropping out information of participants. Studies were considered high, moderate, and low quality if they scored >4, 3–4, and <3, respectively (94, 95). After that, studies were screened by using the Critical Appraisal Skills Programme (CASP), a 10-item checklist, to check the transparency of research practice, wherein a score of >6 shows high quality, 4–6 suggests moderate quality, and <4 indicates low quality (96, 97). Then the quality of included studies was assessed by using a 21-item checklist, the Critical Appraisal Clinical Pharmacokinetic Tool (CACPK), according to which studies were categorized as high, fair to moderate, and low-quality studies with scores >13, 12–13, and <12, respectively (98). The risk of bias was additionally assessed with the Cochrane Collaboration Tool (CCT), and according to this, studies with scores of <3, 3‒4, and >4 were classified as having a high, moderate, and low risk of bias, respectively (99, 100).

### Statistical analysis

The meta-analysis was conducted by using R programming language and employing the “metafor” package (101). The random-effect model was used in this meta-analysis, and heterogeneity was assessed with the I-squared statistic (*I*²) (102). For the visual display of individual studies and pooled results, the forest plots were constructed. Not all studies were considered in the meta-analysis. Only those studies that provided the required quantitative information, such as sample size, mean, and standard deviation (SD) of AUC were included. The studies with insufficient data were only evaluated qualitatively in the systematic review.

## RESULTS

### Results of the literature review

**Fig 2:**
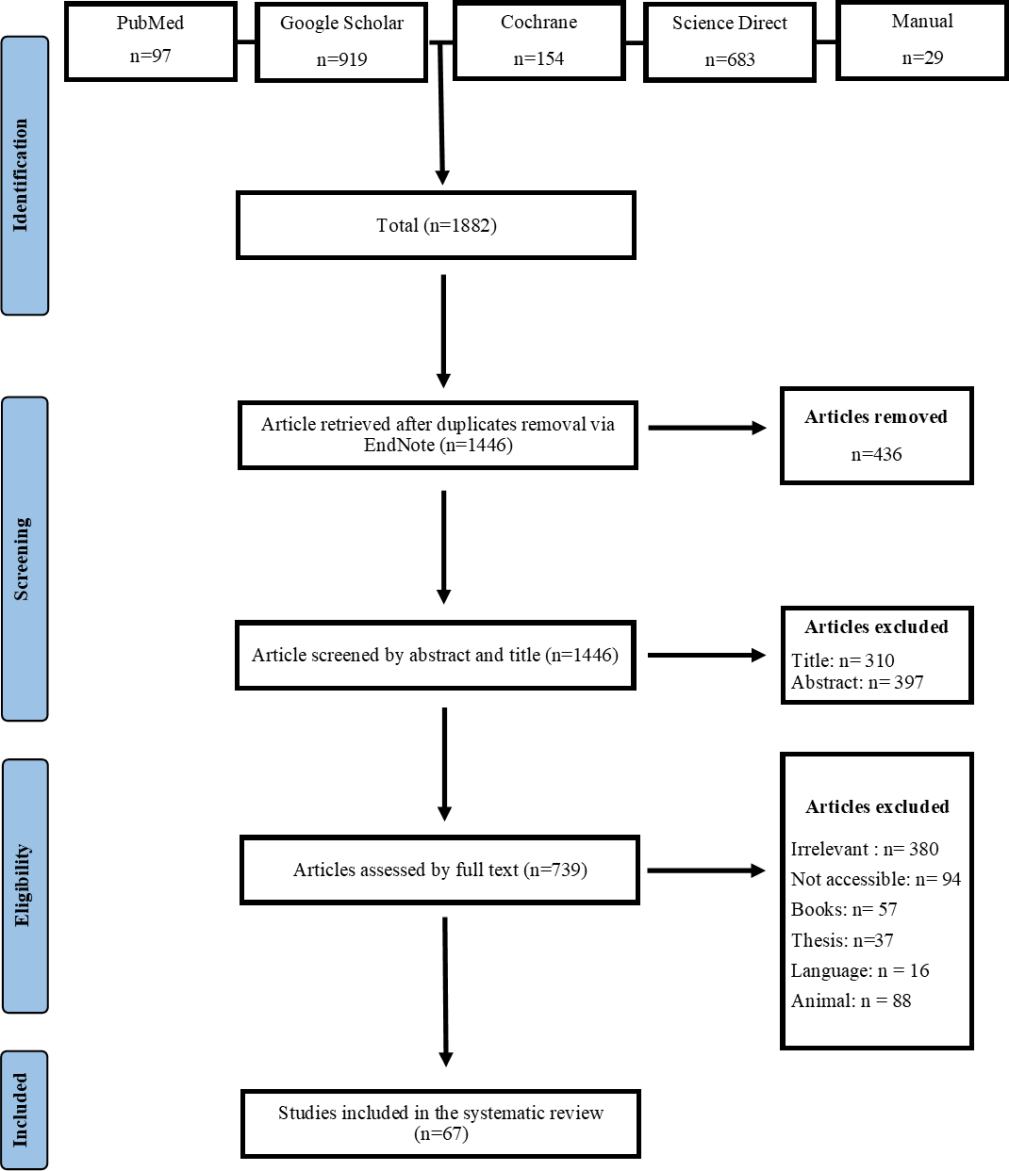
PRISMA flow diagram.

A total of 1,882 studies were identified after an extensive database search, of which 436 were duplicates. The remaining 1,446 studies were further screened; as a result, 67 were included in this review, and 1,379 that did not meet eligibility criteria were excluded. The details are presented in Fig. 2.

### Characteristics of finalized studies

The characteristics of included studies are reported in Table 1 covering authors' names, population, number and age of participants, drug used, dosage form, dose and frequency, mode of administration, and assay technique.

### Quality evaluation of included literature

The quality of 67 studies was assessed by the Jadad scoring tool, CASP, CACPK, and CCT. According to Jadad scoring, 62 studies were of low quality and 5 studies were of moderate quality (Table S2). Similarly, according to the CASP, 3 studies were of moderate quality and 64 studies were of high quality (Table S3). The CACPK enlisted 3 studies of fair-moderate quality and 64 studies of high quality (Table S4). The risk of bias was high, moderate, and low for 9, 35, and 23 studies, respectively, according to the CCT (Table S5).

### PK of MTZ in healthy population

#### IV administration of MTZ

Out of total 67 studies reviewed, 9 were conducted in a healthy population after IV administration of MTZ. The reported AUC_0–24_ and *t*_1/2_ in a study were 81.4 ± 27 µg.h/mL and 7.4 ± 2.2 h, respectively, following 500 mg IV administration of MTZ (50). One study has stated a decrease in Cl_R_ among the elderly population compared to the young individuals, that is, 87 ± 15 mL/min versus 73 ± 30 mL/min (47). In a research study, the reported *C*_max_ in saliva and plasma were 11.8 ± 7.9 µg/mL and 11.3 ± 2.06 µg/ml, respectively (6). The reported Cl_T_ was 72 ± 16 and 80.1 ± 5.2 mL/min in two studies after administering a dose of 500 mg (50, 79). Another study has depicted higher AUC_0–∞_ via the IV route in comparison to the oral one, that is, 159 ± 48 and 151 ±42 ug.h/mL, respectively (71). An increase in *C*_max_ from 22.2 ± 5.0 to 37.7 ± 10 µg/mL was recorded in a study when a dose of MTZ was increased from 500 to 1,500 mg (83). Other PK parameters are given in Table 2.

**TABLE 2:** Pharmacokinetic parameters of metronidazole in healthy population^*g*^

Sr. No.	Authors	Population		ROA	*V*_d_ (L)	*t*_1/2_ (h)	AUC_0–∞_ (µg·h/mL)	Cl_T_ (mL/min)	Cl_R_ (mL/min)	C_max_ (µg/mL)	*T*_max_ (h)
1	Houghton et al. (7)	Healthy		IV	43 ± 6.1	7.0 ± 0.80	123 ± 35	72 ± 16	6.8 ± 2.5	N/M	N/M
2	Muscará et al.. (50)	Healthy		IV	0.80 ± 0.32^*a*^	7.4 ± 2.2	81.4 ± 27^*d*^	1.53 ± 0.37^*b*^	N/M	N/M	N/M
3	Ljungberg et al. (62)	Healthy		IV	N/M	6.1	85.0	117.1^*c*^	N/M	N/M	N/M
4	Loft et al. (47)	Healthy	Elder	IV	47 ± 16	7.8 ± 1.9	N/M	1.20 ± 0.53^*c*^	73 ± 30	N/M	N/M
			Young		54 ± 6	7.2 ± 0.9	N/M	1.25 ± 0.22^*c*^	87 ± 15	N/M	N/M
5	Jensen and Gugler (48)	Healthy		IV	1.052 ± 0.14^*a*^	8.3 ± 0.4	81.61 ± 8.12	1.306 ± 0.1^*c*^	N/M	N/M	N/M
6	Houghton et al. (71)	Healthy		PO	36 ± 7.9	7.3 ± 1.0	151 ± 42	N/M	N/M	N/M	N/M
				IV	34 ± 8.2	7.0 ± 1.1	159 ± 48	N/M	N/M	N/M	N/M
7	Mattila et al. (79)	Healthy		IV	53.2 ± 1.2	7.9 ± 0.6	106.9 ± 10.7	80.1 ± 5.2	N/M	9.4 ± 0.5	N/M
8	Loft et al. (89)	Healthy		IV	50 ± 8	7.7 ± 1.7	447 ± 67	74 ± 12	N/M	N/M	N/M
9	Sprandel et al. (83)	Healthy	500	IV	49.2 ± 12	8.0 ± 1.3	356 ± 68^*f*^	62 ± 7	11 ± 3	22.2 ± 5.0	N/M
			1,000		59 ± 10	9.2 ± 1.5	227 ± 57^*f*^	67 ± 13	12 ± 2	24.8 ± 6.8	N/M
			1,500		62.5 ± 11	9.8 ± 1.5	338 ± 105^*f*^	67 ± 14	12 ± 5	37.7 ± 10	N/M
10	Lau et al. (91)	Healthy	2,000	IV	250	0.67^*a*^	6.0 ± 1.3	48.4 ± 19.3	N/M	N/M	N/M
			250		2,000	0.58^*a*^	8.8 ± 1.2	532.2 ± 104.3	N/M	N/M	N/M
11	Ashiq et al. (51)	Healthy		PO	0.8544 ± 0.09^*^	7.782 ± 0.78	103.17 ± 6.25	1.27 ± 0.13^*b*^	N/M	8.041 ± 0.4	1.7 ± 0.06
12	Farre et al. (63)	Healthy		PO	0.48 ± 0.03^*a*^	7.9 ± 0.7	N/M	51.9 ± 8.7	N/M	N/M	N/M
13	Bergan et al. (30)	Healthy	250	PO	N/M	9.99 ± 6.11	47.40 ± 16.57	N/M	N/M	N/M	N/M
			500		N/M	9.67 ± 1.79	113.54 ± 35.45	N/M	N/M	N/M	N/M
			1,000		N/M	9.31 ± 2.15	214.04 ± 35.45	N/M	N/M	N/M	N/M
14	Kurji et al. (66)	Healthy		PO	N/M	10.03 ± 2.57	50.30 ± 10.73	N/M	N/M	7.58 ± 0.38	1.45 ± 0.1
15	de C Bergamaschi et al. (6)	Healthy	Plasma	PO	0.625 ± 0.19^*a*^	N/M	210.2 ± 45.69	62.5 ± 15.54	N/M	11.3 ± 2.06	1.77 ± 0.7
			Saliva		N/M	N/M	124.2 ± 64.10	N/M	N/M	11.8 ± 7.9	1.50 ± 0.5
16	Jensen and Gugler (48)	Healthy	SOD	PO	0.960 ± 0.08^*a*^	8.4 ± 0.4	79.97 ± 6.73	N/M	N/M	6.9 ± 0.9	2.3 ± 0.6
			MOD		N/M	8.3 ± 0.4	82.31 ± 12.65^*e*^	N/M	N/M	11.2 ± 1.6	2.1 ± 0.3
17	Montalli et al. (56)	Healthy	Smoker	PO	1.071 ± 0.56^*a*^	N/M	297.2 ± 228.4	N/M	0.91 ± 0.5	9.7 ± 2.9	2.9 ± 1.8
			Non-smoker		0.809 ± 0.26^*a*^	N/M	29.3 ± 114.0	N/M	0.7 ± 0.2	11.9 ± 2.0	1.8 ± 0.9
18	Schwartz et al. (29)	Healthy	Males	PO	83.03 ± 11.98	8.41 ± 1.52	N/M	N/M	N/M	12.3	1
19	Hanifah and Mustofa (60)	Healthy	Males	PO	79.49 ± 5.678	11.299 ± 0.52	103.81 ± 5.14	N/M	N/M	10.36 ± 1.3	0.67 ± 0.1
		Healthy	Females		64.67 ± 9.54	7.41 ± 1.24	84.36 ± 10.48	N/M	N/M	6.89 ± 0.39	0.9 ± 0.24
20	Houghton et al. (71)	Healthy		PO	34 ± 8.2	7.0 ± 1.1	159 ± 48	N/M	N/M	N/M	N/M
21	Mattila et al. (79)	Healthy		PO	N/M	8.9 ± 0.6	122.2 ± 10.3	N/M	N/M	9.0 ± 0.5	1.9 ± 0.2
22	Daneshmend et al. (45)	Healthy	British	PO	0.60 ± 0.01^*a*^	7.4 ± 0.3	114.6 ± 7.8	1.06 ± 0.04^*b*^	N/M	N/M	N/M
		Healthy	Sudanese		0.76 ± 0.07^*a*^	7.8 ± 0.6	132.2 ± 22.0	1.21 ± 0.16^*b*^	N/M	N/M	N/M
23	Dorn et al. (28)	Healthy	Obese	PO	73.9 ± 10.3	11.9 ± 3.4	115.8 ± 29.0	77 ± 20.3	N/M	8.99 ± 1.05	0.5
		Healthy	Non-obese		51.8 ± 9.7	9.08 ± 1.85	126.9 ± 29.6	68.83 ± 14.5	N/M	14.7 ± 4.1	0.5
24	Fredricsson et al. (86)	Healthy		PO	0.65 ± 0.05^*a*^	7.3 ± 0.3	107 ± 10.3	N/M	N/M	N/M	N/M
25	Houghton et al. (88)	Healthy		PO	N/M	8.6 ± 1.2	82 ± 17	N/M	N/M	13 ± 2.8	0.61 ± 0.7
26	Cunningham et al. (90)	Healthy		PO	N/M	N/M	133 ± 43	N/M	N/M	12.7 ± 19.5	1.35 ± 0.6
27	Amon et al. (92)	Healthy	250	PO	0.62 ± 0.17^*a*^	5.92 ± 3.93	50.18 ± 29.69	N/M	N/M	N/M	N/M
			1,000		0.76^*a*^	7.8 ± 1.3	281.9 ± 75.4	72.5	N/M	19.6 ± 3.8	1
28	Salas-Herrera et al. (85)	Healthy	Day 1	VAG	N/M	N/M	6.6 ± 2.7^*e*^	N/M	N/M	0.9 ± 0.4	6
			Day 5		N/M	N/M	14.8 ± 6.9^*e*^	N/M	N/M	1.5 ± 0.7	4
29	Mattila et al. (79)	Healthy		VAG	N/M	N/M	31 ± 5	N/M	N/M	1.9 ± 0.2	7.7 ± 1.6
				PR	N/M	8.8 ± 0.4	129.8 ± 18.6	N/M	N/M	8.8 ± 1.1	2.8 ± 0.5
30	Bergan and Arnold (15)	Healthy	500	PR	N/M	10.45 ± 6.06	89.07 ± 24.17	N/M	N/M	5.5	4
			1,000		N/M	8.80 ± 2.23	187.68 ± 47	N/M	N/M	9.5	4
			2,000		N/M	9.46 ± 3.65	280.78 ± 109	N/M	N/M	14	4
31	Alper et al. (93)	Healthy		VAG	N/M	N/M	N/M	N/M	32.4 ± 38.4	1.9 ± 0.2	11.1 ± 1.6
				PO	N/M	N/M	N/M	N/M	142 ± 17.9	N/M	N/M

^*a*^ L/kg.

^*b*^ mL/min/kg.

^*c*^ mL/min × 1.73 m^2^.

^*d*^ AUC_0–24_.

^*e*^ AUC_0–12_.

^*f*^ mg.

^*g*^ SOD: single oral dose, MOD: multiple oral doses, VAG: Intravaginal, PR: per rectal, N/M: not mentioned, ROA: route of administration, V_d_: volume of distribution, t_1/2_:elimination half-life, AUC_0-∞_:area under concentration-time curve from 0–∞,Cl_T:_ total body clearance ,C_max_: maximum plasma concentration, T_max_: time to reach maximum plasma concentration, Cl_R_: renal clearance.

#### Oral administration of MTZ

Among all included studies, 16 were carried out in a healthy population following oral administration of MTZ. In one study, the *C*_max_ was reported to be 7.58 ± 0.38 µg/mL when 500 mg MTZ was delivered to healthy individuals (66). The Cl_T_ of MTZ was demonstrated in two studies, that is, 90.5 ± 43.67 and 51.9 ± 8.7 mL/min, respectively (30, 63). A gender-based difference in the AUC_0–∞_ was observed following oral administration of MTZ, that is, 103.81 ± 5.14 µg·h/mL in males and 84.36 ± 10.48 µg·h/mL in females (60). Additionally, compared to a single dose, repeated administration of MTZ resulted in a 1.6-fold increase in *C*_max_ (48). Furthermore, higher Cl_T_ was depicted among the obese population in comparison to non-obese subjects in a clinical study, that is, 77 ± 20.3 mL/min versus 68.83 ± 14.5 mL/min (28). A subsequent investigation has compared the PK parameters between smokers and non-smokers in which higher AUC_0–∞_ was seen in the former, that is, 297.2 ± 228.4 µg·h/mL versus 29.3 ± 114.0 µg·h/mL (56). One of the research studies has reported a *C*_max_ of 12.3 µg/mL after administering 75 mg MTZ to the male population (29). The details of all other PK parameters are presented in Table 2.

#### Other routes of administration of MTZ

A dose-dependent increase in AUC_0–∞_ was seen in a study following rectal administration of MTZ (15). A study following intravaginal administration of MTZ reported a *C*_max_ of 1.9 ± 0.2 µg/mL (79). Another study has presented a higher level of AUC_0–12_ on day 5 compared to day 1 after the intravaginal route, that is, 14.8 ± 6.9 and 6.6 ± 2.7 µg·h/mL, respectively (85). The additional parameters are presented in Table 2.

### PK of MTZ in diseased population

#### IV administration of MTZ

##### Renal impairment

Of the 67 included studies, 5 have presented PK data in renal patients after IV administration of MTZ. A study has reported Cl_R_ in patients with moderate and severe renal dysfunction, that is, 2.7 ± 1.4 and 2.4 ± 1.1 mL/min, respectively, while no traces of MTZ were detected in renal failure (7). Another study has demonstrated AUC_0–∞_ as 205.44 ± 41.84 g·h/mL in patients receiving continuous ambulatory peritoneal dialysis (CAPD) (53). Hemodialysis (HD) causes an increase of 3.1-fold Cl_T_ among patients suffering from chronic renal failure (65).

##### Hepatic impairment

A total of five studies were conducted in a population with hepatic impairment; among them, one study depicted Cl_T_ in patients with cirrhosis and schistosomiasis, that is, 0.56 ± 0.28 and 0.93 ± 0.19 mL/min/kg (50). In two clinical studies, the AUC_0–∞_was reported as 256.8 ± 56.3 and 175.7 µg·h/mL, respectively, in patients with liver dysfunction (20, 62). Following IV administration of 500 mg MTZ in patients with hepatic encephalopathy, the Cl_T_ was reported as 29 ± 10 mL/min (54). Moreover, in a research study, a 3.1-fold decrease in *C*_max_ of MTZ hydroxy-metabolite was demonstrated among patients with alcoholic liver disease (20).

##### Enteric disease

A study has reported a *C*_max_ of 34.7 ± 11.1 µg/mL in individuals who underwent colorectal surgery following an IV dose of 500 mg MTZ (81). In another study, a 1.4-fold increase in area under the concentration time curve at steady state (AUC)_ss_ was recorded when IV therapy was switched to the oral route of administration (52).

##### Infection

When MTZ was delivered intravenously to patients with anaerobic infection, the Cl_T_ and *t*_1/2_ were reported as 0.89 ± 0.3 mL/min/kg and 10.6 ± 4.5 h, respectively (46). A research study conducted in patients with septic shock has reported lower AUC_0–10_ in muscles compared to plasma, that is, 66 ± 8.3 versus 57.9 ± 29.9 µg·h/mL (55), as described in Table 3.

**TABLE 3:** Pharmacokinetic parameters of metronidazole in the diseased population^*g*^

Sr. no.	Authors	Population		ROA	*V*_d_ (L)	*t*_1/2_ (h)	AUC_0–∞_ (µg·h/mL)	Cl_T_ (mL/min)	Cl_R_ (mL/min)	C_max_ (µg/mL)	*T*_max_ (h)
1	Somogyi et al. (19)	Renal dysfunction		IV	45.5 ± 8.7	9.51	N/M	55.5 ± 17.7	1.43 ± 1.36	N/M	N/M
2	Houghton et al. (7)	Renal dysfunctions	Moderate	IV	36 ± 9.4	7.4 ± 2.4	159 ± 52	60 ± 20	2.7 ± 1.4	N/M	N/M
			HM		N/M	16 ± 4.5	131 ± 47	N/M	N/M	N/M	N/M
			Severe		48 ± 20	11 ± 5.7	180 ± 82	68 ± 59	2.4 ± 1.1	N/M	N/M
			HM		N/M	28 ± 36	136 ± 200	N/M	N/M	N/M	N/M
			RF		55 ± 21	7.2 ± 2.3	99 ± 27	91 ± 29	ND	N/M	N/M
			HM		N/M	34 ± 43	>79	N/M	N/M	N/M	N/M
3	Plaisance et al. (43)	Renal dysfunction		IV	0.98-0.68^*a*^	11.6-13	N/M	0.87-0.68^*b*^	N/M	N/M	N/M
		Severe ill patients			0.78-1.2^*a*^	7.98-42.4	N/M	0.28-1.7^*b*^	N/M	N/M	N/M
4	Guay et al. (53)	Renal patient on HD		IV	719.44 ± 14	8.16 ± 1.59	178.3 ± 17.4	N/M	N/M	N/M	N/M
		Renal patients on CAPD			754 ± 0.1^*a*^	10.93 ± 2.01	205.44 ± 41.8	N/M	N/M	N/M	N/M
5	Somogyi et al. (65)	Renal patient on HD		IV	37.9 ± 15.6	2.14	N/M	196.0 ± 60.6	N/M	N/M	N/M
6	Lau et al. (20)	Alcoholic liver disease		IV	0.77 ± 0.16^*a*^	18.31 ± 6.06	256.8 ± 56.3	0.51 ± 0.11^*b*^	N/M	N/M	N/M
		HM			N/M	24.99 ± 7.65	63 ± 21.5	N/M	N/M	1.66 ± 0.41	10 ± 8.7
7	Muscará et al. (50)	Child-Pugh A		IV	0.74 ± 0.11^*a*^	10.7 ± 2.3	124.9 ± 42.3	0.85 ± 0.26^*b*^	N/M	N/M	N/M
		Child-Pugh B			0.79 ± 0.12^*a*^	13.5 ± 5.1	124.4 ± 25.8	0.79 ± 0.36^*b*^	N/M	N/M	N/M
		Child-Pugh C			0.81 ± 0.14^*a*^	21.5 ± 12.7	174.1 ± 52^*d*^	0.56 ± 0.28^*b*^	N/M	N/M	N/M
		Patients with schistosomiasis			0.79 ± 0.09^*a*^	10.2 ± 2.1	135 ± 33.8^*d*^	0.93 ± 0.19^*b*^	N/M	N/M	N/M
8	Loft et al, (54)	Hepatic encephalopathy		IV	44 ± 9	20 ± 9	N/M	29 ± 10	N/M	N/M	N/M
		HM			N/M	N/M	113 ± 82	N/M	13.6 ± 13.1	N/M	30 ± 14
9	Ljungberg et al. (62)	Hepatic dysfunction		IV	N/M	11.3	175.7	50.7^*c*^	N/M	N/M	N/M
		Severe renal impairment			N/M	6.5	160.25 ± 25.8	N/M	N/M	N/M	N/M
10	Farre et al. (63)	Hepatic dysfunction		IV	0.38 ± 0.03^*a*^	19.9 ± 2.5	N/M	17.5 ± 2.4	N/M	N/M	N/M
11	Ti et al. (46)	Patients with anaerobic infection		IV	0.73 ± 0.1^*a*^	10.6 ± 4.48	N/M	0.89 ± 0.296^*b*^	N/M	N/M	N/M
12	Karjagin et al. (55)	Patients with septic shock	Plasma	IV	53.5 ± 4	13.2 ± 5.3	66 ± 8.3^*e*^	56.2 ± 26.9	N/M	11.4 ± 2	0.5
			Muscle		N/M	27.3 ± 23.4	57.9 ± 29.9^*e*^	N/M	N/M	8.2 ± 4.5	2.3 ± 1.5
13	Ventura Cerdá et al. (81)	Patient undergoing colorectal surgery		IV	0.68 ± 0.20^*a*^	N/M	N/M	52.5 ± 20	N/M	34.7 ± 11.1	N/M
14	Roux et al. (87)	Renal impairment		IV	20.2	6.82	122.5 ± 42	73.33	N/M	N/M	N/M
15	Thiercelin et al. (52)	Patient underwent GIT surgery		IV	52.9 ± 3.7	6.03 ± 0.44	83.1 ± 4.6^*f*^	103 ± 5.5	N/M	N/M	N/M
				PO	35.6 ± 1.9	6.08 ± 0.34	124.7 ± 6.7^*f*^	68.5 ± 3.66	N/M	N/M	N/M
16	Daneshmend et al. (45)	Patients with cirrhosis		PO	0.74 ± 0.04^*a*^	10.8 ± 2.4	157.8 ± 36.6	1.15 ± 0.26^*b*^	N/M	N/M	N/M
		Patients with schistosomiasis			0.79 ± 0.05^*a*^	8.4 ± 1.3	128.7 ± 18.0	1.21 ± 0.15^*b*^	N/M	N/M	N/M
17	Shaffer et al. (10)	Patients with Crohn’s		PO	0.57 ± 0.1^*a*^	8.5 ± 0.7	124 ± 16	53.3 ± 3.3	N/M	N/M	N/M
		Patients with colitis		PO	0.58 ± 0.1^*a*^	6.8 ± 0.5	95 ± 11	68.33 ± 8.3	N/M	N/M	N/M
		Patients with Crohn’s		IV	N/M	N/M	123 ± 7	N/M	N/M	N/M	N/M
		Patients with colitis		IV	N/M	N/M	103 ± 13	N/M	N/M	N/M	N/M
18	Ashiq et al. (51)	Patients with amoebiasis		PO	0.801 ± 0.02^*a*^	7.451 ± 0.32	87.37 ± 2.56	1.23 ± 0.05^*b*^	N/M	7.03 ± 0.2	1.8 ± 0.07
19	Eradiri et al. (57)	Crohn’s disease		PO	0.732 ± 0.09^*a*^	9.5 ± 2.1	70.55 ± 22.11	0.921 ± 0.17^*b*^	0.1 ± 0.02^*b*^	N/M	N/M
20	Bergan et al. (30)	Patients with Ileostomy		PO	33.12 ± 2.60	11.9 ± 0.6	199.20 ± 28.7	32.33 ± 4.1	N/M	N/M	N/M
		Patients with other enteric diseases			37.50 ± 7.84	7.2 ± 2.2	130.81 ± 42.1	61.33 ± 17.5	N/M	N/M	N/M
		Coeliac			35.57 ± 4.80	7.4 ± 1.8	151 ± 39	57.8 ± 12.33	N/M	N/M	N/M
		Ulcerative colitis			40.71 ± 5.22	8.2 ± 2.6	147.5 ± 56.4	61.5 ± 16.1	N/M	N/M	N/M
		Crohn’s			57.73 ± 53.52	5.8 ± 2.0	158 ± 58	59.5 ± 25.6	N/M	N/M	N/M
		Jejunoileal shunt			42.01 ± 11.76	7.5 ± 1.8	140 ± 29	67.83 ± 24.33	N/M	N/M	N/M
21	Kurji et al. (66)	Diabetics		PO	N/M	14.42 ± 3.03	71.77 ± 9.26	N/M	N/M	5.63 ± 0.4	1.7 ± 0.01
22	Mattie et al. (80)	Patients with mixed infection		PO	58.7 ± 30.9	8.34	N/M	81.3 ± 28.4	N/M	N/M	N/M
23	Fonnes et al. (1)	Patients with appendectomy		IP	N/M	5.4-9.5	109-252	N/M	N/M	9.9-16.9	1.0-4.2

^*a*^ L/kg.

^*b*^ mL/min/kg.

^*c*^ mL/min × 1.73 m^2^.

^*d*^ AUC_0–24._

^*e*^ AUC_0–10_.

^*f*^ AUC_ss_.

^*g*^ CAPD: continuous ambulatory peritoneal dialysis, HD: hemodialysis, HM: hydroxy metabolite, IP: intraperitoneal, ND: not detected, RF: renal failure, ROA: route of administration.

### Oral administration of MTZ

#### Hepatic impairment

Only one study that has reported the oral administration of MTZ to patients with hepatic impairment, demonstrated AUC_0–∞_ in patients with cirrhosis and schistosomiasis as 157.8 ± 36 and 128.7 ± 18.0 µg·h/mL, respectively (45).

#### Enteric disease

One of the clinical studies has reported Cl_R_ as 0.1 ± 0.02 mL/min/kg in patients with Crohn’s disease after administering a 250 mg oral dose of MTZ (57). Another study has depicted a 1.3-fold decrease in AUC_0–∞_ in patients with ulcerative colitis compared to Crohn’s patients (10). Moreover, a clinical study has presented Cl_T_ of 32.33 ± 4.1 mL/min in patients after ileostomy (30).

#### Type II diabetes

One study has investigated the effect of diabetes on the PK of MTZ, which reported an increase in *C*_max_ and *T*_max_ by 25.73% and 20.69% in diabetic patients in comparison to the non-diabetic population (66).

#### Infection

A clinical study has reported AUC_0–∞_ 87.37 ± 2.56 µg·h/mL among patients with amoebiasis following oral administration of MTZ (51). Another study has demonstrated Cl_T_ of 81.3 ± 28.4 mL/min when a dose of 500 mg TID was delivered to patients with mixed aerobic and anaerobic infection (80). The detailed information is provided in Table 3.

### Other routes of administration of MTZ

One of the research studies has discussed intraperitoneal administration of MTZ in patients with appendicitis, where the *C*_max_ was reported as 9.9–16.9 µg/mL (15). All PK parameters are summarized in Table 3.

### PK of MTZ in special population

#### IV administration of MTZ

##### Pregnant women

A study has revealed that AUC_0–48_ was 108 ± 33 µg·h/mL after administering a 500 mg IV dose of MTZ to pregnant women (42).

##### Infants

Among 67 studies, only 1 investigated PK parameters in infants, which demonstrated an age-proportional increase in Cl_T_ (67). The additional parameters are shown in Table 4.

**TABLE 4:** Pharmacokinetic parameters of metronidazole in special population^*f*^

Sr. no.	Authors	Population		ROA	*V*_d_ (L)	*t*_1/2_ (h)	AUC_0–∞_ (µg·h/mL)	Cl_T_ (mL/min)	*C*_max_ (µg/mL)	*T*_max_ (h)
1	Visser et al. (42)	Pregnant women		IV	N/M	6.9 ± 2.68	108 ± 33^*c*^	1.19 ± 0.33^*b*^	N/M	N/M
2	Jager-Roman et al. (67)	Infants	28–30^*e*^	IV	0.65 ± 0.06^*a*^	75.3 ± 16.9	N/M	0.12 ± 0.05^*b*^	N/M	N/M
			32–35^*e*^		0.71 ± 0.03^*a*^	35.4 ± 1.5	N/M	0.44 ± 0.06^*b*^	N/M	N/M
			36–40^*e*^		0.69 ± 0.06^*a*^	24.8 ± 1.6	N/M	0.99 ± 0.07^*b*^	N/M	N/M
3	Passmore et al. (9)	Nursing mothers and infants		PO	N/M	N/M	N/M	N/M	*C*_M_: 12.9 ± 0.6 *C*p_I_: 1.6 ± 0.1	N/M
4	Heistrberg et al. (44)	Nursing mothers and infants		PO	N/M	N/M	N/M	N/M	C_M_: 11.6–18 Cp_I_: 0.6–4.9	N/M
5	Amon et al. (27)	Infants		PO	0.87^*a*^	10.66	374.9	0.94^*b*^	N/M	N/M
		Children			0.64 ± 0.18^*a*^	8.98 ± 3.56	175.0 ± 57.1	0.94 ± 0.37^*b*^	N/M	N/M
6	Amon et al. (58)	Pregnant women	1,000	PO	0.752 ± 0.09^*a*^	7.73 ± 1.11	233.35 ± 18.2	N/M	N/M	N/M
		Non-pregnant women	1,000		0.692 ± 0.153	7.80 ± 1.30	281.94 ± 75.39	N/M	N/M	N/M
		Pregnant women	250		0.59 ± 0.19	5.70 ± 3.46	40.09 ± 16.366	N/M	N/M	N/M
		Non-pregnant women	250		0.62 ± 0.17	5.92 ± 30	50.2 ± 3.9	N/M	N/M	N/M
7	Wang et al. (75)	Pregnant women	ET	PO	0.517 ± 0.55^*a*^	N/M	117.4 ± 121.8^*d*^	1.04 ± 1.13^*b*^	17.0 ± 19.7	1.5
			MD		0.793 ± 0.29^*a*^	N/M	69.2 ± 19.9^*d*^	1.8 ± 0.89^*b*^	11.7 ± 3.1	1.3 ± 0.4
			LT		0.717 ± 0.33^*a*^	N/M	79.2 ± 14.7^*d*^	1.37 ± 0.45^*b*^	14.2 ± 4.2	1.3 ± 0.8
8	Lares-Asseff et al. (61)	Malnourished children		PO	1.500 ± 0.87^*a*^	11.73 ± 6.1	191.65 ± 101.7	1.56 ± 0.9^*b*^	10.48 ± 5.2	3.4 ± 1.9
		Nutritionally rehabilitated children			1.598 ± 0.97^*a*^	5.68 ± 1.97	140.04 ± 46.9	3.11 ± 0.1^*b*^	10.49 ± 3.4	4.48 ± 1.7

^*a*^ L/kg.

^*b*^ mL/min/kg.

^*c*^ AUC_0–48_.

^*d*^ AUC_0–12._

^*e*^ Gestational age in weeks.

^*f*^ Cp_I_: infant plasma concentration, Cp_M_: mother plasma concentration, ET: early term, LT: late-term, MT: middle term, ROA: route of administration.

### Oral administration of MTZ

#### Pregnant women

Of a total 67 studies, 2 were conducted in pregnant women. One study has reported a lowered AUC_0–∞_ in pregnant women compared to non-pregnant women, that is, 233.35 ± 18.21 versus 281.94 ± 75.39 µg·h/mL (58). In another study, the *C*_max_ of MTZ was reported to be decreased by 1.5- and 1.2-fold during the middle and later terms of pregnancy in contrast to the early term (75).

#### Infants

A study has depicted higher AUC_0–∞_ in infants than children, that is, 374.9 and 175 µg·h/mL, respectively (27). Another study comparing PK of MTZ in malnourished and nutritionally rehabilitated children has revealed that the former had lower Cl_T_, that is, 1.56 ± 0.90 versus 3.11 ± 0.1 mL/min/kg (61).

#### Nursing mothers and infants

Two studies have found that when oral MTZ was administered to nursing mothers, its traces were observed in infants’ plasma, that is, 1.69 ± 0.17 and 0.6-4.9 µg/mL, respectively (9, 44), as demonstrated in Table 4.

### PK of MTZ in drug-drug interaction and drug-food interaction

#### Drug-drug interaction

A study has reported that silymarin decreased MTZ AUC_0–48_ from 230.58 ± 84.57 to 166.22 ± 63.84 µg·h/mL (73). The diosmin on co-administration with MTZ caused an increase in its *C*_max_, that is, 17.5 ± 4.1 versus 21.2 ± 3.5 µg/mL (72). A clinical study has reported an increase in Cl_T_ of MTZ when co-administered with omeprazole, that is, 75.5 ± 17.83 mL/min versus 82.83 ± 23.8 mL/min at an oral dose of 500 mg (78). The other PK parameters are outlined in Table 5.

**TABLE 5:** Pharmacokinetic parameters of metronidazole in drug-drug and drug-food interaction^*g*^^,*h*^

Sr. no.	Authors	Drugs	*V*_d_ (L)	*t*_1/2_ (h)	AUC_0–∞_ (µg·h/mL)	Cl_T_ (mL/min)	Cl_R_ (mL/min)	*C*_max_ (µg/mL)	*T*_max_ (h)
Pharmacokinetic parameters of metronidazole in drug-drug interaction									
1	Sakurai et al. (26)	MTZ	52.95 ± 6.1560	11.03 ± 2.93	75.53 ± 18.822^*d*^	58.7 ± 16.02	N/M	8.987 ± 1.809	2 ± 4.0
		MTZ + AMOX + VPZ	51.71 ± 5.0214	10.76 ± 2.80	75.520 ± 19580^*d*^	59.01 ± 16.78	N/M	8.874 ± 1.806	3 ± 6.0
2	Dilger et al. (49)	BDS	43.8 ± 15.2	2.1–5.1	0.0048 ± 0.002	0.17 ± 0.08^*b*^	N/M	0.001 ± 0.0003	4.1–6.2
		BDS + MTZ	60.6 ± 38.1	2.6–6.0	0.00477 ± 0.002	0.18 ± 0.09^*b*^	N/M	0.00104 ± 0.0004	3.9–4.8
3	Kim and Park (59)	FEXO	N/M	4.7 ± 1.0	20.757 ± 55.71	N/M	N/M	0.3044 ± 0.1396	2.2 ± 1.1
		FEXO + MTZ	N/M	5.4 ± 2.8	19.99 ± 39.73	N/M	N/M	0.293 ± 0.137	2.4 ± 1.1
4	Loft et al. (64)	MTZ	51 ± 16	7.6 ± 1.3	N/M	77 ± 20	6.4 ± 2.6	N/M	N/M
		Cimetidine + MTZ	51 ± 12	7.9 ± 0.8	N/M	75 ± 21	6.6 ± 1.4	N/M	N/M
5	Das et al. (31)	MTZ	N/M	8.4 ± 1.5	115.0 ± 19.4	N/M	N/M	21.0 ± 19.7	1–1.50
		MTZ + CAZ + AVI	N/M	9.0 ± 1.5	121.0 ± 19.0	N/M	N/M	21.4 ± 17.4	1–2.50
6	Obodozie et al. (32)	NIPRD-AM1 + MTZ	N/M	N/M	73.52	N/M	N/M	7.83	1
7	Rajnarayana et al. (72)	MTZ	N/M	5.7 ± 4.1	249 ± 80	N/M	N/M	17.5 ± 4.1	1.2 ± 0.4
		Diosmin + MTZ	N/M	7.6 ± 2.2	315 ± 88	N/M	N/M	21.2 ± 3.5	1 ± 0.3
8	Rajnarayana et al. (73)	MTZ	0.442 ± 0.215^*a*^	10.61 ± 4.32	230.58 ± 84.57^*c*^	0.51 ± 0.16^*b*^	N/M	16.91 ± 4.24	1.12 ± 0.56
		SIL + MTZ	0.454 ± 0.243^*a*^	8.08 ± 3.96	166.22 ± 63.84^*c*^	0.728 ± 0.29	N/M	12.00 ± 2.92	1.5 ± 0.97
9	Wang et al. (76)	MDZ	N/M	2 ± 0.3	0.19 ± 0.0643	N/M	N/M	0.0586 ± 0.030	1
		MDZ + MTZ	N/M	2.2 ± 0.9	0.177 ± 60.9	N/M	N/M	0.0551 ± 0.0208	1.25
10	Pierce et al. (77)	MTZ	N/M	N/M	30.60 ± 76.627^*e*^	N/M	N/M	28.193 ± 6.249	0.50-3.00
		MTZ + MMX	N/M	N/M	30.43 ± 74.308^*e*^	N/M	N/M	28.057 ± 5.522	0.5-3.00
11	Goddard et al. (69)	MTZ	N/M	N/M	82.2–118.4	58.33–80	N/M	7.94–10.76	N/M
		MTZ + OMEP	N/M	N/M	69.9–123.0	55–96.6	N/M	7.73–10.52	N/M
12	Calafatti et al. (70)	MTZ	N/M	N/M	14.16–20.22^*f*^	N/M	N/M	10.99–16.05	1.08–1.77
		MTZ + OMEP	N/M	N/M	13.54–21.12^*f*^	N/M	N/M	8.29–49.81	0.78–1.5
13	Jessa et al. (78)	MTZ	47.9 ± 10.2	7.49 ± 1.53	62.5156 ± 15.2087	75.5 ± 17.83	N/M	N/M	N/M
		MTZ + OMEP	56.1 ± 18.8	7.91 ± 1.78	57.2541 ± 12.8025	82.83 ± 23.8	N/M	N/M	N/M
14	David et al. (68)	MTZ	N/M	8.7–11	103.4–130.8	N/M	N/M	8.3–10.6	0.5–3
		MTZ + OMEP	N/M	8.2–10	97–124.4	N/M	N/M	8.3–9.5	0.5–6
Pharmacokinetic parameters of metronidazole in drug-food interaction									
15	Hamberg et al. (82)	EDD	6.8	N/M	4310	N/M	11	N/M	3.56
		PDD	7.9	N/M	4820	N/M	7.8	N/M	3.41
16	Wu et al. (84)	Fasting	44.88 ± 5.79	N/M	101.2 ± 19.2	63.83 ± 12.55	N/M	7.562 ± 0.790	1.58 ± 0.71
		Non-fasting	46.62 ± 4.67	N/M	31.88 ± 4.28	66.43 ± 8.83	N/M	2.257 ± 0.381	2.46 ± 1.50
17	Melande et al. (74)	Fasting	N/M	3.9–19.2	N/M	N/M	N/M	6.68–12.5	0.5–2
		Non-fasting	N/M	7.05–19.38	N/M	N/M	N/M	6.32–10.7	0.53–4

^*a*^ L/kg.

^*b*^ mL/min/kg.

^*c*^ AUC_0–48._

^*d*^ AUC_0–12._

^*e*^ AUC_0–24_.

^*f*^ AUC_0–2._

^*g*^ mg.

^*h*^ ALP: Alprazolam, AMOX: amoxicillin, AVI: avibactam, BDS: budesonide, CAZ: ceftazidime, EDD: energy deficient diet, FEXO: fexofenadine, LRP: Lorazepam, LVX: levofloxacin, MDZ: midazolam, MMX: mesalamine, MTZ: metronidazole, OMEP: omeprazole, PDD: protein deficient diet, PHT: Phenytoin, SIL: silymarin, VPZ: vonoprazan.

#### Drug-food interaction

In an investigation, the AUC_0–∞_ was reported to be reduced in the fed state compared to the fasted state, that is, 31.88 ± 4.28 versus 101.2 ± 19.2 µg·h/mL (84). Similarly, an extended *t*_1/2_ was observed when the drug was given on an empty stomach, that is, 7.05–19.38 h (74). The remaining values are mentioned in Table 5.

### Meta-analysis

**Fig 3:**
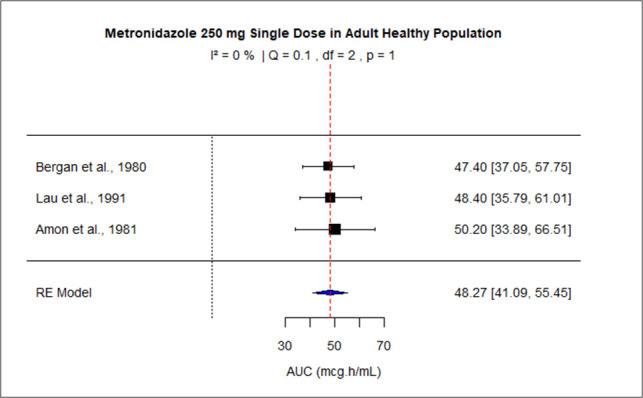
Forest plot of AUC for MTZ 250 mg single dose. The figure displays the mean of AUC and sample size (black square and its size) and 95% CI (the horizontal lines) for adult healthy population across studies, with the blue diamond at the bottom showing the overall pooled AUC estimate and its CI. The vertical red dashed line is a reference for the overall pooled AUC estimate, allowing for a visual comparison across studies. In this figure, the pooled AUC for the 250 mg single dose is 48.27 (95% CI: 41.09–55.45), and *I*^2^ of 0% indicates low heterogeneity across the studies.

**Fig 4:**
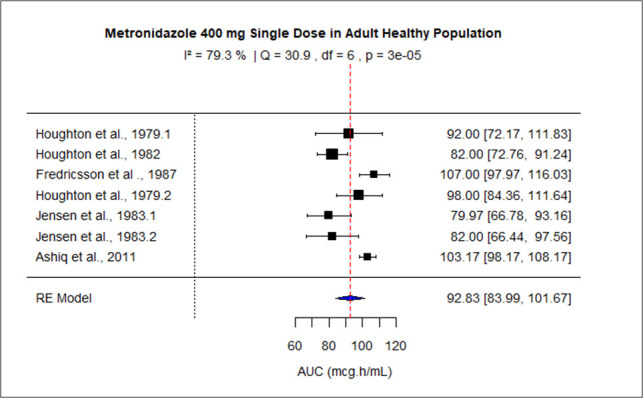
Forest plot of AUC for MTZ 400 mg single dose. The figure displays the mean of AUC and sample size (black square and its size) and 95% CI (the horizontal lines) for adult healthy population across studies, with the blue diamond at the bottom showing the overall pooled AUC estimate and its CI. The vertical red dashed line is a reference for the overall pooled AUC estimate, allowing for a visual comparison across studies. In this figure, the pooled AUC for the 400 mg single dose is 92.83 (95% CI: 83.99–101.67), and *I*^2^ of 79.3% indicates moderate heterogeneity across the studies.

**Fig 5:**
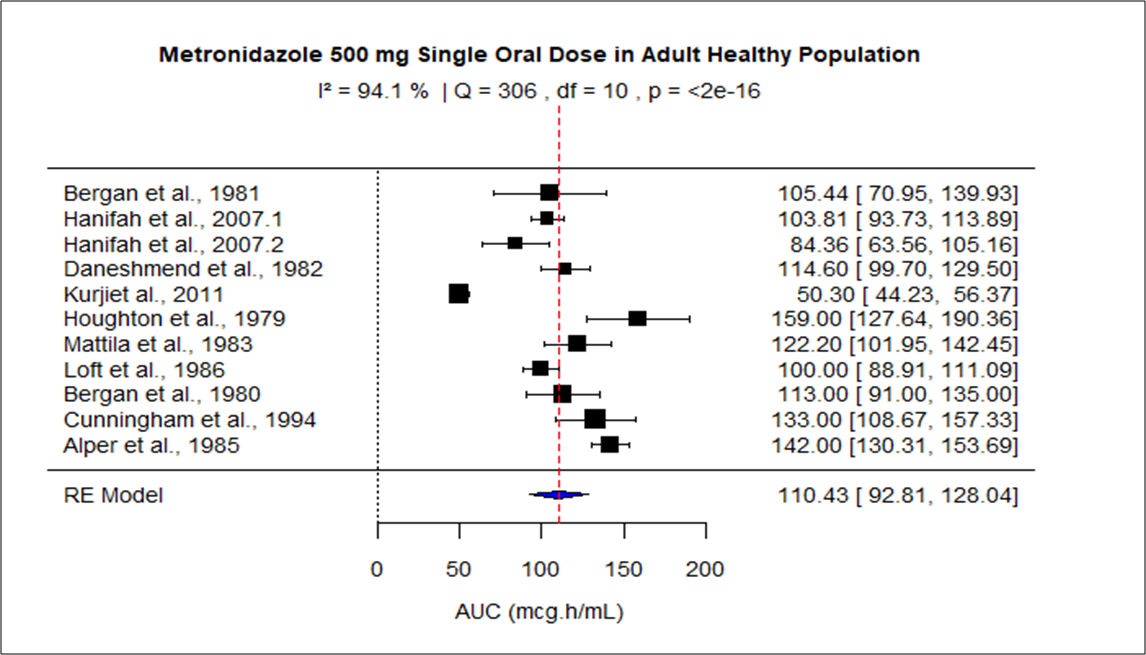
Forest plot of AUC for MTZ 500 mg single oral dose. The figure displays the mean of AUC and sample size (black square and its size) and 95% CI (the horizontal lines) for adult healthy population across studies, with the blue diamond at the bottom showing the overall pooled AUC estimate and its CI. The vertical red dashed line is a reference for the overall pooled AUC estimate, allowing for a visual comparison across studies. In this figure, the pooled AUC for the 500 mg single dose is 110.43 (95% CI: 92.81–128.04), and *I*^2^ of 94.1% indicates high heterogeneity across the studies.

**Fig 6:**
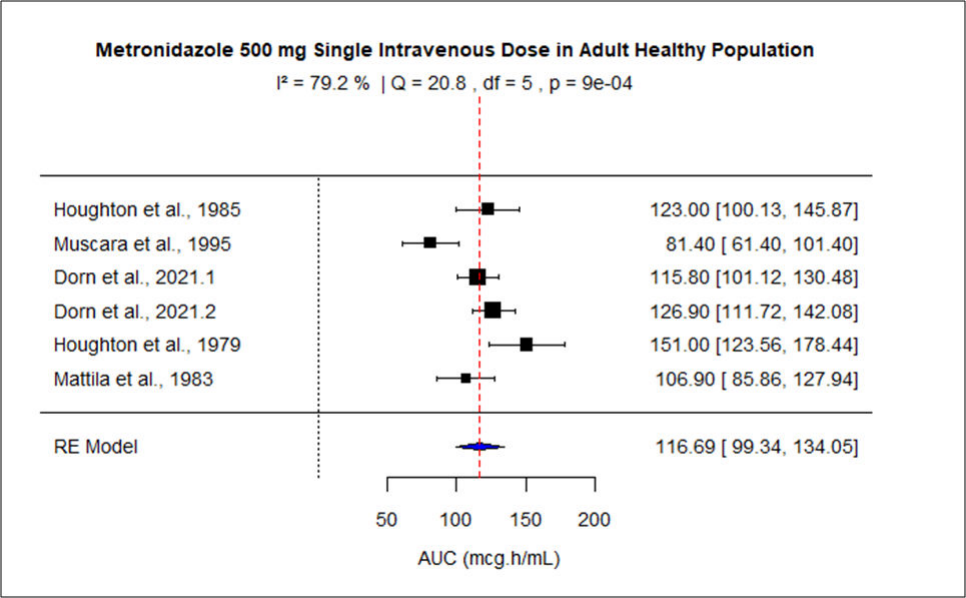
Forest plot of AUC for MTZ 500 mg single IV dose. The figure displays the mean of AUC and sample size (black square and its size) and 95% CI (the horizontal lines) for adult healthy population across studies, with the blue diamond at the bottom showing the overall pooled AUC estimate and its CI. The vertical red dashed line is a reference for the overall pooled AUC estimate, allowing for a visual comparison across studies. In this figure, the pooled AUC for the 500 mg single dose is 116.69 (95% CI: 99.34–134.05), and *I*^2^ of 79.2% indicates moderate heterogeneity across the studies.

**Fig 7:**
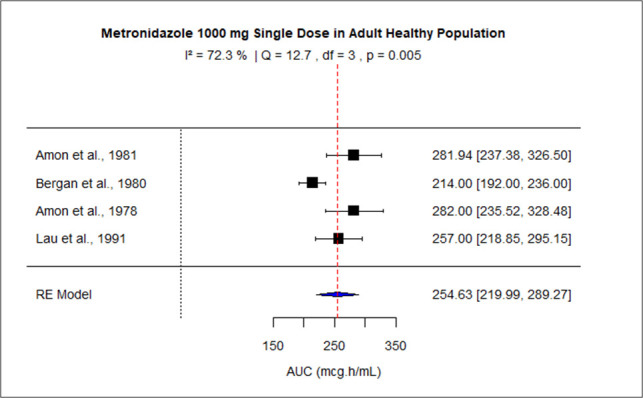
Forest plot of AUC for MTZ 1000 mg single dose. The figure displays the mean of AUC and sample size (black square and its size) and 95% CI (the horizontal lines) for adult healthy population across studies, with the blue diamond at the bottom showing the overall pooled AUC estimate and its CI. The vertical red dashed line is a reference for the overall pooled AUC estimate, allowing for a visual comparison across studies. In this figure, the pooled AUC for the 1,000 mg single dose is 254.63 (95% CI: 220–289.3), and *I*^2^ of 72.3% indicates moderate heterogeneity across the studies.

**Fig 8:**
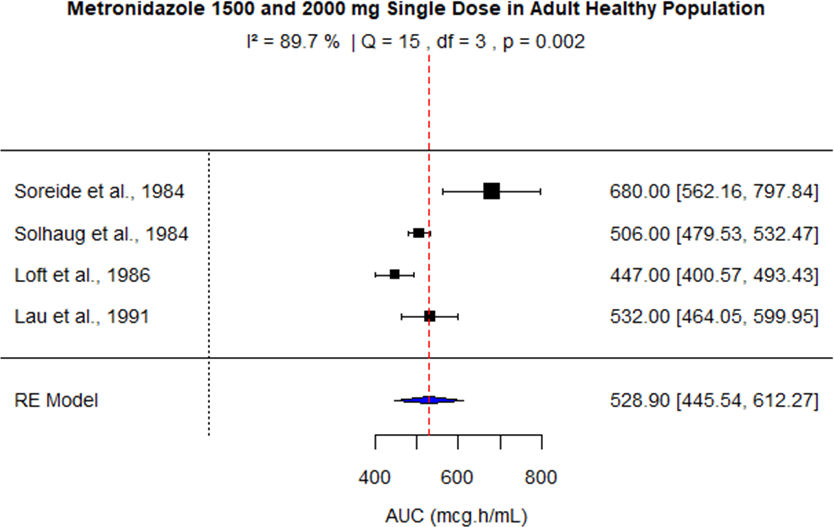
Forest plot of AUC for MTZ 1,500 mg (Soreide and Solhaug) and 2,000 mg (Loft and Lau) single dose. The figure displays the mean of AUC and sample size (black square and its size) and 95% CI (the horizontal lines) for adult healthy population across studies, with the blue diamond at the bottom showing the overall pooled AUC estimate and its CI. The vertical red dashed line is a reference for the overall pooled AUC estimate, allowing for a visual comparison across studies. In this figure, the pooled AUC is 528.9 (95% CI: 445.54–612.27), and *I*^2^ of 89.7% indicates moderate heterogeneity across the studies.

The pooled AUC for doses 250 mg, 400 mg, 500 mg (PO), 500 mg (IV), 1,000 mg, and 1,500–2,000 mg was 48.27 (95% confidence interval [CI]: 41.09–55.45), 92.83 (95% CI: 83.99–101.67), 110.43 (95% CI: 92.81–128.04), 116.69 (95% CI: 99.34–134.05), 245.63 (95% CI: 219.99–289.27), and 528.90 (95% CI: 445.54–612.27), respectively (Fig. 3 to 8). The 0% *I*^2^ of 0% at a dose of 250 mg indicated lower heterogeneity across the studies. Similarly, higher *I*^2^ values after 400 mg, 500 mg (IV), 1,000 mg, and 1,500–2,000 mg doses exhibited significant heterogeneity.

**Fig 9:**
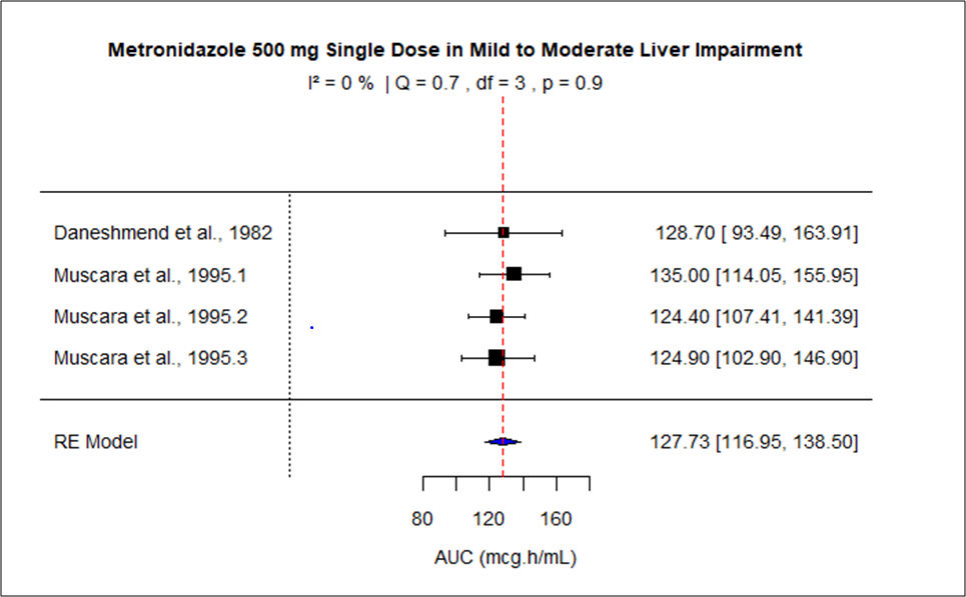
Forest plot of AUC for MTZ 500 mg single dose in Schistosomiasis-induced liver disease (45, 50) and class A and B Child-Pugh liver cirrhosis (50). The figure displays the mean of AUC and sample size (black square and its size) and 95% CI (the horizontal lines) for adult healthy population across studies, with the blue diamond at the bottom showing the overall pooled AUC estimate and its CI. The vertical red dashed line is a reference for the overall pooled AUC estimate, allowing for a visual comparison across studies. In this population, the pooled AUC is 127.73 (95% CI: 116.95–138.5), and *I*^2^ of 0% indicates low heterogeneity across the studies.

**Fig 10:**
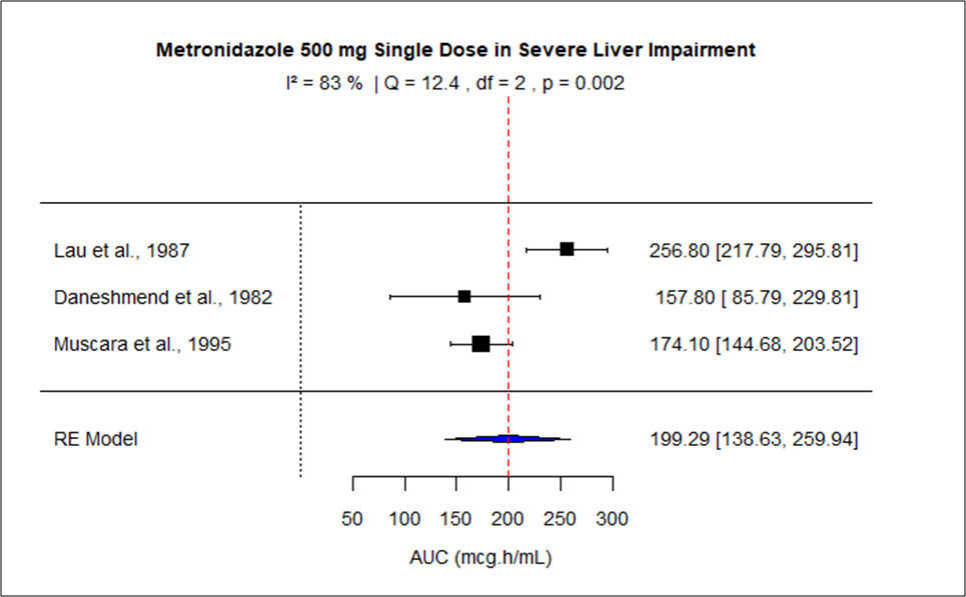
Forest plot of AUC for MTZ 500 mg single dose in severe liver impairment. The figure displays the mean of AUC and sample size (black square and its size) and 95% CI (the horizontal lines) for adult healthy population across studies, with the blue diamond at the bottom showing the overall pooled AUC estimate and its CI. The vertical red dashed line is a reference for the overall pooled AUC estimate, allowing for a visual comparison across studies. In this population, the pooled AUC is 199.29 (95% CI: 138.63–259.94), and *I*^2^ of 83% indicates moderate heterogeneity across the studies.

**Fig 11:**
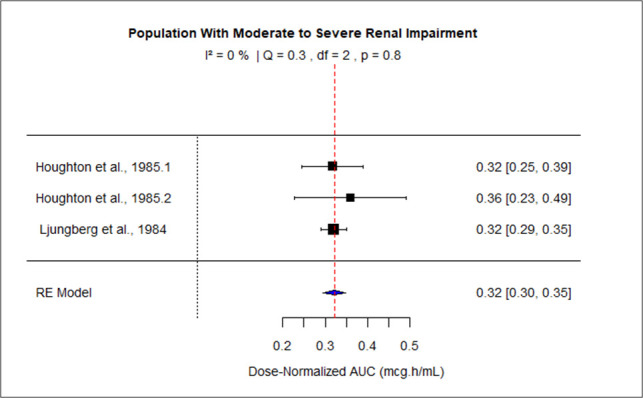
Forest plot of dose-normalized AUC for MTZ in patients with moderate to severe renal impairment.

**Fig 12:**
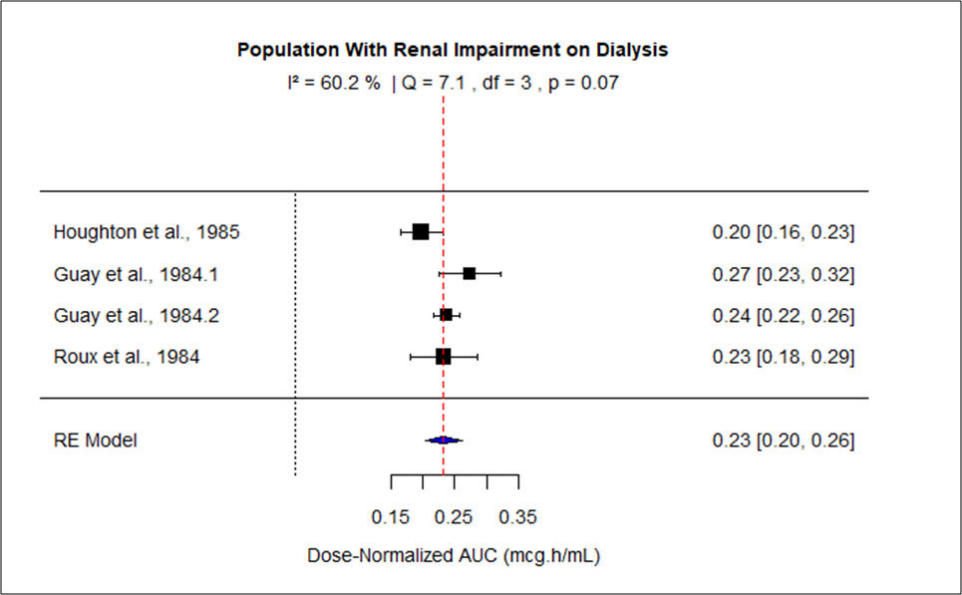
Forest plot of dose-normalized AUC for MTZ in patients with renal impairment on dialysis.

**Fig 13:**
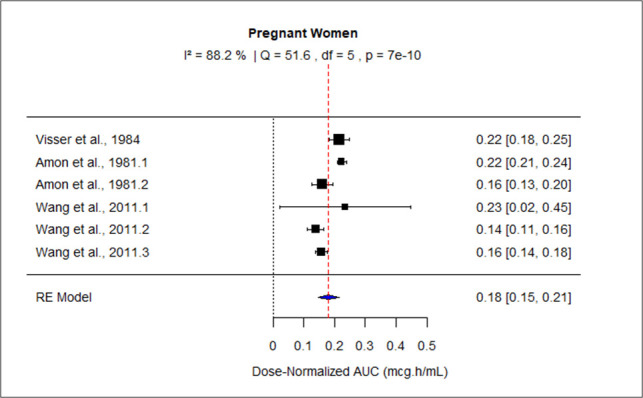
Forest plot of dose-normalized AUC for MTZ in pregnant women.

The pooled effect size of AUC for patients with mild-moderate and severe liver impairment was 127.73 (95% CI: 116.95–138.50), and 199.29 (95% CI: 138.63–2.59.94) correspondingly. The pooled dose-normalized AUC for individuals with moderate to severe renal impairment, patients on dialysis, and pregnant women was 0.32 (95% CI: 0.30–0.35), 0.23 (95% CI: 0.20–0.26), and 0.18 (95% CI: 0.15–0.21), respectively. Low heterogeneity was observed among studies conducted in patients with mild to moderate liver disease and moderate to severe renal impairment as the value of *I*^2^ was 0%. The higher values of *I*^2^ indicated moderate heterogeneity across the studies performed in renal patients on dialysis, pregnant women, and patients with severe liver disease, that is, 60.2%, 88.2%, and 83%, respectively. Figures 9 to 13 present the forest plots of these results.

**Fig 14:**
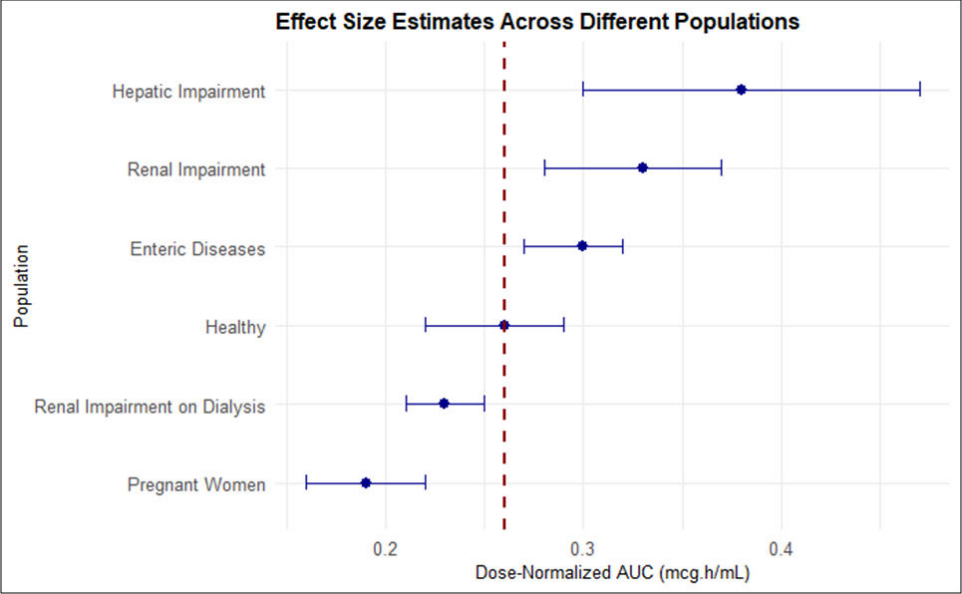
Forest plot of dose-normalized AUC values for each population subgroup. The figure displays dose-normalized AUC values (and 95% confidence intervals [CIs]) for individual population groups: Healthy, Enteric Diseases, Hepatic Impairment, Pregnant Women, Renal Impairment, and Renal Impairment on Dialysis. Each effect size is represented by a circle. The horizontal lines indicate the 95% CIs for each population’s AUC estimate. The vertical dashed line represents the overall pooled AUC estimate of the healthy population, allowing for a visual comparison across populations. Non-overlapping or minimally overlapping CIs suggest meaningful differences between populations. This visual comparison allows for an intuitive assessment of variability across groups.

To illustrate differences in effect sizes across populations, we present the pooled estimates alongside their 95% CIs in Fig. 14. Visual inspection of the CIs enables identification of differences between groups, as non-overlapping or minimally overlapping intervals are generally indicative of statistically meaningful differences (103). This method provides a straightforward and transparent way to assess variation across populations without formal hypothesis testing.

## DISCUSSION

The main objective of this review was to systematically compile, summarize, and evaluate all available data on the PK of MTZ. Out of 67 studies, 36 were conducted in healthy individuals, 23 in diseased persons, and 8 in special populations. Among those 36 studies performed on healthy subjects, 5 evaluated the effect of age, regimen, smoking, gender, and obesity on the PK of MTZ, while 14 depicted drug-drug interaction and 3 discussed the drug-food interactions.

The age-related decline in renal function is the most likely reason for lower Cl_R_ seen in elders using MTZ; nevertheless, since the overall elimination of MTZ is conserved in old age, no dosage adjustment is required (47). MTZ exhibits approximately the same *C*_max_ in saliva as in plasma due to its higher diffusion from blood to saliva. It is a low molecular weight drug; therefore, it can easily permeate all tissues and body fluids (6). Mustofa et al. recommended using saliva instead of plasma to measure the PK parameters of MTZ (104). The elevated *C*_max_ of MTZ in the male population is because of its more efficient absorption in men than in women. The altered PK parameters necessitate dose monitoring in males (60). The interaction between tobacco and MTZ is influenced by the number of cigarettes smoked daily. Smoking 15 or more cigarettes per day reduces the *C*_max_ of MTZ in the body. Polycyclic aromatic hydrocarbon, a chemical found in cigarette smoke, causes the induction of CYP1A1, CYP1A2, and CYP2E1 enzymes that accelerate the hepatic biotransformation of MTZ. Therefore, a dose adjustment in chain smokers is necessary to achieve optimum therapeutic effects (56). Obese persons have greater *V*_d_ and lower *C*_max_ due to the high-fat content in their bodies; therefore, a high dose is recommended for these patients to get the therapeutic concentration (28). Multiple-dosage regimens of MTZ exhibit higher *C*_max_ compared to a single-dose administration because of drug accumulation in the body to reach the study state concentration. The variations are negligible in healthy individuals, but patients with hepatic or renal impairment need dose adjustments to avoid drug toxicity (48). The physicochemical properties of MTZ, particularly poor lipid solubility, may contribute to its low vaginal absorption; yet, these dosage forms are good for local effects (85). The relative bioavailability of MTZ suppositories is 90% compared to the oral tablets due to partial bypass of the first-pass effect; thus, rectal administration might be used as an alternate treatment (15). Moreover, the proportional increase in *C*_max_ of MTZ with dose indicates its linear PK (83).

The Cl_R_ of MTZ is decreased in patients with renal impairment, but no significant accumulation occurs because only 10% of the dose is excreted through Cl_R_ in its parent form and the rest of the drug is metabolized by the liver, making hepatic clearance a major route of elimination (19, 43). However, severe renal dysfunction modifies the PK parameters of MTZ metabolites (hydroxy and acidic metabolites). Since these metabolites are biologically active, they can cause toxicity; thus, close monitoring of renal function is required to avoid potential side effects of the drug (7). Hemodialysis may also change the PK of MTZ as it significantly increases Cl_T_; hence, appropriate time and dose adjustments are necessary for these patients (65). Hepatic clearance accounts for >50% of Cl_T_ of MTZ; that is why liver insufficiency prolongs its *t*_1/2_ and decreases Cl_T_, which may be due to the poor metabolism of MTZ caused by impaired cytochrome P450 system. Consequently, dose adjustment is required to avoid drug toxicity (20, 45, 50, 54, 62, 63). Diabetes-related physiological changes, such as elevated fat content and impaired renal, hepatic, and GIT functioning, are the source of increased *C*_max_ and *T*_max_ of MTZ in the diabetic population. About 80% of diabetic patients develop hepatic glycogen lesion that results in hepatomegaly, which interferes with the metabolism of MTZ (66). Patients with different anaerobic and protozoa infections do not exhibit a substantial change in PK of MTZ, indicating no need for dosage adjustment (46, 51, 55, 80). Oral administration of MTZ in enteric diseases may affect its PK parameters due to differences in GIT absorption. In contrast, IV therapy does not show any change in PK of MTZ (10, 30, 52, 57, 81).

Due to an increase in mean plasma volume and a decrease in plasma protein concentrations, the PK parameters of MTZ differ during pregnancy (42, 58, 75). A considerable amount of MTZ may enter newborns via breast milk; so, it would be important to prescribe it with caution to nursing mothers (9, 44). Infants show higher AUC_0–∞_ compared to adults due to their developing bodies and immature GIT, liver, and kidney functions (27, 67). The prolonged *t*_1/2_ and lower Cl_T_ of MTZ in severely malnourished children warrant dose adjustment in these patients (61).

Drug-drug interactions make treatment goals more difficult to achieve. Cytochrome 450 enzymes, including CYP3As, CYP2E1, and CYP2A6, are involved in the metabolism of MTZ. The PK parameters of MTZ may be affected by the concurrent use of medications that affect these enzymes; it necessitates cautious co-administration of such drugs with MTZ. Diosmin inhibits CYP2A6 and CYP3A4 enzymes, resulting in a decreased Cl_T_ and increased *C*_max_ of MTZ (72). Silymarin increases CL_T_ of MTZ via inducing P-glycoprotein, which on average, results in a 29.3% drop in *C*_max_of the drug (73). Food delays the absorption of MTZ but has little effect on its PK parameters, so no dose adjustment is required (74, 84).

The meta-analysis was conducted to robustly assess the variability of a PK parameter, AUC, across multiple studies. It allows us to estimate the pooled effect size more precisely. This meta-analysis indirectly evaluates AUC-dose proportionality by examining how the AUC scales with increased doses of MTZ. Moreover, it also compares the pooled effect size of AUC among healthy, diseased, and special populations. It shows that MTZ behaves consistently across various dosing regimens and in different populations, which is necessary for the safe and effective use of the drug. It helps clinicians with the individualization of therapy. Our meta-analysis revealed altered MTZ exposure in several special populations compared to healthy individuals. Specifically, lower pooled AUC values were observed in pregnant women and patients undergoing dialysis, while higher pooled AUCs were found in individuals with renal impairment, hepatic insufficiency, and enteric diseases. These findings suggest potential implications for dose adjustment in clinical practice. However, current regulatory sources do not recommend dosing modifications for MTZ during pregnancy. The FDA label for MTZ does not provide specific pharmacokinetic data or dosing guidance for pregnant women (105), and the scientific clinical database (Lexicomp) reports that MTZ PK in pregnancy are similar to those in non-pregnant individuals (1^106^). Therefore, while our analysis identified lower pooled AUCs in pregnancy, this difference may not be clinically significant and does not, at present, justify empirical dose changes without further clinical evidence.

In renal impairment, the FDA reports that decreased renal function does not significantly alter the PK of MTZ itself; however, substantial increases are observed in the systemic exposure of its metabolites, specifically, a twofold increase in hydroxy-MTZ and a fivefold increase in MTZ acetate in end-stage renal disease patients. As a result, dose adjustment is not explicitly recommended, but monitoring for adverse events is advised, especially in dialysis patients, to mitigate potential toxicity from metabolite accumulation (107). This supports our interpretation that while the parent drug AUC may be reduced, caution remains warranted.

In contrast, the FDA recommends dose modifications in severe hepatic impairment (Child-Pugh C), citing a 114% increase in AUC of MTZ and advising a 50% dose reduction for amebiasis and increased dosing intervals for trichomoniasis. For mild to moderate hepatic impairment, no dose adjustment is required, though monitoring is recommended (107). Our pooled results align with this pattern, as increased AUCs in hepatic impairment were consistent with a need for dose reductions in patients with significant liver dysfunction. Generally, our findings complement current global regulatory guidance by providing quantitative pooled evidence of altered exposure across populations. However, we emphasize that any dosing changes should be guided by clinical context, therapeutic indications, and safety considerations, consistent with individualized treatment principles recommended by the FDA and other regulatory authorities.

The strength of this review article is that it covers the maximum number of studies published on healthy, diseased, and special populations until July 2024. However, it is necessary to be aware that certain limitations must be considered. This review includes only English-language articles, which may result in excluding relevant studies published in other languages. Only four databases were accessed for data retrieval, which might lead to the omission of crucial data and jeopardize the accuracy of results. Moreover, 94 relevant studies were inaccessible, which may limit the robustness of the analysis. The majority of the included studies have a clear risk of bias, which could affect the validity of the results. Furthermore, most studies do not give complete information on all included PK parameters.

Because of inter-population variability in AUC, individualized dosage regimes for MTZ should be considered to optimize therapeutic outcomes. Therapeutic drug monitoring can be beneficial in a population at risk of altered PK of MTZ, that is, hepatic patients, to provide adequate drug exposure and minimize toxicity. Future research should develop and validate PBPK models to predict MTZ disposition across diverse physiological and pathological conditions. Moreover, investigating the effect of genetic polymorphism on MTZ metabolism can improve dosing strategies. The results of this review article and meta-analysis should be used to develop dosing guidelines for MTZ by collaborating with regulatory authorities.

## CONCLUSIONS

This systematic review covers the majority of studies on the PK of MTZ in humans from five included databases. The PK studies include data on healthy, diseased, and special populations to identify the factors influencing the PK of MTZ. This information can be used to develop PK models that may help clinicians adjust the dose regimens for different populations to achieve optimal therapeutic effects and avoid toxicity. The heterogeneity in multiple studies suggests the need for future research strategies to ensure consistency in study design, implementation, and result reporting.

## Data Availability

The data discussed in this article are obtained from publicly available sources cited in this review. All data summarized and analyzed during this study are included in the article or its supplementary information file.

## SUPPLEMENTAL MATERIAL

Table S1 aac.01904-24-s0001.docx Screening and exclusion of articles based on their titles, abstract, animal studies, reviews, and accessibility.

ASM does not own the copyrights to Supplemental Material that may be linked to, or accessed through, an article. The authors have granted ASM a non-exclusive, world-wide license to publish the Supplemental Material files. Please contact the corresponding author directly for reuse.
