# Supplementary material for: Clinical pharmacokinetics of metronidazole: a systematic review and meta-analysis
Source: Antimicrob Agents Chemother. 2025 Jul 31;69(9):e01904-24. doi: 10.1128/aac.01904-24 (PMC12406673; doi:10.1128/aac.01904-24)
Supplement: Table S1 — Screening and exclusion of articles based on their titles, abstract, animal studies, reviews, and accessibility. [file aac.01904-24-s0001.docx]

**Clinical Pharmacokinetics of Metronidazole: A Systematic Review and Meta-Analysis**

**Running Head**: Metronidazole Pharmacokinetics: Review & Meta-Analysis

Iqra Shahzad^1†^, Mohammed S. Alasmari^2†^, Ammara Zamir^1^, Muhammad Fawad Rasool^1*^ Faleh Alqahtani^3,4*^,

^1^Department of Pharmacy Practice, Faculty of Pharmacy, Bahhaudin Zakariya University, 60800, Multan, Pakistan.

^2^Drug and poisoning information center, Security Forces Hospital, Riyadh, 11481, Saudi Arabia.

^3^Department of Pharmacology and Toxicology, College of Pharmacy, King Saud University, Riyadh 11451, Saudi Arabia

^4^King Salman Center for Disability Research, Riyadh, 11614, Saudi Arabia

^†^ These authors contributed equally to this work.

^*^Corresponding author: Muhammad Fawad Rasool [fawadrasool@bzu.edu.pk](mailto:fawadrasool@bzu.edu.pk), Faleh Alqahtani [afaleh@ksu.edu.sa](mailto:afaleh@ksu.edu.sa)

**Supplementary Table S1: Screening and Exclusion of Articles Based on Their Titles, Abstract, Animal studies, Reviews and Accessibility**

| **Auhors** | **Title** | **Journal** | **Volume(issue), page*#*** | **Doi** | **Reason** |
| --- | --- | --- | --- | --- | --- |
| Joseph f standing, martin o ongas, caroline ogwang, nancy kagwanja | Dosing of ceftriaxone and metronidazole for children with severe acute malnutrition | Clincal pharmacology and theraputics | 104(6),1165-1174 | 10.1002/cpt.1078 | Abstract |
| I. Amon, k.amon, h. Hüller | Pharmacokinetics and therapeutic efficacy of metronidazole at different dosages | International journal of clinical pharmacology and biopharmacy | 16(8), 384-386 |  | Not accessible |
| John s bradley, helen broadhurst , karen cheng , maria mendez | Safety and efficacy of ceftazidime-avibactam plus metronidazole in the treatment of children ≥3 months to <18 years with complicated intra-abdominal infection: results from a phase 2, randomized, controlled trial | The pediatric  infectious disease journal | 38(10), 816-824 | 10.1097/inf.0000000000002392 | Abstract |
| M. R sampson, b. T bloom , a. Arrieta , e. Capparelli , d. K benjamin | Intestinal fatty-acid binding protein and metronidazole response in premature infants | Journal of neonatal-perinatal medicine | 7(3), 223-228 | 10.3233/npm-1477013 | Abstract |
| Matthew w carroll , doosoo jeon, james m mountz, jong doo lee, yeon joo jeong | Efficacy and safety of metronidazole for pulmonary multidrug-resistant tuberculosis | Antimicrobial agents and chemotherapy | 57(8), 3903-3909 | 10.1128/aac.00753-13 | Different domain |
| O søreide, o leinebø, t bergan, k bakkevold, b salvesen, n m ulvik | Comparative pharmacokinetics of metronidazole and tinidazole used as single dose prophylactic agents | Scandinavian journal of gastroenterology | 90(1),97-106 |  | Not accessible |
| A.h lau, k. Emmons, r. Seligsohn | Pharmacokinetics of intravenous metronidazole at different dosages in healthy subjects | International journal of clinical pharmacology, therapy, and toxicology | 29(10), 386-90 |  | Not accessible |
| Edmund v capparelli , robin bricker-ford , m john rogers , james h mckerrow, sharon l reed | Phase i clinical trial results of auranofin, a novel antiparasitic agent | Antimicrobial agents and chemotherapy | 61(1), 1-22 | 10.1128/aac.01947-16 | Abstract |
| Helen s pentikis, nikki adetoro | Two phase 1, open-label, single-dose, randomized, crossover studies to assess the pharmacokinetics, safety, and tolerability of orally administered granules of secnidazole (2 g) in healthy female volunteers under different administration conditions | Clinical pharmacology in drug development | 7(5), 543-553 | 10.1002/cpdd.406 | Absract |
| F. E cunningham, d. M kraus, l. Brubaker, j. H fischer | Pharmacokinetics of intravaginal metronidazole gel | Journal of clinical pharmacology | 34(11), 1060-1065 | 10.1002/j.1552-4604.1994.tb01981.x | Abstract |
| Pierre-pascal poulet , danielle duffaut, pierre barthet, ivan brumpt | Concentrations and in vivo antibacterial activity of spiramycin and metronidazole in patients with periodontitis treated with high-dose metronidazole and the spiramycin/metronidazole combination | The journal of antimicrobial chemotherapy | 55(3), 347-351 | 10.1093/jac/dki013 | Abstract |
| M. V dahl, m. Jarratt, d. Kaplan, m. R tuley, m. D baker | Once-daily topical metronidazole cream formulations in the treatment of the papules and pustules of rosacea | Journal of the american academy of dermatology | 45(5), 723-730 | 10.1067/mjd.2001.116219 | Different domain |
| Kelly a sprandel, george l drusano, david w hecht, john c rotschafer, larry h danziger, keith a rodvold | Population pharmacokinetic modeling and monte carlo simulation of varying doses of intravenous metronidazole | Diagn microbiol infect dis | 55(4), 303-9 | 10.1016/j.diagmicrobio.2006.06.013 | Abstract |
| Soraia tahan, lígia cristina f l melli, carolina santos mello, mírian silva c rodrigues, humberto bezerra filho, mauro b de morais | Effectiveness of trimethoprim-sulfamethoxazole and metronidazole in the treatment of small intestinal bacterial overgrowth in children living in a slum | Journal of pediatric gastroenterology and nutrition | 57(3), 316-318 | 10.1097/mpg.0b013e3182952e93 | Abstract |
| K m deppermann, m boeckh, s grineisen, f shokry, k borner, p koeppe, c krasemann, j wagner, h lode | Combination effects of ciprofloxacin, clindamycin, and metronidazole intravenously in volunteers | The american journal of medicine | 87(5), 46-48 | 10.1016/0002-9343(89)90020-x | Abstract |
| Noha m badawi , mona a elkafrawy , rania m yehia , dalia a attia | Clinical comparative study of optimized metronidazole loaded lipid nanocarrier vaginal emulgel for management of bacterial vaginosis and its recurrence | Drug delivery | 28(1), 814-825 | 10.1080/10717544.2021.1912211 | Different domain |
| Stephani l stancil, robin e pearce , rachel f tyndale , gregory l kearns , carrie a vyhlidal , j steven leeder , susan abdel-rahman | Evaluating metronidazole as a novel, safe cyp2a6 phenotyping probe in healthy adults | British journal of clinical pharmacology | 85(5), 960-969 | 10.1111/bcp.13884 | Different domain |
| C. Hoffmann, n. Focke, g. Franke, m. Zschiesche, w. Siegmund | Comparative bioavailability of metronidazole formulations (vagimid) after oral and vaginal administration | International journal of clinical pharmacology and therapeutics | 33(4), 232-239 |  | Not accessible |
| S. A calafatti, a. Dos santos, c. M da silva, m deguer, a. F carvalho jr, f d mendes, j. G ferraz, | Transfer of metronidazole to gastric juice: impact of helicobacter pylori infection and omeprazole | Scandinavian journal of gastroenterology | 35(7), 699-704 | 10.1080/003655200750023354 | Absract |
| B. Lembcke, u. R fölsch, w. F caspary, r. Ebert, w. Creutzfeldt | Influence of metronidazole on the breath hydrogen response and symptoms in acarbose-induced malabsorption of sucrose | Digestion | 25(3), 186-193 | 10.1159/000198828 | Different domain |
| Y. Levy, s. A berger, a. Gorea, a. Shnaker, r. Orda | Penetration of clindamycin and metronidazole into inflamed appendiceal tissue | The european journal of surgery | 162(8), 633-635 |  | Not accessible |
| M. C sullivan, c. H nightingale, r. Quintiliani, k. R sweeney | Comparison of the pharmacodynamic activity of cefotaxime plus metronidazole with cefoxitin and ampicillin plus sulbactam | Pharmacotherapy | 15(4), 479-486 |  | Not accessible |
| J. Sjölin, a. Lilja, n. Eriksson, p. Arneborn, o. Cars | Treatment of brain abscess with cefotaxime and metronidazole: prospective study on 15 consecutive patients | Clinical infectious diseases : an official publication of the infectious diseases society of america | 17(5), 857-63 | 10.1093/clinids/17.5.857 | Different domain |
| Emil gatchev, manfred bräter, christian de mey | Bioequivalence of a novel oral metronidazole formulation | Arzneimittel-forschung | 56(8), 612-616 | 10.1055/s-0031-1296760 | Different domain |
| A. J carcas, p. Guerra, j. Frias, a. Soto, a. Fernandez-aijón, c. Montuenga, c. Govantes | Gender differences in the disposition of metronidazole | International journal of clinical pharmacology and therapeutics | 39(5), 213-218 | 10.5414/cpp39213 | Abstract |
| J. S solomkin, e. P dellinger, j. M bohnen, o. D rostein | The role of oral antimicrobials for the management of intra-abdominal infections | New horizons | 6(2), 46-52 |  | Not accessible |
| J. H solhaug, t bergan, o leinebø, a r rosseland, l spada, f vaagenes | The pharmacokinetics of one single preoperative dose of metronidazole or tinidazole | Scandinavian journal of gastroenterology | 90(1), 89-96 |  | Not accessible |
| P k miani, c do nascimento, s sato, a v filho, m j v da fonseca, v pedrazzi | In vivo evaluation of a metronidazole-containing gel for the adjuvant treatment of chronic periodontitis: preliminary results | European journal of clinical microbiology & infectious diseases | 31(7), 1611-1618 | 10.1007/s10096-011-1484-7 | Different domain |
| G r davies, m e wilkie, d s rampton | Effects of metronidazole and misoprostol on indomethacin-induced changes in intestinal permeability | Digestive diseases and sciences | 38(3), 417-425 | 10.1007/bf01316493 | Different domain |
| M. Pellegrini, r. Urso, g giorgi, p. Bayeli, g. Marzocca, d. Cerretani | Is a long-term ranitidine-based triple therapy against helicobacter pylori only a heritage of the past? A prospective, randomized clinicopharmacological study | Alimentary pharmacology & therapeutics | 22(4), 343-348 | 10.1111/j.1365-2036.2005.02575.x | Absract |
| Seeprarani rath, ashmita ramanah, charles bon, isadore kanfer | Application of a dermatopharmacokinetic (dpk) method for bioequivalence assessment of topical metronidazole creams | Journal of pharmacy & pharmaceutical sciences | 23(1), 437-450 | 10.18433/jpps31534 | Abstract |
| S bielecka-grzela, a klimowicz | Application of cutaneous microdialysis to evaluate metronidazole and its main metabolite concentrations in the skin after a single oral dose | Journal of clinical pharmacy and therapeutics | 28(6), 465-469 | 10.1046/j.0269-4727.2003.00516.x | Abstract |
| M. De freitas silva, s. G schramm, e. K kano, e. E m koono, j. L manfio, v. Porta, c. H dos reis serra | Metronidazole immediate release formulations: a fasting randomized open-label crossover bioequivalence study in healthy volunteers | Arzneimittel-forschung | 62(10), 490-495 | 10.1055/s-0032-1321873 | Abstract |
| M p okamoto, a chin, m a gill, a e yellin, t v berne, p n heseltine, m d appleman, c a knupp, d a sclar | Analysis of cefepime tissue penetration into human appendix | Pharmacotherapy | 11(5), 353-358 |  | Not accessible |
| D j walters , j s solomkin, j a paladino | Cost effectiveness of ciprofloxacin plus metronidazole versus imipenem-cilastatin in the treatment of intra-abdominal infections | Pharmacoeconomics | 16(2), 551-561 | 10.2165/00019053-199916050-00011 | Different domain |
| C e haas , d c kaufman, r c dicenzo | Effects of metronidazole on hepatic cyp3a4 activity | Pharmacotherapy | 21(10), 1192-1195 | 10.1592/phco.21.15.1192.33896 | Different domain |
| P. T pollak | A liquid chromatography assay for the study of serum and gastric juice metronidazole concentrations in the treatment of helicobacter pylori | Therapeutic drug monitoring | 18(6), 678-687 | 10.1097/00007691-199612000-00009 | Abstract |
| D kaner, j-p bernimoulin, t dietrich, b-m kleber, a friedmann | Calprotectin levels in gingival crevicular fluid predict disease activity in patients treated for generalized aggressive periodontitis | Journal of periodontal research | 46(4), 417-426 | 10.1111/j.1600-0765.2011.01355.x | Different domain |
| T azuma, s ito, h suto, y ito, h miyaji, y yamazaki, t kato, m kuriyama | Pharmacokinetics of clarithromycin in helicobacter pylori eradication therapy in patients with liver cirrhosis | Alimentary pharmacology & therapeutics | 14(1), 216-222 | 10.1046/j.1365-2036.2000.014s1216.x | Abstract |
| De qiu zhu, kai li hu, wei xing tao, liang feng, hu duan, xin guo jiang, jun chen | Evaluation of the bioequivalence and pharmacokinetics of two formulations of secnidazole after single oral administration in healthy volunteers | Arzneimittel-forschung | 57(11), 723-726 | 10.1055/s-0031-1296674 | Different domain |
| Jean-marc bohbot, eric vicaut, didier fagnen, michel brauman | Treatment of bacterial vaginosis: a multicenter, double-blind, double-dummy, randomised phase iii study comparing secnidazole and metronidazole | Infectious diseases in obstetrics and gynecology | 2010(1), 1-6 | 10.1155/2010/705692 | Different domain |
| J m badia, r de la torre, m farré, r gaya, f martínez-ródenas, j j sancho, a sitges-serra | Inadequate levels of metronidazole in subcutaneous fat after standard prophylaxis | The british journal of surgery | 82(4), 479-482 | 10.1002/bjs.1800820417 | Abstract |
| M schwarz, r isenmann, j thomsen, w gaus, h g beger | Efficacy of oral ofloxacin for single-dose perioperative prophylaxis in general surgery--a controlled randomized clinical study | Langenbeck's archives of surgery | 386(6), 397-401 | 10.1007/s004230100245 | Different domain |
| Jean spénard, christian aumais, julie massicotte, claude tremblay, marc lefebvre | Influence of omeprazole on bioavailability of bismuth following administration of a triple capsule of bismuth biskalcitrate, metronidazole, and tetracycline | Journal of clinical pharmacology | 44(6), 640-645 | 10.1177/0091270004265643 | Abstract |
| Borje darpo, hongqi xue, nikki adetoro, barbara g matthews, helen s pentikis | Thorough qt/qtc evaluation of the cardiac safety of secnidazole at therapeutic and supratherapeutic doses in healthy individuals | Journal of clinical pharmacology | 58(3), 286-293 | 10.1002/jcph.1014 | Different domain |
| Y h choe, s k kim, b k son, d h lee, y c hong, s h pai | Randomized placebo-controlled trial of helicobacter pylori eradication for iron-deficiency anemia in preadolescent children and adolescents | Helicobacter | 4(2), 135-139 | 10.1046/j.1523-5378.1999.98066.x | Abstract |
| C j lloyd, p d earl | Metronidazole: two or three times daily--a comparative controlled clinical trial of the efficacy of two different dosing schedules of metronidazole for chemoprophylaxis following third molar surgery | The british journal of oral & maxillofacial surgery | 32(3), 165-167 | 10.1016/0266-4356(94)90102-3 | Different domain |
| K engel , w schmidt, h g sonntag, f kees | Comparative clinical and pharmacokinetic aspects of cefotetan versus cefoxitin plus metronidazole in vaginal hysterectomy | Chemioterapia : international journal of the mediterranean society of chemotherapy | 7(4), 256-260 |  | Not accessible |
| H g voesten , j e degener, p k dijkstra, l van der goot, a agema, b sikkema, a l van der meer | Optimizing antimicrobial prophylaxis in reconstructive vascular surgery | Vasa. Zeitschrift fur gefasskrankheiten | 22(4), 342-346 |  | Not accessible |
| M e moraes, m de a pierossi, m o moraes, f f bezerra, c m da silva, h b dias, m n muscará, g de nucci, j pedrazzoli júnior | Short-term sucralfate administration does not alter the absorption of metronidazole in healthy male volunteers | International journal of clinical pharmacology and therapeutics | 34(10), 433-437 |  | Not accessible |
| W wu, p s cannon, w yan, y tu, d selva, j qu | Effects of merogel coverage on wound healing and ostial patency in endonasal endoscopic dacryocystorhinostomy for primary chronic dacryocystitis | Eye | 25(6), 746-753 | 10.1038/eye.2011.44 | Different domain |
| J s solomkin 1, h h reinhart, e p dellinger, j m bohnen, o d rotstein, s b vogel, h h simms, c s hill, h s bjornson, d c haverstock, h o coulter, r m echols | Results of a randomized trial comparing sequential intravenous/oral treatment with ciprofloxacin plus metronidazole to imipenem/cilastatin for intra-abdominal infections. The intra-abdominal infection study group | Annals of surgery | 223(3), 303-315 | 10.1097/00000658-199603000-00012 | Abstract |
| S a zelenitsky, r e silverman, h duckworth, g k harding | A prospective, randomized, double-blind studyof single high dose versus multiple standard dose gentamicin both in combination withmetronidazole for colorectal surgicalprophylaxis | The journal of hospital infection | 46(2), 135-140 | 10.1053/jhin.2000.0814 | Different domain |
| R rohwedder, f bonadeo, m benati, g ojea quintana, h schlecker, c vaccaro | Single-dose oral ciprofloxacin plus parenteral metronidazole for perioperative antibiotic prophylaxis in colorectal surgery | Chemotherapy | 39(3), 218-224 | 10.1159/000239129 | Different domain |
| T midtvedt, b carlstedt-duke, t høverstad, e lingaas, e norin, h saxerholt, m steinbakk | Influence of peroral antibiotics upon the biotransformatory activity of the intestinal microflora in healthy subjects | European journal of clinical investigation | 16(1), 7-11 | 10.1111/j.1365-2362.1986.tb01300.x | Different domain |
| Helen s pentikis , nikki adetoro , carol j braun | Lack of a pharmacokinetic interaction between sym-1219 granules containing 2 grams of secnidazole and a combined oral contraceptive in a phase 1, randomized, open-label study in healthy female volunteers | Advance therapy | 33(12), 2229-2241 | 10.1007/s12325-016-0411-9 | Different domain |
| C a furnée, c e west, f van der haar, j g hautvast | Effect of intestinal parasite treatment on the efficacy of oral iodized oil for correcting iodine deficiency in schoolchildren | The american journal of clinical nutrition | 66(6), 1422-1427 | 10.1093/ajcn/66.6.1422 | Different domain |
| Khin-maung-u, t d bolin, v m duncombe, s p pereira, myo-khin, nyunt-nyunt-wai, j m linklater | Effect of short-term intermittent antibiotic treatment on growth of burmese (myanmar) village children | Lancet | 336(8723), 1090-1093 | 10.1016/0140-6736(90)92569-4 | Different domain |
| C peiper, m seelig, k h treutner, v schumpelick | Low-dose, single-shot perioperative antibiotic prophylaxis in colorectal surgery | Chemotherapy | 43(1), 54-59 | 10.1159/000239536 | Different domain |
| D m paton, d r webster | Comparative bioavailability of two tablet preparations of metronidazole | International journal of clinical pharmacology research | 8(40), 227-229 |  | Not accessible |
| M zschiesche, n focke, c hoffmann, g franke, w siegmund | Bioavailability of metronidazole vaginal tablets (vagimid) | International journal of clinical pharmacology, therapy, and toxicology | 30(11), 485-486 |  | Not accessible |
| Van zanten, goldie, hollingsworth, silletti, richardson | Secretion of intravenously administered antibiotics in gastric juice: implications for management of helicobacter pylori | Journal of clinical pathology | 45(3), 225-227 | 10.1136/jcp.45.3.225 | Different domain |
| Stephanie, p. D. Marks, h. R. | Pharmacokinetics of topically applied metronidazole in two different formulations | Skin pharmacology | 10(1), 28-33 |  | Not accessible |
| Legge, j. S. Reid, t. M. Palmer, j. B. | Clinical efficacy, tolerance and pharmacokinetics of temocillin in patients with respiratory tract infections | Drugs | 29(5), 118-121 | 10.2165/00003495-198500295-00025 | Different domain |
| Ludwig, e. Graber, h. Szekely, e. Csiba | Metabolic interactions of ciprofloxacin | Diagnostic microbiology and infectious disease | 13(2), 135-141 | 10.1016/0732-8893(90)90096-e | Abstract |
| Yoo, j. Reid, d. C. Kimball, a. B. | Metronidazole in the treatment of rosacea: do formulation, dosing, and concentration matter? | Journal of drugs in dermatology | 5(4), 317-319 |  | Not accessible |
| Bhoir, s. Gaikwad, p. Bhagwat, a. Jathar | Steady-state pharmacokinetics of immediate-release and controlled-release metronidazole tablets | International journal of pharmacy and pharmaceutical sciences | 4(3),353-356 |  | Not accessible |
| Kaniwa, n. Aoyagi, n. Ogata, ishii, m. | Application of the nonmem method to evaluation of the bioavailability of drug products | Journal of pharmaceutical sciences | 79(12), 1116-1120 | 10.1002/jps.2600791215 | Abstract |
| Liew, k. B. Loh, g. O. K.tan, y. T. F.peh, k. K. | Pharmacokinetics and bioequivalence evaluation of metronidazole tablets in healthy malaysian volunteers | Latin american journal of pharmacy | 32(1), 43-51 |  | Not accessible |
| Ammon, s. Treiber, g. Kees, f.klotz, u. | Influence of age on the steady state disposition of drugs commonly used for the eradication of helicobacter pylori | Alimentary pharmacology & therapeutics | 14(6), 759-766 | 10.1046/j.1365-2036.2000.00756.x | Different domain |
| Ezzeldin, e.el-nahhas, t. M. | New analytical method for the determination of metronidazole in human plasma: application to bioequivalence study | Tropical journal of pharmaceutical research | 11(5), 799-805 |  | Not accessible |
| Kim, m. Jeong, y.park, j. Moon, s. | Pharmacokinetic interaction between tegoprazan and metronidazole/tetracycline/bismuth in healthy korean subjects | Clinical pharmacology and therapeutics | 107(6), 59-65 |  | Not accessible |
| Randell, r. L. Balevic, s. J. Greenberg, r. G. Cohen-wolkowiez, m. Thompson, e. J. Venkatachalam, s. Smith, m. J. | Opportunistic dried blood spot sampling validates and optimizes a pediatric population pharmacokinetic model of metronidazole | Antimicrobial agents and chemotherapy | 2024(4), 153-323 | 10.1128/aac.01533-23 | Abstract |
| Abujamel, t. Cadnum, j. L. Jury, l. A.sunkesula, v. C.kundrapu, s.jump, r. L. Stintzi, a. C.donskey, c. J. | Defining the vulnerable period for re-establishment of clostridium difficile colonization after treatment of c. Difficile infection with oral vancomycin or metronidazole | Plos one | 8(10), 69-76 | 10.1371/journal.pone.0076269 | Abstract |
| Loft, s. Døssing, m. Poulsen, h. E. Sonne, j. Olesen, k. L.simonsen, k. Andreasen, p. B. | Influence of dose and route of administration on disposition of metronidazole and its major metabolites | European journal of clinical pharmacology | 30(4), 467-473 | 10.1007/bf00607962 | Abstract |
| Goel, v. Jain, a. Sharma, g. Jhajharia, a.agarwal, v. K. Ashdhir, p. Pokharna, r. Chauhan, v. | Evaluating the efficacy of nitazoxanide in uncomplicated amebic liver abscess | Indian journal of gastroenterology | 40(3), 272-280 | 10.1007/s12664-020-01132-w | Different domain |
| Miyachi, y. Yamasaki, k. Fujita, t. Fujii, c. | Metronidazole gel (0.75%) in japanese patients with rosacea: a randomized, vehicle-controlled, phase 3 study | Journal of dermatology | 49(3),330-340 | 10.1111/1346-8138.16254 | Abstract |
| Pogorelić, z. Silov, n. Jukić, m. Elezović baloević, s. Poklepović peričić, t.jerončić, a. | Ertapenem monotherapy versus gentamicin plus metronidazole for perforated appendicitis in pediatric patients | Surgical infections | 20(8), 625-630 | 10.1089/sur.2019.025 | Different domain |
| Muzny, c. A. Schwebke, j. R.nyirjesy, p.kaufman, g.mena, l. A.lazenby, g. B.van gerwen, o. T. | Efficacy and safety of single oral dosing of secnidazole for trichomoniasis in women: results of a phase 3, randomized, double-blind, placebo-controlled, delayed-treatment study | Clinical infectious diseases | 73(6), 1282-1289 | 10.1093/cid/ciab242 | Different domain |
| Thomas, g. M.rauth, a. M.bush, r. S.black, b. E.cummings, b. J. | A toxicity study of daily dose metronidazole with pelvic irradiation | Cancer clinical trials | 3(3), 223-230 |  | Not accessible |
| Apostolopoulos, p.ekmektzoglou, k.georgopoulos, s.chounta, e.theofanopoulou, a.kalantzis, c.vlachou, e.tsibouris, p. | 10-day versus 14-day quadruple concomitant nonbismuth therapy for the treatment of helicobacter pylori infection: results from a randomized prospective study in a high clarithromycin resistance country | Journal of clinical gastroenterology | 54(6), 522-527 | 10.1097/mcg.0000000000001328 | Abstract |
| Kaartinen, t.tornio, a.niemi, m.backman, j. | Metronidazole does not significantly elevate plasma concentrations of the cyp2c9 substrate fluvastatin in healthy volunteers | European journal of clinical pharmacology | 78(8), 79-80 | 10.1007/s00228-022-03333 | Abstract |
| Deppermann, k. M.boeckh, m.grineisen, s.shokry, f.borner, k.koeppe, p.krasemann, c.wagner, j.lode, h. | Brief report: combination effects of ciprofloxacin, clindamycin, and metronidazole intravenously in volunteers | American journal of medicine | 87(5), 46-48 |  | Not accessible |
| Taib, a.ruzicka, t.berth-jones, j.jacovella, j.harvey, a. | Assessing treatment and relapse in rosacea after cessation of treatment with ivermectin 1% cream vs metronidazole 0.75% cream | Journal of the dermatology nurses' association | 12(2), 48-51 |  | Not accessible |
| Dubberke, e. R.gerding, d.kelly, c.garey, k. W.rahav, g.mosley, a.tipping, r.dorr, m. B.guris, d. | Efficacy of bezlotoxumab in patients receiving metronidazole, vancomycin, or fidaxomicin for treatment of clostridium difficile infection (cdi) | Open forum infectious diseases | 3(6), 1-68 | 10.1093/ofid/ofw194.45 | Different domain |
| Wright, e. K.kamm, m. A.de cruz, p.hamilton, a. L.selvaraj, f.princen, f.gorelik, a.liew, d.prideaux, l. | Anti-tnf therapeutic drug monitoring in postoperative crohn's disease | Journal of crohn's & colitis | 12(6), 653-661 | 10.1093/ecco-jcc/jjy003 | Abstract |
| Bhansali, s. G.mullane, k.ting, l. S.leeds, j. A.dabovic, k.praestgaard, j.pertel, p. | Pharmacokinetics of lff571 and vancomycin in patients with moderate clostridium difficile infections | Antimicrobial agents and chemotherapy | 59(3), 1441-1445 | 10.1128/aac.04252-14 | Different domain |
| Dean, g.soni, s.pitt, r.ross, j.sabin, c.whetham, j. | Treatment of mild-to-moderate pelvic inflammatory disease with a short-course azithromycin-based regimen versus ofloxacin plus metronidazole: results of a multicentre, randomised controlled trial | Sexually transmitted infections | 97(3), 177-182 | 10.1136/sextrans-2020-054468 | Review |
| Sime, f. B.roberts, m. S.tiong, i. S.gardner, j. H.lehman, s.peake, s. L.hahn, u.warner, m. S.roberts, j. A. | Can therapeutic drug monitoring optimize exposure to piperacillin in febrile neutropenic patients with haematological malignancies? A randomized controlled trial | Journal of antimicrobial chemotherapy | 70(8), 2369-2375 | 10.1093/jac/dkv123 | Review |
| Kim, b.kim, j.huh, c. | Optimal eradication regimen for clarithromycin resistant helicobacter pylori infection in korea: a prospective randomized trial comparing bismuth quadruple therapy and metronidazole triple therapy | Helicobacter | 22(11), 47-48 | 10.1111/hel.12416 | Review |
| Euctr, b. E. | Compare ceftazidime avibactam + metronidazole vs meropenem for hospitalized adults with complicated intra-abdominal infections | Infectious disease | 2019(3),1-4 |  | Not accessible |
| Simon, p.petroff, d.dorn, c.ehmann, l.kloft, c.prettin, c.dietrich, a.zeitlinger, m.kees, f.wrigge, h. | Measurement of soft tissue drug concentrations in morbidly obese and non-obese patients – a prospective, parallel group, open-labeled, controlled, phase iv, single center clinical trial | Contemporary clinical trials communications | 15(12), 1-6 | 10.1016/j.conctc.2019.100375 | Different domain |
| Awada, g.schwarze, j. K.reijmen, e.goyvaerts, c.fasolino, g.aspeslagh, s.neyns, b. | 20p interim safety and efficacy results of a phase ii clinical trial on trametinib and low-dose dabrafenib in patients with advanced brafv600 wild-type melanoma | Annals of oncology | 31(1),10-57 | 10.1016/j.annonc.2020.01.039 | Different domain |
| Schwebke, j.carter, bwaldbaum, a.price, c.castellarnau, a.paull, j.mccloud, p.kinghorn, g. | Results of a phase 3, randomized, double-blind, placebo-controlled study to evaluate the efficacy and safety of astodrimer gel for prevention of recurrent bacterial vaginosis | American journal of obstetrics and gynecology | 221(6), 672-673 | 10.1016/j.ajog.2019.10.087 | Different domain |
| Haifer, c.saikal, a.paramsothy, s.borody, t. J.ghaly, s.kaakoush, n. O.leong, r. W. | Lyophilised oral faecal microbiota transplantation in the management of ulcerative colitis (lotus study): results from the induction phase of a randomized controlled trial | Journal of gastroenterology and hepatology | 35(1), 116-117 | 10.1111/jgh.15271 | Different domain |
| Hillier, s. L.morgan, f. G.waldbaum, a. S.schwebke, j. R.nyirjesy, p.adetoro, n.braun, c. J. | A phase 2 randomized, double-blind, placebo-controlled study to evaluate the effectiveness and safety of single, oral doses of sym-1219, a granule formulation containing 1 and 2 gram doses of secnidazole, for the treatment of women with bacterial vaginosis | American journal of obstetrics and gynecology | 213(6), 885 |  | Not accessible |
| Lurie, l.gutkin, e. | The use of octreotide in refractory gastrointestinal bleeding due to crohn's disease | American journal of gastroenterology | 111(3), 666- 670 | 10.1038/ajg.2016.365 | Abstract |
| Ford, c.litcofsky, k.mcgovern, b.pardi, d.nathan, r.hansen, v. | Engraftment of investigational microbiome drug, ser-262, in subjects receiving vancomycin is associated with reduced rates of recurrence after primary clostridium difficile infection (cdi) | Open forum infectious diseases | 6(2), 547-548 | 10.1093/ofid/ofz360.1367 | Different domain |
| Johnson, s.sambol, s.best, e.wilcox, m.gerding, d.eves, k.pedley, a.kartsonis, n.dorr, m. B. | Efficacy of bezlotoxumab in patients infected with strains of clostridium difficile associated with poor outcomes | Open forum infectious diseases | 3(8), 599 | 10.1093/ofid/ofwl72.1662 | Different domain |
| Mihai, c.mihai, b.cardoneanu, a.dranga, m.gavrilescu, o.drug, v.prelipcean, c. | First line helicobacter pylori eradication in dyspeptic patients | Journal of gastrointestinal and liver diseases | 26(9), 62-63 |  | Not accessible |
| Liou, j.fang, y.chen, c.bair, m.lin, j.wu, m. | Optimized 14-day sequential versus 10-day bismuth quadruple therapy containing high dose esomeprazole in the first-line treatment of helicobacter pylori-a multicenter randomized trial | United european gastroenterology journal | 5(5), 86 | 10.1177/2050640617725668 | Abstract |
| Apostolopoulos, p.ekmektzoglou, k.vlachou, e.chounta, e. Dimopoulos, k.theofanopoulou, a.toumpelis, k. | Day versus 14-day quadruple concomitant non bismuth therapy for the treatment of helicobacter pylori infection: results from a greek randomized prospective study | United european gastroenterology journal | 6(8), 731-732 | 10.1177/2050640618792819 | Abstract |
| Schwartz, j.justman, j.van der straten, a.smith-mccune, k.wheeless, a.littlefield, s.sykes, c. | In vivo drug-drug interactions between tenofovir gel and three commonly used vaginal products | Aids research and human retroviruses | 32(10(, 353 | 10.1089/aid.2016.5000. | Abstract |
| Liou, j. M.chen, c. C.fang, y. J.chen, p. Y.lin, j. T.wu, m. S. | Levofloxacin sequential therapy versus bismuth quadruple therapy in the second-line and third-line treatment of helicobacter pylori infection- a multicenter randomized trial | Gastroenterology | 158(6), 570 | 10.1016/s0016-5085(20)32131-4 | Different domain |
| Everett, s. M.drake, i. M.white, k. L.mapstone, n. P.chalmers, d. M.schorah, c. J.axon, a. T. | Antioxidant vitamin supplements do not reduce reactive oxygen species activity in helicobacter pylori gastritis in the short term | British journal of nutrition | 87(1), 3-11 | 10.1079/bjn2001477 | Different domain |
| Abd-elsalam, s.shehata, m. A. H.elmesseri, h.talaat, r. | Randomized controlled study of a novel triple nitazoxanide (ntz) containing therapeutic regimen versus the traditional regimen for eradication of helicobacter pylori infection | United european gastroenterology journal | 5(5),1-5 | 10.1177/2050640617725676 | Different domain |
| Kim, b. J.lee, h.lee, y. C.jeon, s. W.kim, g. H.kim, h. S.sung, j. K.lee, d. H. | Ten-day concomitant, 10-day sequential, and 7-day triple therapy in first-line treatment of helicobacter pylori infection: a randomized nationwide trial in korea | Gut and liver | 2019(9), 1-6 | 10.5009/gnl19136 | Abstract |
| Healy, d. P.dansereau, r. J.dunn, a. B.clendening, c. E.mounts, a. W.deepe, g. S. | Reduced tetracycline bioavailability caused by magnesium aluminum silicate in liquid formulations of bismuth subsalicylate | Annals of pharmacotherapy | 31(12), 1460-1464 | 10.1177/106002809703101203 | Different domain |
| Barakat, s. H.mahfouz, a.el-gendi, s. Hassen, a. | Comparative study of h.pylori eradication rates of high and frequent dose of omeprazole and amoxicillin dual therapy versus standard triple therapy in children | Pediatrics | 146(1), 587-591 | 10.1097/mpg.0000000000002403 | Abstract |
| Chen, q.zhang, w.fu, q. Y.liang, x.liu, w. Z.xiao, s. D.lu, h. | Rescue therapy for helicobacter pylori eradication: a randomized clinical trial of amoxicillin or tetracycline in bismuth quadruple therapy | Journal of digestive diseases | 17(11), 106-110 | 10.1111/1751-2980.12389 | Abstract |
| Hollender, l. F.bahnini, j.de manzini, n.lau, w. Y.fan, s. T.hermansyur, k.benny, p. | multicentric study of netilmicin once daily versus thrice daily in patients with appendicitis and other intra-abdominal infections | Journal of antimicrobial chemotherapy | 23(5), 773-783 | 10.1093/jac/23.5.773 | Different domain |
| Resina, e. Isbert, j. P. | Rescue therapy with furazolidone in patients with at least five eradication treatment failures and multi-resistant h. Pylori infection | Antibiotics | 10(9), 11-18 | 10.3390/antibiotics10091028 | Abstract |
| Offman, e. M.kassir, n.anderson, p.kalfus, i. N. | Pharmacokinetics and exposure-response of rhb-105, a novel fixed-dosed rifabutin-based combination (rifabutin, amoxicillin, and omeprazole) treatment of helicobacter pylori | Gastroenterology | 158(6),137-138 | 10.1016/s0016-5085(20)32132-6 | Different domain |
| Wang, y.he, y.li, w.li, h.tang, l.dai, x. | Evaluation of ornidazole tablets bioequivalence in chinese healthy participants under fasted and fed conditions using pharmacokinetic parameters | Drugs | 24(4), 28-34 | 10.1007/s40268-024-00457-7 | Abstract |
| Chen, q.long, x.ji, y.liang, x.li, d.gao, h.xu, b.liu, m.chen, y.sun, y. | Susceptibility-based tailored vs. Empiric amoxicillin modified bismuth quadruple therapy as helicobacter pylori therapy: a multicenter randomized controlled trial | United european gastroenterology journal | 6(8), 325-326 | 10.1177/2050640618792819 | Abstract |
| Song, z.zhou, l.zhang, j.he, l.bai, p.xue, y. | Hybrid therapy as first-line regimen for helicobacter pylori eradication in populations with high antibiotic resistance rates | Helicobacter | 21(5), 382-388 | 10.1111/hel.12294 | Different domain |
| Cheng, k.newell, p.chow, j. W.broadhurst, h.wilson, d.yates, k.wardman, a. | Safety profile of ceftazidime-avibactam: pooled data from the adult phase ii and phase iii clinical trial programme | Drug safety | 43(8),751-766 | 10.1007/s40264-020-00934-3 | Review |
| Louie, t. J.miller, m. A.mullane, k. M.weiss, k.lentnek, a.golan, y.gorbach, s.sears, p.shue, y. K. | Fidaxomicin versus vancomycin for clostridium difficile infection | New england journal of medicine | 364(5), 422-431 | 10.1056/nejmoa0910812 | Different domain |
| Wilcox, m. H.gerding, d. N.poxton, i. R.kelly, c.nathan, r.birch, t.cornely, o. A.rahav, g.bouza, e.lee, c. | Bezlotoxumab for prevention of recurrent clostridium difficile infection | New england journal of medicine | 376(4), 305-317 | 10.1056/nejmoa1602615 | Different domain |
| Espin-basany, e.sanchez-garcia, j. L.lopez-cano, m.lozoya-trujillo, r.medarde-ferrer, m.armadans-gil, l.alemany-vilches, l.armengol-carrasco, m. | Prospective, randomised study on antibiotic prophylaxis in colorectal surgery. Is it really necessary to use oral antibiotics? | International journal of colorectal disease | 20(6), 542-546 | 10.1007/s00384-004-0736-8 | Abstract |
| Gerli, s.rossetti, d.di renzo, g. C. | A new approach for the treatment of bacterial vaginosis: use of polyhexamethylene biguanide. A prospective, randomized study | European review for medical and pharmacological sciences | 7(5),127-130 |  | Not accessible |
| Hammami, m. M.de padua, s. J. S.hussein, r.al gaai, e.khodr, n. A.al-swayeh, r.alvi, s. N.binhashim, n. | Generic-reference and generic-generic bioequivalence of forty-two, randomly-selected, on-market generic products of fourteen immediate-release oral drugs | Bmc pharmacology & toxicology | 18(1), 74-78 | 10.1186/s40360-017-0182-1 | Abstract |
| Wu, d.qian, z. Y.guo, t.tang, w.xiang, y.zheng, h. | Determination of cefazedone in human plasma by high performance liquid chromatography-tandem mass spectrometry: application to a pharmacokinetic study on chinese volunteers | Journal of chromatography. B, analytical technologies in the biomedical and life sciences | 878(28), 2911-2915 | 10.1016/j.jchromb.2010.08.008 | Different domain |
| Chen, q.zhang, w.fu, q.liang, x.liu, w.xiao, s.lu, h. | Rescue therapy for helicobacter pylori eradication: a randomized non-inferiority trial of amoxicillin or tetracycline in bismuth quadruple therapy | American journal of gastroenterology | 111(12), 1736-1742 | 10.1038/ajg.2016.443 | Different domain |
| Kim, b.huh, c.kim, j. | The optimal eradication regimen for clarithromycin resistant helicobacter pylori in korea: a prospective randomized trial comparing bismuth quadruple therapy and metronidazole triple therapy | Helicobacter | 23(10), 33-34 | 10.1111/hel.12525 | Abstract |
| Tanabe, h.ando, k.sato, k.ito, t.goto, m.sato, t.fujinaga, a.kawamoto, t.utsumi, t.yanagawa, n. | Efficacy of vonoprazan-based triple therapy for helicobacter pylori eradication: a multicenter study and a review of the literature | Digestive diseases and sciences | 62(11), 3069-3076 | 10.1007/s10620-017-4664-1 | Review |
| Li, x.sun, j.wang, g.zheng, y.yan, b.xie, h.gu, y.ren, h. | Determination of secnidazole in human plasma by high-performance liquid chromatography with uv detection and its application to the bioequivalence studies | Biomedical chromatography | 21(3),304-309 | 10.1002/bmc.758 | Different domain |
| Wang, g. J.gao, x. Y.wu, y.he, h. Q.yu, y.qin, h. H.shen, w. T. | Evaluation of the efficacy and tolerance of artemether emulsion for the treatment of papulopustular rosacea: a randomized pilot study | Journal of dermatological treatment | 30(8), 809-812 | 10.1080/09546634.2019.1610549 | Different domain |
| Figueras-felip, j.basilio-bonet, e.lara-eisman, f.caride-garcia, p.isamat-baro, e.fava-bargallo, p.rosell-abaurrea, f. | Oral is superior to systemic antibiotic prophylaxis in operations upon the colon and rectum | Surgery, gynecology & obstetrics | 158(4),359-362 |  | Not accessible |
| Alves, a. J.aquino, t. M.neto, j. L. C.filho, s. D. S.junor, h. J.gaspar, f. L.luna, mcmmalves, c. J.alves, a. Q.oliveira, c. F. | Bioequivalence between two metronidazole formulations | Latin american journal of pharmacy | 26(2), 266‐269 |  | Not accessible |
| El-mekkaoui, a.zeriouh, m.khannoussi, w.kharrasse, g.abda, n.ismaili, z. | Concomitant therapy, sequential therapy or high dose esomeprazole and amoxicillin dual therapy for first line helicobacter pylori eradication a prospective randomized study | United european gastroenterology journal | 7(8), 1000-1007 | 10.1177/205064061985467 | Abstract |
| Liou, j. M.chen, c. C.fang, y. J.lin, j. T.wu, m. S. | Levofloxacinse quential therapy versus bismuth quadruple therapy in the second-line and third-line treatment of helicobacter pylori : a multicenter randomized trial | United european gastroenterology journal | 7(8), 40-44 | 10.1177/205064061985467 | Abstract |
| Liou, j. M.chen, p. Y.chen, c. C.fang, y. J.bair, m. J.lin, j. T. Wu, m. S. | Levofloxacin sequential therapy versus bismuth quadruple therapy in the second-line and third-line treatment of helicobacter pylori-a multicenter randomized trial | Gut and liver | 68(1), 84‐85 | 10.1136/gutjnl-2019-iddfabstracts.159 | Abstract |
| Vinge, e.andersson, k. E.ando, g.lunell, e. | Biological availability and pharmacokinetics of tinidazole after single and repeated doses | Scandinavian journal of infectious diseases | 15(4), 391‐397 | 10.3109/inf.1983.15.issue-4.10 | Different domain |
| Aekwattanaphol, nattanit ali khumaini mudhar bintang, muhammad paliwal, himanshu srichana, teerapol | Biomolecular interaction of pretomanid and its pro-liposomal formulation with human serum albumin by multi-spectroscopic, surface plasmon resonance and molecular docking approaches | Journal of photochemistry and photobiology a: chemistry | 453(1), 115-636 | 10.1016/j.jphotochem.2024.115636 | Different domain |
| Chavada, vijay d. Bhatt, nejal m. Sanyal, mallika shrivastav, pranav s. | Citrate/melamine functionalized gold nanoparticles for concurrent determination of allopurinol and its major metabolite, oxypurinol in plasma and pharmaceuticals | Journal of industrial and engineering chemistry | 84(1), 141-149 | 10.1016/j.jiec.2019.12.029 | Different domain |
| Genilloud, olga | Actinomycetes: still a source of novel antibiotics | Natural product reports | 34(10), 1203-1232 | 10.1039/c7np00026j | Different domain |
| Moratalla, ángela cotillas, salvador lacasa, engracia fernández-marchante, carmen m. Ruiz, sonia valladolid, ana cañizares, pablo | Occurrence and toxicity impact of pharmaceuticals in hospital effluents: simulation based on a case of study | Process safety and environmental protection | 168(1), 10-21 | 10.1016/j.psep.2022.09.066 | Abstract |
| Yan, xiangjie ma, feihe chen, qixian gou, xiangbo li, xiaohui | Construction of size-transformable supramolecular nano-platform against drug-resistant colorectal cancer caused by fusobacterium nucleatum | Chemical engineering journal | 450(1), 137-145 | 10.1016/j.cej.2022.137605 | Different domain |
| Azanu, david styrishave, bjarne darko, godfred weisser, johan juhl | Occurrence and risk assessment of antibiotics in water and lettuce in ghana | Science of the total environment | 622(1), 293-305 | 10.1016/j.scitotenv.2017.11.287 | Different domain |
| Castaño-trias, m. Rodríguez-mozaz, s. Buttiglieri, g. | A decade of water monitoring in a mediterranean region: pharmaceutical prioritisation for an upgraded analytical methodology | Environmental nanotechnology, monitoring & management | 20(1), 100-115 | 10.1016/j.enmm.2023.100850 | Different domain |
| Chandel, neha ahuja, vishal gurav, ranjit kumar, vinod tyagi, vinay kumar | Progress in microalgal mediated bioremediation systems for the removal of antibiotics and pharmaceuticals from wastewater | Science of the total environment | 825(2), 153-159 | 10.1016/j.scitotenv.2022.153895 | Different domain |
| Chebii, faith k'oreje, kenneth okoth, maurice lutta, samuel | Occurrence and environmental risks of contaminants of emerging concern across the river athi basin, kenya, in dry and wet seasons | Science of the total environment | 914(1), 169-175 | 10.1016/j.scitotenv.2023.169696 | Different domain |
| Coutu, sylvain wyrsch, v. Wynn, h. K. Rossi, l. Barry, d. A. | Temporal dynamics of antibiotics in wastewater treatment plant influent | Science of the total environment | 458(1), 20-26 | 10.1016/j.scitotenv.2013.04.017 | Different domain |
| Jones, ronald n. | Review of the in vitro spectrum of activity of imipenem | The american journal of medicine | 78(6),22-32 | 10.1016/0002-9343(85)90098-1 | Review |
| Keithley, joyce k. | Histamine h2-receptor antagonists | Nursing clinics of north america | 26(2), 361-373 | 10.1016/s0029-6465(22)00252-3 | Review |
| Kotlinska-lemieszek, aleksandra paulsen, ørnulf kaasa, stein klepstad, pål | Polypharmacy in patients with advanced cancer and pain: a european cross-sectional study of 2282 patients | Journal of pain and symptom management | 48(6), 1145-1159 | 10.1016/j.jpainsymman.2014.03.008 | Different domain |
| Levine, donald p. Mcneil, pamela lerner, stephen a. | Randomized, double-blind comparative study of intravenous ciprofloxacin versus ceftazidime in the treatment of serious infections | The american journal of medicine | 87(5), 160-163 | 10.1016/0002-9343(89)90049-1 | Different domain |
| Marzella, nino | Treatment for multidrug resistant gram-negative infections with cefiderocol (fetroja) | The journal for nurse practitioners | 19(3), 104-109 | /10.1016/j.nurpra.2022.11.026 | Different domain |
| Newshan, gayle sherman, deborah witt | Palliative care: pain and symptom management in persons with hiv/aids | Nursing clinics of north america | 34(1), 131-145 | 10.1016/s0029-6465(22)02366-0 | Review |
| Nord, carl e. Kager, lars heimdahl, anders | Impact of antimicrobial agents on the gastrointestinal microflora and the risk of infections | The american journal of medicine | 76(5), 99-106 | 10.1016/0002-9343(84)90250-x | Abstract |
| Oliphant, catherine m. Eroschenko, kathryn | Antibiotic resistance, part 1: gram-positive pathogens | The journal for nurse practitioner | 11(1), 70-78 | 10.1016/j.nurpra.2014.09.018 | Review |
| Porche, demetrius james | State of the art: antiretroviral and prophylactic treatments in hiv/aids | Nursing clinics of north america | 34(1), 95-112 | 10.1016/s0029-6465(22)02364-7 | Review |
| Roe, valerie a. | Antibiotic resistance: a guide for effective prescribing in women's health | Journal of midwifery & women's health | 53(3), 216-226 | 10.1016/j.jmwh.2008.01.003 | Review |
| Goldschmiedt, markus barnett, cora c. Schwarz, barry e. Karnes, william e. | Effect of age on gastric acid secretion and serum gastrin concentrations in healthy men and women | Gastroenterology | 101(4), 977-990 | 10.1016/0016-5085(91)90724-y | Different domain |
| Catalano, robert b. | Pharmacology of analgesic agents used to treat cancer pain | Seminars in oncology nursing | 1(2), 126-140 | 10.1016/s0749-2081(85)80047-4 | Different domain |
| Bernuau, j. Rueff, b. | Treatment of acute variceal bleeding | Clinics in gastroenterology | 14(1), 185-207 | 10.1016/s0300-5089(21)00644-1 | Different domain |
| Hiemenz, john w. Greene, john n. | Special considerations for the patient undergoing allogeneic or autologous bone marrow transplantation | Oncology clinics of north america | 7(5), 961-1002 | 10.1016/s0889-8588(18)30214-4 | Different domain |
| Shea, kevin w. Cunha, burke a. | Teicoplanin | Medical clinics of north america | 79(4), 833-844 | 10.1016/s0025-7125(16)30042-6 | Review |
| Zuckerman, jerry m. Kaye, kenneth m. | The newer macrolides: azithromycin and clarithromycin | Infectious disease clinics of north america | 9(3), 731-745 | 10.1016/s0891-5520(20)30694-2 | Review |
| Bhatia, monica militano, olga jin, zhezhen figurski, michal shaw, leslie moore, virginia | An age-dependent pharmacokinetic study of intravenous and oral mycophenolate mofetil in combination with tacrolimus for gvhd prophylaxis in pediatric allogeneic stem cell transplantation recipients | Biology of blood and marrow transplantation | 16(3), 333-343 | 10.1016/j.bbmt.2009.10.007 | Different domain |
| Mo, simone thursky, karin chronas, alexandros hall, lisa james, rodney | Metronidazole prescribing practices in australian hospitals: measuring guideline compliance and appropriateness to support antimicrobial stewardship | Journal of infection and public health | 16(1), 90-96 | 10.1016/j.jiph.2023.10.039 | Different domain |
| Saibu, oluwatosin a. Singh, gagandeep omoboyowa, damilola a. Oyejoke, adebimpe k. Olugbodi, sunday a. | Discovery of putative natural compounds inhibitor of the germinant spore receptor cspc in clostridioides difficile infection: gaining insights via in silico and bioinformatics approach | Informatics in medicine unlocked | 42(1), 101-139 | 10.1016/j.imu.2023.101339 | Different domain |
| Bernstein, jack m. Erk, stanley d. | Choice of antibiotics, pharmacokinetics, and dose adjustments in acute and chronic renal failure | Medical clinics of north america | 74(4), 1059-1076 | 10.1016/s0025-7125(16)30536-3 | Review |
| Chakrabarti, suparno mautner, vivien osman, husamcollingham, kathryn e. | Adenovirus infections following allogeneic stem cell transplantation: incidence and outcome in relation to graft manipulation, immunosuppression, and immune recovery | Blood | 100(5), 1619-1627 | 10.1182/blood-2002-02-0377 | Different domain |
| Feingold, david s. Wagner, richard f. | Antibacterial therapy | Journal of the american academy of dermatology | 14(4), 535-548 | 10.1016/s0190-9622(86)80441-8 | Different domain |
| Courvalin, patrice | Interpretive reading of in vitro antibiotic susceptibility tests (the antibiogramme) | Clinical microbiology and infection | 2(1), 26-34 | 10.1111/j.1469-0691.1996.tb00872.x | Different domain |
| Xu, rong wang, quanqiu | Automatic construction of a large-scale and accurate drug-side-effect association knowledge base from biomedical literature | Journal of biomedical informatics | 51(1), 191-199 | 10.1016/j.jbi.2014.05.013 | Review |
| Damasceno, glauciene santana guaraldo, lusiele engstrom, elyne montenegro filha, mariza miranda theme | Adverse reactions to antituberculosis drugs in manguinhos, rio de janeiro, brazil | Clinics | 68(3), 329-337 | 10.6061/clinics/2013(03)oa08 | Different domain |
| Kakar, satinder batra, deepa singh, ramandeep nautiyal, ujjwal | Magnetic microspheres as magical novel drug delivery system: a review | Journal of acute disease | 2(1), 1-12 | 10.1016/s2221-6189(13)60087-6 | Review |
| Glick, michae l. | Medical considerations for dental care of patients with alcohol-related liver disease | The journal of the american dental association | 128(1), 61-70 | 10.14219/jada.archive.1997.0027 | Review |
| Janes, s. M. Barker, k. F. Mak, v. Bell, d. | Invasive pulmonary aspergillosis in an insulin-dependent diabetic | Respiratory medicine | 92(70, 972-975 | 10.1016/s0954-6111(98)90201-3 | Different domain |
| Hübner, martin kusamura, shigeki villeneuve, laurent al-niaimi, ahmed alyami, mohammad balonov, konstantin | Guidelines for perioperative care in cytoreductive surgery (crs) with or without hyperthermic intraperitoneal chemotherapy (hipec): enhanced recovery after surgery (eras®) society recommendations — part i: preoperative and intraoperative management | European journal of surgical oncology | 46(12), 2292-2310 | 10.1016/j.ejso.2020.07.041 | Review |
| Muñoz, p. | Multiply resistant gram-positive bacteria: vancomycin-resistant enterococcus in solid organ transplant recipients | American journal of transplantation | 9(1), 50-56 | 10.1111/j.1600-6143.2009.02893.x | Review |
| Lima, elisangela da costa matos, guacira corrêa de vieira, jean m. De l. Gonçalves, ivana c. Da c. R | Suspected adverse drug reactions reported for brazilian children: cross-sectional study | Jornal de pediatria | 95(6), 682-688 | 10.1016/j.jped.2018.05.019 | Different domain |
| Flood, simon bodenham, andrew | Lithium: mimicry, mania, and muscle relaxants | Continuing education in anaesthesia critical care & pain | 10(3), 77-80 | 10.1093/bjaceaccp/mkq008 | Review |
| Lay, chii-shyan lin, jiun-rong | Correlation of cyp2c19 genetic polymorphisms with helicobacter pylori eradication in patients with cirrhosis and peptic ulcer | Journal of the chinese medical association | 73(4), 188-193 | 10.1016/s1726-4901(10)70039-3 | Different domain |
| Cunha, burke a. | Vancomycin | Medical clinics of north america | 79(4), 817-831 | 10.1016/s0025-7125(16)30041-4 | Review |
| Lee, ding-jen cosmatos, dennis marcial, victor a. Fu, karen k. Rotman, marvin | Results of an rtog phase iii trial (rtog 85-27) comparing radiotherapy plus etanidazole with radiotherapy alone for locally advanced head and neck carcinomas | International journal of radiation oncology*biology*physics | 32(3), 567-576 | 10.1016/0360-3016(95)00150-w | Different domain |
| Cruciol-souza, joice mara thomson, joão carlos | A pharmacoepidemiologic study of drug interactions in a brazilian teaching hospital | Clinics | 61(6), 515-520 | 10.1590/s1807-59322006000600005 | Different domain |
| Sadleir, p. H. M. Clarke, r. C. Platt, p. R. | Cefalotin as antimicrobial prophylaxis in patients with known intraoperative anaphylaxis to cefazolin | British journal of anaesthesia | 117(4), 464-469 | 10.1093/bja/aew274 | Different domain |
| Rivera-calimlim, leonor | The significance of drugs in breast milk: pharmacokinetic considerations | Clinics in perinatology | 14(1), 51-70 | 10.1016/s0095-5108(18)30781-4 | Review |
| Valdez, benigno c. Hassan, moustapha andersson, borje s. | Development of an assay for cellular efflux of pharmaceutically active agents and its relevance to understanding drug interactions | Experimental hematology | 52(1), 65-71 | 10.1016/j.exphem.2017.04.011 | Different domain |
| Muñoz-sánchez, deicy pinto, yimmi valencia-hernandez, juan david lora-suarez, fabiana | In vitro activity of ricinus communis (castor oil) on cysts of entamoeba histolytica | Phytomedicine plus | 4(2), 100-138 | 10.1016/j.phyplu.2024.100538 | Different domain |
| Trofe-clark, j.nlemonovich, t. L. | Interactions between anti-infective agents and immunosuppressants in solid organ transplantation | American journal of transplantation | 13(1), 318-326 | 10.1111/ajt.12123 | Different domain |
| Van leeuwen, r. W. F.,swart, e. L. Boven, e. Boom, f. A. | Potential drug interactions in cancer therapy: a prevalence study using an advanced screening method | Annals of oncology | 22(1), 2334-2341 | 10.1093/annonc/mdq761 | Review |
| Einecke, g. Miller, g. G. | Interactions between antiinfective agents and immunosuppressants | American journal of transplantation | 9(2), 263-266 | 10.1111/j.1600-6143.2009.02918.x | Review |
| Wittmann, dietmar h. Schein, moshe | Let us shorten antibiotic prophylaxis and therapy in surgery | The american journal of surgery | 172(6), 26-32 | 10.1016/s0002-9610(96)00347-9 | Review |
| Mombelli, andrea samaranayake, lakshman p. | Topical and systemic antibiotics in the management of periodontal diseases | International dental journal | 54(1), 3-14 | 10.1111/j.1875-595x.2004.tb00246.x | Review |
| Hersh, elliot v. | Adverse drug interactions in dental practice: interactions involving antibiotics: part ii of a series | The journal of the american dental association | 130(2), 236-251 | 10.14219/jada.archive.1999.0173 | Review |
| Malfertheiner, peter moss, steven f. Daniele, patrick pelletier, corey jacob, rinu | Potassium-competitive acid blocker and proton pump inhibitor–based regimens for first-line helicobacter pylori eradication: a network meta-analysis | Gastro hep advances | 1(5), 824-834 | 10.1016/j.gastha.2022.06.009 | Review |
| Zhang, bo xu, xiao-wei zeng, xue-jun li, da-kui | Correlation of thiopurine methyltransferase activity and 6-thioguanine nucleotide concentration in han chinese patients treated with azathioprine 25 to 100 mg: a 1-year, single-center, prospective study | Current therapeutic research | 67(4), 270-282 | 10.1016/j.curtheres.2006.07.002 | Different domain |
| Nakajima, shigemi inoue, hisayuki satake, hiroshi chatani, rena | Two-dimension tailor-made therapy: a new salvage therapy after multiple eradication failures for helicobacter pylori infection | Gastro hep advances | 1(2), 210-222 | 10.1016/j.gastha.2021.11.006 | Different domain |
| Noor, sidra ismail, mohammad haider, iqbal khadim, faiza | Drug-drug interactions in hepatitis patients: do these interactions matter in clinical perspectives? | Annals of hepatology | 17(6), 1001-1011 | 10.5604/01.3001.0012.7200 | Review |
| Abaza, himly el-zayadi, abdul rahman kabil, samir m. | Nitazoxanide in the treatment of patients with intestinal protozoan and helminthic infections: a report on 546 patients in egypt | Current therapeutic research | 59(2), 116-121 | 10.1016/s0011-393x(98)85006-6 | Abstract |
| Adelman, alan m. Daly, mel p. Michocki, robert j. | Alternate drugs | Clinics in geriatric medicine | 6(2), 423-444 | 10.1016/s0749-0690(18)30626-8 | Different domain |
| Akalin, h. E. | Clinical implications of aminopenicillins with β-lactamase inhibitors | International journal of antimicrobial agents | 7(2), 15-19 | 10.1016/0924-8579(96)00314-7 | Different domain |
| Aleem, samia wohlfarth, megan cotten, c. Michael greenberg, rachel g. | Infection control and other stewardship strategies in late onset sepsis, necrotizing enterocolitis, and localized infection in the neonatal intensive care unit | Seminars in perinatology | 44(8), 151-326 | 10.1016/j.semperi.2020.151326 | Abstract |
| Anderson, joe r. Nawarskas, james j. | Cardiovascular drug-drug interaction | Cardiology clinics | 19(2), 215-234 | /10.1016/s0733-8651(05)70209-5 | Review |
| Ansell, jack hirsh, jack poller, leon bussey, henry jacobson, alan | The pharmacology and management of the vitamin k antagonists: the seventh accp conference on antithrombotic and thrombolytic therapy | Chest | 126(3), 204-233 | 10.1378/chest.126.3_suppl.204s | Different domain |
| Appel, gerald b. Neu, harold c. | The use of drugs in renal failure | Disease-a-month | 25(11), 1-44 | 10.1016/s0011-5029(79)80002-4 | Review |
| Archer, johanna s. M. Archer, david f. | Oral contraceptive efficacy and antibiotic interaction: a myth debunked | Journal of the american academy of dermatology | 46(6) 917-923 | 10.1067/mjd.2002.120448 | Review |
| Arns, p. A. Branch, r. A. | Prescribing for patients with liver disease | Baillière's clinical gastroenterology | 3(1), 109-130 | 10.1016/0950-3528(89)90049-3 | Review |
| Baglin, t. | Management of warfarin (coumarin) overdose | Blood reviews | 12(2), 91-98 | 10.1016/s0268-960x(98)90020-0 | Review |
| Bailey, l. Charles reilly, anne f. Rheingold, susan r. | Infections in pediatric patients with hematologic malignancies | Seminars in hematology | 46(2), 313-324 | 10.1053/j.seminhematol.2009.03.010 | Review |
| Bakker, tinka abu-hanna, ameen dongelmans, dave a. Vermeijden, wytze j. Bosman, rob j. | Clinically relevant potential drug-drug interactions in intensive care patients: a large retrospective observational multicenter study | Journal of critical care | 62(1), 124-130 | 10.1016/j.jcrc.2020.11.020 | Different domain |
| Barat, lawrence m. Bloland, peter b. | Drug resistance among mmalaria and other parasite | Infectious disease clinics of north america | 11(4), 969-987 | 10.1016/s0891-5520(05)70400-1 | Review |
| Bassi, claudio | Infections in pancreatic inflammatory disease: clinical trials for antibiotic prophylaxis | Pancreatology | 1(3), 210-212 | 10.1159/000055812 | Review |
| Bassiri-jahromi, shahindokht iravani, kamyar | Fungal brain abscess: report of three cases and review of literature | Asian pacific journal of tropical disease | 4(1), 854-859 | 10.1016/s2222-1808(14)60745-3 | Review |
| Batista, lissette pérez jove, josefa rosinach, mercè gonzalo, victoria | Low efficacy of metronidazole in the eradication of blastocystis hominis in symptomatic patients: case series and systematic literature review | Gastroenterología y hepatología | 40(6), 381-387 | 10.1016/j.gastre.2016.11.012 | Review |
| Beeley, linda | Drugs and breast feeding | Clinics in obstetrics and gynaecology | 8(2), 291-295 | 10.1016/s0306-3356(21)00072-8 | Abstract |
| Pan, xiaolin li, yuqing qiu, yuping tang, qiyun | Efficacy and tolerability of first-line triple therapy with levofloxacin and amoxicillin plus esomeprazole or rabeprazole for the eradication of helicobacter pylori infection and the effect of cyp2c19 genotype: a 1-week, randomized, open-label study in chinese adults | Clinical therapeutics | 32(12), 2003-2011 | 10.1016/j.clinthera.2010.11.005 | Different domain |
| Navathe, reshama schoen, corina n. Heidari, paniz bachilova, sophia | Azithromycin vs erythromycin for the management of preterm premature rupture of membranes | American journal of obstetrics and gynecology | 221(2), 144-148 | 10.1016/j.ajog.2019.03.009 | Different domain |
| Naylor, gregory d. Fredericks, michael r. | Pharmacologic considerations in the dental management of the patient with disorders of the renal system | Dental clinics of north america | 40(3), 665-683 | 10.1016/s0011-8532(22)00130-6 | Abstract |
| Nelson, diana f. Schoenfeld, david weinstein, alan s. Nelson, james s. | A randomized comparison of misonidazole sensitized radiotherapy plus bcnu and radiotherapy plus bcnu for treatment of malignant glioma after surgery; preliminary results of an rtog study | International journal of radiation oncology*biology*physics | 9(8), 1143-1151 | 10.1016/0360-3016(83)90172-4 | Different domain |
| Neu, harold c. | Aztreonam activity, pharmacology, and clinical uses | The american journal of medicine | 88(3), 2-6 | 10.1016/0002-9343(90)90079-s | Review |
| Nichols, ronald lee | Surgical infections: prevention and treatment—1965 to 1995 | The american journal of surgery | 172(1), 68-74 | 10.1016/s0002-9610(96)00049-9 | Review |
| Nightingale, charles h. | Implications of fleroxacin's pharmacokinetic profile | International journal of antimicrobial agents | 4(1), 7-13 | 10.1016/0924-8579(94)90016-7 | Abstract |
| Norman coleman, c. | Hypoxic cell radiosensitizers: expectations and progress in drug development | International journal of radiation oncology*biology*physics | 11(2), 323-329 | 10.1016/0360-3016(85)90154-3 | Review |
| Novack, gary d. | Personalized medicine and the ocular surface | The ocular surface | 8(3), 157-160 | 10.1016/s1542-0124(12)70227-6 | Review |
| Okamoto, mark p. Gill, mark a. Nakahiro, randall k. Chin, alfred | Cost analysis of cefmetazole versus cefoxitin in the treatment of penetrating abdominal trauma | Current therapeutic research | 53(2), 159-166 | 10.1016/s0011-393x(05)80241-3 | Different domain |
| Okkan, sait yazici, zeliha uzel, reha akçasu, alaeddin | Use of ornidazole in fractionated radiotherapy: dose tolerance, serum and tumour tissue concentration | Radiotherapy and oncology | 5(4), 295-301 | 10.1016/s0167-8140(86)80178-5 | Review |
| Opie, lionel h. | Adverse cardiovascular drug interactions | Current problems in cardiology | 25(9), 621-676 | 10.1067/mcd.2000.109090 | Review |
| Oshikoya, k. A. Oreagba, i. A. Ogunleye, o. O. Hassan, m. | Use of complementary medicines among hiv-infected children in lagos, nigeria | Complementary therapies in clinical practice | 20(2), 118-124 | 10.1016/j.ctcp.2013.12.001 | Different domain |
| Overturf, gary d. | Antibiotic treatment of community acquired bacterial meningitis | Transactions of the royal society of tropical medicine and hygiene | 85(1), 9-16 | 10.1016/0035-9203(91)90333-t | Review |
| Owens, robert c. | Antimicrobial stewardship: concepts and strategies in the 21st century | Diagnostic microbiology and infectious disease | 61(1), 110-128 | 10.1016/j.diagmicrobio.2008.02.012 | Review |
| Pankey, george a. | Multicenter, phase iv evaluation of intravenous cirpfloxacin as initial therapy in patients with lower respiratory tract, urinary tract, and skin/skin structure infections | Clinical therapeutics | 17(3), 353-365 | 10.1016/0149-2918(95)80101-4 | Different domain |
| Xiao, yi li, xiaohan fu, xiaowei | A rapid and simple lc–ms/ms method for personalized busulfan dosing in pediatric patients undergoing hematopoietic stem cell transplantation (hsct) | Clinica chimica acta | 479(1), 190-195 | 10.1016/j.cca.2018.01.020 | Abstract |
| Woodson, cheryl e. Sachs, greg a. | Prevention, diagnosis, and management of infection in the nursing home | Clinics in geriatric medicine | 49(3), 507-525 | 10.1016/s0749-0690(18)30732-8 | Abstract |
| Wong-beringer, annie corelli, robin l. Schrock, theodore r. Joseph guglielmo, b. | Influence of timing of antibiotic administration on tissue concentrations during surgery | The american journal of surgery | 169(4), 379-381 | 10.1016/s0002-9610(99)80180-9 | Abstract |
| Witte, e. H. Peters, a. A. W. Smit, i. B. C. Van der linden, m. C. G. J. | A comparison of pefloxacin/metronidazole and doxycycline/metronidazole in the treatment of laparoscopically confirmed acute pelvic inflammatory disease | European journal of obstetrics & gynecology and reproductive biology | 50(2), 153-158 | 10.1016/0028-2243(93)90180-k | Abstract |
| Wieser, andreas li, hanwei zhang, jiang liss, ingrid markwardt, daniel | Evaluating the best empirical antibiotic therapy in patients with acute-on-chronic liver failure and spontaneous bacterial peritonitis | Digestive and liver disease | 51(9), 1300-1307 | 10.1016/j.dld.2019.02.015 | Abstract |
| Wibe, einar oftebro, reidar | A study of factors related to the action of 1-propargyl-5-chloropyrimidin-2-one (ny 3170) and vincristine in human multicellular spheroids | European journal of cancer and clinical oncology | 17(9), 1053-1059 | 10.1016/s0277-5379(81)80013-2 | Abstract |
| White, c. Michael | Thrombin-directed inhibitors: pharmacology and clinical use | American heart journal | 149(1), 54-60 | 10.1016/j.ahj.2004.10.023 | Review |
| Vlase, laurian neag, maria popa, adina muntean, dana leucuta, sorin e | Pharmacokinetic interaction between fluoxetine and omeprazole in healthy male volunteers: a prospective pilot study | Current therapeutic research | 71(6), 360-368 | 10.1016/s0011-393x(10)80002-5 | Different domain |
| Venetis, gregory chatzika, kalliopi pitsiou, georgia kechagias, nikolaos | Saliva and blood concentration of cefuroxime in patients undergoing maxillofacial surgery | Journal of oral and maxillofacial surgery | 70(6), 1398-1403 | 10.1016/j.joms.2012.01.007 | Different domain |
| Vardakas, konstantinos z. Mavros, michael n. | Meta-analysis of randomized controlled trials of vancomycin for the treatment of patients with gram-positive infections: focus on the study design | Mayo clinic proceedings | 87(4), 349-363 | 10.1016/j.mayocp.2011.12.011 | Abstract |
| Varanasi, ravikant v. Varanasi, sangeeta c. Howell, charles d. | Liver diseases | Clinics in geriatric medicine | 15(3), 559-570 | 10.1016/s0749-0690(18)30048-x | Review |
| Van der weiden, r. M. F. Van der meijden, w. I. Bogchelman, d. H. | Treatment failure in trichomoniasis and persistance of the parasite after lactobacillus immunotherapy; two case reports | European journal of obstetrics & gynecology and reproductive biology | 34(1), 171-178 | 10.1016/0028-2243(90)90021-r | Abstract |
| Vaira, d. Gatta, l. Ricci, c. D'anna, l. | Helicobacter pylori: diseases, tests and treatment | Digestive and liver disease | 33(9), 788-794 | 10.1016/s1590-8658(01)80697-6 | Review |
| Ulualp, kenan condon, robert e. | Antibiotic prophylaxsis for scedule operative procedures | Infectious disease clinics of north america | 6(3), 613-625 | 10.1016/s0891-5520(20)30465-7 | Abstract |
| Tsankov, nikolai broshtilova, valentina kazandjieva, jana | Tetracyclines in dermatology | Disease-a-month | 50(6), 332-344 | 10.1016/j.disamonth.2004.05.006 | Review |
| Trzepacz, paula t. Levenson, james l. Tringali, ronald a. | Psychopharmacology and neuropsychiatric syndromes in organ transplantation | General hospital psychiatry | 13(4), 233-245 | 10.1016/0163-8343(91)90124-f | Abstract |
| Trzepacz, paula t. Dimartini, andrea tringali, ronald | Psychopharmacologic issues in organ transplantation: part i: pharmacokinetics in organ failure and psychiatric aspects of immunosuppressants and anti-infectious agents | Psychosomatics | 34(3), 199-207 | 10.1016/s0033-3182(93)71881-2 | Abstract |
| Bellut, hugo arrayago, marine amara, marlène roujansky, ariane micaelo, maité | Real-life use of ceftobiprole for severe infections in a french intensive care unit | Infectious diseases now | 54(1), 104-109 | 10.1016/j.idnow.2023.104790 | Abstract |
| Berne, thomas v. Yellin, albert w. Appleman, maria d. | Antibiotic management of surgically treated gangrenous or perforated appendicitis: comparison of gentamicin and clindamycin versus cefamandole versus cefoperazone | The american journal of surgery | 144(1), 8-13 | 10.1016/0002-9610(82)90594-3 | Abstract |
| Berr, f. Kullak-ublick, g. A. Paumgartner, g. | 7 alpha-dehydroxylating bacteria enhance deoxycholic acid input and cholesterol saturation of bile in patients with gallstones | Gastroenterology | 111(6), 1611-1620 | 10.1016/s0016-5085(96)70024-0 | Review |
| Bianco, theresa m. | Drug interaction | Clinics in podiatric medicine and surgery | 9(2), 223-238 | 10.1016/s0891-8422(23)00516-5 | Abstract |
| Bonomo, robert a. | The new fluoroquinolone antibiotics | Clinical microbiology newsletter | 20(24), 197-201 | 10.1016/s0196-4399(00)88669-9 | Review |
| Bressler, rubin bahl, joseph j. | Principles of drug therapy for the elderly patient | Mayo clinic proceedings | 78(12), 1564-1577 | 10.4065/78.12.1564 | Review |
| Brockmann, william badr, mostafa | Chronic kidney disease: pharmacological considerations for the dentist | The journal of the american dental association | 141(11), 1330-1339 | 10.14219/jada.archive.2010.0077 | Abstract |
| Brook, itzhak | Anaerobic bacteria in upper respiratory tract and head and neck infections: microbiology and treatment | Anaerobe | 18(2), 214-220 | 10.1016/j.anaerobe.2011.12.014 | Review |
| Calva, juan | Antibiotic use in a periurban community in mexico: a household and drugstore survey | Social science & medicine | 42(8), 1121-1128 | 10.1016/0277-9536(95)00385-1 | Review |
| Cattaneo, dario zenoni, stefania murgia, stefano merlini, simona | Comparison of different cyclosporine immunoassays to monitor c0 and c2 blood levels from kidney transplant recipients: not simply overestimation | Clinica chimica acta | 355(1), 153-164 | 10.1016/j.cccn.2004.12.018 | Different domain |
| Nasser, sam | Prevention and treatment of sepsis in total hip replacement surgery | Orthopedic clinics of north america | 23(2), 265-277 | 10.1016/s0030-5898(20)31737-5 | Abstract |
| Navasa, m. Follo, a. Llovet, j. M. Clemente, g. | Randomized, comparative study of oral ofloxacin versus intravenous cefotaxime in spontaneous bacterial peritonitis | Gastroenterology | 111(4), 1011-1017 | 10.1016/s0016-5085(96)70069-0 | Abstract |
| Courtney, m. G. Nunes, d. P. Bergin, c. F. O'driscoll, m. | Randomised comparison of olsalazine and mesalazine in prevention of relapses in ulcerative colitis | The lancet | 339(8804), 1279-1281 | 10.1016/0140-6736(92)91601-4 | Different domain |
| Cummings, doyle m. Uttech, kay m. | Antibiotics for common infections in the elderly | Primary care: clinics in office practice | 17(4), 883-903 | 10.1016/s0095-4543(21)00906-4 | Abstract |
| Cunha, burke a. | Community-acquired pneumonia: diagnostic and therapeutic approach | Medical clinics of north america | 85(1), 43-77 | 10.1016/s0025-7125(05)70304-7 | Abstract |
| D. Gootz, thomas brighty, katherine e. Anderson, marge r. | In vitro activity of cp-99,219, a novel 7-(3-azabicyclo[3.1.0]hexyl) naphthyridone antimicrobial | Diagnostic microbiology and infectious disease | 19(4), 235-243 | 10.1016/0732-8893(94)90037-x | Abstract |
| Da̧browska-zamojcin, e. Pawlik, a. Domański, l. | Cyclosporine and sirolimus interaction in a kidney transplant patient | Transplantation proceedings | 37(5), 2317-2319 | 10.1016/j.transproceed.2005.03.094 | Different domain |
| Craig, william a. Andes, david r. | Parenteral versus oral antibiotic therapy | Medical clinics of north america | 79(3), 497-508 | 10.1016/s0025-7125(16)30052-9 | Review |
| Cranston, ross d. Anton, peter a. Mcgowan, ian m. | Gastrointestinal mucosal biopsy in hiv disease and aids | Gastrointestinal endoscopy clinics of north america | 10(4), 637-667 | 10.1016/s1052-5157(18)30102-8 | Review |
| Croft, nicholas m. Faubion, william a. Kugathasan, subra | Efficacy and safety of adalimumab in paediatric patients with moderate-to-severe ulcerative colitis (envision i): a randomised, controlled, phase 3 study | The lancet gastroenterology & hepatology | 6(8), 616-627 | 10.1016/s2468-1253(21)00142-4 | Abstract |
| Crowson, a. Neil magro, cynthia m. | The dermatopathology of drug eruptions | Current problems in dermatology | 14(4), 123-146 | /10.1016/s1040-0486(02)70014-8 | Abstract |
| Cheung, y. Whitney barco, stefano mathôt, ron a. A. | Pharmacokinetics of dabigatran etexilate and rivaroxaban in patients with short bowel syndrome requiring parenteral nutrition: the pder pan study | Thrombosis research | 160(1), 76-82 | /10.1016/j.thromres.2017.10.025 | Abstract |
| Chang, david c. Wilson, samuel e. | Meta-analysis of the clinical outcome of carbapenem monotherapy in the adjunctive treatment of intra-abdominal infection | The american journal of surgery | 174(30), 284-290 | 10.1016/s0002-9610(97)00137-2 | Abstract |
| Chanock, stephen j. Pizzo, philip a. | Fever in the neutropenic host | Infectious disease clinics of north america | 10(4), 777-796 | 10.1016/s0891-5520(05)70326-3 | Abstract |
| Chaplin, david j. | Keynote address: bioreductive therapy | International journal of radiation oncology*biology*physics | 22(4), 685-687 | 10.1016/0360-3016(92)90503-a | Review |
| Christian, stefanie s. Christian, jeffrey s. | The cephalosporin antibiotics | Primary care update for ob/gyns | 4(5), 168-174 | 10.1016/s1068-607x(97)81469-2 | Abstract |
| Clay, patrick g. Graham, maqual r. Lindsey, cameron c. | Clinical efficacy, tolerability, and cost savings associated with the use of open-label metronidazole plus ceftriaxone once daily compared with ticarcillin/clavulanate every 6 hours as empiric treatment for diabetic lower-extremity infections | The american journal of geriatric pharmacotherapy | 2(3), 181-189 | /10.1016/j.amjopharm.2004.09.006 | Abstract |
| Cline, matthew k. Bailey-dorton, chasse cayelli, maria | Maternal infection: diagnosis and management | Primary care: clinics in office practice | 27(1), 13-33 | 10.1016/s0095-4543(05)70146-9 | Abstract |
| Cohen, michael a. Huband, michael d. Mailloux, gail b. | In vitro antibacterial activities of the fluoroquinolones pd 117596, pd 124816, and pd 127391 | Diagnostic microbiology and infectious disease | 14(3), 245-258 | 10.1016/0732-8893(91)90039-i | Abstract |
| Coleman, c. Norman wasserman, todd h. Urtasun, raul c. | Final report of the phase i trial of the hypoxic cell radiosensitizer sr 2508 (etanidazole) radiation therapy oncology group 83-03 | International journal of radiation oncology*biology*physics | 18(2), 389-393 | 10.1016/0360-3016(90)90105-s | Abstract |
| Collura, jennifer m. Kraus, donna m. | New pediatric antiretroviral agents | Journal of pediatric health care | 14(4), 183-192 | 10.1067/mph.2000.107924 | Review |
| Finch, roger | Antimicrobial therapy: principles of use | Medicine | 33(3), 42-46 | 10.1383/medc.33.3.42.61118 | Abstract |
| Forstein, marshall | The neuropsychiatric aspects of hiv infection | Primary care: clinics in office practice | 19(1), 97-117 | 10.1016/s0095-4543(21)00123-8 | Abstract |
| Fragen, robert j. | Pharmacokinetics and pharmacodynamics of midazolam given via continuous intravenous infusion in intensive care units | Clinical therapeutics | 19(3), 405-419 | 10.1016/s0149-2918(97)80126-9 | Review |
| Futterman, donna chabon, brenda hoffman, neal d. | Hiv and aids in adolescents | Pediatric clinics of north america | 47(1), 171-188 | 10.1016/s0031-3955(05)70200-5 | Abstract |
| Sheridan, robert l. | Infections in critically ill pediatric burn patients | Seminars in pediatric infectious diseases | 11(1), 25-34 | 10.1053/spid.0110025 | Review |
| Shi, shaojun liu, yani li, zhongfang zheng, heng lv, yongning | Variability of carbamazepine and valproate concentrations in elderly nursing home residents | Clinical therapeutics | 32(10), 1832-1841 | 10.1016/j.clinthera.2010.09.015 | Abstract |
| Shields, andrea d. Plante, lauren a. Pacheco, luis d. Louis, judette m. | Society for maternal-fetal medicine consult series #67: maternal sepsis | American journal of obstetrics and gynecology | 229(3), 2-19 | 10.1016/j.ajog.2023.05.019 | Abstract |
| Singh, nishith narayan, s. | Nitazoxanide : a broad spectrum antimicrobial | Medical journal armed forces india | 67(1), 67-68 | 10.1016/s0377-1237(11)80020-1 | Review |
| Smith, don croser, david | Oral manifestations of hiv disease | Baillière's clinical gastroenterology | 4(2), 315-337 | 10.1016/0950-3528(90)90004-z | Abstract |
| Sobel, jack d. Faro, sabastian force, rex w. Foxman, bets | Vulvovaginal candidiasis: epidemiologic, diagnostic, and therapeutic considerations | American journal of obstetrics and gynecology | 178(2), 203-211 | 10.1016/s0002-9378(98)80001-x | Review |
| Sochalski, annette sullman, stephen andriole, vincent t. | Cost-effectiveness study of cefotetan versus cefoxitin and cefotetan versus combination antibiotic regimens | The american journal of surgery | 155(5), 96-101 | 10.1016/s0002-9610(88)80222-8 | Abstract |
| Solomkin, joseph s. | Use of new beta-lactam antibiotics for surgical infections | Surgical clinics of north america | 68(1), 1-24 | 10.1016/s0039-6109(16)44429-4 | Review |
| Spooner, d. Bugden, r. D. Peckham, m. J. Wist, e. W. | The combination of 5-fluorouracil with misonidazole in patients with advanced colorectal cancer | International journal of radiation oncology*biology*physics | 8(3), 387-389 | 10.1016/0360-3016(82)90645-9 | Review |
| Lim, cheryl li ling lee, winnie liew, yi xin tang, sarah si lin | Role of antibiotic prophylaxis in necrotizing pancreatitis: a meta-analysis | Journal of gastrointestinal surgery | 19(3), 480-491 | 10.1007/s11605-014-2662-6 | Abstract |
| Lim, li min singh, kuldip | Termination of pregnancy and unsafe abortion | Best practice & research clinical obstetrics & gynaecology | 28(6), 859-869 | 10.1016/j.bpobgyn.2014.05.005 | Review |
| Locksmith, gregory duff, patrick | Infection, antibiotics, and preterm delivery | Seminars in perinatology | 25(5), 295-309 | 10.1053/sper.2001.27163 | Abstract |
| Lodhi, t. Song, y. P. West, c. Hoskin, p. | Hypoxia and its modification in bladder cancer: current and future perspectives | Clinical oncology | 33(6), 376-390 | 10.1016/j.clon.2021.03.001 | Review |
| Lonsdale, dagan o. Baker, emma h. | Understanding and managing medication in elderly people | Best practice & research clinical obstetrics & gynaecology | 27(5), 767-788 | 10.1016/j.bpobgyn.2013.06.002 | Review |
| Goins, wendell a. Wiles, charles e. Cerra, frank b. | Pharmacology, monitoring, and nutritional support | Critical care clinics | 9(4), 689-713 | 10.1016/s0749-0704(18)30164-7 | Abstract |
| Goldherg, david m. | The cephalosporins | Medical clinics of north america | 71(6), 1113-1133 | 10.1016/s0025-7125(16)30800-8 | Review |
| Hersh, elliot v. Moore, paul a. | Drug interactions in dentistry: the importance of knowing your cyps | The journal of the american dental association | 135(3), 298-311 | 10.14219/jada.archive.2004.0178 | Abstract |
| Hessen, margaret trexler kaye, donald | Principles of selection and use of antibacterial agents | Infectious disease clinics of north america | 9(3), 531-545 | 10.1016/s0891-5520(20)30684-x | Abstract |
| Layton, alison thiboutot, diane | Emerging therapies in rosacea | Journal of the american academy of dermatology | 69(6), 57-65 | 10.1016/j.jaad.2013.04.041 | Abstract |
| Leibel, steven a. Scott, charles b. Pajak, thomas f. | The management of malignant gliomas with radiation therapy: therapeutic results and research strategies | Seminars in radiation oncology | 1(1) 32-49 | 10.1016/1053-4296(91)90007-t | Abstract |
| Levin, v. A. Maor, m. H. Thall, p. F. Yung, w. K. A. Bruner, j. | Phase ii study of accelerated fractionation radiation therapy with carboplatin followed by vincristine chemotherapy for the treatment of glioblastoma multiforme | International journal of radiation oncology*biology*physics | 33(2), 357-364 | 10.1016/0360-3016(95)00160-z | Different domain |
| Levin, victor a. Prados, michael r. Wara, william m. | Radiation therapy and bromodeoxyuridine chemotherapy followed by procarbazine, lomustine, and vincristine for the treatment of anaplastic gliomas | International journal of radiation oncology*biology*physics | 32(1), 75-83 | 10.1016/0360-3016(94)00488-7 | Abstract |
| Roach, albert c. | Antibiotic therapy in septic shock | Critical care nursing clinics of north america | 2(2), 179-186 | 10.1016/s0899-5885(18)30820-7 | Review |
| Romac, diane r. Albertson, timothy e. | Drug interactions in the intensive care unit | Clinics in chest medicine | 20(2), 385-399 | 10.1016/s0272-5231(05)70148-9 | Review |
| Ruderman, william b. Farmer, richard g. | Current management of inflammatory bowel disease | Radiologic clinics of north america | 25(1), 221-232 | 10.1016/s0033-8389(22)02226-6 | Review |
| Ellison, mark j. | Vancomycin, metronidazole, and tetracyclines | Clinics in podiatric medicine and surgery | 9(2), 425-442 | 10.1016/s0891-8422(23)00527-x | Abstract |
| Erskine, david | The use of drugs in patients with gastrointestinal manifestations of aids | Baillière's clinical gastroenterology | 4(2), 563-585 | 10.1016/0950-3528(90)90017-b | Review |
| Erttmann, m. Ullmann, u. Koch, e. M. W. | Results of a clinical and pharmacokinetic study of ceftazidime in patients with postoperative pneumonia on assisted ventilation | Journal of hospital infection | 15(1), 55-59 | 10.1016/0195-6701(90)90080-8 | Different domain |
| Faulx, michael d. Francis, gary s. | Adverse drug reactions in patients with cardiovascular disease | Current problems in cardiology | 33(12), 703-768 | 10.1016/j.cpcardiol.2008.08.002 | Abstract |
| Feagan, brian g. | Maintenance therapy for inflammatory bowel disease | The american journal of gastroenterology | 98(12), 6-17 | 10.1016/j.amjgastroenterol.2003.11.002 | Abstract |
| Hirsh, jack dalen, james e. Deykin, daniel poller, leon | Oral anticoagulants: mechanism of action, clinical effectiveness, and optimal therapeutic range | Chest | 108(4),231-246 | 10.1378/chest.108.4_supplement.231s | Abstract |
| Antimicrobial therapy in gynaecology | Current obstetrics & gynaecology | Current obstetrics & gynaecology | 4(2), 107-112 | 10.1016/0957-5847(94)90025-6 | Abstract |
| Huang, chenrong yang, jian du, yonghong miao, liyan | Measurement of free concentrations of highly protein-bound warfarin in plasma by ultra performance liquid chromatography–tandem mass spectrometry and its correlation with the international normalized ratio | Clinica chimica acta | 393(2), 85-89 | 10.1016/j.cca.2008.03.008 | Different domain |
| Hunt, richard h. | Eradication of helicobacter pylori infection | The american journal of medicine | 100(1), 42-51 | 10.1016/s0002-9343(96)80228-2 | Review |
| Hupp, james r. | Antibacterial, antiviral, and antifungal agents | Oral and maxillofacial surgery clinics of north america | 3(2), 273-285 | 10.1016/s1042-3699(20)30498-2 | Review |
| Hussar, daniel a. | New drugs: ramelteon, tipranavir, nepafenac, and deferasirox | Journal of the american pharmacists association | 46(1), 107-111 | 10.1331/154434506775268643 | Review |
| Ito, hiroaki takazoe, masakazu fukuda, yoshihiro hibi, toshifumi | A pilot randomized trial of a human anti-interleukin-6 receptor monoclonal antibody in active crohn’s disease | Gastroenterology | 126(4), 989-996 | 10.1053/j.gastro.2004.01.012 | Abstract |
| Jackson, r. K. Liew, l. P. Hay, m. P. | Overcoming radioresistance: small molecule radiosensitisers and hypoxia-activated prodrugs | Clinical oncology | 31(5), 290-302 | 10.1016/j.clon.2019.02.004 | Abstract |
| Joseph, warren s. Axler, david a. | Microbiology and antimicrobial therapy of diabetic foot infections | Clinics in podiatric medicine and surgery | 7(3), 467-481 | 10.1016/s0891-8422(23)00381-6 | Abstract |
| Joshi, j. V. Joshi, u. M. Sankholi, g. M. Krishna, u. Mandlekar, a. | A study of interaction of low-dose combination oral contraceptive with ampicillin and metronidazole | Contraception | 22(6), 643-652 | 10.1016/0010-7824(80)90089-x | Abstract |
| Jungnickel, paul w. | Pantoprazole: a new proton pump inhibitor | Clinical therapeutics | 22(11), 1268-1293 | 10.1016/s0149-2918(00)83025-8 | Review |
| Saklayen, mohammad c. | Capd peritonitis: incidence, pathogens, diagnosis, and management | Medical clinics of north america | 74(4), 997-1010 | 10.1016/s0025-7125(16)30532-6 | Review |
| Salinas, fabian pine, jeffrey r. | Approach to pneumonia in adults and the elderly: agents, diagnostic strategies and techniques, treatments, and problems | Immunology and allergy clinics of north america | 13(1), 171-192 | 10.1016/s0889-8561(22)00439-8 | Abstract |
| Saqr, abdelrahman carlson, brooke staley, christopher rashidi, armin al-kofahi, mahmoud kaiser, thomas | Reduced enterohepatic recirculation of mycophenolate and lower blood concentrations are associated with the stool bacterial microbiome after hematopoietic cell transplantation | Transplantation and cellular therapy | 28(7), 372-379 | 10.1016/j.jtct.2022.04.018 | Abstract |
| Sandborn, william j. Van os, erik c. Zins, bradley j. Tremaine, william j. | An intravenous loading dose of azathioprine decreases the time to response in patients with crohn's disease | Gastroenterology | 109(6), 1808-1817 | 10.1016/0016-5085(95)90747-5 | Abstract |
| Salman, huda s. Cynamon, jacov jagust, marci bakal, curtis | Randomized phase ii trial of embolization therapy versus chemoembolization therapy in previously treated patients with colorectal carcinoma metastatic to the liver | Clinical colorectal cancer | 2(3), 173-179 | 10.3816/ccc.2002.n.022 | Abstract |
| Sandborn, william j. Tremaine, william j. Schroeder, kenneth w. | A placebo-controlled trial of cyclosporine enemas for mildly to moderately active left-sided ulcerative colitis | Gastroenterology | 106(6), 1429-1435 | /10.1016/0016-5085(94)90394-8 | Abstract |
| Sandborn, william j. Korzenik, joshua lashner, bret | Once-daily dosing of delayed-release oral mesalamine (400-mg tablet) is as effective as twice-daily dosing for maintenance of remission of ulcerative colitis | Gastroenterology | 138(4), 1286-1296 | 10.1053/j.gastro.2009.12.054 | Different domain |
| Stewart, kenneth s. | bacterial infections | Clinics in obstetrics and gynaecology | 8(2), 315-332 | 10.1016/s0306-3356(21)00074-1 | Abstract |
| Stone, cosby a. Liu, yiwei relling, mary v. Krantz, matthew s. | Immediate hypersensitivity to polyethylene glycols and polysorbates: more common than we have recognized | The journal of allergy and clinical immunology: in practice | 7(5), 1533-1540 | /10.1016/j.jaip.2018.12.003 | Review |
| Kelly, h. William | Pharmacologic problems in the allergic patient with multiple medical problems | Immunology and allergy clinics of north america | 11(1), 17-29 | Https://doi.org/10.1016/s0889-8561(22)00303-4 | Abstract |
| K. Kenneth | Brain abscess | Medical clinics of north america | 69 (2), 345-360 | 10.1016/s0025-7125(16)31047-1 | Review |
| D. M. Kerins | Ampicillin/sulbactam—a combination of an old and a new agent in the treatment of infection | The american journal of the medical sciences | 301 (6), 406-411 | 10.1097/00000441-199106000-00011 | Abstract |
| D. Kertesz and a. W. Chow | Infected pressure and diabetic ulcers | Clinics in geriatric medicine | 8 (4), 835-852 | Https://doi.org/10.1016/s0749-0690(18)30448-8 | Abstract |
| S. R. Khan, u. Venugopal, g. Chandra, s. Bharti, r. K. Maurya and m. Y. Krishnan | Effect of various drugs on differentially detectable persisters of mycobacterium tuberculosis generated by long-term lipid diet | Tuberculosis | 115 (1), 89-95 | Https://doi.org/10.1016/j.tube.2019.02.007 | Abstract |
| S. Klein, t. Wadden and h. J. Sugerman | Aga technical review on obesity | Gastroenterology | 123(3), 882-932 | Https://doi.org/10.1053/gast.2002.35514 | Review |
| S. Kofler, c. Wolf, n. Shvets, z. Sisic, t. Müller, j. Behr, h.-y. Sohn, m. Vogeser, m. Shipkova, b. Meiser, g. Steinbeck, b. Reichart and i. Kaczmarek | The proton pump inhibitor pantoprazole and its interaction with enteric-coated mycophenolate sodium in transplant recipients | The journal of heart and lung transplantation | 30(5) , 565-571 | Https://doi.org/10.1016/j.healun.2010.12.003 | Review |
| K. Kongthavonsakul, a. Lucksiri, s. Eakanunkul, s. Roongjang, s. Issaranggoon na ayuthaya and p. Oberdorfer | Pharmacokinetics and pharmacodynamics of meropenem in children with severe infection | International journal of antimicrobial agents | 48(2), 151-157 | Https://doi.org/10.1016/j.ijantimicag.2016.04.025 | Abstract |
| P. I. Kowlessar, n. H. O. Connell, r. D. Mitchell, s. Elliott and t. S. J. Elliott | Management of patients with streptococcus milleri brain abscesses | Journal of infection | 52(6), 443-450 | Https://doi.org/10.1016/j.jinf.2005.08.028 | Abstract |
| A. K. Kreutner, v. E. Del bene, d. Delamar, j. L. Bodden and c. B. Loadholt | Perioperative cephalosporin prophylaxis in cesarean section: effect on endometritis in the high-risk patient | American journal of obstetrics and gynecology | 13(8), 925-935 | Https://doi.org/10.1016/0002-9378(79)90868-8 | Abstract |
| S. Kuriyama and c. Panosian | Antibiotics ii: aminoglycosides, polymyxins, vancomycin, trimethoprim-sulfamethoxazole, and pentamidine | Clinics in chest medicine | 7(3), 413-423 | Https://doi.org/10.1016/s0272-5231(21)01112-6 | Abstract |
| K. D. Laguardia, m. H. White, p. E. Saigo, s. Hoda, k. Mcguinness and w. J. Ledger | Genital ulcer disease in women infected with human immunodeficiency virus | American journal of obstetrics and gynecology | 172(2), 553-562 | Https://doi.org/10.1016/0002-9378(95)90572-3 | Review |
| E. Kiernan, j. E. Carpenter, c. A. Dunkley, d. Koch, b. W. Morgan, a. R. Steck and b. P. Murray | Elevated methemoglobin levels in a patient treated with hydroxocobalamin after suspected cyanide exposure | The journal of emergency medicine | 59(5), 157-162 | Https://doi.org/10.1016/j.jemermed.2020.07.008 | Different domain |
| T. J. Kinsella, a. Russo, j. B. Mitchell, j. Rowland, j. Jenkins, j. Schwade, c. E. Myers, j. M. Collins, j. Speyer, p. Kornblith, b. Smith, c. Kufta and e. Glatstein | A phase i study of intermittent intravenous bromodeoxyuridine (budr) with conventional fractionated irradiation | International journal of radiation oncology*biology*physics | 10(1), 69-76 | Https://doi.org/10.1016/0360-3016(84)90414-0 | Different domain |
| A. L. Klein and m. H. Sami | Usefulness and safety of cimetidine in patients receiving mexiletine for ventricular arrhythmia | American heart journal | 109(6), 1281-1286 | Https://doi.org/10.1016/0002-8703(85)90352-7 | Review |
| G. Matera, m. C. Berlinghieri and a. Focà | Meropenem: effects on human leukocyte functions and interleukin release | International journal of antimicrobial agents | 5(2), 129-133 | Https://doi.org/10.1016/0924-8579(94)00042-s | Review |
| G. R. Matzke | Nonrenal toxicities of acetaminophen, aspirin, and nonsteroidal anti-inflammatory agents | American journal of kidney diseases | 28(1),63-70 | Https://doi.org/10.1016/s0272-6386(96)90571-5 | Review |
| J. R. May, j. T. Dipiro and j. F. Sisley | Drug interactions in surgical patients | The american journal of surgery | 153(3), 327-335 | Https://doi.org/10.1016/0002-9610(87)90621-0 | Review |
| K. H. Mayer and j. A. Ellal | Lomefloxacin: microbiologic assessment and unique properties | The american journal of medicine | 92(4), s58-s62 | Https://doi.org/10.1016/0002-9343(92)90311-x | Abstract |
| D. Mayne and m. J. Dowzicky | In vitro activity of tigecycline and comparators against organisms associated with intra-abdominal infections collected as part of test (2004–2009) | Diagnostic microbiology and infectious disease | 74(2), 151-157 | Https://doi.org/10.1016/j.diagmicrobio.2012.05.032 | Different domain |
| J. D. Mccue | Antimicrobial therapy | Clinics in geriatric medicine | 8(4), 925-946 | Https://doi.org/10.1016/s0749-0690(18)30454-3 | Abstract |
| J. A. Mcgregor, j. I. French, k. Seo and d. Colorado | Adjunctive clindamycin therapy for preterm labor: results of a double-blind, placebo-controlled trial | American journal of obstetrics and gynecology | 165(4), 867-875 | Https://doi.org/10.1016/0002-9378(91)90430-y | Different domain |
| R. S. Mcleod, b. G. Wolff, a. Hillary steinhart, p. W. Carryer, k. O'rourke, d. F. Andrews, j. E. Blair, j. R. Cangemi, z. Cohen, j. B. Cullen, r. G. Chaytor, g. R. Greenberg, n. M. Jaffer, k. N. Jeejeebhoy, r. L. Maccarty, r. L. Ready and l. H. Weiland | Prophylactic mesalamine treatment decreases postoperative recurrence of crohn's disease | Gastroenterology | 109(2), 404-413 | Https://doi.org/10.1016/0016-5085(95)90327-5 | Different domain |
| I. Melamed, a. M. Griffiths and c. M. Roifman | Benefit of oral immune globulin therapy in patients with immunodeficiency and chronic diarrhea | The journal of pediatrics | 119(3), 486-489 | Https://doi.org/10.1016/s0022-3476(05)82070-0 | Abstract |
| B. P. Meloni, r. C. A. Thompson, j. A. Reynoldson and p. Seville | Albendazole: a more effective antigiardial agent in vitro than metronidazole or tinidazole | Transactions of the royal society of tropical medicine and hygiene | 84(3), 375-379 | Https://doi.org/10.1016/0035-9203(90)90324-8 | Different domain |
| B. M. Mercer and k. L. Arheart | Antibiotic therapy for preterm premature rupture of the membranes | Seminars in perinatology | 20(5), 426-438 | Https://doi.org/10.1016/s0146-0005(96)80010-3 | Review |
| S. L. Markantonis, g. Kostopanagiotou, d. Panidis, v. Smirniotis and d. Voros | Effects of blood loss and fluid volume replacement on serum and tissue gentamicin concentrations during colorectal surgery | Clinical therapeutics | 26(2), 271-281 | Https://doi.org/10.1016/s0149-2918(04)90025-2 | Different domain |
| M. G. Martens, s. Faro, m. Maccato, g. Riddle and h. A. Hammill | Susceptibility of female pelvic pathogens to oral antibiotic agents in patients who develop postpartum endometritis | American journal of obstetrics and gynecology | 164(5), 1383-1386 | Https://doi.org/10.1016/0002-9378(91)91477-e | Abstract |
| S. J. Martin, s. L. Pendland, c. Chen, p. C. Schreckenberger and l. H. Danziger | In vitro activity of clarithromycin alone and in combination with ciprofloxacin or levofloxacin against legionella spp.: enhanced effect by the addition of the metabolite 14-hydroxy clarithromycin | Diagnostic microbiology and infectious disease | 29(3), 167-171 | Https://doi.org/10.1016/s0732-8893(97)81806-8 | Abstract |
| L. Malincarne, m. Marroni, c. Farina, g. Camanni, m. Valente, b. Belfiori, s. Fiorucci, p. Floridi, a. Cardaccia and g. Stagni | Primary brain abscess with nocardia farcinica in an immunocompetent patient | Clinical neurology and neurosurgery | 104(2), 132-135 | Https://doi.org/10.1016/s0303-8467(01)00201-3 | Different domain |
| C. A. Marco and g. D. Kelen | Acute intoxication | Oral and maxillofacial surgery clinics of north america | 8(4), 731-748 | Https://doi.org/10.1016/s0733-8627(20)30234-0 | Review |
| C. L. Marek | Review of antidepressant medications: implications toward treatment | Emergency medicine clinics of north america | 11(4), 571-582 | Https://doi.org/10.1016/s1042-3699(20)30246-6 | Review |
| L. R. Peterson | A review of tigecycline — the first glycylcycline | International journal of antimicrobial agents | 32 (1), 215-222 | Https://doi.org/10.1016/s0924-8579(09)70005-6 | Review |
| L. R. Peterson, l. M. Lissack, k. Canter, c. E. Fasching, c. Clabots and d. N. Gerding | Therapy of lower extremity infections with ciprofloxacin in patients with diabetes mellitus, peripheral vascular disease, or both | The american journal of medicine | 86 (6), 801-808 | Https://doi.org/10.1016/0002-9343(89)90476-2 | Review |
| A. T. Pezzella, v. A. Ferraris and r. A. Lancey | Care of the adult cardiac surgery patient: part ii | Current problems in surgery | 41 (6), 526-574 | Https://doi.org/10.1016/j.cpsurg.2004.04.001 | Review |
| S. Picone, p. Manzoni, m. Bedetta, m. Mostert, d. K. Benjamin and p. Paolillo | Pharmacological resolution of a multiloculated candida spp. Liver abscess in a preterm neonate | Early human development | 89 (1), 47-50 | Https://doi.org/10.1016/s0378-3782(13)70015-x | Abstract |
| P. Piot, e. Van dyck, p. Godts and j. Vanderheyden | A placebo-controlled, double-blind comparison of tinidazole and triple sulfonamide cream for the treatment of nonspecific vaginitis | American journal of obstetrics and gynecology | 147 (1), 85-89 | Https://doi.org/10.1016/0002-9378(83)90090-x | Abstract |
| J. M. Piper, c. Baum and d. L. Kennedy | Prescription drug use before and during pregnancy in a medicaid population | American journal of obstetrics and gynecology | 157 (1), 148-156 | Https://doi.org/10.1016/s0002-9378(87)80368-x | Different domain |
| B. Piraino | Peritoneal infections | Advances in renal replacement therapy | 7 (4), 280-288 | Https://doi.org/10.1053/jarr.2000.18035 | Abstract |
| A. M. Powell and p. Nyirjesy | Recurrent vulvovaginitis | Best practice & research clinical obstetrics & gynaecology | 28 (7), 967-976 | Https://doi.org/10.1016/j.bpobgyn.2014.07.006 | Abstract |
| C. Premer and k. Caruso | Safety profile of the most ordered medications for breastfeeding patients in the emergency department | The american journal of emergency medicine | 80 (1), 1-7 | Https://doi.org/10.1016/j.ajem.2024.02.042 | Abstract |
| A. Qasim, j. Seery and c. A. O'morain | 5-aminosalicylates in inflammatory bowel: disease: choosing choosing the right dose | Digestive and liver disease | 33 (5), 393-398 | Https://doi.org/10.1016/s1590-8658(01)80008-6 | Review |
| S. Y. Quah, s. Wu, j. N. Lui, c. P. Sum and k. S. Tan | N-acetylcysteine inhibits growth and eradicates biofilm of enterococcus faecalis | Journal of endodontics | 38 (1), 81-85 | Https://doi.org/10.1016/j.joen.2011.10.004 | Review |
| R. U. Quenzer and d. R. P. Guay | Antimicrobial management strategies for patients with community-acquired respiratory tract infections | Current therapeutic research | 56 (5), 466-477 | Https://doi.org/10.1016/0011-393x(95)85079-1 | Review |
| R. Quintiliani | Cefixime: a pharmacoeconomic perspective | Current therapeutic research | 57 (12), 892-912 | Https://doi.org/10.1016/s0011-393x(96)80109-3 | Abstract |
| S. Racketa, k. Gandhi and m. Lambie | Meropenem versus piperacillin-tazobactam for the treatment of pancreatic necrosis | Diagnostic microbiology and infectious disease | 109 (2), 116-209 | Https://doi.org/10.1016/j.diagmicrobio.2024.116209 | Abstract |
| M.-u. Rashid, s. Rosenborg, g. Panagiotidis, k. S. Löfdal, a. Weintraub and c. E. Nord | Ecological effect of ceftazidime/avibactam on the normal human intestinal microbiota | International journal of antimicrobial agents | 46 (1), 60-65 | Https://doi.org/10.1016/j.ijantimicag.2015.02.027 | Different domain |
| M.-u. Rashid, a. Weintraub and c. E. Nord | Effect of new antimicrobial agents on the ecological balance of human microflora | Anaerobe | 18 (2), 249-253 | Https://doi.org/10.1016/j.anaerobe.2011.11.005 | Review |
| A. M. Ristuccia and b. A. Cunha | The aminoglycosides | Medical clinics of north america | 66 (1), 303-312 | Https://doi.org/10.1016/s0025-7125(16)31462-6 | Abstract |
| A. A. C. Satokata, j. H. Souza, l. L. O. Silva, m. B. Santiago, s. B. Ramos, l. R. D. Assis, r. D. S. Theodoro, l. R. E. Oliveira, l. O. Regasini and c. H. G. Martins | Chalcones with potential antibacterial and antibiofilm activities against periodontopathogenic bacteria | Anaerobe | 76 (1), 102588 | Https://doi.org/10.1016/j.anaerobe.2022.102588 | Abstract |
| H. J. Sauer | Physiology of lactation and factors affecting lactation | Obstetrics and gynecology clinics of north america | 14 (3), 615-622 | Https://doi.org/10.1016/s0889-8545(21)00081-4 | Abstract |
| H. Schelleman, w. B. Bilker, c. M. Brensinger, f. Wan, y.-x. Yang and s. Hennessy | Fibrate/statin initiation in warfarin users and gastrointestinal bleeding risk | The american journal of medicine | 123 (2), 151-157 | Https://doi.org/10.1016/j.amjmed.2009.07.020 | Review |
| D. Schkarpetkin, m. Reise, r. Wyrwa, a. Völpel, a. Berg, m. Schweder, m. Schnabelrauch, d. C. Watts and b. W. Sigusch | Development of novel electrospun dual-drug fiber mats loaded with a combination of ampicillin and metronidazole | Dental materials | 32 (8), 951-960 | Https://doi.org/10.1016/j.dental.2016.05.002 | Review |
| D. Schneider, r. Gannon, k. Sweeney and e. Shore | Theophylline and antiparasitic drug interactions: a case report and study of the influence of thiabendazole and mebendazole on theophylline pharmacokinetics in adults | Chest | 97 (1), 84-87 | Https://doi.org/10.1378/chest.97.1.84 | Abstract |
| P. Schuman, c. Christensen and j. D. Sobel | Aphthous vaginal ulceration in two women with acquired immunodeficiency syndrome | American journal of obstetrics and gynecology | 174 (5), 1660-1663 | Https://doi.org/10.1016/s0002-9378(96)70630-0 | Abstract |
| J. R. Schwebke and r. A. Desmond | Tinidazole vs metronidazole for the treatment of bacterial vaginosis | American journal of obstetrics and gynecology | 204 (3), 211-216 | Https://doi.org/10.1016/j.ajog.2010.10.898 | Different domain |
| J. R. Schwebke, f. G. Morgan, w. Koltun and p. Nyirjesy | A phase-3, double-blind, placebo-controlled study of the effectiveness and safety of single oral doses of secnidazole 2 g for the treatment of women with bacterial vaginosis | American journal of obstetrics and gynecology | 217 (6), 678-679 | Https://doi.org/10.1016/j.ajog.2017.08.017 | Different domain |
| J.-p. Sculier, a. Coune, f. Meunier, c. Brassinne, c. Laduron, c. Hollaert, n. Collette, c. Heymans and j. Klastersky | Pilot study of amphotericin b entrapped in sonicated liposomes in cancer patients with fungal infections | European journal of cancer and clinical oncology | 24 (3), 527-538 | Https://doi.org/10.1016/s0277-5379(98)90033-5 | Different domain |
| B. E. Scully and h. C. Neu | Use of aztreonam in the treatment of serious infections due to multiresistant gram-negative organisms, including pseudomonas aeruginosa | The american journal of medicine | 78 (2), 251-261 | Https://doi.org/10.1016/0002-9343(85)90435-8 | Abstract |
| D. A. Secco, i. T. Balassiano, r. F. Boente, k. R. Miranda, j. Brazier, v. Hall, j. D. Santos-filho, l. A. Lobo, s. A. Nouér and r. M. C. P. Domingues | Clostridium difficile infection among immunocompromised patients in rio de janeiro, brazil and detection of moxifloxacin resistance in a ribotype 014 strain | Anaerobe | 28 (1), 85-89 | Https://doi.org/10.1016/j.anaerobe.2014.05.013 | Different domain |
| J. H. Serna, a. Wanger and a. K. Dosekun | Successful treatment of mucormycosis peritonitis with liposomal amphotericin b in a patient on long-term peritoneal dialysis | American journal of kidney diseases | 42 (3), 131-134 | Https://doi.org/10.1016/s0272-6386(03)00797-2 | Review |
| L. E. Shapiro and n. H. Shear | Drug interactions/p450 | Current problems in dermatology | 13 (3), 141-152 | Https://doi.org/10.1016/s1040-0486(01)70007-5 | Abstract |
| W. A. Stack, s. D. Mann, a. J. Roy, p. Heath, m. Sopwith, j. Freeman, g. Holmes, r. Long, a. Forbes, m. A. Kamm and c. J. Hawkey | Randomised controlled trial of cdp571 antibody to tumour necrosis factor-α in crohn's disease | The lancet | 349 (9051), 521-524 | Https://doi.org/10.1016/s0140-6736(97)80083-9 | Abstract |
| H. Stass, d. Kubitza, b. Aydeniz, d. Wallwiener, a. Halabi and c. Gleiter | Penetration and accumulation of moxifloxacin in uterine tissue | International journal of gynecology & obstetrics | 102 (2), 132-136 | Https://doi.org/10.1016/j.ijgo.2008.02.020 | Abstract |
| R. W. Steele and g. L. Kearns | Antimicrobial therapy for pediatric patients | Pediatric clinics of north america | 36 (5), 1321-1349 | Https://doi.org/10.1016/s0031-3955(16)36770-0 | Abstract |
| D. C. Steffens and k. R. Rama krishnan | Metabolism, bioavailability, and drug interactions | Clinics in geriatric medicine | 14 (1), 17-32 | Https://doi.org/10.1016/s0749-0690(18)30128-9 | Abstract |
| G. E. Stein, s. Schooley, k. L. Tyrrell, d. M. Citron, d. P. Nicolau and e. J. C. Goldstein | Serum bactericidal activities of moxifloxacin and levofloxacin against aerobic and anaerobic intra-abdominal pathogens | Anaerobe | 14(1), 8-12 | Https://doi.org/10.1016/j.anaerobe.2007.09.005 | Abstract |
| K. Stein, j. Farmer, s. Singhal, f. Marra, s. Sutherland and c. Quiñonez | The use and misuse of antibiotics in dentistry: a scoping review | The journal of the american dental association | 149 (10), 869-884.e5 | Https://doi.org/10.1016/j.adaj.2018.05.034 | Abstract |
| C. Stewart | Prostatitis | Emergency medicine clinics of north america | 6 (3), 391-402 | Https://doi.org/10.1016/s0733-8627(20)30537-x | Abstract |
| C. W. Stratton, l. S. Weeks and k. E. Aldridge | Inhibitory and bactericidal activity of selected β-lactam agents alone and in combination with β-lactamase inhibitors compared with that of cefoxitin and metronidazole against cefoxitin-susceptible and cefoxitin-resistant isolates of the bacteroides fragilis group | Diagnostic microbiology and infectious disease | 15 (4), 321-330 | Https://doi.org/10.1016/0732-8893(92)90018-o | Abstract |
| R. M. Sutherland, p. Keng, p. J. Conroy, d. Mcdermott, b. J. Bareham and w. Passalacqua | In vitro hypoxic cytotoxicity of nitroimidazoles: uptake and cell cycle phase specificity | International journal of radiation oncology*biology*physics | 8 (3), 745-748 | Https://doi.org/10.1016/0360-3016(82)90726-x | Abstract |
| L. B. Svinhufvud, a. Heimdahl and c. E. Nord | Effect of topical administration of vancomycin versus chlorhexidine on α-hemolytic streptococci in oral cavity | Oral surgery, oral medicine, oral pathology | 66 (3), 304-309 | Https://doi.org/10.1016/0030-4220(88)90236-8 | Abstract |
| G. Sweeney, j. D. Watson, i. A. Mcgregor and j. Douglas sleigh | Successful prophylaxis with tinidazole of infection after major head and neck surgery for malignant disease | British journal of plastic surgery | 37 (1), 35-42 | Https://doi.org/10.1016/0007-1226(84)90038-9 | Abstract |
| R. L. Sweet | New approaches for the treatment of bacterial vaginosis | American journal of obstetrics and gynecology | 169 (2), 479-482 | Https://doi.org/10.1016/0002-9378(93)90346-k | Abstract |
| N. Tantranont, s. Hebert and l. D. Truong | Vancomycin nephrotoxicity causing renal transplant acute kidney injury | Transplantation proceedings | 53 (10), 2913-2917 | Https://doi.org/10.1016/j.transproceed.2021.09.033 | Abstract |
| G. Tebano, g. Li, b. Beovic, j. Bielicki, a. Brink, m. A. Enani, b. Godman, s. L. Hinrichsen, d. Kibuule, l.-h. Gabriel, o. Oduyebo, m. Sharland, s. Singh, h. F. L. Wertheim, d. Nathwani and c. Pulcini | Essential and forgotten antibiotics: an inventory in low- and middle-income countries | International journal of antimicrobial agents | 54 (3), 273-282 | Https://doi.org/10.1016/j.ijantimicag.2019.06.017 | Different domain |
| G. T. Terezhalmy and e. Achkar | Pharmacology of the gastrointestinal tract | Dental clinics of north america | 40 (3), 493-520 | Https://doi.org/10.1016/s0011-8532(22)00123-9 | Abstract |
| E. P. Tetangco and a. C. Stein | Medical treatment of intestinal crohn's disease | Seminars in colon and rectal surgery | 33 (1), 100-162 | Https://doi.org/10.1016/j.scrs.2022.100862 | Abstract |
| J. L. Thomason, l. M. Wilcoski and c. A. Mclaughlin | Trichomoniasis | Clinical microbiology newsletter | 8 (2), 9-12 | Https://doi.org/10.1016/0196-4399(86)90062-0 | Abstract |
| N. Tominaga, t. Edeki, j. Li, m. Learoyd, m. R. Bouw and s. Das | Phase i study assessing the safety, tolerability, and pharmacokinetics of avibactam and ceftazidime–avibactam in healthy japanese volunteers | Journal of infection and chemotherapy | 21 (8), 551-558 | Https://doi.org/10.1016/j.jiac.2015.04.006 | Different domain |
| L. Trevisani, s. Sartori, m. Caselli, m. Ruina, g. Verdianelli and v. Abbasciano | A four-day low dose triple therapy regimen for the treatment of helicobacter pylori infection | The american journal of gastroenterology | 93 (3), 390-393 | Https://doi.org/10.1016/s0002-9270(97)00111-1 | Different domain |
| G. Järnerot, h. Sandberg-gertzén and c. Tysk | Medical therapy of active crohn's disease | Baillière's clinical gastroenterology | 12 (1), 73-92 | Https://doi.org/10.1016/s0950-3528(98)90086-0 | Review |
| P. Jewesson, a. Chow, a. Wai, l. Frighetto, d. Nickoloff, j. Smith, l. Schwartz, k. Sleigh, d. Danforth, m. Pezim, j. Stoller and g. Stiver | A double-blind, randomized study of three antimicrobial regimens in the prevention of infections after elective colorectal surgery | Diagnostic microbiology and infectious disease | 29 (3), 155-165 | Https://doi.org/10.1016/s0732-8893(97)81805-6 | Different domain |
| O. H. Jhee, y. S. Lee, l. M. Shaw, y. C. Jeon, m. H. Lee, s. H. Lee and j. S. Kang | Pharmacokinetic and bioequivalence evaluation of two formulations of 100 mg trimebutine maleate (recutin™ and polybutin™) in healthy male volunteers using the lc–ms/ms method | Clinica chimica acta | 375 (1), 69-75 | Https://doi.org/10.1016/j.cca.2006.06.006 | Different domain |
| J. G. Johnson and t. C. Hardin | Aminoglycosides, imipenem, and aztreonam | Clinics in podiatric medicine and surgery | 9 (2), 443-464 | Https://doi.org/10.1016/s0891-8422(23)00528-1 | Abstract |
| L. Jokipii and a. M. M. Jokipii | Treatment of giardiasis: comparative evaluation of ornidazole and tinidazole as a single oral dose | Gastroenterology | 83 (2), 399-404 | Https://doi.org/10.1016/s0016-5085(82)80335-1 | Abstract |
| R. N. Jones | Cefotetan: a review of the microbiologic properties and antimicrobial spectrum | The american journal of surgery | 155 (5), 16-23 | Https://doi.org/10.1016/s0002-9610(88)80207-1 | Abstract |
| F. Gomollón, s. García-lópez, b. Sicilia, j. P. Gisbert and j. Hinojosa | The geteccu clinical guideline for the treatment of ulcerative colitis: a guideline created using grade methodology | Gastroenterología y hepatología | 36 (7), 481-486 | Https://doi.org/10.1016/j.gastrohep.2012.11.002 | Review |
| N. Grall, v. Lazarevic, n. Gaïa, c. Couffignal, c. Laouénan, e. Ilic-habensus, i. Wieder, p. Plesiat, c. Angebault, m. E. Bougnoux, l. Armand-lefevre, a. Andremont, x. Duval and j. Schrenzel | Unexpected persistence of extended-spectrum β-lactamase-producing enterobacteriaceae in the faecal microbiota of hospitalised patients treated with imipenem | International journal of antimicrobial agents | 50 (1), 81-87 | Https://doi.org/10.1016/j.ijantimicag.2017.02.018 | Review |
| S. F. M. Grimmer, d. J. Back, m. L. E. Orme, a. Cowie, i. Gilmore and j. Tjia | The bioavailability of ethinyloestradiol and levonorgestrel in patients with an ileostomy | Contraception | 33 (1), 51-59 | Https://doi.org/10.1016/0010-7824(86)90032-6 | Different domain |
| B. Guery, f. Menichetti, v.-j. Anttila, n. Adomakoh, j. M. Aguado, k. Bisnauthsing, a. Georgopali, s. D. Goldenberg, a. Karas, g. Kazeem, c. Longshaw, j. A. Palacios-fabrega, o. A. Cornely and m. J. G. T. Vehreschild | Extended-pulsed fidaxomicin versus vancomycin for clostridium difficile infection in patients 60 years and older (extend): a randomised, controlled, open-label, phase 3b/4 trial | The lancet infectious diseases | 18 (3), 296-307 | Https://doi.org/10.1016/s1473-3099(17)30751-x | Different domain |
| B. Guignard, p. Bonnabry, a. Perrier, p. Dayer, j. Desmeules and c. F. Samer | Drug-related problems identification in general internal medicine: the impact and role of the clinical pharmacist and pharmacologist | European journal of internal medicine | 26 (6), 399-406 | Https://doi.org/10.1016/j.ejim.2015.05.012 | Review |
| B. Guo, g. He, x. Wu, j. Yu, g. Cao, y. Li, y. Fan, y. Chen, y. Shi, y. Zhang and j. Zhang | Clinical pharmacokinetics of levornidazole in elderly subjects and dosing regimen evaluation using pharmacokinetic/pharmacodynamic analysis | Clinical therapeutics | 39 (7), 1336-1346 | Https://doi.org/10.1016/j.clinthera.2017.05.350 | Different domain |
| R. Guthrie | Community-acquired lower respiratory tract infections: etiology and treatment | Chest | 120 (6), 2021-2034 | Https://doi.org/10.1378/chest.120.6.2021 | Review |
| M. Hadjipavlou, m. Eragat, c. Kenny, m. Pantelidou, w. Mulhem, c. Wood, m. Dall’antonia and m. Y. Hammadeh | Effect of augmented antimicrobial prophylaxis and rectal swab culture–guided targeted prophylaxis on the risk of sepsis following transrectal prostate biopsy | European urology focus | 6 (1), 95-101 | Https://doi.org/10.1016/j.euf.2018.06.016 | Review |
| C. E. W. Halliday, p. M. G. Inge and m. J. G. Farthing | Characterization of bile salt uptake by giardia lamblia | International journal for parasitology | 25 (9), 1089-1097 | Https://doi.org/10.1016/0020-7519(95)00029-2 | Review |
| K. A. Hamod, m. R. Spence, n. B. Rosenshein and m. B. Dillon | Single-dose and multidose prophylaxis in vaginal hysterectomy: a comparison of sodium cephalothin and metronidazole | American journal of obstetrics and gynecology | 136 (1), 976-979 | Https://doi.org/10.1016/0002-9378(80)90620-1 | Review |
| S. B. Hanauer, w. J. Sandborn, p. Rutgeerts, r. N. Fedorak, m. Lukas, d. Macintosh, r. Panaccione, d. Wolf and p. Pollack | Human anti–tumor necrosis factor monoclonal antibody (adalimumab) in crohn’s disease: the classic-i trial | Gastroenterology | 130 (2), 323-333 | Https://doi.org/10.1053/j.gastro.2005.11.030 | Review |
| S. Harauchi, t. Osawa, n. Kubono, h. Itoh, t. Naito and j. Kawakami | Transfer of vaginal chloramphenicol to circulating blood in pregnant women and its relationship with their maternal background and neonatal health | Journal of infection and chemotherapy | 23 (7), 446-451 | Https://doi.org/10.1016/j.jiac.2017.03.015 | Review |
| E. Hassall | Peptic ulcer disease and current approaches to helicobacter pylori | The journal of pediatrics | 138 (4), 462-468 | Https://doi.org/10.1067/mpd.2001.113621 | Review |
| C. S. R. Hatton, c. Bunch, t. E. A. Peto, g. Pasvol, s. J. Russell, c. R. J. Singer, g. Edwards and p. Winstanley | Frequency of severe neutropenia associated with amodiaquine prophylaxis against malaria | The lancet | 327 (8478), 411-414 | Https://doi.org/10.1016/s0140-6736(86)92371-8 | Review |
| R. Heffron, r. S. Mcclelland, j. E. Balkus, c. Celum, c. R. Cohen, n. Mugo, e. Bukusi, d. Donnell, j. Lingappa, j. Kiarie, t. Fiedler, m. Munch, d. N. Fredricks, j. M. Baeten, c. Celum | Efficacy of oral pre-exposure prophylaxis (prep) for hiv among women with abnormal vaginal microbiota: a post-hoc analysis of the randomised, placebo-controlled partners prep study | The lancet hiv | 4 (10), 449-456 | Https://doi.org/10.1016/s2352-3018(17)30110-8 | Review |
| W. C. Hellinger and n. S. Brewer | Carbapenems and monobactams: imipenem, meropenem, and aztreonam | Mayo clinic proceedings | 74 (4), 420-434 | Https://doi.org/10.4065/74.4.420 | Review |
| S. E. Helms, d. L. Bredle, j. Zajic, d. Jatjoura, r. T. Brodell and i. Krishnarao | Oral contraceptive failure rates and oral antibiotics | Journal of the american academy of dermatology | 36 (5), 705-710 | Https://doi.org/10.1016/s0190-9622(97)80322-2 | Review |
| V. C. Hermes, a. P. Loureiro, m. P. Assis, f. Balbinot, i. Frighetto, h. Ziembowicz, r. M. Menezes and m. Carneiro | Pharmacoeconomic and antimicrobial stewardship analysis in waste management: beyond switching drug administration route | American journal of infection control | 51 (12), 1334-1338 | Https://doi.org/10.1016/j.ajic.2023.06.003 | Abstract |
| K. Herrlin, m. Segerdahl, l. L. Gustafsson and e. Kalso | Methadone, ciprofloxacin, and adverse drug reactions | The lancet | 356 (9247), 2069-2070 | Https://doi.org/10.1016/s0140-6736(00)03409-7 | Review |
| J. N. Galgiani, d. F. Busch, c. Brass, l. W. Rumans, j. I. Mangels and d. A. Stevens | Bacteroides fragilis endocarditis, bacteremia and other infections treated with oral or intravenous metronidazole | The american journal of medicine | 65(2), 284-289 | Https://doi.org/10.1016/0002-9343(78)90821-5 | Review |
| H. A. Gallis, r. O. Brennan, s. D. Goodwin, v. Swinney, m. M. Rumbaugh and r. H. Drew | Comparison of the safety and efficacy of intravenous ciprofloxacin and intravenous ceftazidime in the treatment of selected infections | The american journal of medicine | 87 (5), 176-180 | Https://doi.org/10.1016/0002-9343(89)90052-1 | Review |
| D. R. Gandara, p. N. Lara, z. Goldberg, q. T. Le, p. C. Mack, d. H. M. Lau and p. H. Gumerlock | Tirapazamine: prototype for a novel class of therapeutic agents targeting tumor hypoxia | Seminars in oncology | 29 (1), 102-109 | Https://doi.org/10.1053/sonc.2002.31531 | Review |
| V. F. Garagusi | Antimicrobial therapeutic drug monitoring | Clinics in laboratory medicine | 1 (3), 585-597 | Https://doi.org/10.1016/s0272-2712(18)31093-x | Review |
| J. Garau | The clinical potential of fourth-generation cephalosporins | Diagnostic microbiology and infectious disease | 31 (3), 479-480 | Https://doi.org/10.1016/s0732-8893(98)00035-2 | Review |
| L. B. Gasink, n. O. Fishman, m. G. Weiner, i. Nachamkin, w. B. Bilker and e. Lautenbach | Fluoroquinolone-resistant pseudomonas aeruginosa: assessment of risk factors and clinical impact | The american journal of medicine | 119 (60), 519-525 | Https://doi.org/10.1016/j.amjmed.2005.11.029 | Different domain |
| L. O. Gentry | Management of osteomyelitis | International journal of antimicrobial agents | 9 (1), 37-42 | Https://doi.org/10.1016/s0924-8579(97)00375-0 | Abstract |
| D. E. George and m. Glassman | Peptic ulcer disease in children | Gastrointestinal endoscopy clinics of north america | 4 (1), 23-37 | Https://doi.org/10.1016/s1052-5157(18)30519-1 | Abstract |
| B. Gerson and s. Subramaniam | Drug testing as part of the war on drugs | Clinics in laboratory medicine | 18 (4), 781-803 | Https://doi.org/10.1016/s0272-2712(18)30152-5 | Review |
| M. A. Ghazi suliman, k. Ogungbenro, c. Kosmidis, a. Ashworth, j. Barker, a. Szabo-barnes, a. Davies, l. Feddy, i. Fedor, t. Hayes, s. Stirling and i. Malagon | The effect of veno-venous ecmo on the pharmacokinetics of ritonavir, darunavir, tenofovir and lamivudine | Journal of critical care | 40 (1), 113-118 | Https://doi.org/10.1016/j.jcrc.2017.03.010 | Review |
| H. Giamarellou, j. Gazis, g. Petrikkos, a. Antsaklis, d. Aravantinos and g. K. Daikos | A study of cefoxitin, moxalactam, and ceftazidime kinetics in pregnancy | American journal of obstetrics and gynecology | 147 (8), 914-919 | Https://doi.org/10.1016/0002-9378(83)90245-4 | Abstract |
| S. Gibbins, p. Maddalena and l. Golec | Evidence-based care for the infant with necrotizing enterocolitis | Newborn and infant nursing reviews | 8 (3), 144-152 | Https://doi.org/10.1053/j.nainr.2008.06.009 | Review |
| G. L. Gilbert | Infectious diseases | Baillière's clinical obstetrics and gynaecology | 9 (9), 529-543 | Https://doi.org/10.1016/s0950-3552(05)80379-7 | Abstract |
| R. B. Giordani, m. V. De almeida, e. Fernandes, c. França da costa, g. A. De carli, t. Tasca and j. A. S. Zuanazzi | Anti-trichomonas vaginalis activity of synthetic lipophilic diamine and amino alcohol derivatives | Biomedicine & pharmacotherapy | 63 (80, 613-617 | Https://doi.org/10.1016/j.biopha.2008.10.002 | Review |
| A. Giudice, f. Bennardo, s. Barone, a. Antonelli, m. M. Figliuzzi and l. Fortunato | Can autofluorescence guide surgeons in the treatment of medication-related osteonecrosis of the jaw? A prospective feasibility study | Journal of oral and maxillofacial surgery | 76 (5), 982-995 | Https://doi.org/10.1016/j.joms.2017.10.024 | Different domain |
| V. M. Darda, e. Iosifidis, c. Antachopoulos, f. Kirvasilis, c. Zarras, a. B. Haidich, e. Papakonstantinou, a. Kontou, m. Sdougka and e. Roilides | Modifiable risk factors associated with later gut decolonization of carbapenem-resistant gram-negative bacteria in children: a prospective cohort study | Journal of hospital infection | 136(1), 75-84 | Https://doi.org/10.1016/j.jhin.2023.03.024 | Abstract |
| G. A. Davis and m. H. H. Chandler | Drug therapy and drug interactions | Oral and maxillofacial surgery clinics of north america | 8 (8), 245-263 | Https://doi.org/10.1016/s1042-3699(20)30897-9 | Review |
| M. Dershwitz | Is droperidol safe? Probably… | Seminars in anesthesia, perioperative medicine and pain | 23 (4), 291-301 | Https://doi.org/10.1053/j.sane.2004.01.007 | Abstract |
| K. Dettlaff, m. Stawny, a. Gostyńska, m. Popielarz-brzezińska and m. Ogrodowczyk | Compatibility of intravenous metronidazole with some all-in-one parenteral nutrition regimens | Nutrition | 84 (10), 111-120 | Https://doi.org/10.1016/j.nut.2020.111020 | Different domain |
| D. S. Dhami, g. Kunapuli, m. Das, d. Page and s. Natarajan | Drug‐drug interaction discovery: kernel learning from heterogeneous similarities | Smart health | 9-10 (1), 88-100 | Https://doi.org/10.1016/j.smhl.2018.07.007 | Review |
| T. S. Dharmarajan, s. Varma, s. Akkaladevi, a. S. Lebelt and e. P. Norkus | To anticoagulate or not to anticoagulate? A common dilemma for the provider: physicians’ opinion poll based on a case study of an older long-term care facility resident with dementia and atrial fibrillation | Journal of the american medical directors association | 7 (1), 23-28 | Https://doi.org/10.1016/j.jamda.2005.08.002 | Abstract |
| J. T. Dipiro and t. A. Bowden | A comparison of monobactam antibiotics in surgical infections | The american journal of surgery | 157 (6), 607-614 | Https://doi.org/10.1016/0002-9610(89)90712-5 | Review |
| M. Dojo, t. Azuma, t. Saito, m. Ohtani, a. Muramatsu and m. Kuriyama | Effects of cyp2c19 gene polymorphism on cure rates for helicobacter pylori infection by triple therapy with proton pump inhibitor (omeprazole or rabeprazole), amoxycillin and clarithromycin in japan | Digestive and liver disease | 33 (8), 671-675 | Https://doi.org/10.1016/s1590-8658(01)80043-8 | Different domain |
| M. Donaldson and r. Touger-decker | Vitamin and mineral supplements: friend or foe when combined with medications? | The journal of the american dental association | 145 (11), 1153-1158 | Https://doi.org/10.14219/jada.2014.78 | Abstract |
| J. P. Donnelly, r. E. Marcus, j. M. Goldman, j. Cohen, a. M. Worsley, d. Catovsky, j. H. Darrell, s. V. Want and d. A. G. Galton | Ceftazidime as first-line therapy for fever in acute leukaemia | Journal of infection | 11 (3), 205-215 | Https://doi.org/10.1016/s0163-4453(85)93063-4 | Review |
| G. R. Donowitz | Third generation cephalosporins | Infectious disease clinics of north america | 3 (3), 595-612 | Https://doi.org/10.1016/s0891-5520(20)30291-9 | Review |
| L. R. Douglas, j. B. Douglass, j. O. Sieck and p. J. Smith | Oral management of the patient with end-stage liver disease and the liver transplant patient | Oral surgery, oral medicine, oral pathology, oral radiology, and endodontology | 86 (1), 55-64 | Https://doi.org/10.1016/s1079-2104(98)90150-0 | Review |
| W. C. Dunagan and g. Medoff | Formulary control of antimicrobial usage: what price freedom? | Diagnostic microbiology and infectious disease | 16 (3), 265-274 | Https://doi.org/10.1016/0732-8893(93)90120-v | Abstract |
| T. Efferth, f. Herrmann, a. Tahrani and m. Wink | Cytotoxic activity of secondary metabolites derived from artemisia annua l. Towards cancer cells in comparison to its designated active constituent artemisinin | Phytomedicine | 18 (11), 959-969 | Https://doi.org/10.1016/j.phymed.2011.06.008 | Abstract |
| L. J. Egan, l. J. J. Derijks and d. W. Hommes | Pharmacogenomics in inflammatory bowel disease | Clinical gastroenterology and hepatology | 4 (1), 21-28 | Https://doi.org/10.1016/j.cgh.2005.10.003 | Review |
| A. K. Scott | Warfarin usage: can safety be improved? | Pharmacology & therapeutics | 42 (3) , 429-457 | Https://doi.org/10.1016/0163-7258(89)90034-x | Review |
| J. L. Robertson and r. P. Shrewsbury | Video teleconferencing in the compounding laboratory component of a dual-campus doctor of pharmacy program | American journal of pharmaceutical education | 75 (9) , 181-185 | Https://doi.org/10.5688/ajpe759181 | Review |
| H. K. Crewe, s. W. Ellis, m. S. Lennard and g. T. Tucker | Variable contribution of cytochromes p450 2d6, 2c9 and 3a4 to the 4-hydroxylation of tamoxifen by human liver microsomes | Biochemical pharmacology | 53 (2) , 171-178 | Https://doi.org/10.1016/s0006-2952(96)00650-8 | Different domain |
| A. K. Bhattiprolu, s. Kollipara, t. Ahmed, r. Boddu and s. Chachad | Utility of physiologically based biopharmaceutics modeling (pbbm) in regulatory perspective: application to supersede f2, enabling biowaivers & creation of dissolution safe space | Journal of pharmaceutical sciences | 111 (12), 3397-3410 | Https://doi.org/10.1016/j.xphs.2022.09.003 | Abstract |
| F. Xie, l. Liu, y. Wang, y. Peng and s. Li | An uplc-pda assay for simultaneous determination of seven antibiotics in human plasma | Journal of pharmaceutical and biomedical analysis | 210 (1), 114-158 | Https://doi.org/10.1016/j.jpba.2021.114558 | Abstract |
| S. Magréault, s. Leroux, j. Touati, t. Storme and e. Jacqz-aigrain | Uplc/ms/ms assay for the simultaneous determination of seven antibiotics in human serum–application to pediatric studies | Journal of pharmaceutical and biomedical analysis | 174 (1) , 256-262 | Https://doi.org/10.1016/j.jpba.2019.03.004 | Review |
| G. Carosi, g. Filice, f. Suter and a. Dei cas | Trichomonas vaginalis: effect of tinidazole on ultrastructure in vitro | Experimental parasitology | 43 (2) , 315-325 | Https://doi.org/10.1016/0014-4894(77)90036-4 | Review |
| J. Filimonovic, z. S. Ristić, t. Gazibara, v. Saponjic, j. Dotlic, v. Jovanovic, a. Arsovic, i. Vukajlovic, b. Joksimovic, d. Sokolovic, d. Drakul, d. Dimitrijevic, d. Plavsa and m. Milic | Trends and patterns of antibiotics use in serbia from 2006 to 2021: pre-covid-19 period versus covid-19 pandemic | American journal of infection control | 52 (3) , 293-304 | Https://doi.org/10.1016/j.ajic.2023.09.005 | Abstract |
| E. Touitou | Transdermal delivery of anxiolytics: in vitro skin permeation of midazolam maleate and diazepam | International journal of pharmaceutics | 33(1), 37-43 | Https://doi.org/10.1016/0378-5173(86)90036-0 | Different domain |
| M. A. C. Pérez, m. B. Sanz, l. R. Torres, r. G. Ávalos, m. P. González and h. G. Díaz | A topological sub-structural approach for predicting human intestinal absorption of drugs | European journal of medicinal chemistry | 39 (11) , 905-916 | Https://doi.org/10.1016/j.ejmech.2004.06.012 | Different domain |
| T. P. De araujo, i. M. Fittipaldi, d. C. G. Bedor, m. L. Duarte, s. F. Cordery, r. H. Guy, m. B. Delgado-charro, d. P. De santana and l. B. Leal | Topical bio(in)equivalence of metronidazole formulations in vivo | International journal of pharmaceutics | 541 (1), 167-172 | <https://doi.org/10.1016/j.ijpharm.2018.02.032> | Review |
| A. L. A. Gouveia, f. A. B. Santos, l. C. Alves, i. J. Cruz-filho, p. R. Silva, i. T. T. Jacob, j. C. S. Soares, d. K. D. N. Santos, t. R. C. L. Souza, j. F. Oliveira and m. D. C. A. Lima | Thiazolidine derivatives: in vitro toxicity assessment against promastigote and amastigote forms of leishmania infantum and ultrastructural study | Experimental parasitology | 236 (1) , 108- 253 | Https://doi.org/10.1016/j.exppara.2022.108253 | Abstract |
| F. Varum, h. Thorne, r. Bravo, d. Gilgen, c. Hartig, g. P. Nicolas, d. Wild, e. Liakoni and m. Haschke | Targeted colonic release formulations of mesalazine – a clinical pharmaco-scintigraphic proof-of-concept study in healthy subjects and patients with mildly active ulcerative colitis | International journal of pharmaceutics | 625 (1) , 122- 155 | Https://doi.org/10.1016/j.ijpharm.2022.122055 | Abstract |
| B. Negi, p. Poonan, m. F. Ansari, d. Kumar, s. Aggarwal, r. Singh, a. Azam and d. S. Rawat | Synthesis, antiamoebic activity and docking studies of metronidazole-triazole-styryl hybrids | European journal of medicinal chemistry | 150 (1) , 633-641 | Https://doi.org/10.1016/j.ejmech.2018.03.033 | Review |
| M. Shaban, s. Ghaffary, j. Hanaee, a. Karbakhshzadeh and s. Soltani | Synthesis and characterization of new surface modified magnetic nanoparticles and application for the extraction of letrozole from human plasma and analysis with hplc-fluorescence | Journal of pharmaceutical and biomedical analysis | 193 (1) , 113-159 | Https://doi.org/10.1016/j.jpba.2020.113659 | Different domain |
| S. Bereswill, t. Vey and m. Kist | Susceptibility in vitro of helicobacter pylori to cetylpyridinium chloride | Fems immunology and medical microbiology | 24 (2), 189-192 | Https://doi.org/10.1016/s0928-8244(99)00025-5 | Different domain |
| G. L. Southard and k. C. Godowski | Subgingival controlled release of antimicrobial agents in the treatment of periodontal disease | International journal of antimicrobial agents | 9 (4) , 239-253 | Https://doi.org/10.1016/s0924-8579(98)00004-1 | Review |
| Y. Wu and r. Fassihi | Stability of metronidazole, tetracycline hcl and famotidine alone and in combination | International journal of pharmaceutics | 290 (1) , 1-13 | Https://doi.org/10.1016/j.ijpharm.2004.10.015 | Different domain |
| X. Li and g. Xu | Simultaneous determination of ranitidine and metronidazole in pharmaceutical formulations at poly(chromotrope 2b) modified activated glassy carbon electrodes | Journal of food and drug analysis | 22 (3) , 345-349 | Https://doi.org/10.1016/j.jfda.2013.09.050 | Different domain |
| T. G. Do nascimento, e. De jesus oliveira and r. O. Macêdo | Simultaneous determination of ranitidine and metronidazole in human plasma using high performance liquid chromatography with diode array detection | Journal of pharmaceutical and biomedical analysis | 37 (4) , 777-783 | Https://doi.org/10.1016/j.jpba.2004.11.042 | Review |
| C. Sagan, a. Salvador, d. Dubreuil, p. P. Poulet, d. Duffaut and i. Brumpt | Simultaneous determination of metronidazole and spiramycin i in human plasma, saliva and gingival crevicular fluid by lc–ms/ms | Journal of pharmaceutical and biomedical analysis | 38 (2) , 298-306 | Https://doi.org/10.1016/j.jpba.2004.12.033 | Different domain |
| P. Wiczling, w. Struck-lewicka, ł. Kubik, d. Siluk, m. J. Markuszewski and r. Kaliszan | The simultaneous determination of hydrophobicity and dissociation constant by liquid chromatography–mass spectrometry | Journal of pharmaceutical and biomedical analysis | 94 (1) , 180-187 | Https://doi.org/10.1016/j.jpba.2014.01.038 | Different domain |
| P. O. Okonkwo and e. I. Eta | Simultaneous determination of chloroquine and metronidazole in human biological fluid by high pressure liquid chromatography | Life sciences | 42 (5) , 539-545 | Https://doi.org/10.1016/0024-3205(88)90095-1 | Different domain |
| P. K. F. Yeung, r. Little, y. Jiang, s. J. Buckley, p. T. Pollak, h. Kapoor and s. J. O. Veldhuyzen van zanten | A simple high performance liquid chromatography assay for simultaneous: determination of omeprazole and metronidazole in human plasma and gastric fluid1part of this material was presented at the 9th annual meeting and exposition of the aaps, san diego, ca, usa, november 6–10, 1994.1 | Journal of pharmaceutical and biomedical analysis | 17 (8) , 1393-1398 | Https://doi.org/10.1016/s0731-7085(98)00020-x | Abstract |
| I. Lavi and n. Gronich | Serum cholesterol increase in statin users associated with antibiotic use: case-crossover study | European journal of pharmacology | 932 (1) , 17-5209 | Https://doi.org/10.1016/j.ejphar.2022.175209 | Abstract |
| H. Kahma, m.-n. Paludetto, m. Neuvonen, m. Kurkela, a. M. Filppula, m. Niemi and j. T. Backman | Screening of 16 major drug glucuronides for time-dependent inhibition of nine drug-metabolizing cyp enzymes – detailed studies on cyp3a inhibitors | European journal of pharmaceutical sciences | 198 (1) , 106-735 | Https://doi.org/10.1016/j.ejps.2024.106735 | Abstract |
| X. Li, y. Liu, m. Wang, l. Gao, j. Liu, h. Zhang, m. Wu, h. Chen, j. Lou, j. Wang, j. Chen, g. Geng, z. Ma and y. Ding | Safety, pharmacokinetics, and efficacy of rifasutenizol, a novel dual-targeted antibacterial agent in healthy participants and patients in china with helicobacter pylori infection: four randomised clinical trials | The lancet infectious diseases | 24 (6) , 650-664 | Https://doi.org/10.1016/s1473-3099(24)00003-3 | Abstract |
| J. Cassell, i. Balakrishnan, d. Samarasinghe, p. Mistry, h. G. Prentice and s. H. Gillespie | Rp59500 (quinupristin/dalfopristin): three case reports of its use in infection due to enterococcus faecium | Journal of infection | 36 (3) , 324-327 | Https://doi.org/10.1016/s0163-4453(98)94495-4 | Abstract |
| R. Cazorla-luna, r. Ruiz-caro, m.-d. Veiga, r. K. Malcolm and d. A. Lamprou | Recent advances in electrospun nanofiber vaginal formulations for women's sexual and reproductive health | International journal of pharmaceutics | 607 (1) , 121-140 | Https://doi.org/10.1016/j.ijpharm.2021.121040 | Review |
| P. O. Gubbins, b. J. Gurley and j. Bowman | Rapid and sensitive high performance liquid chromatographic method for the determination of itraconazole and its hydroxy-metabolite in human serum1presented at the analysis and pharmaceutical quality section of the eleventh annual american association of pharmaceutical scientists meeting, october 1996, seattle, washington, usa.1 | Journal of pharmaceutical and biomedical analysis | 16 (6) , 1005-1012 | Https://doi.org/10.1016/s0731-7085(97)00062-9 | Abstract |
| P. J. Watts, c. G. Wilson, m. C. Davies and c. D. Melia | Radiolabelling of polymer microspheres for scintigraphic investigations by neutron activation. 2. Effects of irradiation on the properties of eudragit rs-sulphasalazine microspheres | International journal of pharmaceutics | 98 (1), 63-73 | Https://doi.org/10.1016/0378-5173(93)90042-e | Abstract |
| K. V. I. Rolston, g. P. Bodey and a. W. Chow | Prospective, double-blind, randomized trial of teicoplanin versus vancomycin for the therapy of vascular access-associated bacteremia caused by gram-positive pathogens | Journal of infection and chemotherapy | 5 (4) , 208-212 | Https://doi.org/10.1007/s101560050037 | Different domain |
| W. K. J. Huizinga, a. Hirshberg, s. R. Thomson, k. I. Elson, r. T. Salisbury and j. G. Brock-utne | Prophylactic parenteral cefuroxime: subcutaneous concentrations in laparotomy wounds | Journal of hospital infection | 13 (4) , 395-398 | Https://doi.org/10.1016/0195-6701(89)90059-5 | Different domain |
| M. Sharaf, m. Arif, h. I. Hamouda, s. Khan, m. Abdalla, s. Shabana, h. E. Rozan, t. U. Khan, z. Chi and c. Liu | Preparation, urease inhibition mechanisms, and anti-helicobacter pylori activities of hesperetin-7-rhamnoglucoside | Current research in microbial sciences | 3 (1) , 100-103 | Https://doi.org/10.1016/j.crmicr.2021.100103 | Abstract |
| O. A. Adeleke | Premium ethylcellulose polymer based architectures at work in drug delivery | International journal of pharmaceutics: x | 1 (1) , 100-123 | Https://doi.org/10.1016/j.ijpx.2019.100023 | Review |
| J. Fick, r. H. Lindberg, m. Tysklind and d. G. J. Larsson | Predicted critical environmental concentrations for 500 pharmaceuticals | Regulatory toxicology and pharmacology | 58 (3) , 516-523 | Https://doi.org/10.1016/j.yrtph.2010.08.025 | Different domain |
| Z.-c. Wang, y.-t. Duan, y.-j. Qin, p.-f. Wang, y. Luo, q. Wen, y.-a. Yang, j. Sun, y. Hu, y.-l. Sang and h.-l. Zhu | Potentiating 1-(2-hydroxypropyl)-2-styryl-5-nitroimidazole derivatives against antibacterial agents: design, synthesis and biology analysis | European journal of medicinal chemistry | 65 (1) , 456-463 | Https://doi.org/10.1016/j.ejmech.2013.05.004 | Review |
| M. Patberg, a. Isaak, f. Füsser, n. V. Ortiz zacarías, l. Vinnenberg, j. Schulte, l. Michetti, l. Grey, c. Van der horst, p. Hundehege, o. Koch, l. H. Heitman, t. Budde and a. Junker | Piperazine squaric acid diamides, a novel class of allosteric p2x7 receptor antagonists | European journal of medicinal chemistry | 226 (1) , 113- 138 | Https://doi.org/10.1016/j.ejmech.2021.113838 | Abstract |
| A. Srinivasan, j. C. Panetta, s. J. Cross, a. Pillai, b. M. Triplett, d. R. Shook, m. H. Dallas, c. Hartford, a. Sunkara, g. Kang, j. Jacobsen, j. Choi and w. Leung | Phase i study of the safety and pharmacokinetics of plerixafor in children undergoing a second allogeneic hematopoietic stem cell transplantation for relapsed or refractory leukemia | Biology of blood and marrow transplantation | 20 (8) , 1224-1228 | Https://doi.org/10.1016/j.bbmt.2014.04.020 | Different domain |
| T. Madden, m. De lima, n. Thapar, j. Nguyen, s. Roberson, d. Couriel, b. Pierre, e. J. Shpall, r. B. Jones, r. E. Champlin and b. S. Andersson | Pharmacokinetics of once-daily iv busulfan as part of pretransplantation preparative regimens: a comparison with an every 6-hour dosing schedule | Biology of blood and marrow transplantation | 13 (1) , 56-64 | Https://doi.org/10.1016/j.bbmt.2006.08.037 | Different domain |
| S. Shi, y. Liu, z. Li, h. Zheng, y. Lv and h. Chen | Pharmacokinetics and tolerability of intravenous cefotetan disodium for injection in healthy chinese volunteers: a randomized, open-label, single- and multiple-dose study | Clinical therapeutics | 32 (10) , 1832-1841 | Https://doi.org/10.1016/j.clinthera.2010.09.015 | Different domain |
| H. Wu, s. Xie, j. Yu, y. Chen, j. Wu, b. Guo, z. Zhu, y. Zhou, z. Wang and j. Zhang | Pharmacokinetics and pharmacodynamics of levornidazole in patients with intra-abdominal anaerobic infection | Clinical therapeutics | 40 (9) , 1548-1555 | Https://doi.org/10.1016/j.clinthera.2018.07.017 | Different domain |
| F. Börgel, f. Galla, k. Lehmkuhl, d. Schepmann, s. M. Ametamey and b. Wünsch | Pharmacokinetic properties of enantiomerically pure glun2b selective nmda receptor antagonists with 3-benzazepine scaffold | Journal of pharmaceutical and biomedical analysis | 172 (1) , 214-222 | Https://doi.org/10.1016/j.jpba.2019.04.032 | Review |
| Y. S. R. Krishnaiah, p. Veer raju, b. Dinesh kumar, v. Satyanarayana, r. S. Karthikeyan and p. Bhaskar | Pharmacokinetic evaluation of guar gum-based colon-targeted drug delivery systems of mebendazole in healthy volunteers | Journal of controlled release | 88 (1) , 95-103 | Https://doi.org/10.1016/s0168-3659(02)00483-2 | Different domain |
| J. B. Zimmermann, j. J. Horscht, m. A. Weigand, t. Bruckner, e. O. Martin, t. Hoppe-tichy and s. Swoboda | Patients enrolled in randomised clinical trials are not representative of critically ill patients in clinical practice: observational study focus on tigecycline | International journal of antimicrobial agents | 42 (5) , 436-442 | Https://doi.org/10.1016/j.ijantimicag.2013.07.016 | Different domain |
| D. Scotcher, c. Jones, a. Rostami-hodjegan and a. Galetin | Novel minimal physiologically-based model for the prediction of passive tubular reabsorption and renal excretion clearance | European journal of pharmaceutical sciences | 94 (1) , 59-71 | Https://doi.org/10.1016/j.ejps.2016.03.018 | Different domain |
| S.-f. Cui, l.-p. Peng, h.-z. Zhang, s. Rasheed, k. Vijaya kumar and c.-h. Zhou | Novel hybrids of metronidazole and quinolones: synthesis, bioactive evaluation, cytotoxicity, preliminary antimicrobial mechanism and effect of metal ions on their transportation by human serum albumin | European journal of medicinal chemistry | 86 (1) , 318-334 | Https://doi.org/10.1016/j.ejmech.2014.08.063 | Different domain |
| Y. Hu, c. Hu, g. Pan, c. Yu, m. F. Ansari, r. R. Yadav bheemanaboina, y. Cheng, c. Zhou and j. Zhang | Novel chalcone-conjugated, multi-flexible end-group coumarin thiazole hybrids as potential antibacterial repressors against methicillin-resistant staphylococcus aureus | European journal of medicinal chemistry | 222 (1) , 113- 128 | Https://doi.org/10.1016/j.ejmech.2021.113628 | Review |
| J. Pedron, c. Boudot, s. Hutter, s. Bourgeade-delmas, j.-l. Stigliani, a. Sournia-saquet, a. Moreau, e. Boutet-robinet, l. Paloque, e. Mothes, m. Laget, l. Vendier, g. Pratviel, s. Wyllie, a. Fairlamb, n. Azas, b. Courtioux, a. Valentin and p. Verhaeghe | Novel 8-nitroquinolin-2(1h)-ones as ntr-bioactivated antikinetoplastid molecules: synthesis, electrochemical and sar study | European journal of medicinal chemistry | 155 (1) , 135-152 | Https://doi.org/10.1016/j.ejmech.2018.06.001 | Review |
| H. Monney, j. Parrick and r. G. Wallace | Nitroimidazole radiosensitizers: approaches to their chemical synthesis | Pharmacology & therapeutics | 14 (2) , 197-216 | Https://doi.org/10.1016/0163-7258(81)90061-9 | Review |
| J. T. Gaensbauer, n. Dash, s. Verma, d. J. Hall, f. C. Adler-shohet, g. Li, g. Lee, l. Dinnes and k. Wendorf | Multidrug-resistant tuberculosis in children: a practical update on epidemiology, diagnosis, treatment and prevention | Journal of clinical tuberculosis and other mycobacterial diseases | 36 (1), 100- 149 | Https://doi.org/10.1016/j.jctube.2024.100449 | Abstract |
| W.-c. Tai, i. T. Wu, h.-m. Wang, p.-y. Huang, c.-c. Yao, c.-k. Wu, s.-c. Yang, c.-m. Liang, p.-i. Hsu and s.-k. Chuah | The multicenter real-world report of the efficacies of 14-day esomeprazole-based and rabeprazole-based high-dose dual therapy in first-line helicobacter pylori eradication in taiwan, | Journal of microbiology, immunology and infection | 4 (1), 23-29 | Https://doi.org/10.1016/j.jmii.2024.02.009 | Abstract |
| I. Calabrese, g. Cavallaro, c. Scialabba, m. Licciardi, m. Merli, l. Sciascia and m. L. Turco liveri | Montmorillonite nanodevices for the colon metronidazole delivery | International journal of pharmaceutics | 457 (1) , 224-236 | Https://doi.org/10.1016/j.ijpharm.2013.09.017 | Different domain |
| R. B. Sykes, d. P. Bonner and e. A. Swabb | Modern β-lactam antibiotics | Pharmacology & therapeutics | 29 (3) , 321-352 | Https://doi.org/10.1016/0163-7258(85)90007-5 | Abstract |
| C. D. Freeman, c. H. Nightingale and r. Quintiliani | Minocycline: old and new therapeutic uses | International journal of antimicrobial agents | 4 (4) , 325-335 | Https://doi.org/10.1016/0924-8579(94)90034-5 | Abstract |
| R. Beeck, g. Glöckl, j. Krause, p. Schick and w. Weitschies | Mimicking the dynamic colonic microbiota in vitro to gain a better understanding on the in vivo metabolism of xenobiotics: degradation of sulfasalazine | International journal of pharmaceutics | 603 (1), 120-174 | Https://doi.org/10.1016/j.ijpharm.2021.120704 | Review |
| D. Lopes-de-campos, c. Nunes, b. Sarmento, s. Jakobtorweihen and s. Reis | Metronidazole within phosphatidylcholine lipid membranes: new insights to improve the design of imidazole derivatives | European journal of pharmaceutics and biopharmaceutics | 129 (1) , 204-214 | Https://doi.org/10.1016/j.ejpb.2018.05.036 | Different domain |
| K. Ofer, d. Gold and e. Flescher | Methyl jasmonate induces cell cycle block and cell death in the amitochondriate parasite trichomonas vaginalis | International journal for parasitology | 38 (8) , 959-968 | Https://doi.org/10.1016/j.ijpara.2007.12.008 | Different domain |
| J. B. Perkins, j. Kim, c. Anasetti, h. F. Fernandez, l. E. Perez, e. Ayala, m. A. Kharfan-dabaja, m. R. Tomblyn, d. M. Sullivan, j. A. Pidala and t. L. Field | Maximally tolerated busulfan systemic exposure in combination with fludarabine as conditioning before allogeneic hematopoietic cell transplantation | Biology of blood and marrow transplantation | 18 (7) , 1099-1107 | Https://doi.org/10.1016/j.bbmt.2011.12.584 | Different domain |
| S. K. Baidya, s. Banerjee, s. Guti, t. Jha and n. Adhikari | Matrix metalloproteinase-8 (mmp-8) and its inhibitors: a minireview | European journal of medicinal chemistry reports | 10 (1) , 100-130 | Https://doi.org/10.1016/j.ejmcr.2024.100130 | Abstract |
| C. Larsen and m. Johansen | Macromolecular prodrugs iv. Kinetics of hydrolysis of metronidazole monosuccinate dextran ester conjugates in aqueous solution and in plasma — sequential release of metronidazole from the conjugates at physiological ph | International journal of pharmaceutics | 35 (1) , 39-45 | Https://doi.org/10.1016/0378-5173(87)90072-x | Abstract |
| E. J. Giamarellos-bourboulis, a. Spyridaki, a. Savva, m. Georgitsi, t. Tsaganos, m. Mouktaroudi, m. Raftogiannis, a. Antonopoulou, v. Papaziogas, f. Baziaka, k. Sereti, p. Christopoulos, a. Marioli, t. Kanni, p. Maravitsa, i. Pantelidou, k. Leventogiannis, p. Tsiaoussis, k. Lymberopoulou and i. M. Koutelidakis | Intravenous paracetamol as an antipyretic and analgesic medication: the significance of drug metabolism | Journal of pharmacological sciences | 124 (2), 144-152 | Https://doi.org/10.1254/jphs.13133fp | Different domain |
| T. Khawcharoenporn, a. Apisarnthanarak and l. M. Mundy | Intrathecal colistin for drug-resistant acinetobacter baumannii central nervous system infection: a case series and systematic review | Clinical microbiology and infection | 16 (7) , 888-894 | Https://doi.org/10.1111/j.1469-0691.2009.03019.x | Different domain |
| M. L. E. Orme, d. J. Back and s. Ball | Interindividual variation in the metabolism of ethynylestradiol | Pharmacology & therapeutics | 43 (2) , 251-260 | Https://doi.org/10.1016/0163-7258(89)90121-6 | Review |
| C. Tschanz, w. W. Stargel and j. A. Thomas | Interactions between drugs and nutrients | Academic press | 35, 1-26 | Https://doi.org/10.1016/s1054-3589(08)60273-2 | Different domain |
| P. E. Ciummo and n. L. Katz | Interactions and drug-metabolizing enzymes: by understanding the nature of drug-drug interactions, pharmacists can predict and avoid many adverse interactions | American pharmacy | 35 (9) , 41-53 | Https://doi.org/10.1016/s0160-3450(15)30073-8 | Abstract |
| X. Lu, t. Chan, l. Zhu, x. Bao, t. Velkov, q. T. Zhou, j. Li, h.-k. Chan and f. Zhou | The inhibitory effects of eighteen front-line antibiotics on the substrate uptake mediated by human organic anion/cation transporters, organic anion transporting polypeptides and oligopeptide transporters in in vitro models | European journal of pharmaceutical sciences | 115 (1) , 132-143 | Https://doi.org/10.1016/j.ejps.2018.01.002 | Different domain |
| L. Quintieri, p. Palatini, s. Moro and m. Floreani | Inhibition of cytochrome p450 2c8-mediated drug metabolism by the flavonoid diosmetin | Drug metabolism and pharmacokinetics | 26 (6) , 559-568 | Https://doi.org/10.2133/dmpk.dmpk-11-rg-048 | Different domain |
| M. S. Özaslan, n. Balcı, y. Demir, m. Gürbüz and ö. I. Küfrevioğlu | Inhibition effects of some antidepressant drugs on pentose phosphate pathway enzymes | Environmental toxicology and pharmacology | 72 (1) , 103-244 | Https://doi.org/10.1016/j.etap.2019.103244 | Review |
| Z.-z. Li, v. K. R. Tangadanchu, n. Battini, r. R. Y. Bheemanaboina, z.-l. Zang, s.-l. Zhang and c.-h. Zhou | Indole-nitroimidazole conjugates as efficient manipulators to decrease the genes expression of methicillin-resistant staphylococcus aureus | European journal of medicinal chemistry | 179 (1) , 723-735 | Https://doi.org/10.1016/j.ejmech.2019.06.093 | Review |
| P. A. Mccarron and m. Hall | Incorporation of novel 1-alkylcarbonyloxymethyl prodrugs of 5-fluorouracil into poly(lactide-co-glycolide) nanoparticles | International journal of pharmaceutics | 348 (1) , 115-124 | Https://doi.org/10.1016/j.ijpharm.2007.07.014 | Different domain |
| Y. S. R. Krishnaiah, v. Satyanarayana, b. Dinesh kumar, r. S. Karthikeyan and p. Bhaskar | In vivo pharmacokinetics in human volunteers: oral administered guar gum-based colon-targeted 5-fluorouracil tablets | European journal of pharmaceutical sciences | 19 (5) , 355-362 | Https://doi.org/10.1016/s0928-0987(03)00139-8 | Different domain |
| H. Vermeersch, j. P. Remon, d. Permentier and e. Schacht | In vitro antitrichomonal activity of water-soluble prodrug esters of metronidazole | International journal of pharmaceutics | 60 (3) , 253-260 | Https://doi.org/10.1016/0378-5173(90)90079-j | Different domain |
| S. Bhattacharya, d. Sen and c. Bhattacharjee | In vitro antibacterial effect analysis of stabilized pegylated allicin-containing extract from allium sativum in conjugation with other antibiotics | Process biochemistry | 87 (1) , 221-231 | Https://doi.org/10.1016/j.procbio.2019.09.025 | Review |
| F. Montiel, g. Kaltwasser, m. E. Pinto and m. Lam | In vitro antibacterial activity of trospectomycin (u-63,366f) against anaerobic bacteria and aerobic gram-positive cocci in chile | Diagnostic microbiology and infectious disease | 14 (3) , 259-264 | Https://doi.org/10.1016/0732-8893(91)90040-m | Different domain |
| M. A. Cohen, m. D. Huband, g. B. Mailloux, s. L. Yoder, g. E. Roland and c. L. Heifetz | In vitro antibacterial activities of the fluoroquinolones | Diagnostic microbiology and infectious disease | 14 (3) , 245-258 | Https://doi.org/10.1016/0732-8893(91)90039-i | Different domain |
| Y. Yang, l. Bian, x. Hang, c. Yan, y. Huang, f. Ye, g. Zhang, g. Jin and h. Bi | In vitro activity of new tetracycline analogues omadacycline and eravacycline against clinical isolates of helicobacter pylori collected in china | Diagnostic microbiology and infectious disease | 98 (3) , 115129 | Https://doi.org/10.1016/j.diagmicrobio.2020.115129 | Different domain |
| T. D. Gootz, k. E. Brighty, m. R. Anderson, b. J. Schmieder, s. L. Haskell, j. A. Sutcliffe, m. J. Castaldi and p. R. Mcguirk | In vitro activity of cp | Diagnostic microbiology and infectious disease | 19, 235-243 | Https://doi.org/10.1016/0732-8893(94)90037-x | Abstract |
| C. Dorn, a. Kratzer, u. Liebchen, m. Schleibinger, a. Murschhauser, j. Schlossmann, f. Kees, p. Simon and m. G. Kees | Impact of experimental variables on the protein binding of tigecycline in human plasma as determined by ultrafiltration | Journal of pharmaceutical sciences | 107 (2) , 739-744 | Https://doi.org/10.1016/j.xphs.2017.09.006 | Review |
| A. L. O’leary, a. K. Chan, b. A. Wattengel, j. Xu and k. A. Mergenhagen | Impact of doxycycline on clostridioides difficile infection in patients hospitalized with community-acquired pneumonia | American journal of infection control | 52 (3), 280-283 | Https://doi.org/10.1016/j.ajic.2023.09.007 | Abstract |
| G. Dubourg, j.-c. Lagier, f. Armougom, c. Robert, g. Audoly, l. Papazian and d. Raoult | High-level colonisation of the human gut by verrucomicrobia following broad-spectrum antibiotic treatment | International journal of antimicrobial agents | 41 (2), 149-155 | Https://doi.org/10.1016/j.ijantimicag.2012.10.012 | Different domain |
| Y. Nieto, p. Thall, b. Valdez, b. Andersson, u. Popat, p. Anderlini, e. J. Shpall, r. Bassett, a. Alousi, c. Hosing, p. Kebriaei, m. Qazilbash, e. Frazier, a. Gulbis, c. Chancoco, q. Bashir, s. Ciurea, i. Khouri, s. Parmar, n. Shah, l. Worth, g. Rondon, r. Champlin and r. B. Jones | High-dose infusional gemcitabine combined with busulfan and melphalan with autologous stem-cell transplantation in patients with refractory lymphoid malignancies | Biology of blood and marrow transplantation | 18 (11), 1677-1686 | Https://doi.org/10.1016/j.bbmt.2012.05.011 | Different domain |
| A. Capone, m. Giannella, d. Fortini, a. Giordano, m. Meledandri, m. Ballardini, m. Venditti, e. Bordi, d. Capozzi, m. P. Balice, a. Tarasi, g. Parisi, a. Lappa, a. Carattoli and n. Petrosillo | High rate of colistin resistance among patients with carbapenem-resistant klebsiella pneumoniae infection accounts for an excess of mortality | Clinical microbiology and infection | 19 (1) , 23-30 | Https://doi.org/10.1111/1469-0691.12070 | Review |
| J. Li, o. E. Olaleye, x. Yu, w. Jia, j. Yang, c. Lu, s. Liu, j. Yu, x. Duan, y. Wang, k. Dong, r. He, c. Cheng and c. Li | High degree of pharmacokinetic compatibility exists between the five-herb medicine xuebijing and antibiotics comedicated in sepsis care | Acta pharmaceutica sinica b | 9 (5) , 1035-1049 | Https://doi.org/10.1016/j.apsb.2019.06.003 | Different domain |
| M. Woodhead, f. Blasi, s. Ewig, j. Garau, g. Huchon, m. Ieven, a. Ortqvist, t. Schaberg, a. Torres, g. Van der heijden, r. Read and t. J. M. Verheij | Guidelines for the management of adult lower respiratory tract infections - full version | Clinical microbiology and infection | 17 (1) , 1-59 | Https://doi.org/10.1111/j.1469-0691.2011.03672.x | Different domain |
| R. E. Pounder | Gastrointestinal drugs | Elsevier | 21, 361-368 | Https://doi.org/10.1016/s0378-6080(98)80040-8 | Book |
| S. A. Ozkan and b. Uslu | From mercury to nanosensors: past, present and the future perspective of electrochemistry in pharmaceutical and biomedical analysis | Journal of pharmaceutical and biomedical analysis | 130 (1) , 126-140 | Https://doi.org/10.1016/j.jpba.2016.05.006 | Review |
| B. Das, v. S. Rawat, s. K. Ramasubbu and b. Kumar | Frequency, characteristics and nature of risk factors associated with use of qt interval prolonging medications and related drug-drug interactions in a cohort of psychiatry patients | Therapies | 74 (6) , 599-609 | Https://doi.org/10.1016/j.therap.2019.03.008 | Different domain |
| S. Rohaľová, t. Wolaschka, ľ. Balážová, k. Paulovičová, j. Tóthová, s. Pavloková, m. Stahorský and j. Gajdziok | Formulation optimization and evaluation of oromucosal in situ gel loaded with silver nanoparticles prepared by green biosynthesis | European journal of pharmaceutical sciences | 193 (1) , 106-183 | Https://doi.org/10.1016/j.ejps.2023.106683 | Abstract |
| E. Russo, f. Selmin, s. Baldassari, c. G. M. Gennari, g. Caviglioli, f. Cilurzo, p. Minghetti and b. Parodi | A focus on mucoadhesive polymers and their application in buccal dosage forms | Journal of drug delivery science and technology | 32 (1) , 113-125 | Https://doi.org/10.1016/j.jddst.2015.06.016 | Review |
| K. Brøsen, e. Skjelbo, b. B. Rasmussen, h. E. Poulsen and s. Loft | Fluvoxamine is a potent inhibitor of cytochrome p4501a2 | Biochemical pharmacology | 45 (6) , 1211-1214 | Https://doi.org/10.1016/0006-2952(93)90272-x | Different domain |
| L. Quintieri, p. Palatini, a. Nassi, p. Ruzza and m. Floreani | Flavonoids diosmetin and luteolin inhibit midazolam metabolism by human liver microsomes and recombinant cyp 3a4 and cyp3a5 enzymes | Biochemical pharmacology | 75 (6) , 1426-1437 | Https://doi.org/10.1016/j.bcp.2007.11.012 | Different domain |
| L. Quintieri, s. Bortolozzo, s. Stragliotto, s. Moro, m. Pavanetto, a. Nassi, p. Palatini and m. Floreani | Flavonoids diosmetin and hesperetin are potent inhibitors of cytochrome p450 2c9-mediated drug metabolism in vitro | Drug metabolism and pharmacokinetics | 25 (5) , 466-476 | Https://doi.org/10.2133/dmpk.dmpk-10-rg-044 | Review |
| S. R. Van helden, l. T. Schulz, m. Wimmer, v. L. Cancelliere and w. E. Rose | Finding value in novel antibiotics: how can infectious diseases adopt incremental cost-effectiveness to improve new antibiotic utilization? | Diagnostic microbiology and infectious disease | 109 (2), 116-245 | Https://doi.org/10.1016/j.diagmicrobio.2024.116245 | Abstract |
| K. Valko, s. Nunhuck, c. Bevan, m. H. Abraham and d. P. Reynolds | Fast gradient hplc method to determine compounds binding to human serum albumin. Relationships with octanol/water and immobilized artificial membrane lipophilicity | Journal of pharmaceutical sciences | 92 (11), 2236-2248 | Https://doi.org/10.1002/jps.10494 | Abstract |
| H. Majid, a. Puzik, t. Maier, d. Eberhard, a. Bartel, h.-c. Mueller and b. B. Burckhardt | Exploring the transmucosal permeability of cyclobenzaprine: a comparative preformulation by standardized and controlled ex vivo and in vitro permeation studies | International journal of pharmaceutics | 601(1), 12-174 | Https://doi.org/10.1016/j.ijpharm.2021.120574 | Review |
| J. Behra-miellet, l. Dubreuil and l. Calvet | Evaluation of the in vitro activity of ertapenem and nine other comparator agents against 337 anaerobic bacteria | International journal of antimicrobial agents | 28 (1) , 25-35 | Https://doi.org/10.1016/j.ijantimicag.2006.01.011 | Different domain |
| M. Pathak, a. G. A. Coombes, m. Jambhrunkar, d. Wang and k. J. Steadman | Evaluation of polycaprolactone matrices for sustained intravaginal delivery of a natural macromolecular microbicide, lactoferrin | Journal of drug delivery science and technology | 61 (1) , 101-191 | Https://doi.org/10.1016/j.jddst.2019.101191 | Review |
| S. B. Debast, m. P. Bauer and e. J. Kuijper | European society of clinical microbiology and infectious diseases: update of the treatment guidance document for clostridium difficile infection | Clinical microbiology and infection | 20 (1), 1-26 | Https://doi.org/10.1111/1469-0691.12418 | Review |
| A. Pascual, i. Garcia and e. J. Perea | Entry of lomefloxacin and temafloxacin into human neutrophils, peritoneal macrophages, and tissue culture cells | Diagnostic microbiology and infectious disease | 15 (5) , 393-398 | Https://doi.org/10.1016/0732-8893(92)90079-9 | Different domain |
| N. M. Idkaidek and n. M. Najib | Enhancement of oral absorption of metronidazole suspension in humans | European journal of pharmaceutics and biopharmaceutics | 50 (2) , 213-216 | Https://doi.org/10.1016/s0939-6411(00)00098-9 | Different domain |
| B. J. Aungst, n. J. Rogers and e. Shefter | Enhancement of naloxone penetration through human skin in vitro using fatty acids, fatty alcohols, surfactants, sulfoxides and amides | International journal of pharmaceutics | 33(1) , 225-234 | Https://doi.org/10.1016/0378-5173(86)90057-8 | Abstract |
| L. Bach hansen, a. Fullerton, l. L. Christrup and h. Bundgaard | Enhanced transdermal delivery of ketobemidone with prodrugs | 253-260 | 84 (3) , 253-260 | Https://doi.org/10.1016/0378-5173(92)90163-v | Different domain |
| C. Hidalgo-tenorio, s. Sadyrbaeva-dolgova, a. Enríquez-gómez, p. Muñoz, a. Plata-ciezar, j. M. Miró, a. Alarcón, f. J. Martínez-marcos, b. Loeches, f. Escrihuela-vidal, d. Vinuesa, c. Herrero, l. Boix-palop, m. Del mar arenas, e. G. Vázquez, f. A. De las revillas and j. Pasquau | En-dalbacen 2.0 cohort: real-life study of dalbavancin as sequential/consolidation therapy in patients with infective endocarditis due to gram-positive cocci | International journal of antimicrobial agents | 62 (3) , 106918 | Https://doi.org/10.1016/j.ijantimicag.2023.106918 | Abstract |
| J. Du, z. Ma, y. Zhang, t. Wang, x. Chen and d. Zhong | Enantioselective determination of ornidazole in human plasma by liquid chromatography–tandem mass spectrometry on a chiral-agp column | Journal of pharmaceutical and biomedical analysis | 86 (1) , 182-188 | Https://doi.org/10.1016/j.jpba.2013.07.048 | Different domain |
| A. Ortiz-covarrubias, e. Fang, p. G. Prokocimer, s. D. Flanagan, x. Zhu, j. F. Cabré-márquez, t. Tanaka, j. Passarell, j. Fiedler-kelly and e. C. Nannini | Efficacy, safety, tolerability and population pharmacokinetics of tedizolid, a novel antibiotic, in latino patients with acute bacterial skin and skin structure infections | The brazilian journal of infectious diseases | 20 (2) , 184-192 | Https://doi.org/10.1016/j.bjid.2015.12.007 | Different domain |
| X. Pan, y. Li, y. Qiu, q. Tang, b. Qian, l. Yao, r. Shi and g. Zhang | Efficacy and tolerability of first-line triple therapy with levofloxacin and amoxicillin plus esomeprazole or rabeprazole for the eradication of helicobacter pylori infection and the effect of cyp2c19 genotype: a 1-week, randomized, open-label study in chinese adults | Clinical therapeutics | 32 (12) , 2003-2011 | Https://doi.org/10.1016/j.clinthera.2010.11.005 | Different domain |
| G. Elizondo and p. Ostrosky-wegman | Effects of metronidazole and its metabolites on histamine immunosuppression activity | Life sciences | 59 (4), 285-297 | Https://doi.org/10.1016/0024-3205(96)00297-4 | Different domain |
| P. G. Welling | Effects of food on drug absorption | Pharmacology & therapeutics | 43 (3) , 425-441 | Https://doi.org/10.1016/0163-7258(89)90019-3 | Abstract |
| E. Myllyluoma, t. Ahlroos, l. Veijola, h. Rautelin, s. Tynkkynen and r. Korpela | Effects of anti-helicobacter pylori treatment and probiotic supplementation on intestinal microbiota | International journal of antimicrobial agents | 29 (1) , 66-72 | Https://doi.org/10.1016/j.ijantimicag.2006.08.034 | Different domain |
| C. E. Nord | The effect of antimicrobial agents on the ecology of the human intestinal microflora | Veterinary microbiology | 35 (3) , 193-197 | Https://doi.org/10.1016/0378-1135(93)90144-v | Review |
| C. W. Howden, g. G. Birnie and m. J. Brodie | Drug metabolism in liver disease | Pharmacology & therapeutics | 40 (3), 439-474 | Https://doi.org/10.1016/0163-7258(89)90088-0 | Review |
| P. Workman, p. R. Twentyman, f. Y. F. Lee and m. I. Walton | Drug metabolism and chemosensitization: nitroimidazoles as inhibitors of drug metabolism | Biochemical pharmacology | 32(3), 857-864 | Https://doi.org/10.1016/0006-2952(83)90588-9 | Abstract |
| P. Macheras, m. Koupparis and c. Tsaprounis | Drug dissolution studies in milk using the automated flow injection serial dynamic dialysis technique | International journal of pharmaceutics | 33 (1), 125-136 | Https://doi.org/10.1016/0378-5173(86)90046-3 | Review |
| J. Zini, j. Kekkonen, v. A. Kaikkonen, t. Laaksonen, p. Keränen, t. Talala, a. J. Mäkynen, m. Yliperttula and i. Nissinen | Drug diffusivities in nanofibrillar cellulose hydrogel by combined time-resolved raman and fluorescence spectroscopy | Journal of controlled release | 334 (1), 367-375 | Https://doi.org/10.1016/j.jconrel.2021.04.032 | Different domain |
| K. Ciura, s. Ulenberg, h. Kapica, p. Kawczak, m. Belka and t. Bączek | Drug affinity to human serum albumin prediction by retention of cetyltrimethylammonium bromide pseudostationary phase in micellar electrokinetic chromatography and chemically advanced template search descriptors | Journal of pharmaceutical and biomedical analysis | 188 (1) , 113-123 | Https://doi.org/10.1016/j.jpba.2020.113423 | Review |
| F.-f. Li, w.-h. Zhao, v. K. R. Tangadanchu, j.-p. Meng and c.-h. Zhou | Discovery of novel phenylhydrazone-based oxindole-thiolazoles as potent antibacterial agents toward pseudomonas aeruginosa | European journal of medicinal chemistry | 239 (1), 114-121 | Https://doi.org/10.1016/j.ejmech.2022.114521 | Abstract |
| Y.-g. Hu, n. Battini, b. Fang and c.-h. Zhou | Discovery of indolylacryloyl-derived oxacins as novel potential broad-spectrum antibacterial candidates | European journal of medicinal chemistry | 270 (1) , 116-192 | Https://doi.org/10.1016/j.ejmech.2024.116392 | Abstract |
| X. Yang, h. Sun, s. K. Maddili, s. Li, r.-g. Yang and c.-h. Zhou | Dihydropyrimidinone imidazoles as unique structural antibacterial agents for drug-resistant gram-negative pathogens | European journal of medicinal chemistry | 232 (1) , 114-188 | Https://doi.org/10.1016/j.ejmech.2022.114188 | Abstract |
| M. Murray, t. B. Gillani, s. Ghassabian, r. J. Edwards and t. Rawling | Differential effects of hepatic cirrhosis on the intrinsic clearances of sorafenib and imatinib by cyps in human liver | European journal of pharmaceutical sciences | 114 (1) , 55-63 | Https://doi.org/10.1016/j.ejps.2017.12.003 | Abstract |
| S. Kumar and r. Malviya | Dietary fibers and their derivatives for drug delivery applications: advances and prospective | Journal of drug delivery science and technology | 89 (1) , 105-184 | Https://doi.org/10.1016/j.jddst.2023.105084 | Abstract |
| D. L. Kirkapatrick | The development of hypoxic tumor cell cytotoxic agents | Pharmacology & therapeutics | 40 (3), 383-399 | Https://doi.org/10.1016/0163-7258(89)90086-7 | Abstract |
| Y. Yıldırım, i. Ince, b. Gümüştaş, ö. Vardar, n. Yakar, h. Munjaković, g. Özdemir and g. Emingil | Development of doxycycline and atorvastatin-loaded chitosan nanoparticles for local delivery in periodontal disease | Journal of drug delivery science and technology | 82 (1) , 104-122 | Https://doi.org/10.1016/j.jddst.2023.104322 | Abstract |
| T. S. Siddhartha, b. Prasanthi, t. Santosh and j. V. Ratna | Development and validation of high performance liquid chromatographic method for the determination of pyrazinamide in human plasma | Journal of pharmacy research | 7 (1) , 33-38 | Https://doi.org/10.1016/j.jopr.2013.01.023 | Different domain |
| S. Yakkundi, j. Millership, p. Collier, m. D. Shields and j. Mcelnay | Development and validation of a dried blood spot lc–ms/ms assay to quantify ranitidine in paediatric samples | Journal of pharmaceutical and biomedical analysis | 56(5), 1057-1063 | Https://doi.org/10.1016/j.jpba.2011.08.011 | Different domain |
| R. K. Agarwal, d. H. Robinson, g. I. Maze and r. A. Reinhardt | Development and characterization of tetracycline-poly(lactide/glycolide) films for the treatment of periodontitis | Development and characterization of tetracycline-poly(lactide/glycolide) films for the treatment of periodontitis | 23(2), 137-146 | Https://doi.org/10.1016/0168-3659(93)90039-8 | Different domain |
| J. A. Adamovics | Determination of antibiotics and antimicrobial agents in human serum by direct injection onto silica liquid chromatographic columns | Journal of pharmaceutical and biomedical analysis | 5(3), 267-274 | Https://doi.org/10.1016/0731-7085(87)80030-4 | Review |
| L.-l. Wang, n. Battini, r. R. Y. Bheemanaboina, s.-l. Zhang and c.-h. Zhou | Design and synthesis of aminothiazolyl norfloxacin analogues as potential antimicrobial agents and their biological evaluation | European journal of medicinal chemistry | 167 (1), 105-123 | Https://doi.org/10.1016/j.ejmech.2019.01.072 | Review |
| L. L. Christrup, s. S. Davis, m. Frier, c. D. Melia, s. N. Rasmussen, n. Washington, i. R. Wilding and c. Andersen | Deposition of a model substance, 99m tc e-hida, in the oral cavity after administration of lozenges, chewing gum and sublingual tablets | International journal of pharmaceutics | 66(1), 169-174 | Https://doi.org/10.1016/0378-5173(90)90396-l | Different domain |
| N. J. Medlicott, m. J. Rathbone, i. G. Tucker and d. W. Holborow | Delivery systems for the administration of drugs to the periodontal pocket | Advanced drug delivery reviews | 13 (1) , 181-203 | Https://doi.org/10.1016/0169-409x(94)90033-7 | Review |
| M. Vertzoni, a. Carlsson, b. Abrahamsson, k. Goumas and c. Reppas | Degradation kinetics of metronidazole and olsalazine by bacteria in ascending colon and in feces of healthy adults | International journal of pharmaceutics | 413(1), 81-86 | Https://doi.org/10.1016/j.ijpharm.2011.04.028 | Different domain |
| R. T. Mulcahy, j. J. Gipp, g. A. Ublacker, r. Panicucci and r. A. Mcclelland | Cytotoxicity and glutathione depletion by 1-methyl-2-nitrosoimidazole in human colon cancer cells | Cytotoxicity and glutathione depletion by 1-methyl-2-nitrosoimidazole in human colon cancer cells | 38(10), 1667-1671 | Https://doi.org/10.1016/0006-2952(89)90315-8 | Different domain |
| A. Stanley | Cytostatic drugs | Elsevier | 23, 476-493 | Https://doi.org/10.1016/s0378-6080(00)80051-3 | Abstract |
| O. Adeoye and h. Cabral-marques | Cyclodextrin nanosystems in oral drug delivery: a mini review | International journal of pharmaceutics | 531 (2) , 521-531 | Https://doi.org/10.1016/j.ijpharm.2017.04.050 | Review |
| J. C. Shah, y. Sadhale and d. M. Chilukuri | Cubic phase gels as drug delivery systems | Advanced drug delivery reviews | 47(2) , 229-250 | Https://doi.org/10.1016/s0169-409x(01)00108-9 | Review |
| S. Dharavath, r. Vijayan, k. Kumari, p. Tomar and s. Gourinath | Crystal structure of o-acetylserine sulfhydralase (oass) isoform 3 from entamoeba histolytica: pharmacophore-based virtual screening and validation of novel inhibitors | European journal of medicinal chemistry | 192 (1), 112-157 | Https://doi.org/10.1016/j.ejmech.2020.112157 | Different domain |
| F. Parsonson, a. Legg, m. Halford and k. Mccarthy | Contemporaneous management of ampicillin infusions in the outpatient setting through the use of therapeutic drug monitoring | International journal of antimicrobial agents | 55(6), 105-175 | Https://doi.org/10.1016/j.ijantimicag.2020.105975 | Different domain |
| M. Arlotti, p. Grossi, f. Pea, g. Tomei, v. Vullo, f. G. De rosa, g. Di perri, e. Nicastri, f. N. Lauria, g. Carosi, m. Moroni and g. Ippolito | Consensus document on controversial issues for the treatment of infections of the central nervous system: bacterial brain abscesses | International journal of infectious diseases | 14 (1) , 79-92 | Https://doi.org/10.1016/j.ijid.2010.05.010 | Review |
| K. Yamazaki and m. Kanaoka | Computational prediction of the plasma protein‐binding percent of diverse pharmaceutical compounds | Journal of pharmaceutical sciences | 93 (6) , 1480-1494 | Https://doi.org/10.1002/jps.20059 | Different domain |
| P. O. Okonkwo, c. O. Akpala, h. U. Okafor, a. U. Mbah and o. Nwaiwu | Compliance to correct dose of chloroquine in uncomplicated malaria correlates with improvement in the condition of rural nigerian children | Transactions of the royal society of tropical medicine and hygiene | 95 (3) , 320-324 | Https://doi.org/10.1016/s0035-9203(01)90252-4 | Different domain |
| M. Johansen and c. Larsen | A comparison of the chemical stability and the enzymatic hydrolysis of a series of aliphatic and aromatic ester derivatives of metronidazole | International journal of pharmaceutics | 26 (3) , 227-241 | Https://doi.org/10.1016/0378-5173(85)90232-7 | Review |
| J. H. Chia, y. Feng, l. H. Su, t. L. Wu, c. L. Chen, y. H. Liang and c. H. Chiu | Clostridium innocuum is a significant vancomycin-resistant pathogen for extraintestinal clostridial infection | Clinical microbiology and infection | 23 (8) , 560-566 | Https://doi.org/10.1016/j.cmi.2017.02.025 | Abstract |
| P. G. Clay, m. R. Graham, c. C. Lindsey, k. C. Lamp, c. Freeman and a. Glaros | Clinical efficacy, tolerability, and cost savings associated with the use of open-label metronidazole plus ceftriaxone once daily compared with ticarcillin/clavulanate every 6 hours as empiric treatment for diabetic lower-extremity infections in older males | The american journal of geriatric pharmacotherapy | 2 (3) , 181-189 | Https://doi.org/10.1016/j.amjopharm.2004.09.006 | Different domain |
| P. K. L. Chan, k. A. Skov, b. R. James and n. P. Farrell | Chromosome-damaging activity of a ruthenium radio-sensitizer, rucl2(dmso)2(4-nitroimidazole)2, in chinese hamster ovary cells in vitro | Chemico-biological interactions | 59 (1), 247-254 | Https://doi.org/10.1016/s0009-2797(86)80070-9 | Different domain |
| S. C. J. Jorgensen, s. Miljanic, n. Tabbara, d. Somanader, c. L. Y. Tse, c. De castro, i. Malhamé, s. E. Lapinsky and l. Burry | Characterizing the inclusion of pregnant and breastfeeding people in infectious diseases randomized controlled trials: a targeted literature review | Clinical microbiology and infection | 28 (6) , 801-811 | Https://doi.org/10.1016/j.cmi.2021.10.024 | Abstract |
| D. D. Chow and a. H. Karara | Characterization, dissolution and bioavailability in rats of ibuprofen-β-cyclodextrin complex system | International journal of pharmaceutics | 28(2) , 95-101 | Https://doi.org/10.1016/0378-5173(86)90232-2 | Different domain |
| J. D. Williams and g. A. Coles | Capd peritonitis | International journal of antimicrobial agents | 1 (4), 165-174 | Https://doi.org/10.1016/0924-8579(92)90003-a | Different domain |
| M. Ulrickson, j. Aldridge, h. T. Kim, e. P. Hochberg, p. Hammerman, c. Dube, e. Attar, k. K. Ballen, b. R. Dey, s. L. Mcafee, t. R. Spitzer and y.-b. Chen | Busulfan and cyclophosphamide (bu/cy) as a preparative regimen for autologous stem cell transplantation in patients with non-hodgkin lymphoma: a single-institution experience | Biology of blood and marrow transplantation | 15 (11) , 1447-1454 | Https://doi.org/10.1016/j.bbmt.2009.07.014 | Different domain |
| A. H. Mota, p. Rijo, j. Molpeceres and c. P. Reis | Broad overview of engineering of functional nanosystems for skin delivery | International journal of pharmaceutics | 532 (2) , 710-728 | Https://doi.org/10.1016/j.ijpharm.2017.07.078 | Review |
| G. M. Anlezark, r. G. Melton, r. F. Sherwood, w. R. Wilson, w. A. Denny, b. D. Palmer, r. J. Knox, f. Friedlos and a. Williams | Bioactivation of dinitrobenzamide mustards by an e. Coli b nitroreductase | Biochemical pharmacology | 50 (5) , 609-618 | Https://doi.org/10.1016/0006-2952(95)00187-5 | Abstract |
| I. Nir | Antiprotozoal drugs | Elsevier | 14, 237-246 | Https://doi.org/10.1016/s0378-6080(05)80094-7 | Book |
| A. Carvajal and l. H. Martín arias | Antipsychotic drugs | Elsevier | 24, 53-81 | Https://doi.org/10.1016/s0378-6080(01)80011-8 | Book |
| I. Nir | Antiprotozoal drugs | Elsevier | 13, 238-245 | Https://doi.org/10.1016/s0378-6080(89)80034-0 | Book |
| I. Nir | Antiprotozoal drugs | Elsevier | 12, 239-247 | Https://doi.org/10.1016/s0378-6080(88)80094-1 | Book |
| S. Berdaguer, j. Bautista, m. Brunet and j. M. Cisneros | Antimicrobial and immunosuppressive drug interactions in solid organ transplant recipients | Enfermedades infecciosas y microbiología clínica | 30 (1) , 86-92 | Https://doi.org/10.1016/s0213-005x(12)70087-3 | Book |
| B. Negi, d. Kumar, w. Kumbukgolla, s. Jayaweera, p. Ponnan, r. Singh, s. Agarwal and d. S. Rawat | Anti-methicillin resistant staphylococcus aureus activity, synergism with oxacillin and molecular docking studies of metronidazole-triazole hybrids | European journal of medicinal chemistry | 115 (11) , 426-437 | Https://doi.org/10.1016/j.ejmech.2016.03.041 | Abstract |
| E. Perucca | Anticonvulsant drugs | Elsevier | 19, 61-81 | Https://doi.org/10.1016/s0378-6080(05)80132-1 | Book |
| V. K. Lepakhin and a. V. Astakhova | Antifungal drugs | Elsevier | 11, 240-247 | Https://doi.org/10.1016/s0378-6080(87)80033-8 | Book |
| D.-j. Choi, w.-k. Cho and m.-k. Kim | An analysis of the interactions between warfarin and korean traditional herbal medicine in a korean oriental hospital | European journal of integrative medicine | 6 (4) , 502-512 | Https://doi.org/10.1016/j.eujim.2013.11.002 | Abstract |
| G. A. B. Davies-jones | Anticonvulsant drugs | Elsevier | 9, 55-62 | Https://doi.org/10.1016/s0378-6080(85)80011-8 | Book |
| E. Jensen and h. Bundgaard | Aminomethylbenzoate esters of chloramphenicol as a novel prodrug type for parenteral administration | International journal of pharmaceutics | 70 (1) , 137-146 | Https://doi.org/10.1016/0378-5173(91)90173-l | Review |
| A. De boer, f. Van hunsel and a. Bast | Adverse food–drug interactions | Regulatory toxicology and pharmacology | 73 (30) , 859-865 | Https://doi.org/10.1016/j.yrtph.2015.10.009 | Abstract |
| E. V. Hersh, a. Pinto and p. A. Moore | Adverse drug interactions involving common prescription and over-the-counter analgesic agents | Clinical therapeutics | 29 (11) , 2477-2497 | Https://doi.org/10.1016/j.clinthera.2007.12.003 | Abstract |
| R. Larcher, c. Maury, g. Faivre, g. Dagod, y. Dumont, v. Le moing, m. Villiet, x. Capdevila and j. Charbit | Acquisition of extended-spectrum cephalosporin-resistant gram-negative bacteria: epidemiology and risk factors in a 6-year cohort of 507 severe trauma patients | Journal of global antimicrobial resistance | 31 (1) , 363-370 | Https://doi.org/10.1016/j.jgar.2022.10.005 | Abstract |
| F. P. Bonina, l. Montenegro, p. De capraris, e. Bousquet and s. Tirendi | Alkylazacycloalkan-2-one esters as prodrugs of indomethacin for improved delivery through human skin | International journal of pharmaceutics | 77 (1) , 21-29 | <https://doi.org/10.1016/0378-5173(91)90297-2> | Different domain |
| A. Abdulkadir | Interactions of chloroquine and/or ethanol on renal morphology of normal or low protein fed male sprague dawley rats | University of the witwatersrand, faculty of health sciences, |  |  | Thesis |
| A. Aboudalle, f. Fourcade, a. A. Assadi, l. Domergue, h. Djelal, t. Lendormi, s. Taha and a. Amrane | Reactive oxygen and iron species monitoring to investigate the electro-fenton performances. Impact of the electrochemical process on the biodegradability of metronidazole and its by-products | Chemosphere | 199 (1), 486-494 |  | Not accessible |
| B. P. Abraham, t. Ahmed and t. Ali | Inflammatory bowel disease: pathophysiology and current therapeutic approaches | Gastrointestinal pharmacology | 2017(1), 115-146 | <https://doi.org/10.1007/164_2016_122> | Review |
| Lew, daniel p waldvogel, francis a | Osteomyelitis | The lancet | 364 (9431), 369-379 | [/doi.org/10.1016/s0140-6736(04)16727-5](https://doi.org/10.1016/S0140-6736(04)16727-5) | Review |
| I. Adamsson, c. Edlund and c. Nord | Microbial ecology and treatment of helicobacter pylori infections | Journal of chemotherapy | 12 (1) , 5-16 | Https://doi.org/10.1111/j.1523-5378.2010.00774.x | Review |
| A. O. Adebisi and b. R. Conway | Modification of drug delivery to improve antibiotic targeting to the stomach | Therapeutic delivery | 6 (6) , 741-762 | Https://doi.org/10.4155/tde.15.35 | Review |
| B. Adzu, c. Masimirembwa, k. B. Mustapha, r. Thelingwani, r. A. Kirim and k. S. Gamaniel | Effect of niprisan® on cyp3a4 activity in vitro | European journal of drug metabolism and pharmacokinetics | 40 (1), 115-118 | 10.1007/s13318-014-0173-1 | Different domain |
| E. Afrose | In vitro sensitivity test of metronidazole by using clinical isolates of e. Histolytica and e. Bangladeshi | East west university |  |  | Thesis |
| W. Ahmad and p. Negi | N, n-dimethylaniline and zno nanoparticles mediated photochemical transformation of metronidazole | Current chemistry letters | 12(4) , 733-738 | 10.5267/j.ccl.2023.5.002 | Different domain |
| M. A.-f. Ahmed, k. A. Ismail, s. A.-e.-g. Ahmed, a. N. Ibrahim and y. M. Gohar | In vitro activity of curcumin and silver nanoparticles against blastocystis hominis | Infectious diseases in clinical practice | 23(3) , 135-140 | 10.1097/ipc.0000000000000242 | Animal |
| M. S. Al dgihter, s. N. Alvi and m. M. Hammami | A validated assay for rapid determination of tinidazole in human plasma by reversed phase high performance liquid chromatography | European journal of pharmaceutical and medical research | 8 (4), 68-72 |  | Different domain |
| M. Z. Alam, x. Wu, c. Mascio, l. Chesnel and j. G. Hurdle | Mode of action and bactericidal properties of surotomycin against growing and nongrowing clostridium difficile | Antimicrobial agents and chemotherapy | 59 (9) , 5165-5170 | Https://doi.org/10.1128/aac.01087-15 | Different domain |
| C. Alcott, y. Nout‐lomas, d. Wong, m. Aleman and e. Wilkes | Examination, therapeutics, and monitoring of the nervous system | Equine neonatal medicine | 2 (1), 724-758 | Https://doi.org/10.1002/9781119617228.ch30 | Review |
| R. N. T. Al-daemi and h. T. Ghanim | Synthesis and characterization some of imidazol derivatives | Neuroquantology | 18 (9), 107-126 | 10.14704/nq.2020.18.9.nq20223 | Different domain |
| M. Algargoosh, s. Ritchie, e. Duffy, b. Van der werf, m. Thomas and n. Martini | The impact of a pharmacist-led intravenous to oral switch of metronidazole: a before-and-after study | Antibiotics | 11(10), 1303-1309 | https://doi.org/10.3390/antibiotics11101303 | Different domain |
| M. A. Ali | Ex vivo dermis microdialysis: a tool for bioequivalence testing of topical dermatological drug product (demonstration of proof of concept and testing) | Long island university, brooklyn, |  |  | Thesis |
| V. Ali and t. Nozaki | Current therapeutics, their problems, and sulfur-containing-amino-acid metabolism as a novel target against infections by “amitochondriate” protozoan parasites | Clinical microbiology reviews | 20 (1), 164-187 | <https://doi.org/10.1128/cmr.00019-06> | Review |
| Velghe, sofie de troyer, rani stove, christophe | Dried blood spots in therapeutic drug monitoring and toxicology | Expert opinion on drug metabolism & toxicology | 14(1), 1-3 | Https://doi.org/10.1080/17425255.2018.1414181 | Abstract |
| I. D. S. O. America | White paper: recommendations on the conduct of superiority and organism-specific clinical trials of antibacterial agents for the treatment of infections caused by drug-resistant bacterial pathogens | Clinical infectious diseases | 55 (8), 1031-1046 | Https://doi.org/10.1093/cid/cis688 | Different domain |
| W. K. Amery | Why there is a need for pharmacovigilance | Pharmacoepidemiology and drug safety | 8 (1), 61-64 | [https://doi.org/10.1002/(sici)1099-1557](https://doi.org/10.1002/(SICI)1099-1557(199901/02)8:1%3C61::AID-PDS395%3E3.0.CO;2-A) | Review |
| M. L. Amin, t. Ahmed and m. A. Mannan | Development of floating-mucoadhesive microsphere for site specific release of metronidazole | Advanced pharmaceutical bulletin | 6 (2), 195-200 | 10.15171/apb.2016.027 | Different domain |
| G. An | The utility of pharmacometric models in clinical pharmacology research in infants | Current pharmacology reports | 6, 260-266 | Https://doi.org/10.1007/s40495-020-00234-5 | Review |
| J. Ansari, b. Carvalho, s. L. Shafer and p. Flood | Pharmacokinetics and pharmacodynamics of drugs commonly used in pregnancy and parturition | Anesthesia & analgesia | 122 (3) , 786-804 | 10.1097/01.sa.0000504622.07525.6e | Review |
| A. Aracil and j. Green | Plants with antimalarial properties: a systematic review of the current clinical evidence | European journal of integrative medicine | 28 (2019), 76-85 | 10.1016/j.eujim.2019.04.005 | Review |
| N. R. Armstrong and j. D. Wilson | Tinidazole in the treatment of bacterial vaginosis | International journal of women's health | 2009 (1), 59-65 | 10.2147/ijwh.s4455 | Review |
| S. Aslam, r. J. Hamill and d. M. Musher | Treatment of clostridium difficile-associated disease: old therapies and new strategies | The lancet infectious diseases | 5 (9), 549-557 | 10.1016/s1473-3099(05)70215-2 | Review |
| R. Atherton, h. Mckenzie and m. Furr | Behandlung der akuten kolitis beim pferd | Tierärztliche praxis ausgabe g: großtiere/nutztiere | 38 (6), 381-390 | 10.1055/s-0038-1624002 | Abstract |
| A. F. Attah, a. A. Fagbemi, o. Olubiyi, h. Dada-adegbola, a. Oluwadotun, a. Elujoba and c. P. Babalola | Therapeutic potentials of antiviral plants used in traditional african medicine with covid-19 in focus: a nigerian perspective | Frontiers in pharmacology | 12 (1) , 585-596 | https://doi.org/10.3389/fphar.2021.596855 | Abstract |
| J. Autmizguine, p. Brian smith, m. Sampson, p. Ovetchkine, m. Cohen-wolkowiez and k. M watt | Pharmacokinetic studies in infants using minimal-risk study designs | Current clinical pharmacology | 9 (4), 350-358 |  | Not accessible |
| L. M. Avery, c. E. Fominaya, r. C. Crawford, k. P. Pleasants and d. J. Taber | Characterization of potentially unsafe ambulatory antibiotic use and associated outcomes in an adult kidney transplant population | Annals of pharmacotherapy | 52 (10) , 974-982 | 10.1177/1060028018776606 | Abstract |
| S. K. Avvari | Application of physiologically based pharmacokinetic (pbpk) modeling to study the impact of roux-en-y gastric bypass (rygb) surgery on the bioavailability of oral antibiotics | Long island university |  |  | Thesis |
| E. E. Ayogu, k. C. Amorha and o. Okpalaoka | Evaluation of zingiber officinale effects on rifampicin pharmacokinetic parameters using animal model | International journal of pharmaceutical research | 10 (3), 558-564 |  | Not accessible |
| S. H. Bae, h. S. Kim, h. G. Choi, s.-y. Chang and s. H. Kim | Effects of dextran sulfate sodium-induced ulcerative colitis on the disposition of tofacitinib in rats | Biomolecules & therapeutics | 30 (6) , 510-51 | 10.4062/biomolther.2022.049 | Animal |
| J. D. Baggot | Clinical pharmacokinetics in veterinary medicine | Clinical pharmacokinetics | 22 (4) , 254-273 | 10.2165/00003088-199222040-00002 | Animal |
| S. Baglie | Concentrações plasmáticas e salivares e efeito sobre a microbiota oral de duas formulações de amoxicilina. Estudo em voluntários sadios | Faculdade de odontologia de piracicaba sinvaldo baglie farmacêutico …, |  |  | Thesis |
| M. Bahmani, k. Saki, m. Rafieian-kopaei, s. A. Karamati, z. Eftekhari and m. Jelodari | The most common herbal medicines affecting sarcomastigophora branches: a review study | Asian pacific journal of tropical medicine | 7 (1), 14-21 | Https://doi.org/10.1016/s1995-7645(14)60198-x | Review |
| J. P. Bai, j. S. Barrett, g. J. Burckart, b. Meibohm, h. C. Sachs and l. Yao | Strategic biomarkers for drug development in treating rare diseases and diseases in neonates and infants | The aaps journal | 15 (1), 447-454 | 10.1208/s12248-013-9452-z | Review |
| M. Bailén, i. Díaz-castellanos, i. Azami-conesa, s. Alonso fernández, r. A. Martínez-díaz, j. Navarro-rocha, m. T. Gómez-muñoz and a. González-coloma | Anti-trichomonas gallinae activity of essential oils and main compounds from lamiaceae and asteraceae plants | Frontiers in veterinary science | 9 (33), 981-988 | Https://doi.org/10.3389/fvets.2022.981763 | Different domain |
| U. Balaban, a. Inkaya and e. Kara | Nitroimidazole derivative antimicrobial drugs and liver injury | Flora infeksiyon hastaliklari ve klinik mikrobiyoloji dergisi | 28 (3), 313-320 | 10.5578/flora.20237538 | Different domain |
| S. J. Balevic and m. Cohen‐wolkowiez | Innovative study designs optimizing clinical pharmacology research in infants and children | The journal of clinical pharmacology | 58 (10) , 58-72 | 10.1002/jcph.1053 | Review |
| M. L. Banholzer, c. Wandel, p. Barrow, m. Mannino, g. Schmitt, m. Guérard, l. Müller, g. Greig, k. Amemiya and r. Peck | Clinical trial considerations on male contraception and collection of pregnancy information from female partner: update | Clinical and translational medicine | 5 (1) , 1-14 | 10.1186/s40169-016-0103-8 | Review |
| J. Bao, y. Hu, q. Mei, h. Zhen and j. Xu | Effect of rabeprazole on the transport and distribution of levofloxacin in rat stomachs | Experimental and therapeutic medicine | 8 (6) , 1884-1890 | 10.3892/etm.2014.2031 | Animal |
| C. K. Barbosa, v. N. Teixeira and c. T. Pimpão | Antibiotic usage patterns in exotic pets: a study in curitiba, paraná, brazil | Open veterinary journal | 13 (12) , 1543-1549 | 10.5455/ovj.2023.v13.i12.4 | Abstract |
| E. Bardi, a. Vetere, v. Aquaro, e. Lubian, s. Lauzi, g. Ravasio, d. D. Zani, m. Manfredi, m. Tecilla and p. Roccabianca | Use of thrombocyte–leukocyte-rich plasma in the treatment of chronic oral cavity disorders in reptiles: two case reports | Journal of exotic pet medicine | 29 (2019), 32-39 | Https://doi.org/10.1053/j.jepm.2018.06.008 | Different domain |
| C. I. Barker, j. F. Standing, m. A. Turner, j. C. Mcelnay and m. Sharland | Antibiotic dosing in children in europe: can we grade the evidence from pharmacokinetic/pharmacodynamic studies–and when is enough data enough? | Current opinion in infectious diseases | 25 (3), 235-242 | 10.1097/qco.0b013e328353105c | Review |
| H. W. Barron | Appendix ii formulary for common wildlife species | Medical management of wildlife species | 4(1), 449-469 | 10.1002/9781119036708 | Abstract |
| C. E. Barry, h. I. Boshoff and c. S. Dowd | Prospects for clinical introduction of nitroimidazole antibiotics for the treatment of tuberculosis | Current pharmaceutical design | 10 (26), 3239-3262 | <https://doi.org/10.2174/1381612043383214> | Different domain |
| L. C. Bartel, m. M. De mecca and j. A. Castro | Nitroreductive metabolic activation of some carcinogenic nitro heterocyclic food contaminants in rat mammary tissue cellular fractions | Food and chemical toxicology | 47 (1) , 140-144 | 10.1016/j.fct.2008.09.069 | Different domain |
| V. I. C. Bastos | Levels and effects of indoor contaminants in european pets | Universidade de aveiro (portugal), |  |  | Thesis |
| R. J. Beechinor, m. Cohen-wolkowiez, t. Jasion, c. P. Hornik, j. E. Lang, r. Hernandez and d. Gonzalez | A dried blood spot analysis for solithromycin in adolescents, children, and infants: a short communication | Therapeutic drug monitoring | 41 (6) , 761-765 | 10.1097/ftd.0000000000000670 | Abstract |
| O. Befani, e. Grippa, l. Saso, p. Turini and b. Mondovi | Inhibition of monoamine oxidase by metronidazole | Inflammation research | 50 (2) , 136-137 | 10.1007/pl00022395 | Different domain |
| W. E. Bell | Treatment of bacterial infections of the central nervous system | Annals of neurology: official journal of the american neurological association and the child neurology society | 9 (4) , 313-327 | <https://doi.org/10.1002/ana.410090402> | Abstract |
| A. Bendesky and d. Menéndez | Metronidazole: a comprehensive view | Revista de la facultad de medicina unam | 44 (6) , 255-259 |  | Not accessible |
| W. Bennett | Guide to drug dosage in renal failure | Clinical pharmacokinetics | 15 (5), 326-354 | 10.1001/jama.1974.03240110036016 | Abstract |
| W. M. Bennett, r. S. Muther, r. A. Parker, p. Feig, g. Morrison, t. A. Golper and i. Singer | Drug therapy in renal failure: dosing guidelines for adults: part i: antimicrobial agents, analgesics | Annals of internal medicine | 93 (1) , 62-89 | 10.1097/00043764-198203000-00024 | Abstract |
| W. M. Bennett, i. Singer, t. Golper, p. Feig and c. J. Coggins | Guidelines for drug therapy in renal failure | Annals of internal medicine | 86 (6) , 754-783 | 10.7326/0003-4819-86-6-754 | Review |
| T. Bergan | Pharmacokinetic aspects of antibiotic assays | Springer | 191-194 |  | Book |
| T. Bergan, e. Arnold and l. Withander | Comparison of metronidazole assay by microbiological and chemical methods | Methods and findings in experimental and clinical pharmacology | 2(3), 145-150 |  | Not accessible |
| T. Bergan, t. Kalager, k. B. Hellum and c. O. Solberg | Penetration of cefotaxime and desacetylcefotaxime into skin blister fluid | Journal of antimicrobial chemotherapy | 10 (3) , 193-196 | 10.1093/jac/10.3.193 | Abstract |
| S. J. Bergman, c. Speil, m. Short and j. Koirala | Pharmacokinetic and pharmacodynamic aspects of antibiotic use in high-risk populations | Infectious disease clinics of north america | 21 (3) , 821-846 | 10.1016/j.idc.2007.07.004 | Review |
| J. Berkley and k. Jones | Severe, acute malnutrition and infection | Cmam forum |  |  | Book |
| L. H. Bernstein, m. S. Frank, l. J. Brandt and s. J. Boley | Healing of perineal crohn's disease with metronidazole | Gastroenterology | 79 (2) , 357-365 | <https://doi.org/10.1016/0016-5085(80)90155-9> | Different domain |
| J. J. Bertone | Antimicrobial therapy for respiratory disease | Veterinary clinics of north america: equine practice | 13 (3) , 501-517 | [https://doi.org/10.1016/s0749-0739(17)30227-4](https://doi.org/10.1016/S0749-0739(17)30227-4) | Abstract |
| N. Bhojraj | Pathogenic effect of trichomonas vaginalis on various cell lines in vitro | Citeseer |  |  | Book |
| N. H. Binhashim, s. N. Alvi and m. M. Hammami | A validated reversed phase hplc assay for the determination of metronidazole in human plasma | World journal of pharmacy and pharmaceutical sciences | 3 (12) , 32-41 |  | Not accessible |
| J. E. Bollinger | The impact of liver disease on drug metabolism | Surgical procedures on the cirrhotic patient | 29-39 (2), 4 | 10.1007/978-3-319-52396-5_3 | Abstract |
| L. Bomgaars, s. Berg and a. R. Stark | Research in pediatrics | Elsevier | 573-586 | [10.2174/1570179416666190719125730](https://doi.org/10.2174/1570179416666190719125730) | Book |
| P. K. Boniface and f. I. Elizabeth | Flavones as a privileged scaffold in drug discovery: current developments | Current organic synthesis | 16 (7) , 968-1001 | [10.2174/1570179416666190719125730](https://doi.org/10.2174/1570179416666190719125730) | Abstract |
| B. B. Bonner | Chelonian therapeutics | Veterinary clinics of north america: exotic animal practice | 3 (1), 257-332 | [https://doi.org/10.1016/s1094-9194(17)30104-4](https://doi.org/10.1016/S1094-9194(17)30104-4) | Abstract |
| Y. M. Borbély, a. Osterwalder, d. Kröll, p. C. Nett and r. A. Inglin | Diarrhea after bariatric procedures: diagnosis and therapy | World journal of gastroenterology | 23 (26), 4689-4700 | 10.3748/wjg.v23.i26.4689 | Review |
| P. Boreham, j. Upcroft and p. Upcroft | Biochemical and molecular mechanisms of resistance to nitroheterocyclic drugs in giardia intestinalis | Taylor and francis, london | 594-604 |  | Book |
| S. Bose and m. Kumar | Microwave responsive copper-ferrite (cufe2o4) encapsulated in molybdenum-disulfide (mos2) nanoflower catalyst for antibiotic removal via persulfate oxidation | Surfaces and interfaces | 49 (1), 104428-104434 | <https://doi.org/10.1016/j.surfin.2024.104428> | Abstract |
| J. I. Boullata | Drug-nutrition interactions in infectious diseases | Nutrition and infectious diseases: shifting the clinical paradigm | 4 (2), 367-410 |  | Not accessible |
| I. Brook | Pediatric anaerobic infections: diagnosis and management | Crc press, |  |  | Book |
| I. Brook | Meningitis and shunt infection caused by anaerobic bacteria in children | Pediatric neurology | 26 (2), , 99-105 | 10.1016/s0887-8994(01)00330-7 | Review |
| T. A. Broome, m. P. Brown, r. R. Gronwall, m. F. Casey and k. A. Meritt | Pharmacokinetics and plasma concentrations of acetylsalicylic acid after intravenous, rectal, and intragastric administration to horses | Canadian journal of veterinary research | 67 (4) , 297-302 |  | Not accessible |
| S. Brown | Mediterranean tortoises | Handbook of exotic pet medicine | 8 (1), 327-359 | <https://doi.org/10.1002/9781119389934.ch18> | Abstract |
| E. Brunet, a. Caixàs and v. Puig | Review of the management of diarrhea syndrome after bariatric surgery | Endocrinología, diabetes y nutrición | 67 (6) , 401-407 | <https://doi.org/10.1016/j.endien.2020.08.005> | Review |
| J. Bryant, m. Brown, r. Gronwall and k. Merritt | Study of intragastric administration of doxycycline: pharmacokinetics including body fluid, endometrial and minimum inhibitory concentrations | Equine veterinary journal | 32 (3) , 233-238 | <https://doi.org/10.2746/042516400776563608> | Abstract |
| A. Bryskier, j. Lowther and c. Couturier | Helicobacter pylori and antibacterial agents | Antimicrobial agents: antibacterials and antifungals | 2 (1), 1011-1054 | <https://doi.org/10.1128/9781555815929.ch40> | Abstract |
| A. S. Buck, e. D. Martin, j. F. Mazzuchi, m. Merry and e. Mendez jr | The department of defense civilian external peer review program: an interim report | Military medicine | 157 (1) , 40-46 | 10.1093/milmed/157.1.40 | Review |
| F. V. Büdingen, d. Gonzalez, a. N. Tucker and h. Derendorf | Relevance of liver failure for anti-infective agents: from pharmacokinetic alterations to dosage adjustments | Therapeutic advances in infectious disease | 2 (1) , 17-42 | 10.1177/2049936113519089 | Review |
| H. G. N. O. Busatti | Investigação in vitro do potencial giardicida de quatro análogos do metronidazol, | Investigação | 8 (2006), 1-63 | Http://hdl.handle.net/1843/sagf-6xsnex | Language |
| H. Cai, y. Liu, y. Zhu, s. Fang, d. Wang, z. Yan, h. Shen, s. Liao, n. Qi and j. Li | Drug resistance patterns and genotype associations of trichomonas gallinae in meat pigeons (columba livia): insights from guangdong province, china | Frontiers in veterinary science | 10 (1) , 123-134 | <https://doi.org/10.3389/fvets.2023.1343321> | Animal |
| K. Calloe, s. Rognant, s. Friis, c. Shaughnessy, d. A. Klaerke and d. Trachsel | Compounds commonly used in equine medicine inhibits the voltage-gated potassium channel kv11. 1 | Research in veterinary science | 123 (1), 239-246 | <https://doi.org/10.1016/j.rvsc.2019.01.009> | Abstract |
| C. C. Candido, h. V. R. Silva, b. Zavan, m. Ionta, m. I. F. Barbosa and a. C. Doriguetto | Synthesis, characterization and in vitro cytotoxicity of ruthenium (ii) metronidazole complexes: cell cycle arrest at g1/s transition and apoptosis induction in mcf-7 cells | Journal of inorganic biochemistry | 237 (1) , 112-122 | 10.1016/j.jinorgbio.2022.112022 | Abstract |
| A. Carmine, r. Brogden, r. Heel, t. Speight and g. Avery | Tinidazole in anaerobic infections: a review of its antibacterial activity, pharmacological properties and therapeutic efficacy | Drugs | 24 (1), 85-117 | 10.12-6667/82/0800-0085/$16.50/0 | Abstract |
| S. Casella, c. Giannetto, e. Giudice, s. Marafioti, f. Fazio, a. Assenza and g. Piccione | Adp-induced platelet aggregation after addition of tramadol in vitro in fed and fasted horses plasma | Research in veterinary science | 94 (2), 325-330 | <https://doi.org/10.1016/j.rvsc.2012.09.005> | Abstract |
| C. Caulfield, k. Roper and c. Teevan | Use of oral antibiotics in bone and joint infections | Connecticut medicine | 84 (3), 129-134 |  | Not accessible |
| J. R. Cedeiio and d. J. Krogstad | Susceptibility testing of entamoeba histolytica | Journal of infectious diseases | 148 (6) , 1090-1095 | 10.1093/infdis/148.6.1090 | Different domain |
| S. Chaturvedi, m. Y. Malik, m. Rashid, s. Singh, v. Tiwari, p. Gupta, s. Shukla, s. Singh and m. Wahajuddin | Mechanistic exploration of quercetin against metronidazole induced neurotoxicity in rats: possible role of nitric oxide isoforms and inflammatory cytokines | Neurotoxicology | 79 (2020) , 1-10 | <https://doi.org/10.1016/j.neuro.2020.03.002> | Abstract |
| J. Chiarizia | Poster communications session 1: antimicrobials & antibiotic resistance, | Journal of veterinary pharmacology and theraputics | 38 (1), 81-173 | 10.1111/jvp.12247. | Animal |
| S. Chien, d. Gorman, c.-p. Koutsogiannidis, r. Ravishankar, g. Kamath and v. Zamvar | The novel use of oral antibiotic monotherapy in prosthetic valve endocarditis caused by finegoldia magna: a case study | The novel use of oral antibiotic monotherapy in prosthetic valve endocarditis caused by finegoldia magna: a case study | 14 (2019) , 1-5 | https://doi.org/10.1186/s13019-019-0993-9 | Review |
| D. L. Church, r. D. Bryant, h. R. Rabin and e. J. Laishley | Physiolgical effects of metronidazole on clostridium posteurianum | Journal of antimicrobial chemotherapy | 28 (2), 221-228 | <https://doi.org/10.1093/jac/28.2.221> | Abstract |
| O. Cionca, z. Hadnagy, a. Murariu and m. Zahner | Bacterial vaginosis in pregnancy: professional diagnostics as a basis for an optimized therapy | Obstetrica si ginecologie | 65 (4), 199-204 |  | Not accessible |
| E. S. Clark and j. L. Becht | Clinical pharmacology of the gastrointestinal tract | Veterinary clinics of north america: equine practice | 3 (1), 101-122 | [https://doi.org/10.1016/s0749-0739(17)30693-4](https://doi.org/10.1016/S0749-0739(17)30693-4) | Animal |
| J. F. Cocchetto, d. M. Cocchetto, t. D. Bjornsson and t. Bergan | Initial slope technique for estimation of the apparent volume of distribution during constant-rate intravenous infusion | Journal of pharmaceutical sciences | 73 (1), 58-62 | <https://doi.org/10.1002/jps.2600730115> | Abstract |
| D. J. Coleman and a. G. Batchelor | Gas gangrene | The new england journal of medicine | 29 (2), 1129-1131 | 10.1056/nejm197311222892107 | Abstract |
| K. Corley and a. Hollis | Antimicrobial therapy in neonatal foals | Equine veterinary education | 21 (8) , 436-448 | [10.2746/095777309x445352](https://doi.org/10.2746/095777309X445352) | Animal |
| F. Cossu, g. Rombi, s. Mazza and a. Facchini | Radiosensitization by tinidazole: pharmacokinetic study | International journal of clinical pharmacology research | 6 (40) , 317-323 |  | Not accessible |
| C. M. Cotten | Adverse consequences of neonatal antibiotic exposure | Current opinion in pediatrics | 28 (2) , 141-149 | 10.1097/mop.0000000000000338 | Review |
| S. Cox, m. C. Allender and j. Yarbrough | Determination of metronidazole in adult artemia using high performance liquid chromatography | Journal of liquid chromatography & related technologies | 33 (1), 89-96 | 10.1080/10826070903430381 | Different domain |
| B. A. Cunha | Oral antibiotic therapy of serious systemic infections | Medical clinics | 90 (6) , 1197-1222 | 10.1016/j.mcna.2006.07.009 | Review |
| A. E. Cury and m. P. M. Hirschfeld | Interactions between amphotericin b and nitroimidazoles against candida albicans | Mycoses | 40 (6) , 187-192 | <https://doi.org/10.1111/j.1439-0507.1997.tb00212.x> | Animal |
| A. S. Da silva, c. E. Da rosa silva, f. R. Paula and f. E. B. Da silva | Discriminative dissolution method for benzoyl metronidazole oral suspension | Aaps pharmscitech | 17 (2015) , 778-786 | 10.1208/s12249-015-0407-9 | Different domain |
| S. Dallefeld, c. D. Hornik, k. Zimmerman and m. Cohen-wolkowiez | Antibiotic dosing considerations for term and preterm infants | Elsevier | 167-184 |  | Book |
| V. Dartois and c. E barry | Clinical pharmacology and lesion penetrating properties of second-and third-line antituberculous agents used in the management of multidrug-resistant (mdr) and extensively-drug resistant (xdr) tuberculosis | Current clinical pharmacology | 5 (2) , 96-114 | 10.2174/157488410791110797 | Different domain |
| E. Darweish, a. M. Abdel-raoof, h. M. Marzouk, a. M. Ashmawy, y. M. Fayez and m. S. Eissa | Innovative ph-dependent approach for electrochemical determination of a triple eradication therapy targeting h. Pylori infection in pharmaceutical formulation and human plasma sample: modified electrode with prussian blue analogue decorated multi-walled carbon nanotubes (pba@ mwcnt) | Microchemical journal | 181 (2022) , 107784-107789 | Https://doi.org/10.1016/j.microc.2022.107784 | Abstract |
| E. Darweish, m. S. Eissa, y. M. Fayez and h. M. Marzouk | Chromatographic estimation of a novel triple-therapy combination targeting helicobacter pylori eradication in different matrices | Bioanalysis | 13 (20) , 1547-1557 | <https://doi.org/10.4155/bio-2021-0183> | Abstract |
| J. Davis | Antimicrobial therapy in the neonatal foal | Equine neonatal medicine | 29( 2), 1328-1343 | <https://doi.org/10.1002/9781119617228.ch61> | Animal |
| J. L. Davis | Introduction to equine pharmacotherapy | Pharmacotherapeutics for veterinary dispensing | 8 (3), 471-500 | <https://doi.org/10.1002/9781119404576.ch21> | Abstract |
| J. M. Davis, e. M. Connor and a. J. Wood | The need for rigorous evidence on medication use in preterm infants: is it time for a neonatal rule? | Jama | 308 (14) , 1435-1436 | 10.1001/jama.2012.12883 | Review |
| W. A. Dayyih, m. Hailat, h. H. Omaral-asasfeh and h. Thabet | The impact of pomegranate juice on the pharmacokinetic of different drugs, | Sapporo medical journal | 58(1), 1-8 |  | Not accessible |
| A. De boer, f. Moolenaar, l. De leede and d. Breimer | Rectal drug administration: clinical pharmacokinetic considerations | Clinical pharmacokinetics | 7 (1) , 285-311 | 10.2165/00003088 | Abstract |
| G. Delis, m. Koutsoviti-papadopoulou and v. Siarkou | Pharmacodynamics of amoxicillin against pasteurella, | Journal of veterinary pharmacology and theraputics | 32(1), 129–265 | 10.1111/j.1365-2885.2009.01091.x. | Animal |
| E. P. Dellinger | Cephalosporin plus metronidazole for surgical prophylaxis | Surgical infections | 19 (4) , 359-361 | 10.1089/sur.2018.023 | Abstract |
| M. Děrgel, m. Voborník, m. Pojar, m. Karalko, j. Gofus, v. Radochová, š. Studená, j. Maláková, z. Turek and j. Chládek | Lung collapse during mini-thoracotomy reduces penetration of cefuroxime to the tissue: interstitial microdialysis study in animal models | Surgical infections | 22 (3) , 283-291 | <https://doi.org/10.1089/sur.2019.273> | Animal |
| S. Devi, a. Chaudhary, s. Singla and c. Kumari | A recent approach for developing an anti-malarial phytomedicine | Indian journal of pharmaceutical and biological research | 9 (3), 10-15 | Https://doi.org/10.30750/ijpbr.9.3.2 | Animal |
| E. Di cicco, e. Paradis, c. Stephen, m. E. Turba and g. Rossi | Scuticociliatid ciliate outbreak in australian pot-bellied seahorse, hippocampus abdominalis (lesson, 1827): clinical signs, histopathologic findings, and treatment with metronidazole | Journal of zoo and wildlife medicine | 44 (2), 435-440 | http://dx.doi.org/10.1638/2012-127r1.1 | Review |
| B. Dijkmans, h. Mattie, j. Hermans and r. Van furth | Quantification of the effect of anti-anaerobic drugs in experimental bacteroides fragilis infection in mice | Journal of antimicrobial chemotherapy | 13 (1), 79-86 | 10.1093/jac/13.1.79 | Animal |
| B. A. Dijkmans, j. Vaishnav-nair and h. Mattie | Efficacy of rifamycin sv and vancomycin against bacteroides fragilis in vitro and in experimentally infected mice | Current microbiology | 12 (1), 53-58 | 10.1007/bf01567754 | Animal |
| S. Dingsdag | Metabolism of a deuteroporphyrin-nitroimidazole adduct by gastrointestinal bacteria |  |  |  | Thesis |
| G. V. Doern | Optimizing the management of community-acquired respiratory tract infections in the age of antimicrobial resistance | Expert review of anti-infective therapy | 4 (5), 821-835 | <https://doi.org/10.1586/14787210.4.5.821> | Abstract |
| R. Donnelly and g. J. Macphee | Clinical pharmacokinetics and kinetic-dynamic relationships of dilevalol and labetalol | Clinical pharmacokinetics | 21 (2) , 95-109 | 10.2165/00003088-199121020-00002 | Review |
| T. Dorofaeff, r. M. Bandini, j. Lipman, d. E. Ballot, j. A. Roberts and s. L. Parker | Uncertainty in antibiotic dosing in critically ill neonate and pediatric patients: can microsampling provide the answers? | Clinical therapeutics | 38 (9), 1961-1975 | 10.1016/j.clinthera.2016.07.093 | Different domain |
| P. M. Dowling | Miscellaneous antimicrobials: ionophores, nitrofurans, nitroimidazoles, rifamycins, and others | Antimicrobial therapy in veterinary medicine | 8(4), 315-332 | <https://doi.org/10.1002/9781118675014.ch19> | Abstract |
| B. Dunkel | Antimicrobial drug use in critically ill horses | Uk-vet equine | 2 (5), 134-138 | 10.12968/ukve.2018.2.5.134 | Animal |
| R. E. Durand and p. L. Olive | Evaluation of nitroheterocyclic radiosensitizers using spheroids | Elsevier | 9, 75-107 |  | Book |
| K. Eatwell | Antibiotic therapy in reptiles | Journal of herpetological medicine and surgery | 17 (2) , 42-49 | <https://doi.org/10.5818/1529-9651.17.2.42> | Abstract |
| A. M. El-kady, i. A. Abdel-rahman, s. S. Fouad, k. S. Allemailem, t. Istivan, s. F. Ahmed, a. S. Hasan, h. A. Osman and h. A. Elshabrawy | Pomegranate peel extract is a potential alternative therapeutic for giardiasis | Antibiotics | 10 (6) , 2-15 | 10.3390/antibiotics10060705 | Different domain |
| N. D. Embleton, e. Turnbull, s. Turner and j. E. Berrington | Successful blood salvaging from preterm infants: maximizing opportunities, minimizing interventions | Acta paediatrica | 102 (6), 527-529 | 10.1111/apa.12373 | Different domain |
| D. Emiliano di cicco, e. Paradis, c. Stephen, m. Elena, d. Turba and g. Rossi | Scuticociliatid ciliate outbreak in australian pot | Journal of zoo and wildlife medicine | 44 (2), 435-440 | 10.1016/j.cct.2016.03.002 | Review |
| A. England, k. Wade, p. B. Smith, k. Berezny, m. Laughon and b. P. F. C. A. P. T. N. A. C. Committee | Optimizing operational efficiencies in early phase trials: the pediatric trials network experience | Contemporary clinical trials | 47 (1) , 376-382 | 10.1016/j.cct.2016.03.002 | Different domain |
| C. O. Esimone, c. P. Ihekwereme, i. E. Okoye and m. Adikwu | Urinary elimination of ofloxacin in humans is reduced by tea and kola nitida seed | African journal of pharmaceutical sciences and pharmacy | 4 (1), 71-82 |  | Animal |
| J. Espinosa-aguirre, r. De la torre, i. Lares-asseff, j. Rubio, v. Dorado, m. Wong and j. Hernandez | Bacterial mutagens in the urine of patients under tinidazole treatment | Mutation research/environmental mutagenesis and related subjects | 359 (2), 133-140 | 10.1016/s0165-1161(96)90259-9 | Different domain |
| A. Fakhri, s. Rashidi, m. Asif and a. A. Ibrahim | Microwave-assisted synthesis of sic nanoparticles for the efficient adsorptive removal of nitroimidazole antibiotics from aqueous solution | Applied sciences | 7 (2) , 2-12 | 10.3390/app7020205 | Different domain |
| X. Fan, x. Ding and q.-y. Zhang | Hepatic and intestinal biotransformation gene expression and drug disposition in a dextran sulfate sodium-induced colitis mouse model | Acta pharmaceutica sinica b | 10 (1), 123-135 | <https://doi.org/10.1016/j.apsb.2019.12.002> | Abstract |
| Z. Fang, j. Chen, x. Qiu, x. Qiu, w. Cheng and l. Zhu | Effective removal of antibiotic metronidazole from water by nanoscale zero-valent iron particles | Desalination | 268 (3), 60-67 | 10.1016/j.desal.2010.09.051 | Review |
| W. E. Feldman | Bacteroides fragilis ventriculitis and meningitis: report of two cases | American journal of diseases of children | 130 (8), 880-883 | 10.1001/archpedi.1976.02120090090017 | Abstract |
| S. Fest | Antibiotische therapie | Therapeutische grundsätze | 125 (1), 61-67 |  | Not accessible |
| M. Francke, w. Visser, d. Severs, a. De mik-van egmond, d. Hesselink and b. De winter | Body composition is associated with tacrolimus pharmacokinetics in kidney transplant recipients | European journal of clinical pharmacology | 78 (8) , 1273-1287 | 10.1007/s00228-022-03323-0 | Abstract |
| A. G. Fraser | Pharmacokinetic interactions between alcohol and other drugs | Clinical pharmacokinetics | 33 (1), 79-90 | 03 i 2-5963/97/0008-oo79/s06.oolo | Different domain |
| T. Freimann | Musculoskeletal pain among nurses: prevalence, risk factors, and intervention, |  |  |  | Thesis |
| H. B. Fung and t.-l. Doan | Tinidazole: a nitroimidazole antiprotozoal agent | Clinical therapeutics | 27 (12), 1859-1884 | 10.1016/j.clinthera.2005.12.012 | Review |
| R. S. Funk | A formulary for lizards, snakes, and crocodilians | Veterinary clinics of north america: exotic animal practice | 3 (1), 333-358 | 10.1016/s1094-9194(17)30105-6 | Animal |
| M. I. Gadallah, h. R. H. Ali, h. F. Askal and g. A. Saleh | Innovative hptlc-densitometric method for therapeutic monitoring of meropenem and metronidazole in acute pancreatic patients | Microchemical journal | 146 (2019) , 940-947 | 10.1016/j.microc.2019.02.011 | Different domain |
| E. G. Garcia | Treatment of symptomatic intestinal amoebiasis with tinidazole | Drugs | 15 (1), 16-18 | 10.2165/00003495-197800151-00003 | Different domain |
| T. B. Gardner and d. R. Hill | Treatment of giardiasis | Clinical microbiology reviews | 14 (1) , 114-128 | <https://doi.org/10.1128/cmr.14.1.114-128.2001> | Abstract |
| U. Ghaffar, k. Lukose, m. As sayaideh, b. Dass and n. S. Radhakrishnan | Long-term use of metronidazole mimicking hepatic encephalopathy | Annals of internal medicine: clinical cases | 1 (1) , 1-2 | <https://doi.org/10.7326/aimcc.2021.0178> | Review |
| S. Ghosh, o. Falyouna, h. Onyeaka, a. Malloum, c. Bornman, s. S. Alkafaas, z. T. Al-sharify, s. Ahmadi, m. H. Dehghani and a. H. Mahvi | Recent progress on the remediation of metronidazole antibiotic as emerging contaminant from water environments using sustainable adsorbents: a review | Journal of water process engineering | 51 (2023) , 103-405 | <https://doi.org/10.1016/j.jwpe.2022.103405> | Review |
| P. M. Gibbons | Advances in reptile clinical therapeutics | Journal of exotic pet medicine | 23 (1), 21-38 | 10.1053/j.jepm.2013.11.007 | Review |
| F. Giordanetto, j. Boström and c. Tyrchan | Follow-on drugs: how far should chemists look? | Drug discovery today | 16 (15), 722-732 | 10.1016/j.drudis.2011.05.011 | Review |
| C. Golledge, a. Keil and t. Mckenzie | Crisis in medical education | British medical journal | 296 (1), 1601-1602 |  | Abstract |
| T. A. Golper, m. A. Marx, c. Shuler and w. M. Bennett | Drug dosage in dialysis patients | Replacement of renal function by dialysis | 29 (1), 750-820 | 10.1007/978-0-585-36947-1_30 | Review |
| F. R. Gómez, f. S. Serrano, a. M. Egea, g. G. Del castillo, j. S. Cuenca and m. C. Planas | Alta eficacia erradicadora de la infección por helicobacter pylori de una pauta terapéutica intravenosa de 3 días de duración en pacientes con úlcera péptica sangrante | Gastroenterología y hepatología | 25 (6) , 383-386 | 10.1016/s0210-5705(02)70270-7 | Different domain |
| D. Gonzalez, d. L. Palazzi, l. Bhattacharya-mithal, a. Al-uzri, l. P. James, j. Bradley, n. Neu, t. Jasion, c. P. Hornik and p. B. Smith | Solithromycin pharmacokinetics in plasma and dried blood spots and safety in adolescents | Antimicrobial agents and chemotherapy | 60 (4) , 2572-2576 | 10.1128/aac.02561-15 | Different domain |
| D. Gonzalez, i. M. Paul, d. K. Benjamin and m. Cohen-wolkowiez | Advances in pediatric pharmacology, therapeutics, and toxicology | Advances in pediatrics | 61 (1) , 7-31 | 10.1016/j.yapd.2016.04.015 | Review |
| L. González, m. Frajman, e. Sáenz, r. Boza and h. Bolaños | Effect of tinidazole on the cellular immune response | Effect of tinidazole on the cellular immune response | 18 (4) , 499-502 | 10.1093/jac/18.4.499 | Different domain |
| P. Gosling, c. P. Shearman and a. J. Sutcliffe | Proteinuria in malignancy | Proteinuria in malignancy | 296 (6636) , 1601-1609 | [10.1136/bmj.296.6636.1601-a](https://doi.org/10.1136%2Fbmj.296.6636.1601-a) | Abstract |
| P. O. Gubbins and k. E. Bertch | Drug absorption in gastrointestinal disease and surgery | Pharmacotherapy: the journal of human pharmacology and drug therapy | 9 (5) , 285-295 | 10.2165/00003088-199121060-00004 | Review |
| Y. C. Guerra valero, s. C. Wallis, j. Lipman, c. Stove, j. A. Roberts and s. L. Parker | Clinical application of microsampling versus conventional sampling techniques in the quantitative bioanalysis of antibiotics: a systematic review | Bioanalysis | 10 (6) , 407-423 | 10.4155/bio-2017-0269 | Review |
| R. Gupta and b. Atul | Drug metabolism studies in animal models | Indian journal of pharmacology | 32 (4), 62-66 |  | Review |
| R. Gupta, s. Sharma, r. Singh, r. A. Vishwakarma, s. Mignani and p. P. Singh | Functionalized nitroimidazole scaffold construction and their pharmaceutical applications: a 1950–2021 comprehensive overview | Pharmaceuticals | 15 (5), 561-569 | 10.1424-8247/15/5/561 | Abstract |
| K. Gura and l. Chan | Drug therapy and the role of nutrition | Nutrition in pediatrics | 234-250 |  | Book |
| B. Gürcü, y. Başımoğlu koca, s. Inan and m. Tuğlu | Oxidative stress response and cell death in fish intestine due to metronidazole toxicity | Fresenius environmental bulletin | 26 (10), 5849-5857 |  | Not accessible |
| G. Gutierrez and o. Muñoz | Epidemiology of amebiasis | Crc press | 173-189 | 10.1201/9780429282539-12 | Book |
| E. Haggett and w. Wilson | Overview of the use of antimicrobials for the treatment of bacterial infections in horses | Equine veterinary education | 20 (8), 433-448 | 10.2746/095777308x338893 | Animal |
| I. Haller | In vitro activity of the two principal oxidative metabolites of metronidazole against bacteroides fragilis and related species | Antimicrobial agents and chemotherapy | 22 (1), 165-166 | 10.1128/aac.22.1.165 | Animal |
| O. Halter, t. Bergun, t. Flørenes and o. Leinebø | Penetration of metronidazole to tissues | Journal of antimicrobial chemotherapy | 11 (4), 357-360 | <https://doi.org/10.1093/jac/11.4.357> | Abstract |
| J. Hampson | The use of metronidazole in the treatment of malodorous wounds | Journal of wound care | 5 (9), 421-426 | 10.12968/jowc.1996.5.9.421 | Different domain |
| S. Hao, y. Wang, b. Wang, q. Zou, h. Zeng, x. Chen, x. Liu, j. Liu and s. Yu | A novel gastroretentive porous microparticle for anti-helicobacter pylori therapy: preparation, in vitro and in vivo evaluation | International journal of pharmaceutics | 463 (1), 10-21 | 10.1016/j.ijpharm.2013.12.052 | Different domain |
| M. Hares, f. Greca, d. Youngs, s. Bentley, d. Burdon and m. Keighley | Failure of antimicrobial prophylaxis with cefoxitin, or metronidazole and gentamicin in colorectal surgery. Is mannitol to blame? | Journal of hospital infection | 2 (1), 127-133 | <https://doi.org/10.1016/0195-6701(81)90021-9> | Abstract |
| C. Hart, r. Smyth and b. Duerden | Infections in children: proceedings of the fourth liverpool school of tropical medicine and bayer symposium on microbial disease | Journal of medical microbiology | 47 (11), 943-982 | 10.1099/00222615-47-11-943 | Abstract |
| M. H. Hart and j. D. Cherry | Cefamandole failure in haemophilus influenzae endocarditis | The pediatric infectious disease journal | 4 (6) , 695-696 | 10.1097/00006454-198511000-00024 | Abstract |
| M. Havelka | Rezistence trichomonas vaginalis a dalších anaerobních patogenů k metronidazolu, |  |  |  | Language |
| S. S. Hayer, s. Hwang and j. B. Clayton | Antibiotic-induced gut dysbiosis and cognitive, emotional, and behavioral changes in rodents: a systematic review and meta-analysis | Frontiers in neuroscience | 17 (1), 123-127 | [| https://doi.org/10.3389/fnins.2023.1237177](https://doi.org/10.3389/fnins.2023.1237177) | Abstract |
| T. Hellebuyck, f. Pasmans, f. Haesebrouck and a. Martel | Dermatological diseases in lizards | The veterinary journal | 193 (1), 38-45 | <https://doi.org/10.1016/j.tvjl.2012.02.001> | Animal |
| U. Hellgren, o. Ericsson and l. L. Gustafsson | Handbook of drugs for tropical parasitic infections | Crc press |  | <https://doi.org/10.1201/9781482272536> | Book |
| P. Hema | Evaluation of a polymerase chain reaction (pcr) assay for the diagnosis of trichomonas vaginalis infection | Christian medical college, vellore |  |  | Thesis |
| K. V. Herrin | Clinical techniques and supportive care | Reptile medicine and surgery in clinical practice | 159-173 | <https://doi.org/10.1002/9781118977705.ch13> | [abstract](https://doi.org/10.1002/9781118977705.ch13) |
| W. L. Hewitt and m. C. Mchenry | Blood level determinations of antimicrobial drugs: some clinical considerations | Medical clinics of north america | 62 (5) , 1119-1140 | 10.1016/s0025-7125(16)31758-8 | Review |
| A. S. Himebauch and a. Zuppa | Methods for pharmacokinetic analysis in young children | Expert opinion on drug metabolism & toxicology | 10 (4) , 497-509 | <https://doi.org/10.1517/17425255.2014.885502> | Abstract |
| T. Hofstad and k. Sveen | Penetration of metronidazole into preformed cavities in rabbits | Journal of antimicrobial chemotherapy | 6 (2) , 275-278 | 10.1093/jac/6.2.275 | Animal |
| W. B.-t. Hong, w. K. Tan, l. S.-c. Law, d. E.-h. Ong and e. A.-g. Lo | Changes of drug pharmacokinetics in patients with short bowel syndrome: a systematic review | European journal of drug metabolism and pharmacokinetics | 46 (4) , 465-478 | 10.1007/s13318-021-00696-y | Review |
| A. M. Hopkins, c. Mcdonnell, n. P. Breslin, c. A. O'morain and a. W. Baird | Omeprazole increases permeability across isolated rat gastric mucosa pre‐treated with an acid secretagogue | Journal of pharmacy and pharmacology | 54 (3) , 341-347 | 10.1211/0022357021778583 | Animal |
| C. K. Horlen, c. F. Seifert and c. S. Malouf | Toxic metronidazole-induced mri changes | Annals of pharmacotherapy | 34 (11) , 1273-1275 | <https://doi.org/10.1345/aph.10028> | Abstract |
| C. P. Hornik, h. Wu, a. N. Edginton, k. Watt, m. Cohen-wolkowiez and d. Gonzalez | Development of a pediatric physiologically-based pharmacokinetic model of clindamycin using opportunistic pharmacokinetic data | Clinical pharmacokinetics | 56 (1) , 1343-1353 | 10.1007/s40262-017-0525-5 | Different domain |
| N. Hu, y. Huang, x. Gao, s. Li, z. Yan, b. Wei and r. Yan | Effects of dextran sulfate sodium induced experimental colitis on cytochrome p450 activities in rat liver, kidney and intestine | Chemico-biological interactions | 271 (1), 48-58 | 10.1016/j.cbi.2017.04.018 | Animal |
| H. Hunt | Precision targeting of intraperitoneal tumors with peptideguided nanocarriers | Dissertationes medicinae universitatis tartuensis |  |  | Thesis |
| N. Idkaidek, t. Arafat, h. Hamadi, s. Hamadi and i. Al-adham | Saliva versus plasma bioequivalence of azithromycin in humans: validation of class i drugs of the salivary excretion classification system | Drugs in r&d | 17 (1), 219-224 | 10.1007/s40268-016-0170-8 | Different domain |
| C. V. Ikefuti | Avaliação ecotoxicológica, bioquímica, histo-hematológica e eficácia de terapêuticos no controle de doenças em pacu, piaractus mesopotamicus, |  |  |  | Language |
| C. J. Innis, k. Conley, p. Gibbons, n. I. Stacy, h. D. Walden, p. Martelli, s. Luz, k. Krishnasamy, c. Hagen and j. Sykes | Veterinary observations and biological specimen use after a massive confiscation of palawan forest turtles (siebenrockiella leytensis) | Chelonian conservation and biology: celebrating 25 years as the world's turtle and tortoise journal | 21 (1), 46-62 | [https://doi.org/10.2744/ccb-1510.1](https://doi.org/10.2744/CCB-1510.1) | Animal |
| C. J. Innis, d. Young, s. Wetzlich, a. J. Whitcomb and l. Tell | Plasma concentrations and safety assessment of voriconazole in red-eared slider turtles (trachemys scripta elegans) after single and multiple subcutaneous injections | Journal of herpetological medicine and surgery | 24 (1) , 28-35 | 10.5818/1529-9651-24.1.28 | Different domain |
| E. Irusen, t. Jackson and a. Simjee | Asymptomatic intestinal colonization by pathogenic entamoeba histolytica in amebic liver abscess: prevalence, response to therapy, and pathogenic potential | Clinical infectious diseases | 14 (4) , 889-893 | 10.1093/clinids/14.4.889 | Different domain |
| R. Isaza and e. R. Jacobson | Antimicrobial drug use in reptiles | Antimicrobial therapy in veterinary medicine | 623-636 | 10.1002/9781118675014.ch37 | Book |
| D. Ishikawa, t. Sasaki, t. Osada, k. Kuwahara-arai, k. Haga, t. Shibuya, k. Hiramatsu and s. Watanabe | Changes in intestinal microbiota following combination therapy with fecal microbial transplantation and antibiotics for ulcerative colitis | Inflammatory bowel diseases | 23 (1) , 116-125 | [https://doi.org/10.1097/mib.0000000000000975](https://doi.org/10.1097/MIB.0000000000000975) | Abstract |
| S. Ito, g. Koren and t. R. Einarson | Maternal noncompliance with antibiotics during breastfeeding | Annals of pharmacotherapy | 27 (1) , 40-42 | 10.1177/106002809302700110 | Abstract |
| W. Jackson, d. Gonzalez, p. B. Smith, n. Ambalavanan, a. M. Atz, g. M. Sokol, c. D. Hornik, d. Stewart, g. Mundakel and b. B. Poindexter | Safety of sildenafil in extremely premature infants: a phase i trial | Journal of perinatology | 42 (1) , 31-36 | 10.1038/s41372-021-01261-w | Abstract |
| H. S. Jacob, i. M. Goldstein, i. Shapiro, p. R. Craddock, d. E. Hammerschmidt and g. Weissmann | Sudden blindness in acute pancreatitis: possible role of complement-induced retinal leukoembolization | Archives of internal medicine | 141 (1) , 134-136 | 10.1001/archinte.1981.00340010126025 | Abstract |
| R. Janknegt and l. G. Engels | Formulary management of eradication therapies for helicobacter pylori, | [disease management and health outcomes](https://link.springer.com/journal/40283) | 7 (1), 251–26 | 10.2165/00115677-200007050-00003 | Abstract |
| K. Janus, j. Antoszek and s. Suszycki | The effect of short-term starvation or water deprivation on caffeine pharmacokinetics in calves | Research in veterinary science | 70 (2) , 109-113 | 10.1053/rvsc.2000.0446 | Different domain |
| A. Jarrad, a. Debnath, y. Miyamoto, k. Hansford, r. Pelingon, m. Butler, t. Bains, t. Karoli, m. Blaskovich and l. Eckmann | Nitroimidazole carboxamides as antiparasitic agents targeting giardia lamblia, entamoeba histolytica and trichomonas vaginalis | European journal of medicinal chemistry | 120 (2016) , 353-362 | 10.1016/j.ejmech.2016.04.064 | Different domain |
| A. Jerzsele | Comparative veterinary pharmacokinetics | Readings in advanced pharmacokinetics–theory, methods and applications. Intech, rijeka | 179-198 |  | Book |
| Y. Jiang, h. Liu, x. Wu, c. Feng, p. Chang and x. Zhang | Correlation of ornidazole concentration in saliva and serum of healthy volunteers | journal of southern medical university | 30 (9) , 2108-2110 |  | Not accessible |
| S. Jing, q. Zhang, y. Li, h. Chang, c. Xiang, s. Han, g. Yuan, j. Fan and h. He | Identification of new drug candidates against trichomonas gallinae using high-throughput screening | International journal for parasitology: drugs and drug resistance | 23 (1) , 19-27 | <https://doi.org/10.1016/j.ijpddr.2023.08.001> | Abstract |
| V. Joag, o. Obila, p. Gajer, m. C. Scott, s. Dizzell, m. Humphrys, k. Shahabi, s. Huibner, b. Shannon and w. Tharao | Impact of standard bacterial vaginosis treatment on the genital microbiota, immune milieu, and ex vivo human immunodeficiency virus susceptibility | Clinical infectious diseases | 68 (10) , 1675-1683 | 10.1093/cid/ciy762 | Different domain |
| A. Johne and i. Roots | Clinical drug interactions with medicinal herbs | Evidence-based integrative medicine | 2 (4) , 207-228 | 10.2165/01197065-200502040-00004 | Review |
| K. D. Jones and j. A. Berkley | Severe acute malnutrition and infection | Paediatrics and international child health | 34 (1), 1-29 | 10.1179/2046904714z.000000000218 | Abstract |
| P. D. Josephy | Chemical and biological studies of the radiosensitizer misonidazole | University of british columbia, |  | 10.14288/1.0095006 | Thesis |
| L. Jourova, s. Satka, v. Frybortova, i. Zapletalova, p. Anzenbacher, e. Anzenbacherova, p. P. Hermanova, b. Drabonova, d. Srutkova and h. Kozakova | Butyrate treatment of dss-induced ulcerative colitis affects the hepatic drug metabolism in mice | Frontiers in pharmacology | 13 (1), 936-939 | 10.3389/fphar.2022.936013 | Abstract |
| C. M. Karam, p. S. Mckinnon, m. M. Neuhauser and m. J. Rybak | Outcome assessment of minimizing vancomycin monitoring and dosing adjustments | Pharmacotherapy: the journal of human pharmacology and drug therapy | 19 (3) , 257-266 | 10.1592/phco.19.4.257.30933 | Different domain |
| P. N. Karamanakos, p. Pappas, v. Boumba and m. Marselos | Increased brain serotonin rather than increased blood acetaldehyde as a common denominator behind alleged disulfiram-like reactions | International journal of toxicology | 39 (5) , 248-255 | 10.1177/1091581820918169 | Review |
| P. N. Karamanakos, p. Pappas, v. A. Boumba, c. Thomas, m. Malamas, t. Vougiouklakis and m. Marselos | Pharmaceutical agents known to produce disulfiram-like reaction: effects on hepatic ethanol metabolism and brain monoamines | International journal of toxicology | 26 (5), 423-432 | 10.1080/10915810701583010 | Different domain |
| T. Kasepalu | Effects of remote ischaemic preconditioning on organ damage and acylcarnitines’ metabolism in vascular surgery | Dissertationes medicinae universitatis tartuensis |  |  | Thesis |
| M. Kawaguchi-suzuki and b. D. Stamper | Pharmacokinetic and pharmacogenomic considerations for the medicinal use of botanicals | Crc press | 11-35 | 10.1201/9780429195983 | Book |
| I. Kazkayası and g. Telli | Evaluation of anti-inflammatory activity of metronidazole treatment on carrageenan induced paw edema in mice | | Fabad journal of pharmaceutical sciences | | --- | | 47 (2) , 175-182 | 10.55262/fabadeczacilik.1134535 | Different domain |
| J. L. Kennedy, j. C. Forrest, s. G. Young, b. Amick, m. Williams, l. James, j. Snowden, v. M. Cardenas, d. Boothe and c. Kirkpatrick | Temporal variations in seroprevalence of severe acute respiratory syndrome coronavirus 2 infections by race and ethnicity in arkansas | Open forum infectious diseases | 9 (5), 150-154 | [10.1093/ofid/ofac154](https://doi.org/10.1093/ofid/ofac154) | Abstract |
| S. Kimura | Evaluation of orally and rectally administered misoprostol in a low-dose endotoxin challenge in horses | Auburn university | 24-jan |  | Thesis |
| C. King | Rectal drug administration | Journal of equine veterinary science | 14 (10), 521-526 | 10.2165/00003088-198207040-00002 | Abstract |
| K. Kipper, c. I. Barker, j. F. Standing, m. Sharland and a. Johnston | Development of a novel multipenicillin assay and assessment of the impact of analyte degradation: lessons for scavenged sampling in antimicrobial pharmacokinetic study design | Antimicrobial agents and chemotherapy | 62 (1) , 10-17 | <https://doi.org/10.1128/aac.01540-17> | Abstract |
| A. Klimowicz, a. Nowak and s. Bielecka-grzela | Penetration of tinidazole into skin blister fluid following its oral administration | European journal of clinical pharmacology | 43 (1) , 523-526 | 10.1007/bf02285095 | Different domain |
| A. Klimowicz, a. Nowak and s. Bielecka-grzela | Comparison of plasma and skin blister fluid concentrations of two orally administered nitroimidazoles | Journal of dermatological treatment | 4 (4), 199-201 | 10.3109/09546639309089523 | Different domain |
| S. Knotek | Therapeutics and medication | Bsava library | 176-199 |  | Book |
| J.-h. Ko, j. Y. Baek, c.-i. Kang, w. J. Lee, j. Y. Lee, s. Y. Cho, y. E. Ha, s. H. Kim, d. R. Chung and k. R. Peck | Bacteremic meningitis caused by parvimonas micra in an immunocompetent host | Anaerobe | 34 (1), 161-163 | 10.1016/j.anaerobe.2015.05.004 | Abstract |
| J. A. Kolawole | Tollgates to effectiveness and safety of medicines in drug therapy, | University of jos |  |  | Thesis |
| P. Kole, a. Rao, v. Kurawattimath and s. Mandlekar | Lc–ms determination of triazolam and its hydroxy metabolites in mouse dried blood spots: application to transgenic mouse pharmacokinetic studies | Bioanalysis | 9 (13), 987-1000 | <https://doi.org/10.4155/bio-2017-0067> | Abstract |
| E. E. M. Koono | Avaliação de bioequivalência de comprimidos contendo 500 mg de tinidazol | Universidade de são paulo, |  |  | Language |
| J. J. Kores, i. A. Danish, d. T. Balasankar, d. D. A. Chelliah, d. P. Sheela, s. Edison and j. W. Jebaraj | Dft, and wave function analyses of megazol, |  |  | http://dx.doi.org/10.2139/ssrn.4659456 | Language |
| V. Koudriavtsev | Fluorescent imaging analysis of dextran leakage across large sized pores brain microdialysis probes, |  |  | Https://hdl.handle.net/10589/43521 | Language |
| T. Kovaleski, m. A. Malangoni and l. J. Wheat | Treatment of an amebic liver abscess with intravenous metronidazole | Archives of internal medicine | 141 (1), 132-134 | 10.1001/archinte.1981.00340010124024 | Different domain |
| S. Krakovka, f. Ranjbarian, l. A. Luján, a. Saura, n. B. Larsen, a. Jiménez-gonzález, a. Reggenti, h. D. Luján, s. G. Svärd and a. Hofer | Giardia intestinalis thymidine kinase is a high-affinity enzyme crucial for dna synthesis and an exploitable target for drug discovery | Journal of biological chemistry | 298 (6), 1-8 | 10.1016/j.jbc.2022.102028 | Abstract |
| D. Kristina, s. Anne, a. Karel, h. Suada, t. Petra, r. T. Lejla, k. Jasna, r.-t. Maida, b.-r. Lejla and m. Sanita | The role of population pharmacokinetic analysis in rational antibiotic therapy in neonates | Springer | 762-768 | 10.1007/978-981-10-4166-2_114 | Book |
| H. Ku | Uku haljasorg transcriptional mechanisms in thymic central tolerance, | Dissertationes medicinae universitatis tartuensis |  |  | Thesis |
| Y. Kusunoki, n. Ikarashi, y. Hayakawa, m. Ishii, r. Kon, w. Ochiai, y. Machida and k. Sugiyama | Hepatic early inflammation induces downregulation of hepatic cytochrome p450 expression and metabolic activity in the dextran sulfate sodium-induced murine colitis | European journal of pharmaceutical sciences | 54 (1), 17-27 | 10.1016/j.ejps.2013.12.019 | Abstract |
| B. A. Kuzma, d. Tu, a. Goss, f. Iliopoulos, j. B. Slade, a. Wiatrowski, a. Feizpour and c. L. Evans | Instantaneous topical drug quantification using a 3d printed microfluidic device and coherent raman imaging | Opennano | 12 (1), 100-151 | <https://doi.org/10.1016/j.onano.2023.100151> | Abstract |
| J. Labenz | Current role of acid suppressants in helicobacter pylori eradication therapy | Best practice & research clinical gastroenterology | 15 (3) , 413-431 | 10.1053/bega.2001.0188 | Abstract |
| O. Laius | Utilization of osteoporosis medicines, medication adherence and the trend in osteoporosis related hip fractures in estonia | Dissertationes medicinae universitatis tartuensis |  |  | Thesis |
| I. Lares‐asseff, m. G. Pérez, g. A. Camacho, a. R. Toledo, m. D. C. L. A. J. Guillé and m. G. Sosa | Effect of severe protein‐calorie malnutrition on the penetration kinetics of trimethoprim and sulfamethoxazole to the deep tissues of wistar rats | Journal of pharmacy and pharmacology | 55 (4) , 469-477 | 10.1211/0022357021026 | Animal |
| O. Lawal and d. Navaratnam | Causes of central vertigo | Diagnosis and treatment of vestibular disorders | 44 (237), 363-375 |  | Not accessible |
| J. Le and j. S. Bradley | Pharmacodynamic considerations and special populations: pediatrics | Springerantibiotic pharmacodynamics | 561-597 | 10.1007/978-1-4939-3323-5_22 | Book |
| J. Le and j. S. Bradley | Optimizing antibiotic drug therapy in pediatrics: current state and future needs | The journal of clinical pharmacology | 58 (1), 108-122 | 10.1002/jcph.1128 | Abstract |
| J. Le, b. Poindexter, j. E. Sullivan, m. Laughon, p. Delmore, m. Blackford, r. Yogev, l. P. James, c. Melloni and b. Harper | Comparative analysis of ampicillin plasma and dried blood spot pharmacokinetics in neonates | Therapeutic drug monitoring | 40 (1), 103-108 | 10.1097/ftd.0000000000000466 | Different domain |
| M. Le merdy, k. X. Szeto, j. Perrier, m. B. Bolger and v. Lukacova | Pbpk modeling approach to predict the behavior of drugs cleared by metabolism in pregnant subjects and fetuses | Pharmaceutics | 16 (1), 96-100 | [10.3390/pharmaceutics16010096](https://www.mdpi.com/1999-4923/16/1/96) | Abstract |
| M. Leblanc | The current status of antibiotic use in equine reproduction | Equine veterinary education | 21 (3), 156-167 | 10.2746/095777308x357621 | Abstract |
| J. H. Lee, o. K. Suh and m. G. Lee | Pharmacokinetic changes in drugs during protein-calorie malnutrition: correlation between drug metabolism and hepatic microsomal cytochrome p450 isozymes | Archives of pharmacal research | 27 (1), 693-712 | 10.1007/bf02980136 | Abstract |
| Y. R. Lee, d. Mcmahan, c. Mccall and g. K. Perry | Complicated intra-abdominal infections: the old antimicrobials and the new players | Drugs | 75 (1) , 2097-2117 | 10.1007/s40265-015-0506-7 | Review |
| D. Leitsch, m. Drinić, d. Kolarich and m. Duchêne | Down-regulation of flavin reductase and alcohol dehydrogenase-1 (adh1) in metronidazole-resistant isolates of trichomonas vaginalis | Molecular and biochemical parasitology | 183 (2), 177-183 | 10.1016/j.molbiopara.2012.03.003 | Abstract |
| T. L. Lemke | Antiparasitic agents | Foye’s principles of medicinal chemistry. 6th ed, philadelphia: lippincott williams & wilkins | 1028-1082 |  | Book |
| C. Leong and s. Zelenitsky | Treatment strategies for recurrent clostridium difficile infection | The canadian journal of hospital pharmacy | 66 (6) , 361-367 | 10.4212/cjhp.v66i6.1301 | Different domain |
| S. Leroux, m. A. Turner, c. B.-l. Guellec, h. Hill, j. N. Van den anker, g. L. Kearns, e. Jacqz-aigrain, w. Zhao, tinn and g. Consortiums | Pharmacokinetic studies in neonates: the utility of an opportunistic sampling design | Clinical pharmacokinetics | 54 (1) , 1273-1285 | 10.1007/s40262-015-0291-1 | Different domain |
| M. Li, l. Lan, s. Zhang, y. Xu, w. He, d. Xiang, d. Liu, x. Ren and c. Zhang | Il-6 downregulates hepatic carboxylesterases via nf-κb activation in dextran sulfate sodium-induced colitis | Nature communications | 99 (1), 1-11 | 10.1016/j.intimp.2021.107920 | Different domain |
| Z. Liu, x. Dai, h. Zhang, r. Shi, y. Hui, x. Jin, w. Zhang, l. Wang, q. Wang and d. Wang | Gut microbiota mediates intermittent-fasting alleviation of diabetes-induced cognitive impairment | Nature communications | 11 (1), 1-14 | 10.1038/s41467-020-14676-4 | Different domain |
| C. H. Livengood iii | Bacterial vaginosis: an overview for 2009 | Reviews in obstetrics and gynecology | 2 (1), 28-37 |  | Not accessible |
| C. H. Livengood iii, d. G. Ferris, h. C. Wiesenfeld, s. L. Hillier, d. E. Soper, p. Nyirjesy, j. Marrazzo, a. Chatwani, p. Fine and j. Sobel | Effectiveness of two tinidazole regimens in treatment of bacterial vaginosis: a randomized controlled trial | Obstetrics & gynecology | 110 (1) , 302-309 | 10.1016/s1090-798x(08)79020-9 | Abstract |
| V. G. Lj, m. F. Malinis and j. P. Meyer | Emerging role of actinomyces meyeri in brain abscesses: a case report and literature review | Idcases | 10 (1), 26-29 | 10.1016/j.idcr.2017.07.007 | Review |
| N. Lobanovskaya | The role of psa-ncam in the survival of retinal ganglion cells | Dissertationes medicinae universitatis tartuensis |  |  | Thesis |
| F. Lombardo, r. S. Obach, m. Y. Shalaeva and f. Gao | Prediction of volume of distribution values in humans for neutral and basic drugs using physicochemical measurements and plasma protein binding data | Journal of medicinal chemistry | 45 (13), 2867-2876 | 10.1021/jm0200409 | Different domain |
| J. G. Lossick | Treatment of trichomonas vaginalis infections | Springer | 801-818 | 10.1007/978-1-4612-3224-7_18 | Book |
| J. G. Lossick | Therapy of urogenital trichomoniasis | Springer | 324-341 | 10.1007/978-1-4612-3224-7_18 | Book |
| K. G. Lu | Update on antimicrobial therapy in mare reproduction | Clinical theriogenology | 5 (4) , 503-515 |  | Not accessible |
| W. Lumsden, d. Robertson, r. Heyworth and c. Harrison | Treatment failure in trichomonas vaginalis vaginitis | Genitourinary medicine | 64, 217-218 | 10.1136/sti.64.4.217 | Different domain |
| S. Lupi, g. Denamiel and m. Landoni | kinetics and pk/pd modelling of antimicrobial agents | Journal of veterniary pharmacology and theraputics | 35 (3), 103–136 | 10.1111/jvp.12006. | Different domain |
| J. J. Luykx, r. Vis, j. K. Tijdink, m. Dirckx, j. Van hecke and c. H. Vinkers | Psychotic symptoms after combined metronidazole-disulfiram use | Journal of clinical psychopharmacology | 33 (1), 136-137 | 10.1097/jcp.0b013e31827c3041 | Different domain |
| D. M roberts | The relevance of drug clearance to antibiotic dosing in critically ill patients | Current pharmaceutical biotechnology | 12 (12) , 2002-2014 | 10.2174/138920111798808374 | Review |
| H. Ma, s. Bian, p. Han, y. Li, a. Ni, r. Zhang, p. Ge, y. Wang, j. Zhao and y. Zong | Supplementation of exogenous bile acids improve antitrichomonal activity and enhance intestinal health in pigeon (columba livia) | Poultry science | 102(7) , 102722 | <https://doi.org/10.1016/j.psj.2023.102722> | Abstract |
| A. K. Maas | Considerations and conditions involving protozoal inhabitation of the reptilian gastrointestinal tract | Veterinary clinics: exotic animal practice | 17 (2) , 263-297 | <https://doi.org/10.1016/j.cvex.2014.01.008> | Abstract |
| M. Määttä | Hevosten mikrobilääkitys suomessa, |  |  |  | Language |
| C. Mackenzie | Diarrhoea in foals | Uk-vet equine | 5 (1) , 12-17 | <https://doi.org/10.12968/live.2014.19.2.128> | Abstract |
| J. T. Mader, h. S. El-zaim, j. H. Calhoun and j. Lefrock | Antibiotic activities and toxicities | Musculoskeletal infections. New york, ny: marcel dekker | 495-528 |  | Book |
| K. G. Magdesian | Antimicrobial pharmacology for the neonatal foal | Veterinary clinics: equine practice | 33 (1), 47-65 | 10.1016/j.cveq.2016.12.004 | Animal |
| V. Mahachai | Clinical pharmacokinetic considerations in gastroenterology | Springer | 129-157 | 10.1007/978-1-4613-0781-5_7 | Book |
| R. Maller, a. Frydén, k. Nordström and s. Ånséhn | Septicemia and meningitis caused by fusobacterium aquatile | Scandinavian journal of infectious diseases | 10 (2) , 146-148 | 10.3109/inf.1978.10.issue-2.09 | Different domain |
| T. Mandal, k. Yadava and n. Banerjee | Pharmacoklnetic studies of metronidazole in goat | Indian journal of pharmacology | 19 (3), 179-185 |  | Not accessible |
| D. Manna, p. K. Dutta, b. Achari and a. Lohia | A novel galacto-glycerolipid from oxalis corniculata kills entamoeba histolytica and giardia lamblia | Antimicrobial agents and chemotherapy | 54 (11) , 4825-4832 | 10.1128/aac.00546-10 | Different domain |
| P. Männistö, h. Haataja, m. Karhunen, j. Mattila, o. Koskela, a.-m. Suikkari, p. Heinonen and r. Tuimala | Konzentrationen von metronidazol und tinidazol in weiblichen genitalorganen nach intravenöser einzelinfusion und wiederholter oraler gabe | Infection | 12 (1), 197-201 | 10.1007/bf01640899 | Language |
| M. D. Markel | Prevention and management of peritonitis in horses | Veterinary clinics of north america: equine practice | 4 (1), 145-156 | 10.1016/s0749-0739(17)30655-7 | Animal |
| M. E. Marson, j. Altcheh, g. Moscatelli, s. Moroni, f. García-bournissen and g. E. Mastrantonio | Identification of n-benzylacetamide as a major component of human plasmatic metabolic profiling of benznidazole | European journal of drug metabolism and pharmacokinetics | 40, 209-217 | 10.1007/s13318-014-0195-8 | Different domain |
| M. E. Marson, f. G. Bournissen, j. Altcheh, g. Moscatelli, s. Moroni and g. E. Mastrantonio | Presence of benznidazole conjugated metabolites in urine identified by β-glucuronidase treatment | Brazilian journal of pharmaceutical sciences | 56 (1), 18-34 | <https://doi.org/10.1590/s2175-97902019000218034> | Abstract |
| N. Martini, s. Vesentini, c. Bassi, m. Falconi, r. Girelli, a. Messori and p. Pederzoli | Antibiotics secretion into pancreatic fluid | Springer | 129-139 | 10.1007/978-3-642-77418-8_11 | Book |
| H. Mashayekhi-sardoo, h. Kamali, s. Mehri, a. Sahebkar, m. Imenshahidi and a. H. Mohammadpour | Comparison of pharmacokinetic parameters of ranolazine between diabetic and non-diabetic rats | Iranian journal of basic medical sciences | 25 (7), 865-871 | [10.22038/ijbms.2022.64391.14156](https://doi.org/10.22038%2FIJBMS.2022.64391.14156) | Animal |
| T. Mathur, t. K. Barman, m. Kumar, d. Singh, r. Kumar, m. K. Khera, m. Yamada, s.-i. Inoue, d. J. Upadhyay and n. Masuda | In vitro and in vivo activities of ds-2969b, a novel gyrb inhibitor, against clostridium difficile | Antimicrobial agents and chemotherapy | 62 (400, 1-29 | 10.1128/aac.02157-17 | Different domain |
| B. T. Mayer, s. Srinivasan, t. L. Fiedler, j. M. Marrazzo, d. N. Fredricks and j. T. Schiffer | Rapid and profound shifts in the vaginal microbiota following antibiotic treatment for bacterial vaginosis | The journal of infectious diseases | 212 (5), 793-802 | 10.1093/infdis/jiv079 | Review |
| K. Mayumi, t. Akazawa, t. Kanazu, s. Ohnishi and h. Hasegawa | Successful prediction of human pharmacokinetics after oral administration by optimized physiologically based pharmacokinetics approach and permeation assay using human induced pluripotent stem cell–derived intestinal epithelial cells | Journal of pharmaceutical sciences | 109 (4), 1605-1614 | 10.1016/j.xphs.2019.12.019 | Different domain |
| S. A. Mccormack and b. M. Best | Obstetric pharmacokinetic dosing studies are urgently needed | Frontiers in pediatrics | 2 (1), 1-9 | 10.3389/fped.2014.00009 | Different domain |
| P. Mcintyre, p. F. Boreham, r. E. Phillips and r. W. Shepherd | Chemotherapy in giardiasis: clinical responses and in vitro drug sensitivity of human isolates in axenic culture | The journal of pediatrics | 108 (6) , 1005-1010 | 10.1016/s0022-3476(86)80950-7 | Different domain |
| H. C. Mckenzie iii | Diagnosis of enteritis and colitis in the horse | The equine acute abdomen | 7 (1), 376-410 | 10.1002/9781119063254.ch30 | Animal |
| C. E. Mcmurran, a. G. De la fuente, r. Penalva, o. B. Menachem-zidon, y. Dombrowski, g. A. Gonzalez, c. Zhao, f. N. Krause, a. M. Young and j. L. Griffin | The microbiota regulates inflammatory responses to toxin-induced cns demyelination but has minimal impact on remyelination | Biorxiv | 7 (1), 575-829 | Https://doi.org/10.1101/575829 | Abstract |
| F. Mégraud | Current recommendations for helicobacter pylori therapies in a world of evolving resistance | Gut microbes | 4 (6) , 541-548 | 10.4161/gmic.25930 | Review |
| S. D. Mehta | Systematic review of randomized trials of treatment of male sexual partners for improved bacteria vaginosis outcomes in women | 10.1097/olq.0b013e3182631d89 | 39 (10) , 822-830 | 10.1097/olq.0b013e3182631d89 | Review |
| W. Mendling | Trichomonaden-, chlamydien-und pilzinfektionen in der schwangerschaft | Infektionserkrankungen der schwangeren und des neugeborenen | 135-162 | 10.1007/978-3-662-07889-1_10 | Book |
| W. Mendling and w. Mendling | Ausgewählte sexuell übertragbare erkrankungen | Vaginose, vaginitis und zervizitis: mit bildteil zu vulvovaginalerkrankungen | 177-221 | 10.1007/978-3-662-10739-3_3 | Language |
| E. Merilind | Primary health care performance: impact of payment and practice-based characteristics, | Dissertationes medicinae universitatis tartuensis |  |  | Thesis |
| J. Meyer, s. Ryu, s. Pendland, t. Kanyok and l. Danziger | Brief report. In-vitro synergy of paromomycin with metronidazole alone or metronidazole plus hydroxymetronidazole against helicobacter pylori | Journal of antimicrobial chemotherapy (jac) | 43 (3), 403-406 | 10.1093/jac/43.3.403 | Abstract |
| J. Miller | Uninterpretable cerebrospinal fluid absorbance scans caused by antibiotic therapy | Annals of clinical biochemistry | 58 (6) , 572-578 | 10.1177/00045632211027612 | Different domain |
| R. L. Milsap and w. J. Jusko | Pharmacokinetics in the infant | Environmental health perspectives | 102 (11) , 107-110 | 10.1289/ehp.94102s11107 | Review |
| H. E. Mohamed, r. E. Gaafar, w. A. Ibrahim and h. M. Hassan | Modulatory effect of synbiotic and/or antibiotic on biochemical indices, gene expression and meat quality of broiler chicken challenged with clostridium perfringens | Journal of advanced veterinary research | 13 (10) , 2068-2077 |  | Not accessible |
| M. O. Mohammed, h. M. M. Alkubaisi and n. Q. Haj | A new prodrug and bioactivity evaluation of methotrexate based on chitosan | Heliyon | 6 (6), 1-7 | 10.1016/j.heliyon.2020.e04223 | Different domain |
| H. Moi, r. Erkkola, f. Jerve, g. Nelleman, b. Bymose, k. Alaksen and e. Tornqvist | Should male consorts of women with bacterial vaginosis be treated? | Sexually transmitted infections | 65 (4) , 263-268 | 10.1136/sti.65.4.263 | Review |
| J. D. Momper and b. M. Best | Clinical pharmacology of anti-infectives during pregnancy | Elsevier | 177-202 |  | Book |
| J. D. Momper, j. Bradley and b. M. Best | Population pharmacokinetics in pediatric drug development | Fundamentals of pediatric drug dosing | 83-92 | 10.1007/978-3-319-43754-5_6 | Book |
| P. Morales, b. Kerr, c. Oliva, e. Pizarro and m. Kong | Gonadotrophin-releasing hormone antagonists inhibit sperm binding to the human zona pellucida | Human reproduction | 14 (8) , 2069-2074 | 10.1093/humrep/14.8.2069 | Different domain |
| F. Morales-león, c. Von plessing-rossel, l. Villa-zapata, p. Fernández-rocca, c. Sanhueza-sanhueza, h. Bello-toledo and s. Mella-montecinos | Evaluación farmacocinética/farmacodinámica (pk/pd) de un esquema de administración oral de metronidazol en intervalo ampliado para el manejo de infecciones producidas por bacteroides fragilis | Revista chilena de infectología | 32 (2) , 135-141 | 10.4067/s0716-10182015000300001 | Different domain |
| O. Mráček | Využití pracovní stříbrné pevné elektrody při voltametrickém stanovení léčiva metronidazol, |  |  |  | Language |
| J. Mucklow | The fate of drugs in pregnancy | Clinics in obstetrics and gynaecology | 13 (2) , 161-175 | [https://doi.org/10.1016/s0306-3356(21)00005-4](https://doi.org/10.1016/S0306-3356(21)00005-4) | Abstract |
| T. Mukherjee and h. Boshoff | Nitroimidazoles for the treatment of tb: past, present and future | Future medicinal chemistry | 3 (11) , 1427-1454 | 10.4155/fmc.11.90 | Review |
| M. Müller | Resistance to 5-nitroimidazoles in pathogenic microorganisms | Springer | 133-148 | 10.1007/978-1-4684-4151-2_10 | Book |
| J. Münch | Acceptability, swallowability, and palatability of three oral placebo formulations in young children | Dissertation, düsseldorf, heinrich-heine-universität, 2022, |  |  | Thesis |
| R. C. Mundargi, s. A. Patil, s. A. Agnihotri and t. M. Aminabhavi | Development of polysaccharide-based colon targeted drug delivery systems for the treatment of amoebiasis | Drug development and industrial pharmacy | 33 (3) , 255-264 | 10.1080/03639040600897127 | Different domain |
| H. Nagar, s. Berger, b. Hammar and a. Gorea | Penetration of clindamycin and metronidazole into the appendix and peritoneal fluid in children | European journal of clinical pharmacology | 37 (2) , 209-210 | 10.1007/bf00558235 | Different domain |
| N. Nagata, d. Marriott, j. Harkness, j. T. Ellis and d. Stark | Current treatment options for dientamoeba fragilis infections | International journal for parasitology: drugs and drug resistance | 2 (1) , 204-215 | 10.1016/j.ijpddr.2012.08.002 | Review |
| M. Nahata | Variability in clinical pharmacology of drugs in children | Journal of clinical pharmacy and therapeutics | 17 (6) , 365-368 | 10.1111/j.1365-2710.1992.tb01319.x | Review |
| M. D. Nailor and j. D. Sobel | Tinidazole for bacterial vaginosis | Expert review of anti-infective therapy | 5 (3) , 343-348 | <https://doi.org/10.1586/14787210.5.3.343> | Abstract |
| M. D. Nailor and j. D. Sobel | Tinidazole for the treatment of vaginal infections | Expert opinion on investigational drugs | 16 (5), 743-751 | 10.1517/13543784.16.5.743 | Review |
| N. Nanda, r. G. Michel, g. Kurdgelashvili and k. A. Wendel | Trichomoniasis and its treatment | Expert review of anti-infective therapy | 4 (1), 125-135 | <https://doi.org/10.1586/14787210.4.1.125> | Abstract |
| S. K. Nandi, s. Bandyopadhyay, p. Das, i. Samanta, p. Mukherjee, s. Roy and b. Kundu | Understanding osteomyelitis and its treatment through local drug delivery system | Biotechnology advances | 34 (8), 1305-1317 | 10.1016/j.biotechadv.2016.09.005 | Different domain |
| S. Nduka, l. Adonu, e. Okonta and j. Okonta | Original research the influence of ginger (zingiber officinale) extract on the pharmacokinetic profile of pefloxacin, | International journal of applied research in natural products | 6 (2), 15-18. |  | Not accessible |
| M. Neely, d. Bayard, a. Desai, l. Kovanda and a. Edginton | Pharmacometric modeling and simulation is essential to pediatric clinical pharmacology | The journal of clinical pharmacology | 58 (10), 73-85 | 10.1002/jcph.1316 | Different domain |
| M. S. Nguyen | Oral health status and prevalence of temporomandibular disorders in 65–74-year-olds in vietnam | Dissertationes medicinae universitatis tartuensis |  |  | Thesis |
| D. P. Nicolau, k. B. Patel, r. Quintiliani and c. H. Nightingale | Cephalosporin-metronidazole combinations in the management of intra-abdominal infection | Diagnostic microbiology and infectious disease | 22 (1), 189-194 | 10.1016/0732-8893(95)00079-p | Different domain |
| M. L. Nigro, a. Gadano and m. Carballo | Evaluation of genetic damage induced by a nitroimidazole derivative in human lymphocytes: tinidazole (tnz) | Toxicology in vitro | 15 (3) , 209-213 | [https://doi.org/10.1016/s0887-2333(01)00010-8](https://doi.org/10.1016/S0887-2333(01)00010-8) | Abstract |
| I. Nilsson-ehle | High-performance liquid chromatography for analyses of antibiotics in biological fluids | Journal of liquid chromatography | 6 (2) , 251-293 | 10.1080/01483918308062876 | Different domain |
| T. M. Norton | Chelonian emergency and critical care | Seminars in avian and exotic pet medicine | 14 (2) , 106-130 | Https://doi.org/10.1053/j.saep.2005.04.005 | Review |
| T. M. Norton and m. C. Allender | Natural history and medical management of terrestrial and aquatic chelonians | Medical management of wildlife species: a guide for practitioners | 363-381 | https://doi.org/10.1002/9781119036708.ch28 | Book |
| A. D. Nunn | The biology of technetium based hypoxic tissue localising compounds | Springer | 19-45 |  | Book |
| P. Nyirjesy and j. R. Schwebke | Secnidazole: next-generation antimicrobial agent for bacterial vaginosis treatment | Future microbiology | 13 (5) , 507-524 | 10.2217/fmb-2017-0270 | Different domain |
| E. A. S. O’fallon | Emergency management of equid foals in the field | Veterinary clinics: equine practice | 37 (2), 407-420 | 10.1016/j.cveq.2021.04.009 | Animal |
| L. R. O'grady and e. D. Ralph | Anaerobic meningitis and bacteremia caused by fusobacterium species | American journal of diseases of children | 130 (8), 871-873 | 10.1001/archpedi.1976.02120090081015 | Review |
| K. Ojamaa | Epidemiology of gynecological cancer in estonia | Dissertationes medicinae universitatis tartuensis |  |  | Thesis |
| E. C. Oldfield iv, e. C. Oldfield iii and d. A. Johnson | Clinical update for the diagnosis and treatment of clostridium difficile infection | World journal of gastrointestinal pharmacology and therapeutics | 5 (1) , 1-26 | 10.4292/wjgpt.v5.i1.1 | Review |
| L. A. P. L. D. Oliveira | Efeito da exposição de giardia duodenalis ao metronidazol nas sequências gênicas associadas à resistência ao fármaco, |  |  |  | Language |
| M. Ongas, j. Standing, b. Ogutu, j. Waichungo, j. A. Berkley and k. Kipper | Liquid chromatography–tandem mass spectrometry for the simultaneous quantitation of ceftriaxone, metronidazole and hydroxymetronidazole in plasma from seriously ill, severely malnourished children | Wellcome open research | 43(2), 1-25 | 10.12688/wellcomeopenres.11728.1 | Different domain |
| C. O. Onyeji, s. I. Igbinoba, g. Olayiwola and a. Adehin | Insight into clinically effective herbal antimalarial products: effects on drug metabolizing enzymes and p-glycoprotein | African journal of pharmacy and pharmacology | 11 (48) , 591-613 | [https://doi.org/10.5897/ajpp2017.4870](https://doi.org/10.5897/AJPP2017.4870) | Abstract |
| C. Ordaz-pichardo, n. León-sicairos, v. I. Hernández-ramírez, p. Talamás-rohana and m. De la garza | Effect of bovine lactoferrin in a therapeutic hamster model of hepatic amoebiasis | Biochemistry and cell biology | 90 (3), 425-434 | <https://doi.org/10.1139/o11-084> | Abstract |
| T. Orsiere, m. De meo, p. Rathelot, j. Pompili, m. Galas, m. Castegnaro, p. Vanelle and g. Duménil | Implication of nitro group reduction in the mutagenic and chromosome damaging activities of 22 new 5-nitroisoquinolines by the salmonella mutagenicity test and the cytokinesis-blocked micronucleus assay | Food and chemical toxicology | 41 (2), 275-290 | [https://doi.org/10.1016/s0278-6915(02)00226-0](https://doi.org/10.1016/S0278-6915(02)00226-0) | Abstract |
| N. G. Osborne | Tinidazole | Journal of gynecologic surgery | 20 (2), 71-73 | 10.1089/1042406041422235 | Review |
| K. A. Oshikoya, h. M. Sammons and i. Choonara | A systematic review of pharmacokinetics studies in children with protein-energy malnutrition | European journal of clinical pharmacology | 66 (1), 1025-1035 | 10.1007/s00228-010-0851-0 | Review |
| K. A. Oshikoya, k. Smith, h. Sammons and i. Choonara | Decreased metabolism of 13c-caffeine via hepatic cyp1a2 in marasmus and kwashiorkor based on breath test | Journal of basic and clinical physiology and pharmacology | 26 (1) , 105-113 | 10.1515/jbcpp-2013-0081 | Different domain |
| A. Ottas | The metabolomic profiling of psoriasis, atopic dermatitis and atherosclerosis | Dissertationes medicinae universitatis tartuensis |  |  | Thesis |
| M. J. Palte | Characterization and exploitation of the glycocalyx for drug delivery | The university of wisconsin-madison, |  |  | Thesis |
| H. Pandya, h. Mulla, m. Hubbard, r. Cordell, p. S. Monks, s. Yakkundi, j. Mcelnay, a. Nunn, m. Turner and e. Consortium | Essential medicines containing ethanol elevate blood acetaldehyde concentrations in neonates | European journal of pediatrics | 175 (1), 841-847 | 10.1007/s00431-016-2714-x | Different domain |
| M. G. Papich | Antimicrobial therapy for gastrointestinal diseases | Veterinary clinics: equine practice | 19 (3), 645-663 | 10.1016/j.cveq.2003.08.009 | Review |
| A. Pastyříková | Současné možnosti léčby střevní a mimostřevní amébózy, |  |  |  | Language |
| G. N. Patel, r. Patel and h. Patel | Formulation and in-vitro evaluation of microbially triggered colon specific drug delivery using sesbania gum | E-journal of science & technology | 6 (2), 33-44 | [10.26265/e-jst.v6i2.675](https://doi.org/10.26265/e-jst.v6i2.675) | Different domain |
| T. W. Paton, w. R. Cornish, m. Arifie manuel and b. G. Hardy | Drug therapy in patients undergoing peritoneal dialysis: clinical pharmacokinetic considerations | Clinical pharmacokinetics | 10 (5) , 404-426 | 10.2165/00003088-198510050-00003 | Different domain |
| J. L. Patterson | Characterization of adherence, cytotoxicity and biofilm formation of gardnerella vaginalis | Virginia commonwealth university, |  |  | Book |
| K. Pauter, m. Szultka-młyńska and b. Buszewski | Determination and identification of antibiotic drugs and bacterial strains in biological samples | Molecules | 25 (11) , 2-42 | 10.3390/molecules25112556 | Review |
| L. Peikova, s. Balkanski and m. Georgieva | Methods for analysis of fluorinated quinolones in mixtures with nitroimidazole antibacterial drugs | Current pharmaceutical analysis | 18 (10), 968-982 | <https://doi.org/10.2174/1573412918666220921085708> | Abstract |
| L. E. Pelepenko, a. C. P. Janini, b. P. Gomes, a. De-jesus-soares and m. A. Marciano | Effects of bismuth exposure on the human kidney—a systematic review | Antibiotics | 11 (12), 1741-1748 | <https://doi.org/10.3390/antibiotics11121741> | Abstract |
| G. M. Penuliar, k. Nakada-tsukui and t. Nozaki | Phenotypic and transcriptional profiling in entamoeba histolytica reveal costs to fitness and adaptive responses associated with metronidazole resistance | Frontiers in microbiology | 6 (1), 1-17 | <https://doi.org/10.3389/fmicb.2015.00354> | Different domain |
| R. Pezzani, b. Salehi, s. Vitalini, m. Iriti, f. A. Zuñiga, j. Sharifi-rad, m. Martorell and n. Martins | Synergistic effects of plant derivatives and conventional chemotherapeutic agents: an update on the cancer perspective | Medicina | 55 (4) , 4-15 | 10.3390/medicina55040110 | Different domain |
| E. J. D. M. Pharmacokinet | Bulus adzu, collen masimirembwa, kudirat bola mustapha, roslyn thelingwani, rukaiyatu abdullahi kirim & karniyus shingu gamaniel, | Eurpeon journal of drug metabolism and pharmacokinet | 19 (3), 1-5 | 10.1007/s13318-014-0173-1 | Different domain |
| N. Pooley, l. Ghosh and p. Sharon | Up-regulation of e-selectin and intercellular adhesion molecule-1 differs between crohn's disease and ulcerative colitis | Digestive diseases and sciences | 40 (1) , 219-225 | 10.1007/bf02063969 | Different domain |
| R. Porosk | The role of oxidative stress in wolfram syndrome 1 and hypothermia | Dissertationes medicinae universitatis tartuensis |  |  | Thesis |
| A. Posyniak, s. Semeniuk, j. Żmudzki, j. Niedzielska and b. Biernacki | Tissue concentration of dimetridazole in laying hens | Food additives & contaminants | 13 (8) , 871-877 | 10.1080/02652039609374475 | Different domain |
| I. Pountos, t. Georgouli, k. Henshaw, b. Howard and p. Giannoudis | Mesenchymal stem cell physiology can be affected by antibiotics: an in vitro study | Cellular and molecular biology | 60 (4) , 1-7 |  | Not accessible |
| L. Pradelli | Topical metronidazole and clotrimazole in the treatment of vulvo-vaginal infections during pregnancy | Clinical management issues | 1 (1) , 21-29 | <https://doi.org/10.7175/cmi.v1i1.617> | Review |
| D. Preisig, d. Haid, f. J. Varum, r. Bravo, r. Alles, j. Huwyler and m. Puchkov | Drug loading into porous calcium carbonate microparticles by solvent evaporation | European journal of pharmaceutics and biopharmaceutics | 87 (3) , 548-558 | 10.1016/j.ejpb.2014.02.009 | Review |
| V. Purohit and a. K. Basu | Mutagenicity of nitroaromatic compounds | Chemical research in toxicology | 13 (8) , 673-692 | 10.1021/tx000002x | Review |
| Q. Qin, h. Qin, h. Luo, w. Wei, l. Liu and l. Li | Theoretical study of adsorption characteristics and the environmental influence for metronidazole on photocatalytic tio 2 anatase surfaces | Journal of molecular modeling | 25 (1) , 1-10 | 10.1007/s00894-019-3967-x | Review |
| P. Quartararo and s. Fiorino | Treatment of vaginal trichomoniasis with a single dose of tinidazole | Current medical research and opinion | 2 (3), 153-157 | 10.1185/03007997409113631 | Review |
| R. Rafique, m. N. Asi, m. Saqib, m. H. Hussain, a.-u.-r. Sial and a. Mushtaq | Preoperative administration effect of metronidazole, ceftriaxone sodium and their combination on stenotic index of jejunal anastomotic segment of dogs | Korean journal of veterinary research | 54 (1) , 27-30 | 10.14405/kjvr.2014.54.1.27 | Animal |
| S. L. Raidal | Equine pleuropneumonia | British veterinary journal | 151 (3) , 233-262 | Https://doi.org/10.1016/s0007-1935(95)80175-8get rights and content | Abstract |
| P. Raiti | Husbandry, diseases, and veterinary care of the bearded dragon (pogona vitticeps) | Journal of herpetological medicine and surgery | 22 (3), 117-131 | [doi.org/10.5818/1529-9651-22.3.117](https://doi.org/10.5818/1529-9651-22.3.117) | Animal |
| R. Rajakumaran, r. Chetty and m. Kumar | Emerging contaminant removal from domestic wastewater by advanced treatment technologies | Springer | 263-290 | 10.1007/978-3-030-95443-7_12 | Book |
| A. Rakitin | Metabolic effects of acute and chronic treatment with valproic acid in people with epilepsy, | Dissertationes medicinae universitatis tartuensis |  |  | Thesis |
| E. Ralph | Successful antimicrobial therapy of hepatic, intra-abdominal and intrapelvic abscesses | Canadian medical association journal | 131 (6), 605-607 |  | Not accessible |
| C. Rand, s. Stanley and n. Pusterla | Effects of intrarectally administered omeprazole paste on gastric fluid ph in healthy adult horses | The veterinary record | 169 (5) , 126-130 | 10.1136/vr.d3909 | Animal |
| R. L. Randell, d. K. Benjamin jr and r. G. Greenberg | An innovative approach to building an effective and efficient pediatric trial network | Hospital pediatrics | 12 (9) , 309-311 | <https://doi.org/10.1542/hpeds.2022-006811> | Abstract |
| V. Rastogi, p. Yadav, n. Lal, p. Rastogi, b. Singh, n. Verma and a. Verma | Mathematical prediction of pharmacokinetic parameters-an in-vitro approach for investigating pharmaceutical products for ivivc | Future journal of pharmaceutical sciences | 4 (2) , 175-184 | 10.1016/j.fjps.2018.03.001 | Different domain |
| M. Reitano, j. R. Masci and e. J. Bottone | Amebiasis: clinical and laboratory perspectives | Critical reviews in clinical laboratory sciences | 28 (5) , 357-385 | 10.3109/10408369109106869 | Review |
| Z. Ren, a. A. Bremer and a. C. Pawlyk | Drug development research in pregnant and lactating women | American journal of obstetrics and gynecology | 225 (1), 33-42 | 10.1016/j.ajog.2021.04.227 | Review |
| P. Rhemrev and f. Kosasih | Trichomonas vaginitis, and its treatment with tinidazole | Springer | 155-160 |  | Book |
| H. E. Rice, r. L. Brown, g. Gollin, m. G. Caty, j. Gilbert, m. A. Skinner, p. L. Glick and r. G. Azizkhan | Results of a pilot trial comparing prolonged intravenous antibiotics with sequential intravenous/oral antibiotics for children with perforated appendicitis | Archives of surgery | 136 (12) , 1391-1395 | 10.1001/archsurg.136.12.1391 | Different domain |
| C. Richardson | Comparison of recurrent and sporadic clostridium difficile infection and microbiological investigation of response of recurrent clostridium difficile infection to fecal microbiota transplantation | University of guelph, |  | 10.1007/978-3-663-20205-9_9 | Thesis |
| T. Ripa, l. Weström, p.-a. Mårdh and k.-e. Andersson | Concentrations of tinidazole in body fluids and tissues in gynaecological patients | Chemotherapy | 23 (4) , 227-235 | 10.1159/000221990 | Different domain |
| N. D. Rivera-chaparro, m. Cohen-wolkowiez and r. G. Greenberg | Dosing antibiotics in neonates: review of the pharmacokinetic data | Future microbiology | 12 (11) , 1001-1016 | 10.2217/fmb-2017-0058 | Review |
| J. Rivera-utrilla, g. Prados-joya, m. Sánchez-polo, m. Ferro-garcía and i. Bautista-toledo | Removal of nitroimidazole antibiotics from aqueous solution by adsorption/bioadsorption on activated carbon | Journal of hazardous materials | 170 (1), 298-305 | 10.1016/j.jhazmat.2009.04.096 | Review |
| J. Rivera-utrilla, m. Sánchez-polo and r. Ocampo-pérez | Removal of antibiotics from water by adsorption/biosorption on adsorbents from different raw materials | Adsorption processes for water treatment and purification | 5 (2), 139-204 | 10.1007/978-3-319-58136-1_6 | Review |
| M.-e. Rochon, a. Moussa and j. Autmizguine | Antibiotic considerations for necrotizing enterocolitis | Elsevier | 155-166 | [https://doi.org/10.1016/b978-0-323-54391-0.00013-8](https://doi.org/10.1016/B978-0-323-54391-0.00013-8) | Book |
| D. Röck | Untersuchung zur pharmakokinetischen interaktion zwischen budesonid und metronidazol bei gesunden freiwilligen | Universität tübingen, |  |  | Thesis |
| K. Rockwell, n. Rademacher, m. L. Osborn and j. G. Nevarez | Extravasation of contrast media after subcarapacial vessel injection in three chelonian species | Journal of zoo and wildlife medicine | 53 (2) , 402-411 | <https://doi.org/10.1638/2021-0074> | Abstract |
| V. Rodighiero | Effects of liver disease on pharmacokinetics: an update | Clinical pharmacokinetics | 37 (5) , 399-431 | 10.2165/00003088-199937050-00004 | Review |
| M. Roland | Prophylactic regimens in colorectal surgery: an open, randomized, consecutive trial on metronidazole used alone or in combination with ampicillin or doxycycline | World journal of surgery | 10 (6), 1003-1008 | 10.1007/bf01658658 | Different domain |
| M. Roland, t. Bergan, t. Bjerkeset, h. Erichsen, r. Hoel, s. Johansen, i. Liavåg, s. Reinertsen, a. Rosseland and t. Teigan | Prophylactic regimens in colorectal surgery: comparisons between metronidazole used alone or with ampicillin for one or three days | World journal of surgery | 9 (4) , 626-632 | 10.1007/bf01656069 | Different domain |
| A. S. Rosca, j. Castro, l. G. Sousa, a. França, m. Vaneechoutte and n. Cerca | In vitro interactions within a biofilm containing three species found in bacterial vaginosis (bv) support the higher antimicrobial tolerance associated with bv recurrence | Journal of antimicrobial chemotherapy | 77 (8), 2183-2190 | <https://doi.org/10.1093/jac/dkac155> | Abstract |
| J.-f. Rossignol | Cryptosporidium and giardia: treatment options and prospects for new drugs | Experimental parasitology | 124 (1), 45-53 | 10.1016/j.exppara.2009.07.005 | Review |
| M. D. Roth‐cline and r. M. Nelson | Ethical and practical considerations in conducting neonatal research | Pediatric drug development, 2nd edition | 73-82 | <https://doi.org/10.1002/9781118312087.ch07> | Abstract |
| M. Ryan | Neurotoxic effects of pharmaceutical agents i: anti-infectives, | Clinical neurotoxicology e-book | 338 |  | Book |
| S. S dhaneshwar and g. Vadnerkar | Rational design and development of colon-specific prodrugs | Current topics in medicinal chemistry | 11 (18), 2318-2345 | 10.2174/156802611797183249 | Review |
| H. Saaed and s. Muhammad | Comparison of topical metronidazole, ciprofloxacin, cimetidine, and meloxicam treatment in plaque-induced gingivitis | Sulaimani dental journal | 3 (1), 11-11 | <https://doi.org/10.17656/sdj.10059> | Abstract |
| S. A. Saganuwan | Unique pharmacokinetic and pharmacodynamic parameters of antimicrobials in goats | Intechopen, |  |  | Book |
| D. Said, l. Elsamad and y. Gohar | Validity of silver, chitosan, and curcumin nanoparticles as anti-giardia agents | Parasitology research | 111 (1), 545-554 | 10.1007/s00436-012-2866-1 | Different domain |
| P. S. Salaberria | Pharmacokinetic and in vitro sensitivity studies on ampicillin and congener prodrugs in equidae | University of glasgow (united kingdom), |  |  | Book |
| I. Salas-herrera, r. Pearson, a. Jhonston and p. Turner | Concentration of metronidazole in cenical mucus and serum after single and repeated oral doses | Journal of antimicrobial chemotherapy | 28 (2), 283-289 | <https://doi.org/10.1093/jac/28.2.283> | Different domain |
| A. Saleem, a. Qasim, h. J. O’connor and c. A. O’morain | Pylera® for the eradication of helicobacter pylori infection | Expert review of anti-infective therapy | 7 (7), 793-799 | 10.1586/eri.09.55 | Review |
| B. Salvesen, o. Leinebo and t. Bergan | Assay of metronidazole by hplc compared with microbial method | Scandinavian journal of gastroenterology | 91 (1), 31-43 |  | Not accessible |
| M. Sánchez-polo, j. López-peñalver, g. Prados-joya, m. A. Ferro-garcía and j. Rivera-utrilla | Gamma irradiation of pharmaceutical compounds, nitroimidazoles, as a new alternative for water treatment | Water research | 43 (16), 4028-4036 | <https://doi.org/10.1016/j.watres.2009.05.033> | Different domain |
| M. Sánchez-polo, j. Rivera-utrilla, g. Prados-joya, m. Ferro-garcía and i. Bautista-toledo | Removal of pharmaceutical compounds, nitroimidazoles, from waters by using the ozone/carbon system | Water research | 42 (15), 4163-4171 | 10.1016/j.watres.2008.05.034 | Review |
| L. Sander, e. V. G. Frandsen, d. Arnbjerg, k. Warrer and t. Karring | Effect of local metronidazole application on periodontal healing following guided tissue regeneration. Clinical findings | Journal of periodontology | 65 (10), 914-920 | <https://doi.org/10.1902/jop.1994.65.10.914> | Different domain |
| B. Sanderson, e. White and m. Baldson | Amine content of vaginal fluid from patients with trichomoniasis and gardnerella associated non-specific vaginitis | Sexually transmitted infections | 59 (5), 302-305 | <https://doi.org/10.1136/sti.59.5.302> | Different domain |
| M. M. Santamaría, j. J. A. Villafranca, j. Abilés, a. F. López, l. V. Rodas, b. T. Goitia and p. U. Navarro | Systematic review of drug bioavailability following gastrointestinal surgery | European journal of clinical pharmacology | 74 (1), 1531-1545 | 10.1007/s00228-018-2539-9 | Review |
| R. M. Saraiva, l. F. Portela, g. P. E. Da silveira, n. L. Da silva gomes, d. P. Pinto, a. C. D. A. Da silva, l. H. C. Sangenis, f. M. Carneiro, j. Almeida-silva and p. W. Marinho | Disulfiram repurposing in the combined chemotherapy of chagas disease: a protocol for phase i/ii clinical trial | Medicine: case reports and study protocols | 2 (7), 1-10 | 10.1097/md9.0000000000000110 | Abstract |
| R. Sarhan, g. Saad, h. Ezz eldin and m. Hetta | A novel impact of boswellia serrata on blastocystis spp. Infected mice | Parasitologists united journal | (2) 116-122 | [10.21608/puj.2019.12062.1042](https://dx.doi.org/10.21608/puj.2019.12062.1042) | Abstract |
| E. Sarkiala | Treatment of periodontitis in dogs with tinidazole | Journal of small animal practice | 34 (2), 90-94 | <https://doi.org/10.1111/j.1748-5827.1993.tb02617.x> | Different domain |
| S. Satka, v. Frybortova, i. Zapletalova, p. Anzenbacher, e. Anzenbacherova, h. Kozakova, d. Srutkova, t. Hudcovic and l. Jourova | Effect of dss-induced ulcerative colitis and butyrate on the cytochrome p450 2a5: contribution of the microbiome | International journal of molecular sciences | 23 (19), 11627 | <https://doi.org/10.3390/ijms231911627> | Abstract |
| C. Sauvey, g. Ehrenkaufer, a. Debnath and r. Abagyan | Antimalarial drug mefloquine kills both trophozoite and cyst stages of entamoeba mefloquine and entamoeba histolytica | Biorxiv | 3 (9), 50-59 | 10.1101/501999v1.abstract | Abstract |
| P. R. Sawyer, r. Brogden, r. Pinder, t. Speight and g. Avery | Tinidazole: a review of its antiprotozoal activity and therapeutic efficacy | Drugs | 11 (6), 423-440 | 10.2165/00003495-197611060-00003 | Review |
| A. Saxena, s. Verma and s. Sharma | Metronidazole nanogel: a review of its potency in the management of rosaceae skin disorder | Journal of pharmaceutical negative results | 13 (8), 5176-5185 | [https://doi.org/10.47750/pnr.2022.13.s08.679](https://doi.org/10.47750/pnr.2022.13.S08.679) | Review |
| C. L. Schell, j. H. Harris, a. A. Honkanen, m. S. Edwards and t. J. Raia jr | Report on gardnerella vaginitis | Military medicine | 157 (1), 37-40 | [doi.org/10.1093/milmed/157.1.37](https://doi.org/10.1093/milmed/157.1.37) | Abstract |
| W. Schleif, f. Hamblin, a. D. Everett, e. M. Graham, j. Cross, c. Fernald, r. Follett, b. Lopes, d. Martinez and h. Monforte | Tiny bodies, big needs: prospective biobanking of neonatal clinical remnant samples | Biopreservation and biobanking | 19 (2), 106-110 | <https://doi.org/10.1089/bio.2020.0113> | Abstract |
| H. Schraudolf | Effects of metronidazole on growth, chloroplast structure and differentiation in gametophytes of anemia phyllitidis l. Sw | Protoplasma | 113 (1), 144-149 | 10.1007/bf01282004 | Abstract |
| K. Schwemmle | Systemische antibiotikabehandlung bei peritonitis | Springer | 117-129 | 10.1007/978-3-642-69112-6_13 | Different domain |
| L. Sciascia, i. Calabrese, g. Cavallaro, m. Merli, c. Scialabba and m. L. T. Liveri | Modified montmorillonite as drug delivery agent for enhancing antibiotic therapy | Minerals | 11 (12), 1315-1320 | <https://doi.org/10.3390/min11121315> | Abstract |
| S. Scientifiques | Drug prescription in renal-impaired dogs, | [revue de médecine vétérinaire,](https://www.cabidigitallibrary.org/action/doSearch?do=Revue+de+M%C3%A9decine+V%C3%A9t%C3%A9rinaire) | 14 (11), 757-782 | 10.5555/19972205119 | Animal |
| K. Semjonov | Development of pharmaceutical quench-cooled molten and melt-electrospun solid dispersions for poorly water-soluble indomethacin | Dissertationes medicinaeuniversitatis tartuensis |  |  | Thesis |
| P. Sendi and w. Zimmerli | Antimicrobial treatment concepts for orthopaedic device-related infection | Clinical microbiology and infection | 18 (12), 1176-1184 | <https://doi.org/10.1111/1469-0691.12003> | Review |
| M. N. Sepehr, t. J. Al-musawi, e. Ghahramani, h. Kazemian and m. Zarrabi | Adsorption performance of magnesium/aluminum layered double hydroxide nanoparticles for metronidazole from aqueous solution | Arabian journal of chemistry | 10 (5), 611-623 | <https://doi.org/10.1016/j.arabjc.2016.07.003> | Different domain |
| N. Shah, o. P. Sharma, t. Mehta and a. Amin | Design of experiment approach for formulating multi-unit colon-targeted drug delivery system: in vitro and in vivo studies | Drug development and industrial pharmacy | 42 (5), 825-835 | <https://doi.org/10.3109/03639045.2015.1082581> | Review |
| H. Shemer, y. K. Kunukcu and k. G. Linden | Degradation of the pharmaceutical metronidazole via uv, fenton and photo-fenton processes | Chemosphere | 63 (2), 269-276 | <https://doi.org/10.1016/j.chemosphere.2005.07.029> | Review |
| J. Sherrard, c. Ison, j. Moody, e. Wainwright, j. Wilson and a. Sullivan | United kingdom national guideline on the management of trichomonas vaginalis 2014 | International journal of std & aids | 25 (8), 541-549 | 10.1177/0956462414525947 | Review |
| J. Sherrard, r. Pitt, k. R. Hobbs, m. Maynard, e. Cochrane, j. Wilson and c. Tipple | British association for sexual health and hiv (bashh) united kingdom national guideline on the management of trichomonas vaginalis 2021 | International journal of std & aids | 33 (8), 740-750 | 10.1177/09564624221103035 | Different domain |
| Y. H. Shishavan and m. Amjadi | A new enhanced chemiluminescence reaction based on polymer dots for the determination of metronidazole | Spectrochimica acta part a: molecular and biomolecular spectroscopy | 260(1), 1-7 | 10.1016/j.saa.2021.119992 | Different domain |
| P. Singh, e. J. Alm, j. M. Kelley, v. Cheng, m. Smith, z. Kassam, j. Nee, j. Iturrino and a. Lembo | Effect of antibiotic pretreatment on bacterial engraftment after fecal microbiota transplant (fmt) in ibs-d | Gut microbes | 14 (1), 2020067 | <https://doi.org/10.1080/19490976.2021.2020067> | Abstract |
| C. Sköld, s. Winiwarter, j. Wernevik, f. Bergström, l. Engström, r. Allen, k. Box, j. Comer, j. Mole and a. Hallberg | Presentation of a structurally diverse and commercially available drug data set for correlation and benchmarking studies | Journal of medicinal chemistry | 49 (23), 6660-6671 | 10.1021/jm0506219 | Abstract |
| L. Škvorová | Voltametrické stanovení vybraných nitroimidazolových léčiv, |  |  |  | Language |
| N. M. Slovis | Diarrhea in the neonate: is there anything really new? | Neonatology questions and controversies: infectious disease, immunology, and pharmacology | 66 (1), 201-223 | 10.5555/20210342732 | Review |
| M. J. Smith, a. Boutzoukas, j. Autmizguine, m. L. Hudak, e. Zinkhan, b. T. Bloom, g. Heresi, a. P. Lavery, s. E. Courtney and g. M. Sokol | Antibiotic safety and effectiveness in premature infants with complicated intraabdominal infections | The pediatric infectious disease journal | 40 (6), 550-555 | 10.1097/inf.0000000000003034 | Abstract |
| J. D. Sobel | Antibiotic consideration in bacterial vaginosis | Current infectious disease reports | 11 (6), 471-475 | 10.1007/s11908-009-0068-5 | Review |
| B. Söder, u. Nedlich and l. J. Jin | Longitudinal effect of non‐surgical treatment and systemic metronidazole for 1 week in smokers and non‐smokers with refractory periodontitis: a 5‐year study | Journal of periodontology | 70 (7), 761-771 | 10.1902/jop.1999.70.7.761 | Different domain |
| J. S. Solomkin | Treatment of intra‐abdominal infections | Quinolone antimicrobial agents | 4 (3), 217-225 | <https://doi.org/10.1128/9781555817817.ch12> | Abstract |
| M. Sörberg, h. Hanberger, m. Nilsson and l. E. Nilsson | Pharmacodynamic effects of antibiotics and acid pump inhibitors on helicobacter pylori | Antimicrobial agents and chemotherapy | 41 (10), 2218-2223 | 10.1128/aac.41.10.2218 | Different domain |
| M. M. Soriano and s. Johnson | Treatment of clostridium difficile infections | Infectious disease clinics | 29 (1), 93-108 | <https://doi.org/10.1016/j.idc.2014.11.005> | Abstract |
| W. T. Speck, a. B. Stein and h. S. Rosenkranz | Mutagenicity of metronidazole: presence of several active metabolites in human urine | Journal of the national cancer institute | 56 (2), 283-284 | 10.1093/jnci/56.2.283 | Different domain |
| S. L. Spurlock and e. A. Hanie | Antibiotics in the treatment of wounds | Veterinary clinics of north america: equine practice | 5 (3), 465-482 | [https://doi.org/10.1016/s0749-0739(17)30569-2](https://doi.org/10.1016/S0749-0739(17)30569-2) | Abstract |
| Stange | Therapie der entzündlichen darmerkrankungen | Praxis | 91 (47), 2029-2039 | 10.1024/0369-8394.91.47.2029 | Abstract |
| V. W. Stevens | Antibiotic exposure and risk of clostridium difficile infection: a retrospective cohort study | University of rochester, |  |  | Thesis |
| D. J. Stewart, r. Goel, m. C. Cripps, s. Huan, j. Yau and s. Verma | Multiple resistance modulators combined with carboplatin for resistant malignancies: a pilot study | Investigational new drugs | 15 (1), 267-277 | 10.1023/a:1005993705237 | Abstract |
| B. H. Stock | Hepatic drug metabolism in pregnancy | Drug metabolism and drug interactions | 5 (1), 53-81 | [doi.org/10.1515/dmdi.1984.5.1.53](https://doi.org/10.1515/DMDI.1984.5.1.53) | Abstract |
| A. Sturm | Gardnerella vaginalis in infections of the urinary tract | Journal of infection | 18 (1), 45-49 | 10.1016/s0163-4453(89)93642-6 | Different domain |
| S. Suryawati and b. Santoso | Determination of isoniazid half-life from salivary samples | International journal of clinical pharmacology, therapy, and toxicology | 24 (1), 18-22 |  | Not accessible |
| D. H. Swenson, b. H. Laster and r. L. Metzger | Synthesis and evaluation of a boronated nitroimidazole for boron neutron capture therapy | Journal of medicinal chemistry | 39 (7) , 1540-1544 | 10.1021/jm950689w | Review |
| C.-c. Szeto, p. K.-t. Li, d. W. Johnson, j. Bernardini, j. Dong, a. E. Figueiredo, y. Ito, r. Kazancioglu, t. Moraes and s. Van esch | Ispd catheter-related infection recommendations: 2017 update | Peritoneal dialysis international | 37 (2), 141-154 | 10.3747/pdi.2016.00120 | Different domain |
| S. Tabaqchali, m. Wilks and r. Thin | Gardnerella vaginalis and anaerobic bacteria in genital disease | Sexually transmitted infections | 59 (2), 111-115 | 10.1136/sti.59.2.111 | Different domain |
| M. A. Tabari, b. Poźniak, a. Abrishami, a. A. Moradpour, m. H. Shahavi, s. Kazemi and m. R. Youssefi | Antitrichomonal activity of metronidazole-loaded lactoferrin nanoparticles in pigeon trichomoniasis | Parasitology research | 120 (9), 3263-3272 | 10.1007/s00436-021-07263-z | Animal |
| E. A. E.-a. M. Tahoun | Protective effect of moringa oleifera against metronidazole-induced toxicity in male albino rats | Journal of bioscience and applied research | 3 (2), 137-149 | 10.2356-9174,2356-9182 | Animal |
| S. Taj, m. Zuber, v. B. Hanumanthaiah, r. Venkataraman, s. K. Puttegowda, s. Afrid and s. Kiran | Metronidazole induced cutaneous adverse drug reaction-a systematic review of descriptive studies | Current reviews in clinical and experimental pharmacology formerly current clinical pharmacology | 19 (3), 269-284 | <https://doi.org/10.2174/2772432819666230601155545> | Abstract |
| R. Tamme | Associations between pubertal hormones and physical activity levels, and subsequent bone mineral characteristics, | Dissertationes medicinae universitatis tartuensis |  |  | Thesis |
| K. Tekes, h. Kalasz, m. Y hasan, e. Adeghate, f. Darvas, n. Ram and a. Adem | Aliphatic and aromatic oxidations, epoxidation and s-oxidation of prodrugs that yield active drug metabolites | Current medicinal chemistry | 18 (32), 4885-4900 | 10.2174/092986711797535227 | Review |
| P. Teotia and n. Dwivedi | In silico identification and optimization of natural inhibitors for drug target sites in cryptosporidium parvum: a review | International journal | 4 (4), 644-652 |  | Not accessible |
| H. Thabe and h. Thabe | Surgical problems—general requirements | The rheumatoid hip | 4 (1), 69-88 | 10.1007/978-3-642-75887-4_4 | Review |
| Theuretzbacher, ursula zeitlinger, markus | Antibacterial distribution and drug–drug interactions in cancer patients | Principles and practice of cancer infectious diseases | 3 (2), 443-454 | 10.1007/978-1-60761-644-3_38 | Abstract |
| P. Thangaraju, h. Velmurugan and k. Neelambaran | Current status of pharmacokinetic research in children: a systematic review of clinical trial records | Current reviews in clinical and experimental pharmacology formerly current clinical pharmacology | 19 (1), 78-92 | <https://doi.org/10.2174/2772432818666221223155455> | Abstract |
| J. Tiigimäe-saar | Botulinum neurotoxin type a treatment for sialorrhea in central nervous system diseases | Dissertationes medicinae universitatis tartuensis |  |  | Thesis |
| J. Tillonen | Ethanol, acetaldehyde and gastrointestinal flora | Helsingin yliopisto |  |  | Thesis |
| J. Tillonen, s. Väkeväinen, v. Salaspuro, y. Zhang, m. Rautio, h. Jousimies‐somer, k. Lindros and m. Salaspuro | Metronidazole increases intracolonic but not peripheral blood acetaldehyde in chronic ethanol‐treated rats | Alcoholism: clinical and experimental research | 24 (4), 570-575 | <https://doi.org/10.1111/j.1530-0277.2000.tb02026.x> | Animal |
| P. Todd, k. Goa and h. Langtry | Antimicrobial agents | Drug prescribing in renal failure: dosing guidelines for adults and children | 21(1), 205-213 |  | Not accessible |
| M. F. Tomasello, c. Nardon, v. Lanza, g. Di natale, n. Pettenuzzo, s. Salmaso, d. Milardi, p. Caliceti, g. Pappalardo and d. Fregona | New comprehensive studies of a gold (iii) dithiocarbamate complex with proven anticancer properties: aqueous dissolution with cyclodextrins, pharmacokinetics and upstream inhibition of the ubiquitin-proteasome pathway | European journal of medicinal chemistry | 138 (1), 115-127 | 10.1016/j.ejmech.2017.06.013 | Review |
| K. Toome | Homing peptides for targeting of brain diseases | Dissertationes medicinae universitatis tartuensis | 7-135 |  | Thesis |
| R. Trachea | Conference of indian pharmacological society december 29-31, 1991, ahmedabad | Indian journal of pharmacology | 24 (1), 38-67 |  | Different domain |
| D. Triger | Liver disorders: miscellaneous papers | Current opinion in gastroenterology | 4 (4), 631-646 |  | Abstract |
| J. Trimbos | Security of various knots commonly used in surgical practice | Obstetrics & gynecology | 64 (2), 274-280 |  | Abstract |
| P. Trivalairat, k. Trivalairat, a. Tassamakorn and w. Purivirojkul | Blood recovery of wild mekong snail-eating turtles (malayemys subtrijuga schlegel and müller, 1845) in captivity from leech infestation | International journal for parasitology: parasites and wildlife | 22 (1), 126-135 | <https://doi.org/10.1016/j.ijppaw.2023.10.001> | Abstract |
| M. Trocha, a. Merwid-ląd, d. Ksiądzyna, m. Szandruk and a. Szeląg | Impact of malnutrition on drugs' action | Gastroenterologia polska/gastroenterology | 17 (1) , 11-16 |  | Not accessible |
| T.-h. Tsai | Assaying protein-unbound drugs using microdialysis techniques | Handbook of behavioral neuroscience | 16 (2006), 573-587 | [https://doi.org/10.1016/s1569-7339(06)16030-0](https://doi.org/10.1016/S1569-7339(06)16030-0) | Abstract |
| F. C. Tulunay and m. Orme | European collaboration: towards drug developement and rational drug therapy: proceedings of the sixth congress of the european association for clinical pharmacology and therapeutics istanbul, | Springer science & business media, | 24–28 |  | Book |
| N.-t. Tung, t.-h. Nguyen, t.-t. Pham and t.-q. Nguyen | Pectin/hpmc dry powder coating formulations for colon specific targeting tablets of metronidazole | Journal of drug delivery science and technology | 33 (1), 19-27 | <https://doi.org/10.1016/j.jddst.2016.03.004> | Abstract |
| M. A. Turner | Neonatal drug development | Early human development | 87 (11) , 763-768 | Https://doi.org/10.1016/j.earlhumdev.2011.08.014 | Review |
| K. Turnheim | Pharmacokinetic dosage guidelines for elderly subjects | Expert opinion on drug metabolism & toxicology | 1 (1) , 33-48 | <https://doi.org/10.1517/17425255.1.1.33> | Review |
| M. B. Uddin and s. F. Rabby | Antibiotics prescribing in labor and delivery patients in a tertiary care hospital: bangladesh perspective | International journal of pharma sciences and research | 6, 1412-1417 |  | Not accessible |
| J. A. Upcroft and p. Upcroft | Drug susceptibility testing of anaerobic protozoa | Antimicrobial agents and chemotherapy | 45 (6), 1810-1814 | Https://doi.org/10.1128/aac.45.6.1810-1814.2001 | Different domain |
| P. Upcroft and j. A. Upcroft | Drug targets and mechanisms of resistance in the anaerobic protozoa | Clinical microbiology reviews | 14 (1), 150-164 | 10.1128/cmr.14.1.150-164.2001 | Review |
| R. Velarde-salcedo, l. F. Pérez-gonzález, a. S. Rodríguez-báez, f. J. Arriaga-garcía, r. D. C. Milán-segovia, s. Romano-moreno and s. E. Medellín-garibay | Model-informed precision dosing of antimicrobial drugs in pediatrics: experiences from a pilot scale program | European journal of pediatrics | 182 (9) , 4143-4152 | 10.1007/s00431-023-05103-z | Abstract |
| N. I. M. Velázquez | Modelling fate of antibiotics of human use in dutch surface water | Wageningen university | Jan-50 |  | Thesis |
| J. Vinen | antibiotics in the emergency department |  | 446-457 |  | Book |
| M. Vogel, t. Hartmann, m. Köberle, m. Treiber, i. B. Autenrieth and u. K. Schumacher | Rifampicin induces mdr1 expression in candida albicans | Journal of antimicrobial chemotherapy | 61 (3), 541-547 | <https://doi.org/10.1093/jac/dkm513> | Different domain |
| C. Voogd | On the mutagenicity of nitroimidazoles | Mutation research/reviews in genetic toxicology | 86 (3), 243-277 | <https://doi.org/10.1016/0165-1110(81)90006-3> | Abstract |
| C. Voogd, j. Van der stel and j. Jacobs | The mutagenic action of nitroimidazoles. Iii. Tinidazole, ipronidazole, panidazole and ornidazole | Mutation research/fundamental and molecular mechanisms of mutagenesis | 48 (2), 155-161 | <https://doi.org/10.1016/0027-5107(77)90155-5> | Abstract |
| T. Vougiouklakis | Pharmaceutical agents known to produce disulfiram-like reaction: effects on hepatic ethanol metabolism and brain, | Diet, nutritional status and drug metabolism | 26 (1), 423-432 | 10. 1080/10915810701583010 | Animal |
| I. Walter-sack and u. Klotz | Influence of diet and nutritional status on drug metabolism | Clinical pharmacokinetics | 31 (1) , 47-64 | 10.2165/00003088-199631010-00004 | Abstract |
| S. Wan, z. Hua, l. Sun, x. Bai and l. Liang | Biosorption of nitroimidazole antibiotics onto chemically modified porous biochar prepared by experimental design: kinetics, thermodynamics, and equilibrium analysis | Process safety and environmental protection | 104 (1), 422-435 | 10.1016/j.psep.2016.10.001 | Abstract |
| J.-s. Wang | In vivo and in vitro studies on drug metabolism and interactions involving mibefradil, isradipine, lidocaine, selegiline and metronidazole | Helsingin yliopisto, |  |  | Thesis |
| Q. Wang, t. Ren, j. Zhao, c.-h. Wong, h. E. Chan and z. Zuo | Exclusion of unsuitable cns drug candidates based on their physicochemical properties and unbound fractions in biomatrices for brain microdialysis investigations | Journal of pharmaceutical and biomedical analysis | 178 (1), 1-29 | 10.1016/j.jpba.2019.112946 | Different domain |
| S. Wanwimolruk and v. Prachayasittikul | Cytochrome p450 enzyme mediated herbal drug interactions | Excli journal | 13 (1), 347-391 |  | Review |
| J. P. Ward | Tinidazole (fasigyn)—single‐dose therapy for trichomonas vaginalis | Medical journal of australia | 2 (17) , 651-652 | 10.5694/j.1326-5377.1976.tb98933.x | Abstract |
| J. F. Warner, r. L. Perkins and l. Cordero | Metronidazole therapy of anaerobic bacteremia, meningitis, and brain abscess | Archives of internal medicine | 139 (2), 167-169 | 10.1001/archinte.1979.03630390027013 | Abstract |
| A. Weidenbach and h. Leix | Treatment of trichomonal vaginitis with a single dose of tinidazole | Current medical research and opinion | 2 (3) , 147-152 | 10.1185/03007997409113630 | Different domain |
| J.-f. Westphal and j.-m. Brogard | Drug administration in chronic liver disease | Drug safety | 17 (1), 47-73 | 10.2165/00002018-199717010-00004 | Different domain |
| N. J. White | Antiparasitic drugs in children | Clinical pharmacokinetics | 17 (1) , 138-155 | 10.2165/00003088-198900171-00010 | Abstract |
| R. H. Whitlock and c. Buckley | Botulism | Veterinary clinics of north america: equine practice | 13 (1) , 107-128 | [https://doi.org/10.1016/s0749-0739(17)30259-6](https://doi.org/10.1016/S0749-0739(17)30259-6) | Abstract |
| M. H. Wilcox | Editorial commentary: critically ill patients with clostridium difficile infection: are 2 antibiotics better than one? | Oxford university press | 61 (6), 942-944 | <https://doi.org/10.1093/cid/civ413> | Abstract |
| P. Williams and j. A. Berkley | Severe acute malnutrition update: current whoguidelines and the whoessential medicine list for children, |  | 28-feb |  | Different domain |
| R. Williamson and g. Pipkin | Does bismuth prevent antimicrobial resistance of helicobacter pylori? | Springer | 416-425 |  | Book |
| J. T. Wilson, g. L. Kearns, d. Murphy and s. J. Yaffe | Paediatric labelling requirements: implications for pharmacokinetic studies | Clinical pharmacokinetics | 26 (1) , 308-325 | 10.2165/00003088-199426040-00006 | Abstract |
| W. D. Wilson | Rational selection of antimicrobials for use in horses |  | 47, 75-93 |  | Not accessible |
| D. Wong, b. Sykes, n. M. Slovis, c. Brockus, a. M. Mccoy and b. Dunkel | Gastrointestinal disorders | Equine neonatal medicine | 10 (3), 436-494 | <https://doi.org/10.1002/9781119617228.ch17> | Abstract |
| M.-x. Wu and h.-d. Li | Hplc simultaneous determination of the four nitroimidazoles formulation | Chinese journal of pharmaceutical analysis | 28 (1) , 68-71 | 10.2008/00000028/00000001/00018 | Abstract |
| Y. T. Wu and t. H. Tsai | Microdialysis in the hepatobiliary system: monitoring drug metabolism, hepatobiliary excretion, and enterohepatic circulation | Applications of microdialysis in pharmaceutical science | 4(9), 275-294 | Doi:10.1002/9781118011294 | Abstract |
| L. C. Wunderlich, c. Davis and j. Curd | In vitro study of bacterial growth inhibition in concentrated sug-ar solutions: microbiological basis for the use of sugar in treating infected wounds. Jorge chirife,* le6n, | Antimicrobial agents and chemotherapy | 23(5), 1-3 |  | Abstract |
| C. Xiang, y. Li, s. Jing, s. Han and h. He | Trichomonas gallinae kills host cells using trogocytosis | Pathogens | 12 (8), 1008-1012 | <https://doi.org/10.3390/pathogens12081008> | Abstract |
| L. Xu, w. Li, p. Désesquelles, n.-t. Van-oanh, s. Thomas and j. Yang | A statistical model and dft study of the fragmentation mechanisms of metronidazole by advanced oxidation processes | The journal of physical chemistry a | 123 (4) , 933-942 | 10.1021/acs.jpca.8b10554 | Different domain |
| M. Xu and f.-z. Li | Microdialysis sampling technique and its application in biopharmaceutical analysis | Chinese journal of pharmaceutical analysis | 26 (7), 1030-1034 |  | Not accessible |
| A. Yago | Treatment for cutaneous leishmaniasis a comparison between the effects of metronidazole and pentavalent antimony on the movement and multiplication of leishmania promastigotes | Japanese journal of tropical medicine and hygiene | 19 (4), 371-385 | <https://doi.org/10.2149/tmh1973.19.371> | Language |
| A. Yago | Treatment of cutaneous leishmaniasis report of a case treated with metronidazole and local heat | Japanese journal of tropical medicine and hygiene | 19 (1) , 1-14 | <https://doi.org/10.2149/tmh1973.19.1> | Language |
| J. A. Yáñez, c. M. Remsberg, c. L. Sayre, m. L. Forrest and n. M. Davies | Flip-flop pharmacokinetics–delivering a reversal of disposition: challenges and opportunities during drug development | Therapeutic delivery | 2 (5), 643-672 | 10.4155/tde.11.19 | Abstract |
| S. Yoon, g. Lee, j. Yu, k. Lee, k. Lee, j. Si, h. J. You and g. Ko | Distinct changes in microbiota-mediated intestinal metabolites and immune responses induced by different antibiotics | Antibiotics | 11 (12) , 1762-1766 | [10.3390/antibiotics11121762](https://www.mdpi.com/2079-6382/11/12/1762) | Abstract |
| M. Zarezadeh, a. Saedisomeolia, m. Shekarabi, m. Khorshidi, m. R. Emami and d. J. Müller | The effect of obesity, macronutrients, fasting and nutritional status on drug-metabolizing cytochrome p450s: a systematic review of current evidence on human studies | European journal of nutrition | 60 (1), 2905-2921 | 10.1007/s00394-020-02421-y | Review |
| H.-f. Zhang | A pyrenophora tritici-repentis necrosis toxin: protein isolation and characterization and gene cloning and expression | North dakota state university, | 24-jan |  | Book |
| L. Zhang, j. Cai, j. Xiao and z. Ye | Identification of core genes and pathways between geriatric multimorbidity and renal insufficiency: potential therapeutic agents discovered using bioinformatics analysis | Bmc medical genomics | 15 (1) , 212-216 | 10.1186/s12920-022-01370-1 | Abstract |
| Y. Zhang, j. Liu, z. Wu, x. Mei, w. Zhu and a. Wang | Rapid promoting thrombus formation and fibrin cross-linked bi-doped mesoporous bioglass for hemostatic agent | Materials today chemistry | 25 (2012), 96-100 | <https://doi.org/10.1016/j.mtchem.2022.100980> | Abstract |
| Y. Zhao, m. F. Hebert and r. Venkataramanan | Basic obstetric pharmacology | Elsevier | 38 (8), 475-486 | <https://doi.org/10.1053/j.semperi.2014.08.011> | Abstract |
| Z. Zhao, y. Hou, c. Wang, z. Man, g. Sun, j. Shang and x. Cheng | Sulfite activated by in situ preparation of zero-valent iron agar composite membrane for the removal of metronidazole: synergistic effect of radical and nonradical pathways | Separation and purification technology | 334 (2014) , 125-164 | <https://doi.org/10.1016/j.seppur.2023.125964> | Abstract |
| B. F. Zohra | Etude par modélisation moléculaire des dérivés métronidazole, | Université mohamed khider de biskra |  |  | Thesis |
| J. Zoubir, n. Bougdour, w. E. Hayaoui, c. Radaa, a. Idlahcen, a. Assabbane and i. Bakas | Electrochemical detection of metronidazole using silver nanoparticle-modified carbon paste electrode | Electrocatalysis | 13 (4) , 386-401 |  | Not accessible |
| Gjerløff, christian, arnold, elisabeth | Pharmacokinetics of intravenous metronidazole in man | Acta pharmacologica et toxicologica | 51(2), 132-135 |  | Not accessible |
| Brogden, heel,speight, avery | Metronidazole in anaerobic infections: a review of its activity, pharmacokinetics and therapeutic use | Drugs | 16(1), 387-417 |  | Not accessible |
| Child, jason chen, xinhui, mistry, rakesh d, somme, stig, macbrayne, christine | Pharmacokinetic and pharmacodynamic properties of metronidazole in pediatric patients with acute appendicitis: a prospective study | Journal of the pediatric infectious diseases society | 8(4), 297-302 | 10.1093/jpids/piy040 | Review |
| Stancil, stephani, van haandel, leon, abdel-rahman, susan pearce, robin e | Development of a uplc-ms/ms method for quantitation of metronidazole and 2-hydroxy metronidazole in human plasma and its application to a pharmacokinetic study | Journal of chromatography b | 1092(1), 272-278 | 10.1016/j.jchromb.2018.06.024 | Different domain |
| Easmon, ison, kaye, timewell, dawson | Pharmacokinetics of metronidazole and its principal metabolites and their activity against gardnerella vaginalis | Sexually transmitted infections | 58(4), 246-249 | [10.1136/sti.58.4.246](https://doi.org/10.1136/sti.58.4.246) | Abstract |
| Ralph, edward d | Clinical pharmacokinetics of metronidazole | Clinical pharmacokinetics | 8(1), 43-62 |  | Not accessible |
| Britzi, m, gross, m, lavy, e, soback, s, steinman, a | Bioavailability and pharmacokinetics of metronidazole in fed and fasted horses | Journal of veterinary pharmacology and therapeutics | 33(5), 511-514 | 10.1111/j.1365-2885.2010.01171 | Animals |
| Steinman, a, gips, m, lavy, e, sinay, i, soback, s | Pharmacokinetics of metronidazole in horses after intravenous, rectal and oral administration | Journal of veterinary pharmacology and therapeutics | 23(6), 353-357 | [10.1046/j.1365-2885.2000.00294.x](https://doi.org/10.1046/j.1365-2885.2000.00294.x) | Animals |
| Martin, claudesastre, bmallet, mnbruguerolle, bbrun, jpde micco, p gouin, f | Pharmacokinetics and tissue penetration of a single 1,000-milligram, intravenous dose of metronidazole for antibiotic prophylaxis of colorectal surgery | Antimicrobial agents and chemotherapy | 35(12), 2602-2605 | 10.1128/aac.35.12.2602 | Abstract |
| Rabin, hrurtasun, rcpartington, jkoziol, dsharon, m walker, k | High-dose metronidazole: pharmacokinetics and bioavailability using an iv preparation and application of its use as a radiosensitizer1, 2, 3 | Cancer treatment reports | 64(11), 1087-1095 |  | Not accessible |
| Tabari, ma poźniak, błażejyoussefi, mrroudaki sarvandani, mr giorgi, m | Comparative pharmacokinetics of metronidazole in healthy and trichomonas gallinae infected pigeons (columba livia, var. Domestica) | British poultry science | 62(4), 485-491 | [10.1080/00071668.2021.1881043](https://doi.org/10.1080/00071668.2021.1881043) | Animals |
| Tsai, tung-hu chen, yen-fei | Pharmacokinetics of metronidazole in rat blood, brain and bile studied by microdialysis coupled to microbore liquid chromatography | Journal of chromatography | 987(1), 277-282 | 10.1016/s0021-9673(02)01454-1 | Animals |
| Sekis, ivana ramstead, kerry rishniw, mark schwark, wayne smcdonough, sean p goldstein, richard epapich, mark simpson, kenneth w | Single-dose pharmacokinetics and genotoxicity of metronidazole in cats | Journal of feline medicine and surgery | 2009(11), 60-68 | [10.1016/j.jfms.2008.06.01](https://doi.org/10.1016/j.jfms.2008.06.011) | Animals |
| Nilsson-ehle, ingrid ursing, b nilsson-ehle, peter | Liquid chromatographic assay for metronidazole and tinidazole: pharmacokinetic and metabolic studies in human subjects | Antimicrobial agents and chemotherapy | 19(5), 754-760 | 10.1128/aac.19.5.754 | Abstract |
| Bodri, michael s rambo, tracy m wagner, robert a venkataramanan, raman | Pharmacokinetics of metronidazole administered as a single oral bolus to red rat snakes, elaphe guttata | Journal of herpetological medicine and surgery | 16(1), 15-19 | [10.5818/1529-9651.16.1.15](https://doi.org/10.5818/1529-9651.16.1.15) | Animals |
| Mustapha, kudirat bola bakare-odunola, moji t magaji, garba obodozie-ofoegbu, obiageri o akumka, david d | Pharmacokinetics of chloroquine and metronidazole in rats | Journal of applied pharmaceutical science | 5(8), 090-094 | 10.7324/japs.2015.50814 | Animals |
| Kolmstetter, christine m cox, sherry ramsay, edward c | Pharmacokinetics of metronidazole in the yellow rat snake, elaphe obsoleta quadrivitatta | Journal of herpetological medicine and surgery | 11(2), 4-8 | 10.5818/1529-9651.11.2.4 | Animals |
| Swain, ea magdesian, kg kass, ph edman, je knych, hk | Pharmacokinetics of metronidazole in foals: influence of age within the neonatal period | Journal of veterinary pharmacology and therapeutics | 38(3), 227-234 | [10.1111/jvp.12164](https://doi.org/10.1111/jvp.12164) | Animals |
| Al-dabagh, ii mohammad, fk | Pharmacokinetics and distribution of metronidazole administered intraperitoneally in mice | Pharmacology | 2008(3), 858-863 |  | Not accessible |
| Yu-hsing, tu youmin, wang allen jr, loyd v albers, donald d gorgin, mousissian k | Pharmacokinetics of metronidazole administered intravenously to male rats | International journal of pharmaceutics | 61(1), 119-125 | [10.1016/0378-5173(90)90050-e](https://doi.org/10.1016/0378-5173(90)90050-E) | Animals |
| Kreeft, jh ogilvie, ri dufresne, lr | Metronidazole kinetics in dialysis patients | Surgery | 93(1), 149-153 | 10.5555/uri:pii:0039606083902921 | Abstract |
| Kolmstetter, christine m frazier, donita cox, sherry ramsay, edward c | Pharmacokinetics of metronidazole in the green iguana, iguana iguana | Bulletin of the association of reptilian and amphibian veterinarians | 8(3), 4-7 | 10.5818/1076-3139.8.3.4 | Animals |
| Rosenblatt, jon e edson, randall s | Metronidazole | Mayo clinic proceedings | 62(11), 1013-1017 | 10.1016/s0025-6196(12)65074-5 | Book |
| Turgut, evren h özyazici, mine | Bioavailability file: metronidazole | Fabad journal of pharmaceutical sciences | 29(1), 39-49 |  | Review |
|  |  |  |  |  |  |
| Lau, alan h lam, nancy p piscitelli, stephen c wilkes, linda danziger, larry h | Clinical pharmacokinetics of metronidazole and other nitroimidazole anti-infectives | Clinical pharmacokinetics | 23(5) 328-364 | i 0-0328/$18. 50/0 | Review |
| Ali, bh charles, bg al-yousif, m bashir, ak alhadrami, g | Comparative pharmacokinetics of metronidazole in camels, sheep and goats | Acta veterinaria brno | 72(1), 49-53 |  | Animals |
| Monro, am | Blood levels of chemotherapentic drugs and the pharmacokinetics of tinidazole and metronidazole | Current medical research and opinion | 2(3), 130-137 | 10.1185/03007997409113627 | Not accessible |
| Kuroda, taisuke nagata, shun-ichi tamura, norihisa kinoshita, yuta niwa, hidekazu mita, hiroshi minami, takuto fukuda, kentaro | Single-dose pharmacokinetics of orally administered metronidazole and intravenously administered imipenem in healthy horses and computer-based simulation of pleural fluid concentrations with multiple dosing | American journal of veterinary research | 81(10), 783-789 | [10.2460/ajvr.81.10.783](https://doi.org/10.2460/ajvr.81.10.783) | Animals |
| Ezzeldin, essam | Pharmacodynamic and pharmacokinetics of metronidazole in protein malnourished rats | African journal of pharmacy and pharmacology | 6(43), 2982-2993 | 10.5897/ajpp12.374 | Animals |
| Zhang, shuqi fang, mengna zhang, qi li, xiaoting zhang, tianhong | Evaluating the bioequivalence of metronidazole tablets and analyzing the effect of in vitro dissolution on in vivo absorption based on pbpk modeling | Drug development and industrial pharmacy | 45(10), 1646-1653 | 10.1080/03639045.2019.1648502 | Different domain |
| Elewski, boni e | Percutaneous absorption kinetics of topical metronidazole formulations in vitro in the human cadaver skin model | Advances in therapy | 24(1), 239-246 |  | Not accessible |
| Tally, francis p sullivan, c edmond | Metronidazole: in vitro activity, pharmacology and efficacy in anaerobic bacterial infections | Pharmacotherapy: the journal of human pharmacology and drug therapy | 1(1), 28-38 | 10.1002/j.1875-9114.1981.tb03551.x | Review |
| Agudelo, m vesga, o | Therapeutic equivalence requires pharmaceutical, pharmacokinetic, and pharmacodynamic identities: true bioequivalence of a generic product of intravenous metronidazole | Antimicrobial agents and chemotherapy | 56(5), 2659-2665 | 10.1128/aac.06012-11 | Animals |
| Roux, annie f moirot, emmanuel delhotal, brigitte leroy, jacques a bonmarchand, guy p humbert, guy flouvat, bernard | Clinical pharmacology & therapeutics | Clinical pharmacology & therapeutics | 36(3), 363-368 | 10.1038/clpt.1984.188 | Abstract |
| Noguchi, y tanaka, t | Aspects of the pharmacology and pharmacokinetics of nitroimidazoles with special reference to tinidazole | Drugs | 15(1), 10-15 |  | Not accessible |
| Li, hui, zheng, xiaoxiang, zhang, shaomin | Pharmacokinetics of metronidazole colon-targeted capsules in dogs | 2010 4th international conference on bioinformatics and biomedical engineering | 2010(1),1-4 | 10.1109/icbbe.2010.5515920 | Animals |
| Asín-prieto, eduardosoraluce, amaia trocóniz, iñaki f cimarras, eugenia campo de ugarte sobrón, jaione sáenz rodríguez-gascón, alicia isla, arantxazu | Population pharmacokinetic models for cefuroxime and metronidazole used in combination as prophylactic agents in colorectal surgery: model-based evaluation of standard dosing regimens | International journal of antimicrobial agents | 45(5), 504-511 | 10.1016/j.ijantimicag.2015.01.008 | Different domain |
| Loft, steffen poulsen, henrik e sonne, jesper døssing, martin | Metronidazole clearance: a one‐sample method and influencing factors | Clinical pharmacology & therapeutics | 43(4), 420-428 | 10.1038/clpt.1988.53 | Different domain |
| Hong, zhang yuan-qiang, liu | Determination and study on pharmacokinetics of metronidazole and tinidazole in human breast milk | Analysis and testing technology and instruments | 6(2), 112-114 |  | Language |
| Tan, shin yee kan, elaine lim, wei yin chay, grace law, jason hk soo, gian wan bukhari, nadeem irfan segarra, ignacio | Metronidazole leads to enhanced uptake of imatinib in brain, liver and kidney without affecting its plasma pharmacokinetics in mice | Journal of pharmacy and pharmacology | 63(7), 918-925 | 10.1111/j.2042-7158.2011.01296.x | Animals |
| Kuzma, benjamin a senemar, sharareh ramezanli, tannaz ghosh, priyanka raney, sam g stagni, grazia | The dose-duration effect on cutaneous pharmacokinetics of metronidazole from topical dermatological formulations in yucatan mini-pigs | European journal of pharmaceutics and biopharmaceutics | 175(1), 43-52 | 10.1016/j.ejpb.2022.05.001 | Animals |
| Wood, ba, faulkner, jk, monro, am | The pharmacokinetics, metabolism and tissue distribution of tinidazole | Journal of antimicrobial chemotherapy | 10(1), 43-57 | 10.1093/jac/10.suppl_a.43 | Different domain |
| Neff‐davis, carol a davis, lloyd e gillette, edward l | Metronidazole: a method for its determination in biological fluids and its disposition kinetics in the dog | Journal of veterinary pharmacology and therapeutics | 4(2), 121-127 | Doi.org/10.1111/j.1365-2885.1981.tb00720.x | Animals |
| Schwab, matthias klotz, ulrich | Pharmacokinetic considerations in the treatment of inflammatory bowel disease | Clinical pharmacokinetics | 40(10), 723-751 | 01/0010-0723/$22.00/0 | Review |
| Simms-cendan, judith s | Metronidazole | Primary care update for ob/gyns | 3(5), 153-156 | 10.1016/1068-607x(96)00016-9 | Review |
| Dingsdag, simon a hunter, neil | Metronidazole: an update on metabolism, structure–cytotoxicity and resistance mechanisms | Journal of antimicrobial chemotherapy | 73(2), 265-279 | 10.1093/jac/dkx351 | Review |
| Cybulski, w larsson, pia tjälve, hans kowalska‐pylka, h sylla, m semeniuk, s | Disposition of metronidazole in hens (gallus gallus) and quails (coturnix coturnix japonica): pharmacokinetics and whole‐body autoradiography | Journal of veterinary pharmacology and therapeutics | 19(5), 352-358 | 10.1111/j.1365-2885.1996.tb00063.x | Animals |
| Frasca, denis dahyot-fizelier, claire adier, christophe mimoz, olivier debaene, bertrand couet, william marchand, sandrine | Metronidazole and hydroxymetronidazole central nervous system distribution: 1. Microdialysis assessment of brain extracellular fluid concentrations in patients with acute brain injury | Antimicrobial agents and chemotherapy | 58(2), 1019-1023 | 10.1128/aac.01760-13 | Abstract |
| Pargal, a rao, c bhopale, kk pradhan, ks masani, kb kaul, cl | Comparative pharmacokinetics and amoebicidal activity of metronidazole and satranidazole in the golden hamster, mesocricetus auratus | Journal of antimicrobial chemotherapy | 32(3), 483-489 | Doi.org/10.1093/jac/32.3.483 | Animals |
| Preisig, daniel varum, felipe bravo, roberto hartig, claudia spleiss, johannes abbes, sonia caobelli, federico wild, damian puchkov, maxim huwyler, jörg | Colonic delivery of metronidazole-loaded capsules for local treatment of bacterial infections: a clinical pharmacoscintigraphy study | European journal of pharmaceutics and biopharmaceutics | 165(1), 22-30 | 10.1016/j.ejpb.2021.05.002 | Abstract |
| Dannelley, jennifer f martin, erin m chaaban, hala miller, jamie l | Review of metronidazole dosing in preterm neonates | American journal of perinatology | 34(9), 833-838 | 10.1055/s-0037-1599822 | Review |
| Da silva neto, mjj mackay, g agaram, r macleod, m watson, dg thomson, ah | Evaluation of amoxicillin, metronidazole and gentamicin dosage regimens for use in antibiotic prophylaxis in colorectal surgery | Journal of antimicrobial chemotherapy | 76(12), 3212-3219 | 10.1093/jac/dkab337 | Abstract |
| Soares, guilherme augusto rodrigues, gustavo serafim buranello, lais pereira de oliveira, ricardo brandt de arruda miranda, josé ricardo | Pharmacomagnetography assessment of the prokinetic effect on metronidazole absorption | Journal of pharmacy and pharmacology | 75(12), 1560-1568 | 10.1093/jpp/rgad088 | Abstract |
| Sobel, ryan sobel, jack d | Metronidazole for the treatment of vaginal infections | Expert opinion on pharmacotherapy | 16(7), 1109-1115 | 10.1517/14656566.2015.1035255 | Review |
| Kasten, mary jo | Clindamycin, metronidazole, and chloramphenicol | Mayo clinic proceedings | 74(8), 825-833 | 10.4065/74.8.825 | Abstract |
| Frasca, denisdahyot-fizelier, claire adier, christophe mimoz, olivier debaene, bertrand couet, william marchand, sandrine | Metronidazole and hydroxymetronidazole central nervous system distribution: 2. Cerebrospinal fluid concentration measurements in patients with external ventricular drain | Antimicrobial agents and chemotherapy | 58(2), 1024-1027 | [10.1128/aac.01762-13](https://doi.org/10.1128/aac.01762-13) | Abstract |
| Kiang, tony kl häfeli, urs o ensom, mary hh | A comprehensive review on the pharmacokinetics of antibiotics in interstitial fluid spaces in humans: implications on dosing and clinical pharmacokinetic monitoring | Clinical pharmacokinetics | 53(1), 695-730 | 10.1007/s40262-014-0152-3 | Review |
| Bendesky, andrés menéndez, daniel ostrosky-wegman, patricia | Is metronidazole carcinogenic? | Mutation research/reviews in mutation research | 511(2), 133-144 | 10.1016/s1383-5742(02)00007-8 | Review |
| Jackson, eric a cardoni, alex a stranz, marc h bradley, wayne e | New drug evaluations: metronidazole (flagyl iv®, searle) | Drug intelligence & clinical pharmacy | 15(11), 838-846 | 10.1177/106002808101501101 | Different domain |
| Rediguieri, camila f porta, valentina nunes, diana sg nunes, taina m junginger, hans e kopp, sabine midha, kamal k shah, vinod p | Biowaiver monographs for immediate release solid oral dosage forms: metronidazole | Journal of pharmaceutical sciences | 100(5), 1618-1627 | 10.1002/jps.22409 | Review |
| Chris jensen, j gugler, roland | Interaction between metronidazole and drugs eliminated by oxidative metabolism | Clinical pharmacology & therapeutics | 37(4), 407-410 | [10.1038/clpt.1985.63](https://doi.org/10.1038/clpt.1985.63) | Different domain |
| Sattar, masankey, mg cawley, mi kaye, cm holt, je | The penetration of metronidazole into synovial fluid | Postgraduate medical journal | 58(675), 20-24 | 10.1136/pgmj.58.675.20 | Review |
| Pearce, robin e cohen-wolkowiez, michael sampson, mario r kearns, gregory l | The role of human cytochrome p450 enzymes in the formation of 2-hydroxymetronidazole: cyp2a6 is the high affinity (low km) catalyst | Drug metabolism and disposition | 41(9), 1686-1694 | 10.1124/dmd.113.052548 | Different domain |
| Kurian, maria ganapathy, dhanraj jain, ashish r | Recent advances of metronidazole-a review | Drug invention today | 10(6), 3536-3541 |  | Review |
| Adegboye, ta itiola, io | Physical and release properties of metronidazole suppositories | Tropical journal of pharmaceutical research | 7(1), 887-896 | 10.4314/tjpr.v7i1.14673 | Different domain |
| Ofoefule, sabinus i ibezim, emmanuel c esimone, okechukwu c pepple, miriam n njoku, chinedu n orisakwe, ebere o | Bioavailability of metronidazole in rabbits after administration of a rectal suppository | American journal of therapeutics | 11(3), 190-193 |  | Not accessible |
| Burda, anthony m fischbein, connie b howe, tina sigg, todd r wahl, michael a | Hemodialysis clearance of metronidazole following overdose | Annals of pharmacotherapy | 39(7-8), 1366-1366 | 10.1345/aph.1g052 | Abstract |
| Hall, p kaye, cm mcintosh, n steele, j | Intravenous metronidazole in the newborn | Archives of disease in childhood | 58(7), 529-531 | 10.1136/adc.58.7.529 | Abstract |
| Bakare-odunola, mt mustapha, kb garba, m obodozie, oo enemali, is | The influence of nifadin®, niprisan® and niprd/92/001/1-1 (am-1) on the pharmacokinetics of metronidazole in rats | European journal of drug metabolism and pharmacokinetics | 35(1), 55-58 | 10.1007/s13318-010-0008-7 | Animals |
| Guillé, beatriz e pérez alvarez, fernando villegaslópez, alejandra r toledo bravo-luna, miguel a jiménez soriano-rosales, rosa e lares-asseff, ismael arrellin, gerardo guillé, maria g pérez | Effects of mesocaval shunt on the pharmacokinetics of metronidazole in young rats | Proceedings-western pharmacology society | 48(1), 65-69 |  | Not accessible |
| Martin, claude bruguerolle, b mallet, mn condomines, m sastre, b gouin, f | Pharmacokinetics and tissue penetration of a single dose of ornidazole (1,000 milligrams intravenously) for antibiotic prophylaxis in colorectal surgery | Antimicrobial agents and chemotherapy | 34(10), 1921-1924 | 10.1128/aac.34.10.1921 | Different domain |
| García ortiz, patricia hansen, steen honoré shah, vinod p menné, torkil benfeldt, eva | The effect of irritant dermatitis on cutaneous bioavailability of a metronidazole formulation, investigated by microdialysis and dermatopharmacokinetic method | Contact dermatitis | 59(1), 23-30 | 10.1111/j.1600-0536.2008.01348.x | Different domain |
| Schwebke, jane r | Metronidazole: utilization in the obstetric and gynecologic patient | Sexually transmitted diseases | 22(6), 370-376 |  | Abstract |
| Lewis, russell e klepser, michael e ernst, erika j snabes, michael a jones, rn | Comparison of oral immediate-release (ir) and extended-release (er) metronidazole bactericidal activity against bacteroides spp. Using an in vitro model of infection | Diagnostic microbiology and infectious disease | 37(1), 51-55 | 10.1016/s0732-8893(00)00120-6 | Different domain |
| Commander, sarah jane benjamin, daniel k wu, huali thompson, elizabeth j lane, morgan clark, reese h greenberg, rachel g hornik, christoph p | Exposure-response relationships of metronidazole in infants: integration of electronic health record data with population pharmacokinetic modeling-derived exposure simulation | The pediatric infectious disease journal | 42(1), 27-31 | 10.1097/inf.0000000000003726 | Abstract |
| Wang, laura a gonzalez, daniel leeder, j steven tyndale, rachel f pearce, robin e benjamin jr, daniel kl | Metronidazole metabolism in neonates and the interplay between ontogeny and genetic variation | Journal of clinical pharmacology | 57(2), 230-234 | 10.1002/jcph.797 | Abstract |
| Shakir, l javeed, a ashraf, m riaz, a | Metronidazole and the immune system | Die pharmazie-an international journal of pharmaceutical sciences | 66(6), 393-398 | 10.1691/ph.2011.0790 | Abstract |
| Ravari, hassan jangjoo, ali motamedifar, jalal moazzami, kasra | Oral metronidazole as antibiotic prophylaxis for patients with nonperforated appendicitis | Clinical and experimental gastroenterology | 4(1), 273-276 | 10.2147/ceg.s18153 | Different domain |
| El-bagary, ramzia el-zaher, asmaa ahmed elkady, ehab mandour, asmaa abdelkerim | Simultaneous determination of ciprofloxacin hydrochloride and metronidazole in spiked human plasma by ultra performance liquid chromatography-tandem mass spectroscopy | Journal of applied pharmaceutical science | 6(3), 41-47 | 10.7324/japs.2016.60307 | Different domain |
| Lebkowska wieruszewska, beata serih, firas lisowski, andrew poapolathep, amnart giorgi, mario | Comparative pharmacokinetic evaluation of metronidazole in sheep and goats | Journal of veterinary pharmacology and therapeutic | 2024(1), 20-24 | 10.2139/ssrn.4824787 | Animals |
| Pyörälä, s kotilainen, t silvennoinen, p hänninen, u mero, m kaartinen, l | Pharmacokinetics of tinidazole in the horse | Journal of veterinary pharmacology and therapeutic | 13(1), 76-80 | 10.1111/j.1365-2885.1990.tb00750.x | Animals |
| Bhavsar, sk malik, jk | Disposition kinetics of metronidazole in cow calves | Indian journal of pharmacology | 25(4), 246-248 |  | Not accessible |
| Baggot, jd wilson, wd hietala, s | Clinical pharmacokinetics of metronidazole in horses | Journal of veterinary pharmacology and therapeutic | 11(4), 417-420 | 10.1111/j.1365-2885.1988.tb00205.x | Animals |
| Lamp, kenneth lacy, melinda k freeman, collin | Metronidazole, clindamycin, and streptogramin pharmacodynamics |  | 233-258 |  | Book |
| Alper, michael m barwin, b norman mclean, william m | Systemic absorption of metronidazole by the vaginal route | Obstetrics & gynecology | 65(6),781-784 |  | Not accessible |
| Krishnaiah, ysr reddy, pr bhaskar satyanarayana, v karthikeyan, rs | Studies on the development of oral colon targeted drug delivery systems for metronidazole in the treatment of amoebiasis | International journal of pharmaceutics | 236(1-2), 43-55 | 10.1016/s0378-5173(02)00006-6 | Different domain |
| Earl, p sisson, pr ingham, hr | Twelve-hourly dosage schedule for oral and intravenous metronidazole | Journal of antimicrobial chemotherapy | 23(4), 619-621 | 10.1093/jac/23.4.619 | Different domain |
| Senemar, sharareh kuzma, benjamin a ramezanli, tannaz ghosh, priyanka raney, sam g rantou, elena stagni, grazia | Bioequivalence evaluation of topical metronidazole products using dermal microdialysis in new zealand rabbits | Aaps pharmscitech | 24(7), 200-204 |  | Not accessible |
| Adamsson, inger nord, carl erik lundquist, per sjöstedt, svante edlund, charlotta | Comparative effects of omeprazole, amoxycillin plus metronidazole versus omeprazole, clarithromycin plus metronidazole on the oral, gastric and intestinal microflora in helicobacter pylori-infected patients | Journal of antimicrobial chemotherapy | 44(5), 629-640 | 10.1093/jac/44.5.629 | Abstract |
| Wilby, kyle j gilchrist, samuel e ensom, mary hh | A review of the pharmacokinetic implications of schistosomiasis | Clinical pharmacokinetics | 52(10, 647-656 | 10.1007/s40262-013-0055-8 | Review |
| Leitsch, david | A review on metronidazole: an old warhorse in antimicrobial chemotherapy | Parasitology | 146(9), 1167-1178 | 10.1017/s0031182017002025 | Review |
| Bhavsar, sk malik, jk | Pharmacokinetics of metronidazole in calves | British veterinary journal | 150(4), 389-393 | 10.1016/s0007-1935(05)80156-3 | Animals |
| Buttar, hs | Fate of metronidazole following intra vaginal and intravenous administration to rabbits | Journal of toxicology and environmental health, part a current issues | 9(2), 305-316 | 10.1080/15287398209530163 | Animals |
| Brook, itzhak | Treatment of anaerobic infections in children with metronidazole | Developmental pharmacology and therapeutics | 6(3), 187-198 | 10.1159/000457294 | Different domain |
| Ingoldby, cjh dean, ph vowden, p giles, gr | Inappropriate use of metronidazole in gastrointestinal surgery | Alimentary pharmacology & therapeutics | 3(3), 277-284 | 10.1111/j.1365-2036.1989.tb00214.x | Different domain |
| Farid, nehal f elgendy, marwa o abdelwahab, nada s | Sustainable tlc-densitometric method for pharmacokinetic study of the concurrently used ibuprofen and metronidazole: green metric assessment | Microchemical journal | 179(1), 107-111 | 10.1016/j.microc.2022.107582 | Abstract |
| Sharma, priya gupta, jyoti | Metronidazole: an overview of the disease | Research in pharmacy and health sciences | 8(4), 197-204 | 10.32463/rphs.2022.v09i04.02 | Review |
| Dallmann, andré ince, ibrahim coboeken, katrin eissing, thomas hempel, georg | A physiologically based pharmacokinetic model for pregnant women to predict the pharmacokinetics of drugs metabolized via several enzymatic pathways | Clinical pharmacokinetics | 57(1), 749-768 | 10.1007/s40262-017-0594-5 | Different domain |
| Vick, zaria denise | Metronidazole neurotoxicity |  |  |  | Thesis |
| Bergogne-bérézin, e bryskier, a | The suppository form of antibiotic administration: pharmacokinetics and clinical application | Journal of antimicrobial chemotherapy | 43(2), 177-185 | 10.1093/jac/43.2.177 | Different domain |
| Kumanan, t sujanitha, vathulan sri ranganathan, s | Metronidazole for amoebiasis: a tale of more than half a century | Jaffna medical journal | 33(1), 6-13 | 10.4038/jmj.v33i1.116 | Review |
| Džidić-krivić, amina kusturica, jasna sher, emina karahmet selak, nejra osmančević, nejra karahmet farhat, esma sher, farooq | Effects of intestinal flora on pharmacokinetics and pharmacodynamics of drugs | Drug metabolism reviews | 55(1-2), 126-139 | 10.1080/03602532.2023.2186313 | Abstract |
| Okonta, jm uboh, m obonga, wo | Herb-drug interaction: a case study of effect of ginger on the pharmacokinetic of metronidazole in rabbit | Indian journal of pharmaceutical sciences | 70(2), 230-232 | 10.4103/0250-474x.41462 | Animals |
| Calabrese, ilaria cavallaro, gennara scialabba, cinzia licciardi, mariano merli, marcello sciascia, luciana liveri, maria liria turco | Montmorillonite nanodevices for the colon metronidazole deliver | International journal of pharmaceutics | 457(1), 224-236 | 10.1016/j.ijpharm.2013.09.017 | Different domain |
| Kolawole, jacob a ameh, innocent u | Chronopharmacokinetics of metronidazole in healthy human volunteers | Journal of pharmacy & bioresources | 1(1), 29-34 | [10.4314/jpb.v1i1.32045](https://doi.org/10.4314/jpb.v1i1.32045) | Abstract |
| Flouvat, bl imbert, c dubois, dm temperville, bp roux, af chevalier, gc humbert, g | Pharmacokinetics of tinidazole in chronic renal failure and in patients on haemodialysis | British journal of clinical pharmacology | 15(16), 735-741 | 10.1111/j.1365-2125.1983.tb01558.x | Different domain |
| Dai, yinmei zhang, hong wang, xiuhua | Pharmacokinetics of metronidazole and tinidazole in human breast and the effects on breast-fed infants | Chinese journal of perinatal medicine | 12(1), 1-6 |  | Not accessible |
| Mandal, tk yadava, kp pandey, sn banerjee, nc | Pharmacokinetics of metronidazole in buffalo calves | [indian journal of animal sciences,](https://www.cabidigitallibrary.org/action/doSearch?do=Indian+Journal+of+Animal+Sciences) | 56(6), 642-646 |  | Not accessible |
| Cohen-wolkowiez, michaelwatt, kevin m zhou, chenguang bloom, barry t poindexter, brenda castro, lisa | Developmental pharmacokinetics of piperacillin and tazobactam using plasma and dried blood spots from infants | Antimicrobial agents and chemotherapy | 58(5), 2856-2865 | 10.1128/aac.02139-13 | Abstract |
| Patel, hm bhavsar, sk thaker, am malik, jk | Pharmacokinetics of metronidazole in sheep following intravenous and oral administration | [indian veterinary journal](https://www.cabidigitallibrary.org/action/doSearch?do=Indian+Veterinary+Journal) | 71(8),771-774 |  | Animals |
| Alapati, vr chaluvadi, mr krishna, devarakonda r | Influence of rifampicin pretreatment on the pharmacokinetics of tinidazole in healthy male volunteers | Clinical drug investigation | 21(1),783-787 | 3/01/0011-0783/$22.00 | Abstract |
| Pyörälä, s silvennoinen, p hänninen, u mero, m | Pharmacokinetics of tinidazole in cows‐a preliminary study | Journal of veterinary pharmacology and therapeutics | 13(4), 425-427 |  | Not accessible |
| Da mata, neidmar jales, letícia custódio, ada isa sales, mayara maria monteiro, wenddy de lima cavalcanti lacerda da mata, raniere | Pharmacodynamics and pharmacokinetics of anti-infective agents in pregnant women | Pregnancy and anti-infective agents | 4(1), 21-44 |  | Not accessible |
| Al jalali, valentin zeitlinger, markus | Systemic and target-site pharmacokinetics of antiparasitic agents | Clinical pharmacokinetics | 59(7), 827-847 | 10.1007/s40262-020-00871-5 | Abstract |
| Li, jinhui hao, xinghui wang, chenguang iu, haiyan | Improving the solubility, dissolution, and bioavailability of metronidazole via cocrystallization with ethyl gallate | Pharmaceutics | 13(4) 545-559 | 10.1007/s40262-020-00871-5 | Abstract |
| Cohen-wolkowiez, michael benjamin jr, daniel k ross, ashley james, laura p sullivan, janice e walsh, michele c zadell, arlene newman, nancy | Population pharmacokinetics of piperacillin using scavenged samples from preterm infants | Therapeutic drug monitoring | 34(3), 312-319 | 10.1097/ftd.0b013e3182587665 | Abstract |
| Soule, ashley f green, sarah b blanchette, lisa m | Clinical efficacy of 12-h metronidazole dosing regimens in patients with anaerobic or mixed anaerobic infections | Therapeutic advances in infectious disease | 5(3), 57-62 | 10.1177/20499361187664 | Abstract |
| Westphal, jean-frederic brogard, jean-marie | Clinical pharmacokinetics of newer antibacterial agents in liver disease | Clinical pharmacokinetics | 24(1), 46-58 |  | Abstract |
| Hu, nan ling, jing dong, lulu jiang, yan zhou, qi zou, sulan | Pharmacokinetics of omeprazole in rats with dextran sulfate sodium–induced ulcerative colitis | Drug metabolism and pharmacokinetics | 35(3), 297-303 | 10.1016/j.dmpk.2020.02.002 | Animals |
| Robbie, marilyn olson sweet, richard l | Metronidazole use in obstetrics and gynecology: a review | American journal of obstetrics and gynecology | 145(7),865-881 | 10.1016/0002-9378(83)90693-2 | Review |
| Sarna, justyna r furtado, sarah brownell, a keith w | Neurologic complications of metronidazole | Canadian journal of neurological sciences | 40(6), 768-776 | 10.1017/s0317167100015870 | Review |
| Haddad, nicholas carr, maddie balian, steve lannin, james kim, yuri toth, courtney jarvis, jennifer | The blood–brain barrier and pharmacokinetic/pharmacodynamic optimization of antibiotics for the treatment of central nervous system infections in adults | Antibiotics | 11(12), 18-43 | 10.3390/antibiotics11121843 | Abstract |
| Merdjan, h baumelou, a diquet, b chick, o singlas, e | Pharmacokinetics of ornidazole in patients with renal insufficiency; influence of haemodialysis and peritoneal dialysis | British journal of clinical pharmacology | 19(2), 211-217 | 10.1111/j.1365-2125.1985.tb02633.x | Different domain |
| Karhunen, m | Placental transfer of metronidazole and tinidazole in early human pregnancy after a single infusion | British journal of clinical pharmacology | 18(2), 254-257 | 10.1111/j.1365-2125.1984.tb02465.x | Different domain |
| Miljkovic, vojkanarsic, biljana bojanic, zoran nikolic, g nikolic, lj kalicanin, biljana savic, v | Interactions of metronidazole with other medicines: a brief review | Die pharmazie-an international journal of pharmaceutical sciences | 60nine | 10.1691/ph.2014.3951 | Book |
| Momper, jeremiah d capparelli, edmund v wade, kelly c kantak, anand dhanireddy, ramasubbareddy cummings, james jnedrelow, jonathan hhudak, mark lmundakel, gratias t natarajan, girija | Population pharmacokinetics of fluconazole in premature infants with birth weights less than 750 grams | Antimicrobial agents and chemotherapy | 60(9), 5539-5545 | 10.1128/aac.00963-16 | Different domain |
| Butranova, olga i ushkalova, elena a zyryanov, sergey k chenkurov, mikhail s | Developmental pharmacokinetics of antibiotics used in neonatal icu: focus on preterm infants | Biomedicines | 11(3), 940-944 | 10.3390/biomedicines11030940 | Different domain |
| Pena, ma horga, jf zapater, p | Variations of pharmacokinetics of drugs in patients with cirrhosis | Expert review of clinical pharmacology | 9(3), 441-458 | 10.1586/17512433.2016.1135733 | Different domain |
| Greenstein, gary | The role of metronidazole in the treatment of periodontal diseases | Journal of periodontology | 64(1), 1-15 | 10.1902/jop.1993.64.1.1 | Different domain |
| O'keefe, j paul troc, kristine a thompson, kenneth d | Activity of metronidazole and its hydroxy and acid metabolites against clinical isolates of anaerobic bacteria | Antimicrobial agents and chemotherapy | 22(3), 426-430 | 10.1128/aac.22.3.426 | Abstract |
| Sadowska, a car, h rodziewicz, l cepowicz, d kedra, b | The relationship between metronidazole concentration and clinicopathological parameters in patients with colon cancer: a pilot study | Progress in health sciences | 3(2), 74-80 |  | Not accessible |
| Meyer, brigitte kornek, gabriela v nikfardjam, mariam karth, georg delle heinz, gottfried locker, gottfried j jaeger, walter thalhammer, florian | Multiple-dose pharmacokinetics of linezolid during continuous venovenous haemofiltration | Journal of antimicrobial chemotherapy | 56(1), 172-179 | 10.1093/jac/dki133 | Different domain |
| Sudesh chandra, sudesh chandra gupta, pk shashi gupta, shashi gupta vijjan, vk | Disposition kinetics and bioavailability of metronidazole in guinea fowl | Indian journal of poultry science | 32(1), 39-42 |  | Not accessible |
| Männistö, pt saijonmaa, o haataja, h | Effect of enzyme induction and inhibition on the fate of metronidazole and tinidazole in the rat | Pharmacology & toxicology | 60(1), 24-28 | 10.1111/j.1600-0773.1987.tb01714.x | Animals |
| Bennett, william m aronoff, george r morrison, gail golper, thomas a pulliam, joseph wolfson, marsha singer, irwin | Drug prescribing in renal failure: dosing guidelines for adults | American journal of kidney diseases | 3(3), 155-193 | 10.1016/s0272-6386(83)80060-2 | Animals |
| Stojanova, jana arancibia, marcelo ghimire, samiksha sandaradura, indy | Understanding the pharmacokinetics of antibiotics in pregnancy: is there a role for therapeutic drug monitoring? A narrative review | Therapeutic drug monitoring | 44(1), 50-64 | 10.1097/ftd.0000000000000950 | Review |
| Edwards, geoffrey breckenridge, alasdair m | Clinical pharmacokinetics of anthelmintic drugs | Clinical pharmacokinetics | 15(2), 67-93 | 10.2165/00003088-198815020-00001 | Abstract |
| Loft, s nielsen, aj borg, be poulsen, he | Metronidazole and antipyrine metabolism in the rat: clearance determination from one saliva sample | Xenobiotica | 21(10), 33-46 | 10.3109/00498259109039448 | Animals |
| Van hoogdalem, ewoud j de boer, albertus g breimer, douwe d | Pharmacokinetics of rectal drug administration, part ii: clinical applications of peripherally acting drugs, and conclusions | Clinical pharmacokinetics | 21(2), 110-128 | 0312.5963/ 91 /0008·0110/$09.50/0 | Different domain |
| Gustafsson, kajsa tatz, amos j dahan, roee britzi, malka soback, stefan ahmad, wiessam abu prince, hagar kelmer, gal | The concentration of metronidazole in the distal interphalangeal joint following intravenous regional limb perfusion via the cephalic vein in standing horses | Veterinary and comparative orthopaedics and traumatology | 34(4), 287-293 | 10.1055/s-0041-1726083 | Animals |
| Sweeney, raymond w sweeney, corinne r weiher, janice | Clinical use of metronidazole in horses | Journal of the american veterinary medical association | 198(6), 1045-1048 | [10.2460/javma.1991.198.06.1045](https://doi.org/10.2460/javma.1991.198.06.1045) | Animals |
| González, iliana sotelo, angela jung, helgi | Effect of malnutrition on the pharmacokinetics of cefuroxime axetil in young rats | Journal of pharmacy & pharmaceutical sciences | 11(1), 9-21 | 10.18433/j3z59s | Animals |
| Kim, dong-hyun | Gut microbiota-mediated drug-antibiotic interactions | Drug metabolism and disposition | 43(10), 1581-1589 |  | Not accessible |
| St peter, wendy l redic-kill, kimberly a halstenson, charles e | Clinical pharmacokinetics of antibiotics in patients with impaired renal function | Clinical pharmacokinetics | 22(1), 169-210 |  | Not accessible |
| Ellis, katelyn e council-troche, r mcalister von dollen, karen a beachler, theresa m bailey, c scott davis, jennifer l lyle, sara k | Pharmacokinetics of intrarectal altrenogest in horses | Journal of equine veterinary science | 72(10), 41-46 | 10.1016/j.jevs.2018.10.001 | Animals |
| Freeman, collin d klutman, neil e lamp, kenneth c | Metronidazole: a therapeutic review and update | Drugs | 54(1), 679-708 |  | Not accessible |
| Goldstein, ellie jc sutter, vera l finegold, sydney m | Comparative susceptibilities of anaerobic bacteria to metronidazole, ornidazole, and sc-28538 | Antimicrobial agents and chemotherapy | 14(4), 609-613 | 10.1128/aac.14.4.609 | Different domain |
| Saha, dibyajyoti paul, swati | Pharmacokinetics of ciprofloxacin in animals | Egyptian academic journal of biological sciences, b. Zoology | 5(1), 23-32 | [10.21608/eajbsz.2013.13508](https://doi.org/10.21608/eajbsz.2013.13508) | Animals |
| Thakkar, nilay gonzalez, daniel cohen-wolkowiez, michael massaro, mm bernhardt, janice zane, nicole r laughon, matthew m | An opportunistic study evaluating pharmacokinetics of sildenafil for the treatment of pulmonary hypertension in infants | Journal of perinatology | 36(9), 744-747 | 10.0743-8346/16 | Different domain |
| Yousefi, masoud rahmani, kouroshmjalilzadeh yengejeh, reza sabzalipour, sima goudarzi, gholamreza | Green synthesis of zero iron nanoparticles and its application in the degradation of metronidazole | Journal of health sciences & surveillance system | 9(1), 66-70 | [10.30476/jhsss.2020.88584.1146](https://doi.org/10.30476/jhsss.2020.88584.1146) | Abstract |
| Palte, michael j davis, amy kf mcgrath, nicholas a spiegel, carol a raines, ronald t | Ribonucleoside 3′-phosphates as pro-moieties for an orally administered drug | Chemmedchem | 7(8), 1361-1364 | 10.1002/cmdc.201200243 | Different domain |
| Pineda, leslie c watt, kevin m | New antibiotic dosing | Clinics in perinatology | 42(1), 167-172 | 10.1016/j.clp.2014.10.009 | Review |
| Zheng, kang li, ao wu, weiwei qian, shaosong liu, baohua pang, qiuxiang | Preparation, characterization, in vitro and in vivo evaluation of metronidazole–gallic acid cocrystal: a combined experimental and theoretical investigation | Journal of molecular structure | 1197(1), 727-735 | 10.1016/j.molstruc.2019.07.102 | Animals |
| Dash, ranjeet prasad babu, r jayachandra srinivas, nuggehally r | Eappraisal and perspectives of clinical drug–drug interaction potential of α-glucosidase inhibitors such as acarbose, voglibose and miglitol in the treatment of type 2 diabetes mellitus | Xenobiotica | 48(1), 89-108 | 10.1080/00498254.2016.1275063 | Abstract |
| Amon, i kraatz, g amon, k peters, r | Metronidazole in patients with renal insufficiency | Drugs under experimental and clinical research | 8(3), 231-234 | 10.83x0120709 | Abstract |
| Lau, a | Pharmacokinetics of metronidazole in hospitalized patients | International journal of clinical pharmacology, therapy, and toxicology | 24(12),643-645 |  | Not accessible |
| Erickson, steven h oppenheim, gene l smith, gary h | Metronidazole in breast milk | Obstetrics & gynecology | 57(1), 48-50 |  | Not accessible |
| Mudry, md martínez‐flores, i palermo, am carballo, ma | Embryolethality induced by metronidazole (mtz) in rattus norvegicus | Teratogenesis, carcinogenesis, and mutagenesis | 21(3), 197-205 | 10.1002/tcm.1008 | Animals |
| Mcneil, jj louis, wj | Clinical pharmacokinetics of labetalol | Clinical pharmacokinetics | 9(1), 157-167 |  | Abstract |
| Cudmore, sarah l delgaty, kiera l hayward-mcclelland, shannon f petrin, dino p garber, gary e | Treatment of infections caused by metronidazole-resistant trichomonas vaginalis | Clinical microbiology reviews | 17(4), 783-793 | 10.1128/cmr.17.4.783-793.2004 | Different domain |
| Qin, ya-juan wang, peng-fei makawana, jigar a wang, zhong-chang wang, ze-nan jiang, ai-qin | Design, synthesis and biological evaluation of metronidazole–thiazole derivatives as antibacterial inhibitors | Bioorganic & medicinal chemistry letters | 24(22), 5279-5283 | 10.1016/j.bmcl.2014.09.054 | Different domain |
| Bush, andrew holt, jacquelyn e sankey, michael g kaye, clive m | Penetration of metronidazole into continuous ambulatory peritoneal dialysate | Peritoneal dialysis international | 3(4), 173-174 | 10.1177/089686088300300403 | Abstract |
| Maideen, nmp | Pharmacologically relevant drug interactions of α-glucosidase inhibitors | Journal of diabetes metabolism and disorder |  | Journal of diabetes metabolism and disorder | Abstract |
| Mimura, yasuyuki yahiro, mana masumoto, miw fukui, risako okamoto, rina aichi, makoto | The pharmacokinetics of oral metronidazole in patients with metronidazole‐induced encephalopathy undergoing maintenance hemodialysis | Hemodialysis international | 24(4), 528-533 | 10.1111/hdi.12857 | Review |
| Lau, ah chang, cw sabatini, s | Hemodialysis clearance of metronidazole and its metabolites | Antimicrobial agents and chemotherapy | 29(2), 235-238 | 10.1128/aac.29.2.235 | Different domain |
| Aleanizy, fadilah sfouq alqahtani, fulwah al gohary, omaimah el tahir, eram al shalabi, rania | Determination and characterization of metronidazole–kaolin interaction | Saudi pharmaceutical journal | 23(2), 167-176 | 10.1016/j.jsps.2014.06.006 | Different domain |
| Cherian, philip t wu, xiaoqian yang, lei scarborough, jerrod s singh, aman p alam, zahidul a lee, richard e hurdle, julian g | Gastrointestinal localization of metronidazole by a lactobacilli-inspired tetramic acid motif improves treatment outcomes in the hamster model of clostridium difficile infection | Journal of antimicrobial chemotherapy | 70(11), 3061-3069 | 10.1093/jac/dkv231 | Different domain |
| Sulochana, suresh p syed, muzeeb handrasekar, devaraj v | Clinical drug–drug pharmacokinetic interaction potential of sucralfate with other drugs: review and perspectives | Veterinary and comparative orthopaedics and traumatology | 41(2), 469-503 |  | Not accessible |
| Schouwenburg, stef wildschut, enno d de hoog, m | The pharmacokinetics of beta-lactam antibiotics using scavenged samples in pediatric intensive care patients: the expat kids study protocol | Frontiers in pharmacology | 12(2), 75-80 | 10.3389/fphar.2021.750080 | Review |
| Brooks, jason v furney, synthia k orme, ian m | Metronidazole therapy in mice infected with tuberculosis | Antimicrobial agents and chemotherapy | 43(50, 1285-1288 | 10.1128/aac.43.5.1285 | Animals |
| Ogata, h aoyagi, n kaniwa, n shibazaki, t ejima, e takagishi, y ogura, t | Biavailability of metronidazole from sugar-coated tablets in humans. I. Effect of gastric acidity and correlation with in vitro dissolution rate | International journal of pharmaceutics | 23(3),277-288 | 10.1016/0378-5173(85)90156-5 | Abstract |
| Ma, luyao zeng, wanying tan, zhiyi wang, rui yang, yi lin, shubin | Activated hepatic nuclear factor-κb in experimental colitis regulates cyp2a5 and metronidazole disposition | Molecular pharmaceutics | 20(2), 1222-1229 | 10.1021/acs.molpharmaceut.2c00890 | Different domain |
| Kokwaro, gilbert o ismail, sabariah glazier, anthony p ward, stephen a edwards, geoffrey | Effect of malaria infection and endotoxin-induced fever on the metabolism of antipyrine and metronidazole in the rat | Biochemical pharmacology | 45(6), 1243-1249 | 10.1016/0006-2952(93)90276-3 | Animals |
| Coppola, paola kerwash, essam cole, susan | Physiologically based pharmacokinetics model in pregnancy: a regulatory perspective on model evaluation | Frontiers in pediatrics | 9(1), 687-978 | 10.3389/fped.2021.687978 | Abstract |
| Burnhill, geoffrey starkey, elizabeth | What do i need to know about metronidazole? | Archives of disease in childhood-education and practice | 103(6), 307-309 | 10.1136/archdischild-2017-313589 | Review |
| Korzeniowski, oksana m | Antibacterial agents in pregnancy | Infectious disease clinics of north america | 9(3), 639-651 | 10.1016/s0891-5520(20)30690-5 | Review |
| Pellegrini, m urso, r giorgi, g bayeli, pf marzocca, g cerretani, d | Is a long-term ranitidine-based triple therapy against | Alimentary pharmacology & therapeutics | 22(4), 245-253 | 10.1111/j.1365-2036.2005.02575.x | Review |
| Boreus, lo hartvig, p | Current literature references on clinical pharmacokinetics | Clinical pharmacokinetics | 7(1), 181-184 |  | Review |
| Lozniewski, a de korwin, jd muhale, f jehl, f | Gastric diffusion of antibiotics used against helicobacter pylori | International journal of antimicrobial agents | 9(3), 181-193 | 10.1016/s0924-8579(97)00049-6 | Review |
| Da silveira vasconcelos, mirele mota, erika freitas gomes-rochette, neuza felix nunes-pinheiro, diana célia sousa | Ginger (zingiber officinale roscoe) | Nonvitamin and nonmineral nutritional supplements | 2019(1), 235-239 | 10.1016/b978-0-12-812491-8.00034-5 | Book |
| Mikelsaar, m siigur, u | Metronidazole and the intestinal microecology of rats | Microbial ecology in health and disease | 5(30), 139-146 | 10.3109/08910609209141308 | Animals |
| Obodozie, obiageri o | Pharmacokinetics and drug interactions of herbal medicines: a missing critical step in the phytomedicine/drug development process | Readings in advanced pharmacokinetics-theory, methods and application | 1-357 |  | Book |
| Erikstrup, lise tornvig aarup, mie hagemann-madsen, rikke dagnaes-hansen, frederik kristensen, brian | Treatment of clostridium difficile infection in mice with vancomycin alone is as effective as treatment with vancomycin and metronidazole in combination | Bmj open gastroenterology | 2(1), 1-38 | 10.1136/bmjgast-2015-000038 | Animals |
| Ralph, edward d kirby, william mm | Bioassay of metronidazole with either anaerobic or aerobic incubation | Journal of infectious diseases | 32(5), 587-591 | Doi.org/10.1093/infdis/132.5.587 | Different domain |
| Soares, guilherme a pires, deivid w pinto, leonardo a rodrigues, gustavo s prospero, andré g biasotti, gabriel ga | The influence of omeprazole on the dissolution processes of ph-dependent magnetic tablets assessed by pharmacomagnetography | Pharmaceutics | 1270-1274 | 10.3390/pharmaceutics13081274 | Different domain |
| Dickey, laura j nailor, michael d sobel, jack d | Guidelines for the treatment of bacterial vaginosis: focus on tinidazole | Therapeutics and clinical risk management | 2009(1), 485-489 | 10.2147/tcrm.s3777 | Review |
| Nduka, sunday o okonta, matthew j esimone, charles o | Effects of zingiber officinale on the plasma pharmacokinetics and lung penetrations of ciprofloxacin and isoniazid | American journal of therapeutics | 20(5), 507-513 | 10.1097/mjt.0b013e31820544be | Different domain |
| Johnson, jacob k laughon, matthew m | Antimicrobial agent dosing in infants | Clinical therapeutics | 38(9), 1948-1960 | 10.1016/j.clinthera.2016.06.017 | Review |
| Lossick, joseph g | Treatment of sexually transmitted vaginosis/vaginitis | Reviews of infectious diseases | 12(6), 665-681 | 10.1093/clinids/12.supplement_6.s665 | Different domain |
| Chifan, maria | Consideration of metronidazole toxicity on development and reproduction | Clujul medical | 83(4), 596-599. |  | Not accessible |
| Freeman, collin d nightingale, charles h nicolau, david p belliveau, paul p tessier, pamela r fu, qiang | Bactericidal activity of low‐dose ceftizoxime plus metronidazole compared with cefoxitin and ampicillin‐sulbactam | Pharmacotherpy | 3(1), 1-7 | [10.1002/j.1875-9114.1994.tb02806.x](https://doi.org/10.1002/j.1875-9114.1994.tb02806.x) | Abstract |
| Hobbiss, jh carr, nd schofield, pf | Are we using the correct dose of metronidazole in colorectal surgery? | Journal of the royal society of medicine | 81(2), 95-96 | 10.1177/014107688808100215 | Different domain |
| Allars, helen coleman, malcolm d norton, raymond s | 1 h nuclear magnetic resonance study of metronidazole metabolism by perfused rat liver | European journal of drug metabolism and pharmacokinetics | 10(3), 253-260 | 10.1007/bf03189750 | Animals |
| Xiao, jianbo hogger, petra | Influence of diabetes on the pharmacokinetic behavior of natural polyphenols | Current drug metabolism | 15(1), 23-29 |  | Not accessible |
| Goldman, peter | The development of 5-nitroimidazoles for the treatment and prophylaxis of anaerobic bacterial infections | Journal of antimicrobial chemotherapy | 10(1), 23-33 | 10.1093/jac/10.suppl_a.23 | Different domain |
| Uddin, sj rouf, r sen, pk alam, ma sarder, mm shilpi, ja | In-vitro studies of the effect of arsenic on the binding of metronidazole at the binding sites of bovine serum albumin | Khulna university studies | 6(1-2), 49-53 | 10.53808/kus.2005.6.1and2.0408-l | Animals |
| Tarral, antoine blesson, séverine mordt, olaf valverde torreele, els sassella, daniela bray, michael a | Determination of an optimal dosing regimen for fexinidazole, a novel oral drug for the treatment of human african trypanosomiasis: first-in-human studies | Clinical pharmacokinetics | 53(6), 565-580 | 10.1007/s40262-014-0136-3 | Different domain |
| Maeda, yasuko ng, siew c durdey, paul burt, caroline torkington, jared rao, p kumar dhruva mayberry, john moshkovska, tanya | Randomized clinical trial of metronidazole ointment versus placebo in perianal crohn's disease | Journal of british surgery | 97(9), 1340-1347 | 10.1002/bjs.7121 | Different domain |
| Orr, leo e puthawala, ajmel syed, am nisar fleming, peter a | Metronidazole and interstitial implantation in the treatment of extensive recurrent head and neck cancers | Cancer | 48(1), 43-47 | 10.1002/1097-0142(19810701)48 | Different domain |
| Di perri, g strosselli, m rondanelli, eg | Therapy of entamebiasis | Journal of chemotherapy | 1(2), 113-122 | /10.1080/1120009x.1989.11738877 | Review |
| Ajiji, priscilla uzunali, anil ripoche, emmanuelle vittaz, emilie | Investigating the efficacy and safety of metronidazole during pregnancy; a systematic review and meta-analysis | European journal of obstetrics & gynecology and reproductive biology: x | 11(1), 100-128 | 10.1016/j.eurox.2021.100128 | Review |
| Raether, w hänel, h | Nitroheterocyclic drugs with broad spectrum activity | Parasitology research | 90(1), 19-39 | 10.1007/s00436-002-0754-9 | Review |
| Elkomy, mohammed h abou-taleb, heba a eid, hussein m yassin, heba a | Fabrication and in vitro/in vivo appraisal of metronidazole intra-gastric buoyant sustained-release tablets in healthy volunteers | Pharmaceutics | 14(4), 863-867 | 10.3390/pharmaceutics14040863 | Abstract |
| Chen, mei-ling yu, lawrence | The use of drug metabolism for prediction of intestinal permeability | Molecular pharmaceutics | 6(1), 74-81 | 10.1021/mp8001864 | Different domain |
| Meyer, joette m ryu, seonyoung pendland, susan l kanyok, thomas p danziger, larry h | In-vitro synergy of paromomycin with metronidazole alone or metronidazole plus hydroxymetronidazole against helicobacter pylori | Journal of antimicrobial chemotherapy | 43(3), 403-406 | 10.1093/jac/43.3.403 | Different domain |
| El-yazbi, amira f aboukhalil, faten m khamis, essam f elkhatib, mohammed aw el-sayed, mahmoud a youssef, rasha m | Simple simultaneous determination of moxifloxacin and metronidazole in complex biological matrices | Rsc advances | 12(25), 15694-15704 | 10.1039/d2ra01631a | Abstract |
| Amid, reza tabeie, mohammad bagher kadkhodazadeh, mahdi mehdizadeh, amir reza youssefi, navid | Local concentration of systemic amoxicillin and metronidazole in healthy and inflamed gingiva: a comparative in vivo study | Drug metabolism and drug interactions | 27(2), 113-118 | 10.1515/dmdi-2012-0003 | Different domain |
| Doan, tri-hanh-dung bernet-camard, marie-françoise hoÿs, sandra janoir, claire péchiné, séverine | Impact of subinhibitory concentrations of metronidazole on morphology, motility, biofilm formation and colonization of clostridioides difficile | Antibiotics | 11(5), 624-628 | 10.3390/antibiotics11050624 | Different domain |
| Bähr, v ullmann, u | The influence of metronidazole and its two main metabolites on murine in vitro lymphocyte transformation | European journal of clinical microbiology | 2(6), 568-570 | 10.1007/bf02016567 | Animals |
| Dobiáš, lubomír černá, milena rössner, pavel šrám, radim | Genotoxicity and carcinogenicity of metronidazole | Veterinary and comparative orthopaedics and traumatology | 317(3), 177-194 | 10.1016/0165-1110(94)90001-9 | Review |
| Garcia-bournissen, facundo altcheh, jaime giglio, norberto mastrantonio, guido della védova, carlos omar | Pediatric clinical pharmacology studies in chagas disease: focus on argentina | Pediatric drugs | 11(1), 33-37 | 10.174-5878/09/0001-0033/$49.95/0 | Review |
| Barker, charlotte is germovsek, eva hoare, rollo l lestner, jodi m lewis, joanna | Pharmacokinetic/pharmacodynamic modelling approaches in paediatric infectious diseases and immunology | Advanced drug delivery reviews | 73(1), 127-139 | 10.1016/j.addr.2014.01.002 | Different domain |
| Turgeon, pl dufresne, lr ogilvie, ri kreeft, jh | Biologic activity of metronidazole in plasma and urine of volunteers with normal or impaired renal function | Surgery | 93(1), 154-157 |  | Not accessible |
| Tabari, ma poźniak, błażej youssefi, mr | Comparative pharmacokinetics of metronidazole in healthy and trichomonas gallinae infected pigeons | British poultry science | 62(4), 485-491 | Https://doi.org/10.1080/00071668.2021.1881043 | Animals |
| Krishnaiah, y. S. Veer raju, p. Dinesh kumar, b. | Pharmacokinetic evaluation of guar gum-based colon-targeted oral drug delivery systems of metronidazole in healthy volunteers | Eurpeon journal of drug metabolism and pharmacokinet | 28 (4), 287-294 |  | Not accessible |
| Cohen-wolkowiez, m. Sampson, m. Bloom, b. T. | Determining population and developmental pharmacokinetics of metronidazole using plasma and dried blood spot samples from premature infants | Pediatric infection dieases journal | 32(9), 956-961 | 10.1097/inf.0b013e3182947cf8 | Different domain |
| Männistö, p karhunen, m mattila, j | Concentrations of metronidazole and tinidazole in female reproductive organs after a single intravenous infusion and after repeated oral administration | Infection | 12(3), 197-201 | 10.1007/bf01640899 | Different domain |
| Świtała, m poźniak, b pasławska, u grabowski, t | Metronidazole pharmacokinetics during rapid growth in turkeys–relation to changes in haemodynamics and drug metabolism | Journal of veterinary pharmacology and therapeutics | 39 (4), 373-380 | Https://doi.org/10.1111/jvp.12283 | Animals |
| Suyagh, maysa collier, paul s millership, jeffrey s iheagwaram, godwill | Metronidazole population pharmacokinetics in preterm neonates using dried blood-spot sampling | Pediatrics | 127 (2), 367-374 | Https://doi.org/10.1542/peds.2010-0807 | Different domain |
| Jeon, ji-young kim, sun-young moon, seol ju oh, kyeongmin | Pharmacokinetic interactions between tegoprazan and metronidazole/tetracycline/bismuth and safety assessment in healthy korean male subjects | Clinical therapeutics | 43(4), 722-734 | Https://doi.org/10.1016/j.clinthera.2021.01.026 | Different domain |
| Loft, s. Sonne, j. Poulsen, h. E. | Inhibition of induction of metronidazole and antipyrine metabolism | European journal of clinical pharmacology | 32 (1), 35‐41 | 10.1007/bf00609955 | Abstract |
| Ioannides-demos, l. L. Farmer, c. Spicer, w. J. | Design and trial of a metronidazole loading dose regimen for patients undergoing emergency surgery | The australian and new zealand journal of surgery | 59 (12), 953-957 | 10.1111/j.1445-2197.1989.tb07638.x | Different domain |
| Boeckh, m. Lode, h. Deppermann, k. M. Grineisen, s. Shokry, f. | Pharmacokinetics and serum bactericidal activities of quinolones in combination with clindamycin, metronidazole, and ornidazole | Antimicrobial agents chemotherapy | 34 (12), 2407-2414 | 10.1128/aac.34.12.2407 | Different domain |
| Naderer, o. J. Dupuis, r. E. Heinzen, e. L. | The influence of norfloxacin and metronidazole on the disposition of mycophenolate mofetil | Journal of clinical pharmacology | 45 (2), 219-26 | 10.1177/0091270004271555 | Different domain |
| Spénard, j. Aumais, c. Massicotte, j. Brunet, j. S. | Effects of food and formulation on the relative bioavailability of bismuth biskalcitrate, metronidazole, and tetracycline given for helicobacter pylori eradication | British journal of clinical pharmacology | 60(4), 374-377 | 10.1111/j.1365-2125.2005.02441.x | Different domain |
| Houghton, g. W. Hundt, h. K. Muller, f. O. Templeton, r. | A comparison of the pharmacokinetics of metronidazole in man after oral administration of single doses of benzoylmetronidazole and metronidazole | British journal of clinical pharmacology | 14 (2), 201‐206 | 10.1111/j.1365-2125.1982.tb01962.x | Different domain |
| Fredricsson, bengt hagström, bertil nord, carl-erikrane, anders | Systemic concentrations of metronidazole and its main metabolites after intravenous oral and vaginal administration | Gynecologic and obstetric investigation | 24(3), 200-207 | Https://doi.org/10.1159/000298803 | Different domain |
| Homeida, mamoun a daneshmend, tawfique k | Metronidazole metabolism following oral benzoylmetronidazole suspension in children with giardiasis | Journal of antimicrobial chemotherapy | 18(2), 213-219 | https://doi.org/10.1093/jac/18.2.213 | Different domain |
| Rubenson, a rosetzsky, a | Single dose prophylaxis with metronidazole in infants during abdominal surgery: a pharmacokinetic study | European journal of clinical pharmacology | 29(1), 625-628 |  | Not accessible |
| Jager-roman, e. Doyle, p. E. Baird-lambert, j. Cvejic | Pharmacokinetics and tissue distribution of metronidazole in the newborn infant | The journal of pediatrics | 100(4),651-654 | Https://doi.org/10.1016/s0022-3476(82)80779-8 | Different domain |
| Upadhyaya, p bhatnagar, v basu | Pharmacokinetics of intravenous metronidazole in neonates | Journal of pediatric surgery | 23(3), 263-265 | Https://doi.org/10.1016/s0022-3468(88)80736-x | Different domain |
| Bergan, tom thorsteinsson, sigurdur b | Pharmacokinetics of metronidazole and its metabolites in reduced renal function | Chemotherapy | 32(4),305-318 |  | Not accessible |
| Wheeler, la de meo, m halula, m george, l | Use of high-pressure liquid chromatography to determine plasma levels of metronidazole and metabolites after intravenous administration | Antimicrobial agents and chemotherapy | 13(2),205-209 | 10.1128/aac.13.2.205 | Different domain |
| Blyden, g. Tscavone, j. M.greenblatt, d. J. | Metronidazole impairs clearance of phenytoin but not of alprazolam or lorazepam | Journal of clinical pharmacology | 28(3), 240-245 | 10.1002/j.1552-4604.1988.tb03139.x | Different domain |
| Nilsson, c aschan, j hentschke, p ringden | The effect of metronidazole on busulfan pharmacokinetics in patients undergoing hematopoietic stem cell transplantation | Bone marrow transplantation | 31(6), 429-435 | 10.1038/sj.bmt.1703896 | Different domain |

**Supplementary Table S2: Quality Assessment of included Article Based on JADAD Scoring**

| **Sr. no** | **Reference** | **Was the study described as randomized?** | **Was the method used to generate the sequence of randomization described and appropriate?** | **Was the study described as double blind?** | **Was the method of double blinding described and appropriate?** | **Was there a description of withdrawls and dropouts?** | **Total score** |
| --- | --- | --- | --- | --- | --- | --- | --- |
| 1 | Siv Fonnes et al ([^1^](#_ENREF_1)) | 0 | 0 | 0 | 0 | 0 | 0 |
| 2 | Yuuichi Sakurai et al([^2^](#_ENREF_2)) | 1 | 0 | 1 | 1 | 1 | 4 |
| 3 | A. A. Visser et al([^3^](#_ENREF_3)) | 1 | 0 | 0 | 0 | 0 | 1 |
| 4 | C. Bergamaschi et al([^4^](#_ENREF_4)) | 1 | 0 | 0 | 0 | 0 | 1 |
| 5 | Andrew Somogyi et al([^5^](#_ENREF_5)) | 0 | 0 | 0 | 0 | 0 | 0 |
| 6 | G. W. Houghton et al([^6^](#_ENREF_6)) | 0 | 0 | 0 | 0 | 0 | 0 |
| 7 | J. L. Shaffer et al([^7^](#_ENREF_7)) | 0 | 0 | 0 | 0 | 0 | 0 |
| 8 | Alan H. Lau et al([^8^](#_ENREF_8)) | 0 | 0 | 0 | 0 | 0 | 0 |
| 9 | C. M. Passmore et al([^9^](#_ENREF_9)) | 0 | 1 | 0 | 0 | 0 | 1 |
| 10 | Karen I. Plaisance et al([^10^](#_ENREF_10)) | 0 | 0 | 0 | 0 | 0 | 0 |
| 11 | Lars Heisterberg et al ([^11^](#_ENREF_11)) | 0 | 1 | 0 | 0 | 0 | 1 |
| 12 | T K Daneshmend et al([^12^](#_ENREF_12)) | 0 | 0 | 0 | 0 | 0 | 0 |
| 13 | Teow Yee Ti et al([^13^](#_ENREF_13)) | 0 | 0 | 0 | 0 | 0 | 0 |
| 14 | Steffen Loft et al([^14^](#_ENREF_14)) | 0 | 0 | 0 | 0 | 0 | 0 |
| 15 | J. Chris Jensen et al([^15^](#_ENREF_15)) | 0 | 0 | 0 | 0 | 0 | 0 |
| 16 | Karin Dilger et al([^16^](#_ENREF_16)) | 0 | 0 | 0 | 0 | 0 | 0 |
| 17 | M. N. Muscara et al([^17^](#_ENREF_17)) | 0 | 0 | 0 | 0 | 0 | 0 |
| 18 | Bilal Ashiq et al([^18^](#_ENREF_18)) | 0 | 0 | 0 | 0 | 0 | 0 |
| 19 | J. F. Thiercelinet al([^19^](#_ENREF_19)) | 1 | 1 | 0 | 0 | 0 | 2 |
| 20 | David R. Guay et al([^20^](#_ENREF_20)) | 0 | 0 | 0 | 0 | 0 | 0 |
| 21 | S. Loft et al([^21^](#_ENREF_21)) | 0 | 0 | 0 | 0 | 0 | 0 |
| 22 | I Amon et al([^22^](#_ENREF_22)) | 0 | 0 | 0 | 0 | 0 | 0 |
| 23 | Christoph Dorn et al([^23^](#_ENREF_23)) | 1 | 0 | 0 | 0 | 0 | 1 |
| 24 | JuriKarjagin et al([^24^](#_ENREF_24)) | 0 | 0 | 0 | 0 | 0 | 0 |
| 25 | Victor.Montalli et al([^25^](#_ENREF_25)) | 0 | 0 | 0 | 0 | 0 | 0 |
| 26 | O. Eradiri et al([^26^](#_ENREF_26)) | 0 | 0 | 0 | 0 | 0 | 0 |
| 27 | D.E Sachwarts et al([^27^](#_ENREF_27)) | 0 | 0 | 0 | 0 | 0 | 0 |
| 28 | Ingrid Amon et al([^28^](#_ENREF_28)) | 0 | 0 | 0 | 0 | 0 | 0 |
| 29 | Tom Bergan et al([^29^](#_ENREF_29)) | 0 | 0 | 0 | 0 | 0 | 0 |
| 30 | Kyoung-Ah Kim et al([^30^](#_ENREF_30)) | 1 | 1 | 1 | 1 | 0 | 4 |
| 31 | S. Hanifah et al([^31^](#_ENREF_31)) | 0 | 0 | 0 | 0 | 0 | 0 |
| 32 | I.Lares-Asseff et al([^32^](#_ENREF_32)) | 0 | 0 | 0 | 0 | 0 | 0 |
| 33 | BengtLjungberg et al([^33^](#_ENREF_33)) | 0 | 0 | 0 | 0 | 0 | 0 |
| 34 | Geoffrey Farre et al([^34^](#_ENREF_34)) | 0 | 0 | 0 | 0 | 0 | 0 |
| 35 | S. Loft et al([^35^](#_ENREF_35)) | 0 | 0 | 0 | 0 | 0 | 0 |
| 36 | A. Somogyi et al([^36^](#_ENREF_36)) | 0 | 0 | 0 | 0 | 0 | 0 |
| 37 | Hayder A. Kurjiet al([^37^](#_ENREF_37)) | 0 | 0 | 0 | 0 | 0 | 0 |
| 38 | E. Jager-Roman et al([^38^](#_ENREF_38)) | 0 | 0 | 0 | 0 | 0 | 0 |
| 39 | Shampa Das et al([^39^](#_ENREF_39)) | 1 | 1 | 1 | 0 | 0 | 3 |
| 40 | L.F David et al([^40^](#_ENREF_40)) | 1 | 1 | 1 | 0 | 0 | 3 |
| 41 | Andrew F. Goddard et al([^41^](#_ENREF_41)) | 1 | 0 | 1 | 0 | 0 | 2 |
| 42 | S. A. Calafattiet al([^42^](#_ENREF_42)) | 1 | 0 | 1 | 0 | 0 | 2 |
| 43 | G.W. Houghton et al([^43^](#_ENREF_43)) | 0 | 0 | 0 | 0 | 0 | 0 |
| 44 | O. Obodozieet al([^44^](#_ENREF_44)) | 1 | 1 | 0 | 0 | 0 | 2 |
| 45 | K. Rajnarayanaet al([^45^](#_ENREF_45)) | 0 | 0 | 0 | 0 | 0 | 0 |
| 46 | K.Rajnarayana et al([^46^](#_ENREF_46)) | 0 | 0 | 0 | 0 | 0 | 0 |
| 47 | A. Melande et al([^47^](#_ENREF_47)) | 0 | 0 | 0 | 0 | 0 | 0 |
| 48 | XinWanget al([^48^](#_ENREF_48)) | 0 | 0 | 0 | 0 | 0 | 0 |
| 49 | J. S Wang et al([^49^](#_ENREF_49)) | 1 | 0 | 1 | 0 | 0 | 2 |
| 50 | David Pierceet al([^50^](#_ENREF_50)) | 1 | 1 | 1 | 0 | 0 | 3 |
| 51 | M. J. Jessa et al([^51^](#_ENREF_51)) | 1 | 0 | 1 | 0 | 0 | 2 |
| 52 | JyrkiMattila et al([^52^](#_ENREF_52)) | 1 | 0 | 0 | 0 | 0 | 1 |
| 53 | H. Mattieet al([^53^](#_ENREF_53)) | 1 | 0 | 0 | 0 | 0 | 1 |
| 54 | J.M.Ventura et al([^54^](#_ENREF_54)) | 0 | 0 | 0 | 0 | 0 | 0 |
| 55 | O. Hamberg et al([^55^](#_ENREF_55)) | 0 | 0 | 0 | 0 | 0 | 0 |
| 56 | Kelly A. Sprandelet al([^56^](#_ENREF_56)) | 1 | 1 | 0 | 0 | 0 | 2 |
| 57 | Y. Wuet al([^57^](#_ENREF_57)) | 0 | 0 | 0 | 0 | 0 | 0 |
| 58 | Salas‐Herrera et al([^58^](#_ENREF_58)) | 0 | 0 | 0 | 0 | 0 | 0 |
| 59 | BerganTom et al([^59^](#_ENREF_59)) | 0 | 0 | 0 | 0 | 0 | 0 |
| 60 | Fredricsson et al([^60^](#_ENREF_60)) | 0 | 0 | 0 | 0 | 0 | 0 |
| 61 | Roux et al([^61^](#_ENREF_61)) | 0 | 0 | 0 | 0 | 0 | 0 |
| 62 | Houghton et al([^62^](#_ENREF_62)) | 0 | 0 | 0 | 0 | 0 | 0 |
| 63 | Loft et al([^63^](#_ENREF_63)) | 0 | 0 | 0 | 0 | 0 | 0 |
| 64 | Cunningham et al([^64^](#_ENREF_64)) | 1 | 0 | 0 | 0 | 0 | 1 |
| 65 | Lau et al([^65^](#_ENREF_65)) | 0 | 0 | 0 | 0 | 0 | 0 |
| 66 | Amon et al([^66^](#_ENREF_66)) | 0 | 0 | 0 | 0 | 0 | 0 |
| 67 | Alper et al([^67^](#_ENREF_67)) | 0 | 0 | 0 | 0 | 0 | 0 |

**Supplementary Table S3: Quality Assessment of included Article Based on Critical Appraisal Skill Program (CASP)**

| **Sr. no** | **Reference** | **1** | **2** | **3** | **4** | **5** | **6** | **7** | **8** | **9** | **10** | **CASP Score** |
| --- | --- | --- | --- | --- | --- | --- | --- | --- | --- | --- | --- | --- |
| 1 | Siv Fonnes et al ([^1^](#_ENREF_1)) | Y | Y | Y | Y | Y | Y | N | Y | Y | Y | 9 |
| 2 | Yuuichi Sakurai et al([^2^](#_ENREF_2)) | Y | Y | Y | Y | Y | Y | N | Y | Y | Y | 9 |
| 3 | A. A. Visser et al([^3^](#_ENREF_3)) | Y | Y | Y | Y | CT | Y | N | CT | CT | Y | 6 |
| 4 | C. Bergamaschi et al([^4^](#_ENREF_4)) | Y | Y | Y | Y | Y | Y | N | Y | Y | Y | 9 |
| 5 | Andrew Somogyi et al([^5^](#_ENREF_5)) | Y | Y | Y | Y | Y | Y | N | Y | Y | Y | 9 |
| 6 | G. W. Houghton et al([^6^](#_ENREF_6)) | Y | Y | CT | Y | Y | Y | N | Y | Y | Y | 8 |
| 7 | J. L. Shaffer et al([^7^](#_ENREF_7)) | Y | Y | CT | Y | Y | Y | N | CT | CT | Y | 6 |
| 8 | Alan H. Lau et al([^8^](#_ENREF_8)) | Y | Y | CT | Y | Y | Y | N | CT | Y | Y | 7 |
| 9 | C. M. Passmore et al([^9^](#_ENREF_9)) | Y | Y | Y | Y | Y | Y | N | Y | Y | Y | 9 |
| 10 | Karen I. Plaisance et al([^10^](#_ENREF_10)) | Y | Y | CT | Y | Y | Y | N | CT | Y | Y | 7 |
| 11 | Lars Heisterberg et al ([^11^](#_ENREF_11)) | Y | Y | CT | Y | Y | Y | N | N | Y | Y | 7 |
| 12 | T K Daneshmend et al([^12^](#_ENREF_12)) | Y | Y | Y | Y | Y | Y | N | Y | Y | Y | 9 |
| 13 | Teow Yee Ti et al([^13^](#_ENREF_13)) | Y | Y | Y | Y | Y | Y | N | N | Y | Y | 8 |
| 14 | Steffen Loft et al([^14^](#_ENREF_14)) | Y | Y | CT | Y | Y | Y | N | Y | Y | Y | 8 |
| 15 | J. Chris Jensen et al([^15^](#_ENREF_15)) | Y | Y | CT | Y | Y | Y | N | CT | Y | Y | 7 |
| 16 | Karin Dilger et al([^16^](#_ENREF_16)) | Y | Y | Y | Y | Y | Y | N | N | Y | Y | 8 |
| 17 | M. N. Muscara et al([^17^](#_ENREF_17)) | Y | Y | Y | Y | Y | Y | N | Y | Y | Y | 9 |
| 18 | Bilal Ashiq et al([^18^](#_ENREF_18)) | Y | Y | Y | Y | Y | Y | N | Y | Y | Y | 9 |
| 19 | J. F. Thiercelinet al([^19^](#_ENREF_19)) | Y | Y | Y | Y | Y | Y | N | N | Y | Y | 8 |
| 20 | David R. Guay et al([^20^](#_ENREF_20)) | Y | Y | Y | Y | Y | Y | N | Y | Y | Y | 9 |
| 21 | S. Loft et al([^21^](#_ENREF_21)) | Y | Y | Y | Y | Y | Y | N | Y | Y | Y | 9 |
| 22 | I Amon et al([^22^](#_ENREF_22)) | Y | Y | CT | Y | Y | Y | N | Y | Y | Y | 8 |
| 23 | Christoph Dorn et al([^23^](#_ENREF_23)) | Y | Y | Y | Y | Y | Y | N | Y | Y | Y | 9 |
| 24 | JuriKarjagin et al([^24^](#_ENREF_24)) | Y | Y | Y | Y | Y | Y | N | N | Y | Y | 8 |
| 25 | Victor.Montalli et al([^25^](#_ENREF_25)) | Y | Y | Y | Y | Y | Y | N | Y | Y | Y | 9 |
| 26 | O. Eradiri et al([^26^](#_ENREF_26)) | Y | Y | Y | Y | Y | Y | N | Y | Y | Y | 9 |
| 27 | D.E Sachwarts et al([^27^](#_ENREF_27)) | Y | Y | Y | Y | Y | Y | N | N | Y | Y | 8 |
| 28 | Ingrid Amon et al([^28^](#_ENREF_28)) | Y | Y | Y | Y | Y | Y | N | Y | Y | Y | 8 |
| 29 | Tom Bergan et al([^29^](#_ENREF_29)) | Y | Y | CT | Y | Y | Y | N | Y | CT | Y | 7 |
| 30 | Kyoung-Ah Kim et al([^30^](#_ENREF_30)) | Y | Y | Y | Y | Y | Y | N | Y | Y | Y | 9 |
| 31 | S. Hanifah et al([^31^](#_ENREF_31)) | Y | Y | Y | Y | Y | Y | N | Y | N | Y | 8 |
| 32 | I.Lares-Asseff et al([^32^](#_ENREF_32)) | Y | Y | Y | Y | Y | Y | N | Y | Y | Y | 9 |
| 33 | BengtLjungberg et al([^33^](#_ENREF_33)) | Y | Y | Y | Y | Y | Y | N | Y | Y | Y | 9 |
| 34 | Geoffrey Farre et al([^34^](#_ENREF_34)) | Y | Y | Y | Y | Y | Y | N | Y | Y | Y | 9 |
| 35 | S. Loft et al([^35^](#_ENREF_35)) | Y | Y | CT | Y | Y | Y | N | Y | Y | Y | 8 |
| 36 | A. Somogyi et al([^36^](#_ENREF_36)) | Y | Y | Y | Y | Y | Y | N | Y | Y | Y | 9 |
| 37 | Hayder A. Kurjiet al([^37^](#_ENREF_37)) | Y | Y | Y | Y | Y | Y | N | Y | Y | Y | 9 |
| 38 | E. Jager-Roman et al([^38^](#_ENREF_38)) | Y | Y | CT | Y | Y | Y | N | Y | Y | Y | 8 |
| 39 | Shampa Das et al([^39^](#_ENREF_39)) | Y | Y | Y | Y | Y | Y | N | N | Y | Y | 8 |
| 41 | L.F David et al([^40^](#_ENREF_40)) | Y | Y | Y | Y | Y | Y | N | Y | Y | Y | 9 |
| 42 | Andrew F. Goddard et al([^41^](#_ENREF_41)) | Y | Y | Y | Y | Y | Y | N | Y | Y | Y | 9 |
| 43 | S. A. Calafattiet al([^42^](#_ENREF_42)) | Y | Y | Y | Y | Y | Y | N | Y | Y | Y | 9 |
| 44 | G.W. Houghton et al([^43^](#_ENREF_43)) | N | Y | CT | Y | Y | Y | N | N | Y | Y | 6 |
| 45 | O. Obodozieet al([^44^](#_ENREF_44)) | Y | Y | Y | Y | Y | Y | N | Y | Y | Y | 9 |
| 46 | K. Rajnarayanaet al([^45^](#_ENREF_45)) | Y | Y | Y | Y | Y | Y | N | Y | Y | Y | 9 |
| 47 | K.Rajnarayana et al([^46^](#_ENREF_46)) | Y | Y | Y | Y | Y | Y | N | Y | Y | Y | 9 |
| 48 | A. Melande et al([^47^](#_ENREF_47)) | Y | Y | Y | Y | Y | Y | N | Y | Y | Y | 9 |
| 49 | XinWanget al([^48^](#_ENREF_48)) | Y | Y | Y | Y | Y | Y | N | Y | Y | Y | 9 |
| 50 | J. S Wang et al([^49^](#_ENREF_49)) | Y | Y | Y | Y | Y | Y | N | Y | Y | Y | 9 |
| 51 | David Pierceet al([^50^](#_ENREF_50)) | Y | Y | Y | Y | Y | Y | N | N | Y | Y | 9 |
| 52 | M. J. Jessa et al([^51^](#_ENREF_51)) | Y | Y | Y | Y | Y | Y | N | Y | Y | Y | 9 |
| 53 | JyrkiMattila et al([^52^](#_ENREF_52)) | Y | Y | Y | Y | Y | Y | N | Y | Y | Y | 9 |
| 54 | H. Mattieet al([^53^](#_ENREF_53)) | Y | Y | N | Y | Y | Y | N | N | Y | Y | 7 |
| 56 | J.M.Ventura et al([^54^](#_ENREF_54)) | Y | Y | Y | Y | Y | Y | N | Y | Y | Y | 9 |
| 57 | O. Hamberg et al([^55^](#_ENREF_55)) | N | Y | Y | Y | Y | Y | N | Y | Y | Y | 8 |
| 58 | Kelly A. Sprandelet al([^56^](#_ENREF_56)) | Y | Y | Y | Y | Y | Y | N | Y | Y | Y | 9 |
| 59 | Y. Wuet al([^57^](#_ENREF_57)) | Y | Y | Y | Y | Y | Y | N | Y | Y | Y | 9 |
| 60 | Salas‐Herrera et al([^58^](#_ENREF_58)) | Y | Y | Y | Y | Y | Y | N | Y | Y | Y | 9 |
| 61 | BerganTom et al([^59^](#_ENREF_59)) | Y | Y | Y | Y | Y | Y | N | N | N | Y | 8 |
| 60 | Fredricsson et al([^60^](#_ENREF_60)) | Y | Y | Y | Y | Y | CT | CT | Y | Y | Y | 8 |
| 61 | Roux et al([^61^](#_ENREF_61)) | Y | Y | Y | Y | Y | Y | CT | CT | Y | Y | 8 |
| 62 | Houghton et al([^62^](#_ENREF_62)) | Y | Y | Y | Y | Y | Y | CT | CT | Y | Y | 8 |
| 63 | Loft et al([^63^](#_ENREF_63)) | Y | Y | Y | Y | Y | Y | CT | Y | Y | Y | 9 |
| 64 | Cunningham et al([^64^](#_ENREF_64)) | Y | Y | Y | Y | Y | Y | CT | Y | Y | Y | 9 |
| 65 | Lau et al([^65^](#_ENREF_65)) | Y | Y | Y | Y | Y | Y | CT | Y | Y | Y | 9 |
| 66 | Amon et al([^66^](#_ENREF_66)) | Y | Y | Y | Y | Y | Y | CT | Y | Y | Y | 9 |
| 67 | Alper et al([^67^](#_ENREF_67)) | Y | Y | Y | Y | Y | Y | CT | Y | Y | Y | 9 |

Y= Yes, N= No, CT= can’t tell

**Questions:**

1: Was there a clear statement of the aims of the research?

2: Is a qualitative methodology appropriate?

3: Was the research design appropriate to address the aims of the research?

4: Are the study's theoretical underpinnings clear, consistent, and conceptually coherent?

5: Was the recruitment strategy appropriate to the aims of the search?

6: Was the data collected in a way that addressed the research issue?

7: Has the relationship between researchers and participants been adequately considered?

8: Have ethical issues been taken into consideration?

9: Was the data analysis sufficiently rigorous?

10: Is there a clear statement of findings?

**Supplementary Table S4: Quality Assessment of included Article Based on Critical Appraisal Clinical Pharmacokinetic Tool (CACP)**

| **Sr. no** | **Reference** | **1** | **2** | **3** | **4** | **5** | **6** | **7** | **8** | **9** | **10** | **11** | **12** | **13** | **14** | **15** | **16** | **17** | **18** | **19** | **20** | **21** | **Total Score** |
| --- | --- | --- | --- | --- | --- | --- | --- | --- | --- | --- | --- | --- | --- | --- | --- | --- | --- | --- | --- | --- | --- | --- | --- |
| 1 | Siv Fonnes et al ([^1^](#_ENREF_1)) | Y | Y | Y | Y | Y | Y | Y | IDK | Y | Y | Y | IDK | Y | Y | Y | Y | N | Y | Y | Y | Y | 18 |
| 2 | Yuuichi Sakurai et al([^2^](#_ENREF_2)) | Y | Y | Y | Y | Y | Y | Y | IDK | Y | Y | Y | IDK | Y | Y | Y | Y | N | Y | Y | Y | Y | 18 |
| 3 | A. A. Visser et al([^3^](#_ENREF_3)) | Y | N | Y | Y | Y | N | N | IDK | Y | Y | Y | IDK | Y | N | IDK | Y | N | Y | Y | Y | Y | 13 |
| 4 | C. Bergamaschi et al([^4^](#_ENREF_4)) | Y | Y | Y | Y | Y | Y | Y | IDK | Y | Y | Y | IDK | Y | Y | Y | Y | N | Y | Y | Y | Y | 18 |
| 5 | Andrew Somogyi et al([^5^](#_ENREF_5)) | Y | Y | Y | Y | Y | Y | Y | IDK | Y | Y | Y | IDK | Y | Y | Y | Y | Y | Y | Y | Y | Y | 19 |
| 6 | G. W. Houghton et al([^6^](#_ENREF_6)) | Y | N | Y | Y | Y | Y | Y | IDK | Y | Y | Y | IDK | Y | Y | Y | Y | N | Y | Y | Y | Y | 17 |
| 7 | J. L. Shaffer et al([^7^](#_ENREF_7)) | Y | Y | Y | Y | Y | Y | Y | IDK | Y | Y | Y | IDK | Y | Y | Y | Y | N | Y | Y | Y | Y | 18 |
| 8 | Alan H. Lau et al([^8^](#_ENREF_8)) | Y | Y | Y | Y | Y | Y | Y | IDK | Y | Y | Y | IDK | Y | Y | Y | Y | N | Y | Y | Y | Y | 18 |
| 9 | C. M. Passmore et al([^9^](#_ENREF_9)) | Y | Y | Y | Y | Y | N | N | IDK | Y | Y | Y | IDK | Y | Y | Y | Y | N | Y | Y | Y | Y | 16 |
| 10 | Karen I. Plaisance et al([^10^](#_ENREF_10)) | Y | Y | Y | Y | Y | Y | Y | IDK | Y | Y | Y | IDK | Y | Y | Y | Y | N | Y | Y | Y | Y | 18 |
| 11 | Lars Heisterberg et al ([^11^](#_ENREF_11)) | Y | Y | Y | Y | Y | N | N | IDK | Y | Y | Y | IDK | IDK | IDK | IDK | Y | N | Y | Y | Y | Y | 13 |
| 12 | T K Daneshmend et al([^12^](#_ENREF_12)) | Y | Y | Y | Y | Y | Y | Y | IDK | Y | Y | Y | IDK | Y | Y | Y | Y | Y | Y | Y | Y | Y | 19 |
| 13 | Teow Yee Ti et al([^13^](#_ENREF_13)) | Y | Y | Y | Y | Y | IDK | IDK | IDK | Y | Y | Y | IDK | Y | Y | Y | Y | N | Y | Y | Y | Y | 16 |
| 14 | Steffen Loft et al([^14^](#_ENREF_14)) | Y | Y | Y | Y | Y | Y | Y | IDK | Y | Y | Y | IDK | Y | N | IDK | Y | N | Y | Y | Y | Y | 17 |
| 15 | J. Chris Jensen et al([^15^](#_ENREF_15)) | Y | Y | Y | Y | Y | Y | Y | IDK | Y | Y | Y | IDK | Y | Y | Y | Y | N | Y | Y | Y | Y | 18 |
| 16 | Karin Dilger et al([^16^](#_ENREF_16)) | Y | Y | Y | Y | Y | Y | Y | IDK | Y | Y | Y | IDK | Y | Y | Y | Y | N | Y | Y | Y | Y | 18 |
| 17 | M. N. Muscara et al([^17^](#_ENREF_17)) | Y | Y | Y | Y | Y | Y | Y | IDK | Y | Y | Y | IDK | Y | Y | Y | Y | N | Y | Y | Y | Y | 18 |
| 18 | Bilal Ashiq et al([^18^](#_ENREF_18)) | Y | Y | Y | Y | Y | Y | Y | IDK | Y | Y | Y | IDK | Y | Y | Y | Y | N | Y | Y | Y | Y | 18 |
| 19 | J. F. Thiercelinet al([^19^](#_ENREF_19)) | Y | Y | Y | Y | Y | Y | Y | IDK | Y | Y | Y | IDK | Y | Y | Y | Y | N | Y | Y | Y | Y | 18 |
| 20 | David R. Guay et al([^20^](#_ENREF_20)) | Y | Y | Y | Y | Y | Y | Y | IDK | Y | Y | Y | IDK | Y | Y | Y | Y | N | Y | Y | Y | Y | 18 |
| 21 | S. Loft et al([^21^](#_ENREF_21)) | Y | Y | Y | Y | Y | Y | Y | IDK | Y | Y | Y | IDK | Y | Y | Y | Y | Y | Y | Y | Y | Y | 19 |
| 22 | I Amon et al([^22^](#_ENREF_22)) | Y | Y | Y | Y | Y | Y | Y | IDK | Y | Y | Y | IDK | N | N | IDK | Y | N | Y | Y | Y | Y | 15 |
| 23 | Christoph Dorn et al([^23^](#_ENREF_23)) | Y | Y | Y | Y | Y | Y | Y | IDK | Y | Y | Y | IDK | Y | Y | Y | Y | N | Y | Y | Y | Y | 17 |
| 24 | JuriKarjagin et al([^24^](#_ENREF_24)) | Y | Y | Y | Y | Y | Y | Y | IDK | Y | Y | Y | IDK | Y | Y | Y | Y | Y | Y | Y | Y | Y | 18 |
| 25 | Victor.Montalli et al([^25^](#_ENREF_25)) | Y | Y | Y | Y | Y | Y | Y | IDK | Y | Y | Y | IDK | Y | Y | Y | Y | N | Y | Y | Y | Y | 18 |
| 26 | O. Eradiri et al([^26^](#_ENREF_26)) | Y | Y | Y | Y | Y | Y | Y | IDK | Y | Y | Y | IDK | Y | IDK | IDK | Y | N | Y | Y | Y | Y | 16 |
| 27 | D.E Sachwarts et al([^27^](#_ENREF_27)) | Y | N | Y | Y | Y | Y | Y | IDK | Y | Y | Y | IDK | Y | Y | Y | Y | N | Y | Y | Y | Y | 17 |
| 28 | Ingrid Amon et al([^28^](#_ENREF_28)) | Y | Y | Y | Y | Y | Y | Y | IDK | Y | Y | Y | IDK | Y | N | IDK | Y | N | Y | Y | Y | Y | 17 |
| 29 | Tom Bergan et al([^29^](#_ENREF_29)) | Y | Y | Y | Y | Y | Y | Y | IDK | Y | Y | Y | IDK | Y | Y | Y | Y | N | Y | Y | Y | Y | 18 |
| 30 | Kyoung-Ah Kim et al([^30^](#_ENREF_30)) | Y | Y | Y | Y | Y | Y | Y | IDK | Y | Y | Y | IDK | Y | N | IDK | Y | N | Y | Y | Y | Y | 17 |
| 31 | S. Hanifah et al([^31^](#_ENREF_31)) | Y | Y | Y | Y | Y | Y | Y | IDK | Y | Y | Y | IDK | Y | Y | Y | Y | N | Y | Y | Y | Y | 18 |
| 32 | I.Lares-Asseff et al([^32^](#_ENREF_32)) | Y | Y | Y | Y | Y | Y | IDK | IDK | Y | Y | Y | IDK | Y | Y | Y | Y | Y | Y | Y | Y | Y | 18 |
| 33 | BengtLjungberg et al([^33^](#_ENREF_33)) | Y | Y | Y | Y | Y | Y | Y | IDK | Y | Y | Y | IDK | Y | Y | Y | Y | Y | Y | Y | Y | Y | 17 |
| 34 | Geoffrey Farre et al([^34^](#_ENREF_34)) | Y | Y | Y | Y | Y | Y | Y | IDK | Y | Y | Y | IDK | Y | N | Y | Y | Y | Y | Y | Y | Y | 16 |
| 35 | S. Loft et al([^35^](#_ENREF_35)) | Y | Y | Y | Y | Y | Y | Y | IDK | Y | Y | Y | IDK | Y | Y | N | Y | N | Y | Y | Y | Y | 17 |
| 36 | A. Somogyi et al([^36^](#_ENREF_36)) | Y | Y | Y | Y | Y | Y | Y | IDK | Y | Y | Y | IDK | Y | Y | Y | Y | N | Y | Y | Y | Y | 18 |
| 37 | Hayder A. Kurjiet al([^37^](#_ENREF_37)) | Y | Y | Y | Y | Y | Y | Y | IDK | Y | Y | Y | IDK | N | N | Y | Y | N | Y | Y | Y | Y | 16 |
| 38 | E. Jager-Roman et al([^38^](#_ENREF_38)) | Y | Y | Y | Y | Y | Y | Y | IDK | Y | Y | Y | IDK | IDK | IDK | IDK | Y | N | Y | Y | Y | Y | 15 |
| 39 | Shampa Das et al([^39^](#_ENREF_39)) | Y | Y | Y | Y | Y | Y | Y | IDK | Y | Y | Y | IDK | N | Y | Y | Y | N | Y | Y | Y | Y | 17 |
| 40 | L.F David et al([^40^](#_ENREF_40)) | Y | Y | Y | Y | Y | Y | Y | IDK | Y | Y | Y | IDK | Y | Y | Y | Y | N | Y | Y | Y | Y | 18 |
| 41 | Andrew F. Goddard et al([^41^](#_ENREF_41)) | Y | Y | Y | Y | Y | Y | Y | IDK | Y | Y | Y | IDK | Y | Y | Y | Y | Y | Y | Y | Y | Y | 18 |
| 42 | S. A. Calafattiet al([^42^](#_ENREF_42)) | Y | Y | Y | Y | Y | Y | Y | IDK | Y | Y | Y | IDK | Y | Y | Y | Y | N | Y | Y | Y | Y | 18 |
| 43 | G.W. Houghton et al([^43^](#_ENREF_43)) | N | Y | Y | Y | Y | Y | Y | IDK | Y | Y | Y | IDK | N | Y | Y | Y | Y | Y | Y | Y | Y | 17 |
| 44 | O. Obodozieet al([^44^](#_ENREF_44)) | Y | Y | Y | Y | Y | Y | Y | IDK | Y | Y | Y | IDK | Y | Y | Y | Y | N | Y | Y | Y | Y | 18 |
| 45 | K. Rajnarayanaet al([^45^](#_ENREF_45)) | Y | Y | Y | Y | Y | Y | Y | IDK | Y | Y | Y | IDK | Y | Y | Y | Y | N | Y | Y | Y | Y | 18 |
| 46 | K.Rajnarayana et al([^46^](#_ENREF_46)) | Y | Y | Y | Y | Y | Y | Y | IDK | Y | Y | Y | IDK | Y | IDK | Y | Y | N | Y | Y | Y | Y | 17 |
| 47 | A. Melande et al([^47^](#_ENREF_47)) | Y | Y | Y | Y | Y | Y | Y | IDK | Y | Y | Y | IDK | Y | IDK | Y | Y | N | Y | Y | Y | Y | 17 |
| 48 | XinWanget al([^48^](#_ENREF_48)) | Y | N | Y | Y | Y | Y | Y | IDK | Y | Y | Y | IDK | Y | Y | Y | Y | N | Y | Y | Y | Y | 17 |
| 49 | J. S Wang et al([^49^](#_ENREF_49)) | Y | N | Y | Y | Y | Y | Y | IDK | Y | Y | Y | IDK | Y | N | N | Y | N | Y | Y | Y | Y | 15 |
| 50 | David Pierceet al([^50^](#_ENREF_50)) | Y | Y | Y | Y | Y | Y | Y | IDK | Y | Y | Y | IDK | IDK | Y | Y | Y | N | Y | Y | Y | Y | 17 |
| 51 | M. J. Jessa et al([^51^](#_ENREF_51)) | Y | Y | Y | Y | Y | Y | Y | IDK | Y | Y | Y | IDK | Y | Y | Y | Y | Y | Y | Y | Y | Y | 19 |
| 52 | JyrkiMattila et al([^52^](#_ENREF_52)) | Y | Y | Y | Y | Y | Y | Y | IDK | Y | Y | Y | IDK | Y | Y | Y | Y | N | Y | Y | Y | Y | 18 |
| 53 | H. Mattieet al([^53^](#_ENREF_53)) | Y | Y | Y | Y | Y | N | N | IDK | Y | Y | Y | IDK | N | N | N | Y | N | Y | Y | Y | Y | 13 |
| 54 | J.M.Ventura et al([^54^](#_ENREF_54)) | Y | Y | Y | Y | Y | Y | Y | IDK | Y | Y | Y | IDK | Y | Y | Y | Y | N | Y | Y | Y | Y | 18 |
| 55 | O. Hamberg et al([^55^](#_ENREF_55)) | N | Y | Y | Y | Y | N | N | IDK | Y | Y | Y | IDK | N | Y | Y | Y | N | Y | Y | Y | Y | 14 |
| 56 | Kelly A. Sprandelet al([^56^](#_ENREF_56)) | Y | Y | Y | Y | Y | Y | Y | IDK | Y | Y | Y | IDK | Y | IDK | IDK | Y | N | Y | Y | Y | Y | 16 |
| 57 | Y. Wuet al([^57^](#_ENREF_57)) | Y | Y | Y | Y | Y | Y | Y | IDK | Y | Y | Y | IDK | Y | Y | Y | Y | N | Y | Y | Y | Y | 18 |
| 58 | Salas‐Herrera et al([^58^](#_ENREF_58)) | Y | Y | Y | Y | Y | Y | Y | IDK | Y | Y | Y | IDK | Y | IDK | IDK | Y | N | Y | Y | Y | Y | 16 |
| 59 | BerganTom et al([^59^](#_ENREF_59)) | Y | Y | Y | Y | Y | Y | Y | IDK | Y | Y | Y | IDK | Y | Y | Y | Y | N | Y | Y | Y | Y | 18 |
| 60 | Fredricsson et al([^60^](#_ENREF_60)) | Y | Y | Y | Y | Y | Y | Y | IDK | Y | Y | Y | IDK | Y | Y | Y | Y | N | Y | Y | Y | Y | 18 |
| 61 | Roux et al([^61^](#_ENREF_61)) | Y | Y | Y | Y | Y | Y | Y | IDK | Y | Y | Y | IDK | Y | Y | N | Y | N | Y | Y | Y | Y | 18 |
| 62 | Houghton et al([^62^](#_ENREF_62)) | Y | Y | Y | Y | Y | Y | Y | IDK | Y | Y | Y | IDK | Y | Y | Y | Y | N | Y | Y | Y | Y | 17 |
| 63 | Loft et al([^63^](#_ENREF_63)) | Y | Y | Y | Y | Y | Y | Y | IDK | Y | Y | Y | IDK | IDK | Y | Y | Y | N | Y | Y | Y | Y | 17 |
| 64 | Cunningham et al([^64^](#_ENREF_64)) | Y | Y | Y | Y | Y | Y | Y | IDK | Y | Y | Y | IDK | Y | N | Y | Y | N | Y | Y | Y | Y | 17 |
| 65 | Lau et al([^65^](#_ENREF_65)) | Y | Y | Y | Y | Y | Y | Y | IDK | Y | Y | Y | IDK | Y | Y | Y | Y | N | Y | Y | Y | Y | 18 |
| 66 | Amon et al([^66^](#_ENREF_66)) | Y | Y | Y | Y | Y | Y | Y | IDK | Y | Y | Y | IDK | IDK | N | IDK | Y | N | Y | Y | Y | Y | 15 |
| 67 | Alper et al([^67^](#_ENREF_67)) | Y | Y | Y | Y | Y | Y | Y | IDK | Y | Y | Y | IDK | Y | IDK | Y | Y | N | Y | Y | Y | Y | 17 |

Y= Yes, N= No, IDK= I don’t know

Questions

1: Was a clear description of the objectives of the study provided?

2: Was a clear and comprehensive rationale provided to support the purpose of the study?

3: Was the chosen study design appropriately selected and justified

4: Was the dosing of the drug in the study justified for the intended study?

5: Were the outcome measures endpoints of the study appropriate to address the objectives of the study?

6: Were the exclusion criteria of included participants included AND appropriate for the intended outcomes of the study?

7: Where applicable, were the relevant baseline characteristics of the participant’s adequately described?

8: Were plausible interacting covariates described a *pripori* or in post hoc evaluation?

9: Was the description of the used biological sample analytical methods sample analysis methods or citations of prior validation studies provided in the publication or affiliated appendix?

10: Was the method of data sampling of analytics is appropriate for the study?

11: Was a clear description of sampling site provided and justified?

12: Was the number of half-lives elapsed within the sampling periods appropriate for analyzed drug?

13: Were sampling storage conditions appropriate and described in manner that could be accurately replicated?

14: If applicable, was there a clear description of pharmacokinetic model, its development, validation and justification for use?

15: Was the described population pharmacokinetic approach validation method appropriate for the analysis?

16: Were the essential pharmacokinetic parameters required to make the results applicable in clinical settings included?

17: Were the pharmacokinetic equations used to calculate the patients’ pharmacokinetic parameters presented or cited within article?

18: Were the chosen statistical tests and software to perform the statistical analysis appropriate to achieve the study objectives?

19: Were all patients enrolled in the study accounted for?

20: In the event of missing data or outliers, was the process for analysis justified and appropriate?

21: Were appropriate summary statistics to describe centrality and variance used to presented pharmacokinetic results?

**Supplementary Table S5: Quality Assessment of included Article Based on Cochrane Collaboration Tool**

| **Sr. no** | **Reference** | **Random sequence (selection bias)** | **Allocation concealment (selection bias)** | **Blinding of participant and researchers (performance bias)** | **Blinding of outcome assessment (detection bias)** | **Incomplete outcome data (attrition bias)** | **Selective reporting (reporting bias)** | **Other bias** | **Total score** |
| --- | --- | --- | --- | --- | --- | --- | --- | --- | --- |
| 1 | Siv Fonnes et al ([^1^](#_ENREF_1)) | UR | LR | LR | UR | LR | HR | LR | 4 |
| 2 | Yuuichi Sakurai et al([^2^](#_ENREF_2)) | LR | LR | LR | LR | LR | LR | LR | 6 |
| 3 | A. A. Visser et al([^3^](#_ENREF_3)) | LR | LR | LR | LR | LR | LR | LR | 6 |
| 4 | C. Bergamaschi et al([^4^](#_ENREF_4)) | LR | LR | LR | LR | LR | LR | LR | 6 |
| 5 | Andrew Somogyi et al([^5^](#_ENREF_5)) | UR | UR | UR | UR | LR | LR | LR | 3 |
| 6 | G. W. Houghton et al([^6^](#_ENREF_6)) | UR | UR | UR | HR | LR | LR | LR | 3 |
| 7 | J. L. Shaffer et al([^7^](#_ENREF_7)) | UR | UR | UR | UR | LR | LR | LR | 3 |
| 8 | Alan H. Lau et al([^8^](#_ENREF_8)) | UR | UR | UR | UR | LR | LR | LR | 3 |
| 9 | C. M. Passmore et al([^9^](#_ENREF_9)) | UR | UR | UR | LR | LR | LR | LR | 4 |
| 10 | Karen I. Plaisance et al([^10^](#_ENREF_10)) | LR | LR | LR | LR | LR | LR | LR | 6 |
| 11 | Lars Heisterberg et al ([^11^](#_ENREF_11)) | UR | UR | UR | LR | LR | LR | LR | 5 |
| 12 | T K Daneshmend et al([^12^](#_ENREF_12)) | UR | UR | UR | LR | LR | LR | LR | 5 |
| 13 | Teow Yee Ti et al([^13^](#_ENREF_13)) | UR | UR | UR | HR | LR | LR | LR | 3 |
| 14 | Steffen Loft et al([^14^](#_ENREF_14)) | UR | UR | UR | LR | LR | LR | LR | 3 |
| 15 | J. Chris Jensen et al([^15^](#_ENREF_15)) | UR | UR | UR | UR | LR | LR | LR | 3 |
| 16 | Karin Dilger et al([^16^](#_ENREF_16)) | UR | UR | UR | LR | LR | LR | LR | 4 |
| 17 | M. N. Muscara et al([^17^](#_ENREF_17)) | UR | UR | UR | LR | LR | LR | LR | 4 |
| 18 | Bilal Ashiq et al([^18^](#_ENREF_18)) | UR | UR | UR | LR | LR | LR | LR | 4 |
| 19 | J. F. Thiercelinet al([^19^](#_ENREF_19)) | LR | LR | LR | LR | LR | LR | LR | 6 |
| 20 | David R. Guay et al([^20^](#_ENREF_20)) | UR | UR | UR | LR | LR | LR | LR | 4 |
| 21 | S. Loft et al([^21^](#_ENREF_21)) | UR | UR | UR | LR | HR | HR | LR | 2 |
| 22 | I Amon et al([^22^](#_ENREF_22)) | UR | UR | UR | UR | UR | LR | LR | 2 |
| 23 | Christoph Dorn et al([^23^](#_ENREF_23)) | LR | UR | UR | LR | LR | LR | LR | 5 |
| 24 | JuriKarjagin et al([^24^](#_ENREF_24)) | UR | UR | UR | HR | LR | LR | LR | 3 |
| 25 | Victor.Montalli et al([^25^](#_ENREF_25)) | UR | UR | UR | UR | LR | LR | LR | 3 |
| 26 | O. Eradiri et al([^26^](#_ENREF_26)) | UR | UR | UR | LR | LR | LR | LR | 5 |
| 27 | D.E Sachwarts et al([^27^](#_ENREF_27)) | UR | UR | UR | LR | HR | HR | LR | 2 |
| 28 | Ingrid Amon et al([^28^](#_ENREF_28)) | UR | UR | UR | UR | HR | HR | LR | 1 |
| 29 | Tom Bergan et al([^29^](#_ENREF_29)) | UR | UR | UR | UR | HR | HR | LR | 1 |
| 30 | Kyoung-Ah Kim et al([^30^](#_ENREF_30)) | LR | UR | UR | LR | LR | LR | LR | 5 |
| 31 | S. Hanifah et al([^31^](#_ENREF_31)) | UR | UR | UR | LR | LR | LR | LR | 4 |
| 32 | I.Lares-Asseff et al([^32^](#_ENREF_32)) | UR | UR | UR | LR | LR | LR | LR | 4 |
| 33 | BengtLjungberg et al([^33^](#_ENREF_33)) | UR | UR | UR | HR | HR | HR | LR | 1 |
| 34 | Geoffrey Farre et al([^34^](#_ENREF_34)) | UR | UR | UR | HR | LR | LR | LR | 3 |
| 35 | S. Loft et al([^35^](#_ENREF_35)) | UR | UR | UR | LR | LR | LR | LR | 4 |
| 36 | A. Somogyi et al([^36^](#_ENREF_36)) | UR | UR | UR | LR | HR | HR | LR | 2 |
| 37 | Hayder A. Kurjiet al([^37^](#_ENREF_37)) | UR | UR | UR | LR | LR | LR | LR | 4 |
| 38 | E. Jager-Roman et al([^38^](#_ENREF_38)) | UR | UR | UR | LR | LR | HR | LR | 3 |
| 39 | Shampa Das et al([^39^](#_ENREF_39)) | UR | UR | UR | LR | LR | LR | LR | 4 |
| 40 | L.F David et al([^40^](#_ENREF_40)) | LR | LR | LR | LR | LR | LR | LR | 7 |
| 41 | Andrew F. Goddard et al([^41^](#_ENREF_41)) | LR | UR | UR | LR | LR | LR | LR | 5 |
| 42 | S. A. Calafattiet al([^42^](#_ENREF_42)) | LR | UR | UR | LR | HR | HR | LR | 3 |
| 43 | G.W. Houghton et al([^43^](#_ENREF_43)) | UR | UR | UR | LR | LR | LR | LR | 4 |
| 44 | O. Obodozieet al([^44^](#_ENREF_44)) | LR | LR | LR | LR | HR | HR | LR | 5 |
| 45 | K. Rajnarayanaet al([^45^](#_ENREF_45)) | UR | UR | UR | LR | HR | HR | LR | 2 |
| 46 | K.Rajnarayana et al([^46^](#_ENREF_46)) | UR | UR | UR | LR | LR | LR | LR | 4 |
| 47 | A. Melande et al([^47^](#_ENREF_47)) | UR | UR | UR | HR | LR | LR | LR | 3 |
| 48 | XinWanget al([^48^](#_ENREF_48)) | UR | UR | UR | LR | LR | LR | LR | 4 |
| 49 | J. S Wang et al([^49^](#_ENREF_49)) | LR | UR | UR | LR | LR | LR | LR | 5 |
| 50 | David Pierceet al([^50^](#_ENREF_50)) | LR | LR | LR | LR | LR | LR | LR | 7 |
| 51 | M. J. Jessa et al([^51^](#_ENREF_51)) | LR | UR | UR | LR | LR | LR | LR | 5 |
| 52 | JyrkiMattila et al([^52^](#_ENREF_52)) | LR | UR | UR | LR | LR | LR | LR | 5 |
| 53 | H. Mattieet al([^53^](#_ENREF_53)) | LR | UR | UR | LR | HR | HR | LR | 3 |
| 54 | J.M.Ventura et al([^54^](#_ENREF_54)) | UR | UR | UR | LR | HR | HR | LR | 2 |
| 55 | O. Hamberg et al([^55^](#_ENREF_55)) | UR | UR | UR | LR | HR | HR | LR | 2 |
| 56 | Kelly A. Sprandelet al([^56^](#_ENREF_56)) | LR | LR | LR | LR | HR | HR | LR | 5 |
| 57 | Y. Wuet al([^57^](#_ENREF_57)) | UR | UR | UR | LR | LR | LR | LR | 5 |
| 58 | Salas‐Herrera et al([^58^](#_ENREF_58)) | UR | UR | UR | LR | HR | HR | LR | 2 |
| 59 | BerganTom et al([^59^](#_ENREF_59)) | UR | UR | UR | LR | LR | LR | LR | 4 |
| 60 | Fredricsson et al([^60^](#_ENREF_60)) | UR | LR | LR | LR | LR | HR | LR | 5 |
| 61 | Roux et al([^61^](#_ENREF_61)) | UR | LR | UR | LR | LR | HR | LR | 4 |
| 62 | Houghton et al([^62^](#_ENREF_62)) | UR | UR | UR | LR | LR | HR | LR | 3 |
| 63 | Loft et al([^63^](#_ENREF_63)) | UR | UR | UR | LR | LR | HR | LR | 3 |
| 64 | Cunningham et al([^64^](#_ENREF_64)) | LR | LR | LR | LR | LR | HR | LR | 6 |
| 65 | Lau et al([^65^](#_ENREF_65)) | LR | LR | UR | LR | LR | HR | LR | 5 |
| 66 | Amon et al([^66^](#_ENREF_66)) | LR | UR | UR | LR | LR | HR | LR | 4 |
| 67 | Alper et al([^67^](#_ENREF_67)) | LR | UR | LR | LR | LR | HR | LR | 5 |

HR: Higher risk, LR: Lower risk, UR: Unclear risk

**PRISMA 2020 Checklist**

| **Section and Topic** | | **Item #** | | **Checklist item** | | **Location where item**  **Is reported** | |
| --- | --- | --- | --- | --- | --- | --- | --- |
| **TITLE** | | | | | |  | |
| Title | | 1 | | Identify the report as a systematic review. | | 1 | |
| **ABSTRACT** | | | | | |  | |
| Abstract | | 2 | | See the PRISMA 2020 for Abstracts checklist. | | 2 | |
| **INTRODUCTION** | | | | | |  | |
| Rationale | | 3 | | Describe the rationale for the review in the context of existing knowledge. | | 4 | |
| Objectives | | 4 | | Provide an explicit statement of the objective(s) or question(s) the review addresses. | | 4-5 | |
| **METHODS** | | | | | |  | |
| Eligibility criteria | | 5 | | Specify the inclusion and exclusion criteria for the review and how studies were grouped for the syntheses. | | 7 | |
| Information sources | | 6 | | Specify all databases, registers, websites, organizations, reference lists and other sources searched or consulted to identify studies. Specify the date when each source was last searched or consulted. | | 5 | |
| Search strategy | | 7 | | Present the full search strategies for all databases, registers and websites, including any filters and limits used. | | 6 | |
| Selection process | | 8 | | Specify the methods used to decide whether a study met the inclusion criteria of the review, including how many reviewers screened each record and each report retrieved, whether they worked independently, and if applicable, details of automation tools used in the process. | | 7 | |
| Data collection process | | 9 | | Specify the methods used to collect data from reports, including how many reviewers collected data from each report, whether they worked independently, any processes for obtaining or confirming data from study investigators, and if applicable, details of automation tools used in the process. | | 7 | |
| Data items | | 10a | | List and define all outcomes for which data were sought. Specify whether all results that were compatible with each outcome domain in each study were sought (e.g. for all measures, time points, analyses), and if not, the methods used to decide which results to collect. | | 7 | |
|  |  | 10b | | List and define all other variables for which data were sought (e.g. participant and intervention characteristics, funding sources). Describe any assumptions made about any missing or unclear information. | | 7 | |
| Study risk of bias assessment | | 11 | | Specify the methods used to assess risk of bias in the included studies, Including details of the tool(s) used, how many reviewers assessed each study and whether they worked independently, and if applicable, details of automation tools used in the process. | | 8 | |
| Effect measures | | 12 | | Specify for each outcome the effect measure(s) (e.g. risk ratio, mean difference) used in the synthesis or presentation of results. | | 8 | |
| Synthesis methods | | 13a | | Describe the processes used to decide which studies were eligible for each synthesis (e.g. tabulating the study intervention characteristics and comparing against the planned groups for each synthesis (item #5)). | | 7 | |
|  |  | 13b | | Describe any methods required to prepare the data for presentation or synthesis, such as handling of missing summary statistics, or data conversions. | | 7 | |
|  |  | 13c | | Describe any methods used to tabulate or visually display results of individual studies and syntheses. | | 7 | |
|  |  | 13d | | Describe any methods used to synthesize results and provide a rationale for the choice(s). If meta-analysis was performed, describe the model(s), method(s) to identify the presence and extent of statistical heterogeneity, and software package(s) used. | | 8 | |
|  |  | 13e | | Describe any methods used to explore possible causes of heterogeneity among study results (e.g. sub group analysis, meta-regression). | | 8 | |
|  |  | 13f | | Describe any sensitivity analyses conducted to assess robustness of the synthesized results. | | 8 | |
| Reporting bias assessment | | 14 | | Describe any methods used to assess risk of bias due to missing results in a synthesis (a rising from reporting biases). | | 8 | |
| Certainty assessment | | 15 | | Describe any methods used to assess certainty (or confidence) in the body of evidence for an outcome. | | 8 | |
|  | **RESULTS** | | | | |  |  |
|  | Study selection | 16a | | Describe the results of the search and selection process, from the number of records identified in the search to the number of studies included in the review, ideally using a flow diagram. | | 10 |  |
|  |  | 16b | | Cite studies that might appear to meet the inclusion criteria, but which were excluded, and explain why they were excluded. | | S1 |  |
|  | Study characteristics | 17 | | Cite each included study and present its characteristics. | | 12-16 |  |
|  | Risk of bias in studies | 18 | | Present assessments of risk of bias for each included study. | | 11 |  |
|  | Results of individual studies | 19 | | For all outcomes, present, for each study: (a) summary statistics for each group (where appropriate) and (b) an effect estimate and its precision (e.g. confidence/credible interval), ideally using structured tables or plots. | | 17-37 |  |
|  | Results of syntheses | 20a | | For each synthesis, briefly summaries the characteristics and risk of bias among contributing studies. | | 38 |  |
|  |  | 20b | | Present results of all statistical syntheses conducted. If meta-analysis was done, present for each the summary estimate and its precision (e.g. confidence/credible interval) and measures of statistical heterogeneity. If comparing groups, describe the direction of the effect. | | 38-52 |  |
|  |  | 20c | | Present results of all investigations of possible causes of heterogeneity among study results. | | 38 |  |
|  |  | 20d | | Present results of all sensitivity analyses conducted to assess the robustness of the synthesized results. | | 38 |  |
|  | Reporting biases | 21 | | Present assessments of risk of bias due to missing results (arising from reporting biases) for each synthesis assessed. | | 11 |  |
|  | Certainty of evidence | 22 | | Present assessments of certainty (or confidence) in the body of evidence for each outcome assessed. | | 11 |  |
|  | **DISCUSSION** | | | | |  |  |
|  | Discussion | | 23a | | Provide a general interpretation of the results in the context of other evidence. | 53-56 |  |
|  |  |  | 23b | | Discuss any limitations of the evidence included in the review. | 56 |  |
|  |  |  | 23c | | Discuss any limitations of the review processes used. | 56 |  |
|  |  |  | 23d | | Discuss implications of the results for practice, policy, and future research. | 56 |  |
|  | **OTHERINFORMATION** | | | | |  |  |
|  | Registration and protocol | | 24a | | Provide registration information for the review, including register name and registration number, or state that the review was not registered. | 58 |  |
|  |  |  | 24b | | Indicate where the review protocol can be accessed, or state that a protocol was not prepared. |  |  |
|  |  |  | 24c | | Describe and explain any amendments to information provided at registration or in the protocol. |  |  |
|  | Support | | 25 | | Describe sources of financial or non-financial support for the review, and the role of the funders or sponsors in the review. | 58 |  |
|  | Competing interests | | 26 | | Declare any competing interests of review authors. | 58 |  |
|  | Availability of data, code and other materials | | 27 | | Report which of the following are publicly available and where they can be found : template data collection forms; data extracted from included studies; data used for all analyses; analytic code; any other materials used in the review. | 57 |  |

**Reference**

1. Fonnes SW, J. J. Holzknecht, B. J. Arpi, M. Rosenberg, J. 2020. The plasma pharmacokinetics of fosfomycin and metronidazole after intraperitoneal administration in patients undergoing appendectomy for uncomplicated appendicitis. Fundam Clin Pharmacol 34:504-512.

2. Sakurai Y, Shiino M, Okamoto H, Nishimura A, Nakamura K, Hasegawa S. 2016. Pharmacokinetics and Safety of Triple Therapy with Vonoprazan, Amoxicillin, and Clarithromycin or Metronidazole: A Phase 1, Open-Label, Randomized, Crossover Study. Adv Ther 33:1519-35.

3. Visser A, Hundt H. 1984. The pharmacokinetics of a single intravenous dose of metronidazole in pregnant patients. Journal of Antimicrobial Chemotherapy 13:279-283.

4. de CBC, Berto LA, Venâncio PC, Cogo K, Franz-Montan M, Motta RH, Santamaria MP, Groppo FC. 2014. Concentrations of metronidazole in human plasma and saliva after tablet or gel administration. J Pharm Pharmacol 66:40-7.

5. Somogyi AA, Kong CB, Gurr FW, Sabto J, Spicer WJ, McLean AJ. 1984. Metronidazole pharmacokinetics in patients with acute renal failure. Journal of Antimicrobial Chemotherapy 13:183-189.

6. Houghton G, Dennis M, Gabriel R. 1985. Pharmacokinetics of metronidazole in patients with varying degrees of renal failure. British journal of clinical pharmacology 19:203-209.

7. Shaffer J, Kershaw A, Houston J. 1986. Disposition of metronidazole and its effects on sulphasalazine metabolism in patients with inflammatory bowel disease. British journal of clinical pharmacology 21:431-435.

8. Lau AH, Evans R, Chang C, Seligsohn R. 1987. Pharmacokinetics of metronidazole in patients with alcoholic liver disease. Antimicrobial agents and chemotherapy 31:1662-1664.

9. Passmore C, McElnay J, Rainey E, D'Arcy P. 1988. Metronidazole excretion in human milk and its effect on the suckling neonate. British journal of clinical pharmacology 26:45-51.

10. Plaisance KI, Quintiliani R, Nightingale CH. 1988. The pharmacokinetics of metronidazole and its metabolites in critically ill patients. Journal of Antimicrobial Chemotherapy 21:195-200.

11. Heisterberg L, Branebjerg PE. 1983. Blood and milk concentrations of metronidazole in mothers and infants.

12. Daneshmend T, Homeida M, Kaye C, Elamin A, Roberts C. 1982. Disposition of oral metronidazole in hepatic cirrhosis and in hepatosplenic schistosomiasis. Gut 23:807-813.

13. Ti TY, Lee HS, Khoo YM. 1996. Disposition of intravenous metronidazole in Asian surgical patients. Antimicrob Agents Chemother 40:2248-51.

14. Loft S, Egsmose C, Sonne J, Poulsen HE, Døssing M, Andreasen PB. 1990. Metronidazole elimination is preserved in the elderly. Human & experimental toxicology 9:155-159.

15. Jensen JC, Gugler R. 1983. Single and multiple dose metronidazole kinetics. Clinical Pharmacology & Therapeutics 34:481-487.

16. Dilger K, Fux R, Röck D, Mörike K, Gleiter CH. 2007. Effect of high-dose metronidazole on pharmacokinetics of oral budesonide and vice versa: a double drug interaction study. J Clin Pharmacol 47:1532-9.

17. Muscará MN, Pedrazzoli J, Jr., Miranda EL, Ferraz JG, Hofstätter E, Leite G, Magalhães AF, Leonardi S, De Nucci G. 1995. Plasma hydroxy-metronidazole/metronidazole ratio in patients with liver disease and in healthy volunteers. Br J Clin Pharmacol 40:477-80.

18. Ashiq B, Usman M, Ashraf M, Omer O, Khokhar M, Saeed-ul-Hassan S. 2011. Comparative pharmacokinetics of metronidazole in healthy volunteers and in patients suffering from amoebiasis. Pak J Pharm 24:41-46.

19. Thiercelin J, Diquet B, Levesque C, Ghesquiére F, Simon P, Viars P. 1984. Metronidazole kinetics and bioavailability in patients undergoing gastrointestinal surgery. Clinical Pharmacology & Therapeutics 35:510-519.

20. Guay D, Meatherall R, Baxter H, Jacyk W, Penner B. 1984. Pharmacokinetics of metronidazole in patients undergoing continuous ambulatory peritoneal dialysis. Antimicrobial agents and chemotherapy 25:306-310.

21. Loft S, Sonne J, Døssing M, Andreasen PB. 1987. Metronidazole pharmacokinetics in patients with hepatic encephalopathy. Scandinavian journal of gastroenterology 22:117-123.

22. Amon I, Amon K, Scharp H, Franke G, Nagel F. 1983. Disposition kinetics of metronidazole in children. European journal of clinical pharmacology 24:113-119.

23. Dorn C, Petroff D, Stoelzel M, Kees MG, Kratzer A, Dietrich A, Kloft C, Zeitlinger M, Kees F, Wrigge H. 2021. Perioperative administration of cefazolin and metronidazole in obese and non-obese patients: a pharmacokinetic study in plasma and interstitial fluid. Journal of Antimicrobial Chemotherapy 76:2114-2120.

24. Karjagin J, Pähkla R, Karki T, Starkopf J. 2005. Distribution of metronidazole in muscle tissue of patients with septic shock and its efficacy against Bacteroides fragilis in vitro. Journal of Antimicrobial Chemotherapy 55:341-346.

25. Montalli VA, de Cassia Bergamaschi C, Ramacciato JC, Nolasco FP, Groppo FC, de Brito Jr RB, Haas DA, Motta RHL. 2012. The effect of smoking on the bioavailability of metronidazole in plasma and saliva. The Journal of the American Dental Association 143:149-156.

26. Eradiri O, Jamali F, Thomson AB. 1987. Steady‐state pharmacokinetics of metronidazole in Crohn's disease. Biopharmaceutics & drug disposition 8:249-259.

27. Schwartz D, Jeunet F. 1976. Comparative pharmacokinetic studies of ornidazole and metronidazole in man. Chemotherapy 22:19-29.

28. Amon I, Amon K, Franke G, Mohr C. 1981. Pharmacokinetics of metronidazole in pregnant women. Chemotherapy 27:73-79.

29. Bergan T, Bjerke PE, Fausa O. 1981. Pharmacokinetics of metronidazole in patients with enteric disease compared to normal volunteers. Chemotherapy 27:233-238.

30. Kim K-A, Park J-Y. 2010. Effect of metronidazole on the pharmacokinetics of fexofenadine, a P-glycoprotein substrate, in healthy male volunteers. European journal of clinical pharmacology 66:721-725.

31. Hanifah S, Mustofa E. Sex Differences on the Metronidazole’s Pharmacokinetic Profile.

32. Lares-Asseff I, Cravioto J, Santiago P, Perez-Ortiz B. Pharmacolunetics of metronidazole in severelv malnourished and nutritionallv rehabihated children.

33. Ljungberg B, Nilsson-Ehle I, Ursing B. 1984. Metronidazole: pharmacokinetic observations in severely ill patients. Journal of Antimicrobial Chemotherapy 14:275-283.

34. Farrell G, Baird-Lambert J, Cvejic M, Buchanan N. 1984. Disposition and metabolism of metronidazole in patients with liver failure. Hepatology 4:722-726.

35. Loft S, Døssing M, Sonne J, Dalhof K, Bjerrum K, Poulsen H. 1988. Lack of effect of cimetidine on the pharmacokinetics and metabolism of a single oral dose of metronidazole. European journal of clinical pharmacology 35:65-68.

36. Somogyi A, Kong C, Sabto J, Gurr F, Spicer W, McLean A. 1983. Disposition and removal of metronidazole in patients undergoing haemodialysis. European journal of clinical pharmacology 25:683-687.
[truncated: 6,852 more chars]
